# Zosteropenillines: Polyketides from the Marine-Derived Fungus *Penicillium thomii*

**DOI:** 10.3390/md15020046

**Published:** 2017-02-17

**Authors:** Shamil Sh. Afiyatullov, Elena V. Leshchenko, Dmitrii V. Berdyshev, Maria P. Sobolevskaya, Alexandr S. Antonov, Vladimir A. Denisenko, Roman S. Popov, Mikhail V. Pivkin, Anatoly A. Udovenko, Evgeny A. Pislyagin, Gunhild von Amsberg, Sergey A. Dyshlovoy

**Affiliations:** 1 G.B. Elyakov Pacific Institute of Bioorganic Chemistry, Far Eastern Branch of Russian Academy of Sciences, 159 Prospect 100-letiya Vladivostoka, Vladivostok 690022, Russia; berdyshev@piboc.dvo.ru (D.V.B.); sobolevskaya_m@mail.ru (M.P.S.); alexanderantonovpiboc@gmail.com (A.S.A.); vladenis@piboc.dvo.ru (V.A.D.); prs_90@mail.ru (R.S.P.); pivkin@piboc.dvo.ru (M.V.P.); pislyagin@hotmail.com (E.A.P.); dyshlovoy@gmail.com (S.A.D.); 2 School of Natural Science, Far Eastern Federal University, 8 Sukhanova St., Vladivostok 690090, Russia; 3 Institute of Chemistry, Far Eastern Branch of Russian Academy of Sciences, 159 Prospect 100-letiya Vladivostoka, Vladivostok 690022, Russia; udovenko@ich.dvo.ru; 4 Laboratory of Experimental Oncology, Department of Oncology, Hematology and Bone Marrow Transplantation with Section Pneumology, Hubertus Wald-Tumorzentrum, University Medical Center, 20246 Hamburg, Germany; g.von-amsberg@uke.de

**Keywords:** marine fungi, *Penicillium thomii*, polyketide decalin derivative, X-ray, ECD spectra

## Abstract

Twelve new polyketides, zosteropenillines A–L (**1**–**12**), together with known polyketide pallidopenilline A (**13**), were isolated from the ethylacetate extract of the fungus *Penicillium thomii* associated with the seagrass *Zostera marina*. Their structures were established based on spectroscopic methods. The absolute configuration of zosteropenilline A (**1**) as 4*R*, 5*S*, 8*S*, 9*R*, 10*R*, and 13*S* was determined by a combination of the modified Mosher’s method, X-ray analysis, and NOESY data. Absolute configurations of zosteropenillines B–D (**2**–**4**) were determined by time-dependent density functional theory (TD-DFT) calculations of ECD spectra. The effect of compounds **1**–**3**, **7**, **8**, **10**, and **11** on the viability of human drug-resistant prostate cancer cells PC3 as well as on autophagy in these cancer cells and inhibitory effects of compounds **1**, **2**, and **8**–**10** on NO production in LPS-induced RAW 264.7 murine macrophages were examined.

## 1. Introduction

**Figure 1:**
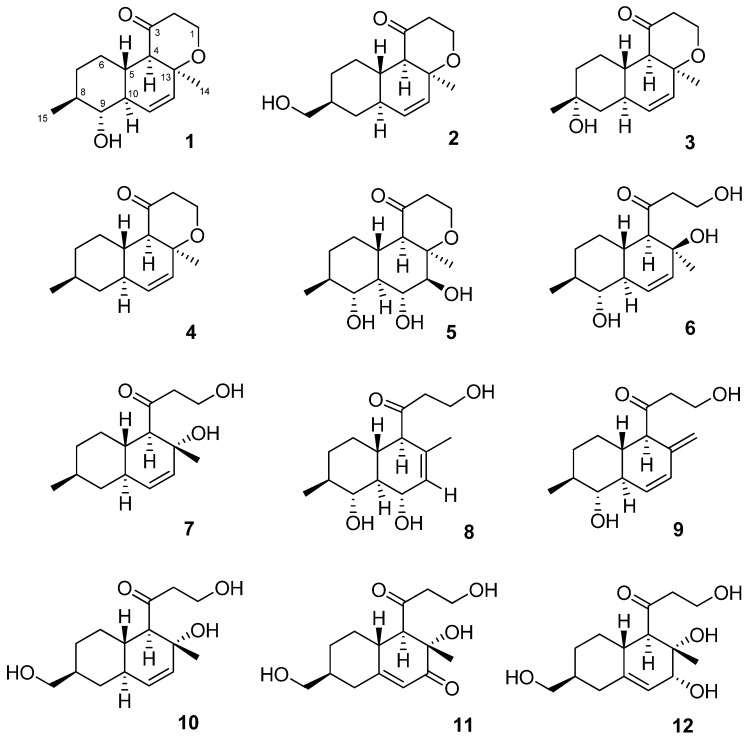
Structures of zosteropenillines A–L (**1**–**12**).

Marine-derived fungi are a prolific source of new secondary metabolites, many of which are biologically active [1,2,3]. In previously published works, we described the isolation and identification of 10 new meroterpenoids–austalides and seven new 6,6-spiroketals–sargassopenillines A–G from fungi *Penicillium thomii* and *P. lividum* associated with the brown alga *Sargassum miyabei* [4,5] and four new eudesmane-type sesquiterpenes–thomimarines A–D from *P. thomii* KMM 4667 associated with the sea grass *Zostera marina* [6]. Recently, we have isolated 11 new polyketides, pallidopenillines that have a polyketidic decalin scaffold from *P. thomii* associated with the brown alga *Sargassum pallidum* [7]. During our ongoing search for new natural compounds from marine-derived fungi, we investigated the strain *P. thomii* KMM 4674 isolated from the seagrass *Z. marina*. Herein, we report the isolation, structure elucidation and biological assay results of 12 new polyketides containing “decalin” motif produced by this fungus (Figure 1).

## 2. Results and Discussion

### 2.1. Structure Elucidation

**Figure 2:**
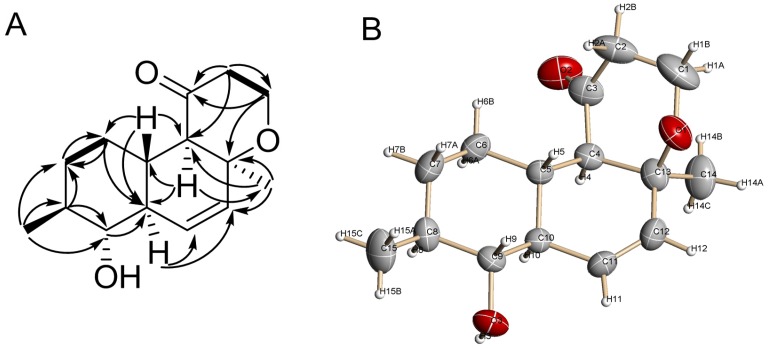
(**A**) Key HMBC and COSY correlations of **1**; (**B**) Crystal structure of **1**.

The molecular formula of **1** was established to be C_15_H_22_O_3_ from a HRESIMS peak at *m*/*z* 273.1461 [M + Na]^+^ and by ^13^C NMR analyses. A close inspection of ^1^H NMR, ^13^C NMR, DEPT, and HSQC data of **1** (Table 1 and Table 2) revealed the presence of two methyl (δ_H_ 1.26, δ_C_ 23.2 and δ_H_ 1.03, δ_C_ 18.4) groups, four methylenes (δ_C_ 39.9, 32.7, 28.7), including one oxygen-bearing (δ_H_ 4.11, 3.89, δ_C_ 60.9), and seven methines (δ_H_ 1.89, δ_C_ 37.5, δ_H_ 1.41, δ_C_ 40.2, δ_H_ 1.65, δ_C_ 48.6, δ_H_ 2.09, δ_C_ 62.8, δ_H_ 6.24, δ_C_ 130.1, δ_H_ 5.69, δ_C_ 131.6), together with one oxygen-bearing (δ_H_ 2.92, δ_C_ 77.9), one carbonyl carbon (δ_C_ 210.2), and one oxygenated quaternary carbon (δ_C_ 74.7). These data and five unsaturated degrees from the molecular formula suggested that compound **1** possessed three rings. COSY-45 data and HMBC correlations (Figure 2) from H-4 (δ_H_ 2.09) to C-5 (δ_C_ 37.5), C-10 (δ_C_ 48.6), C-14 (δ_C_ 23.2), from H-5 (δ_H_ 1.89) to C-4 (δ_C_ 62.8), C-6 (δ_C_ 28.7), C-10, C-13 (δ_C_ 74.7), from H_2_-6 (δ_H_ 1.28, 1.14) to C-5, C-7 (δ_C_ 32.7), C-8 (δ_C_ 40.2) and C-10, from H-7a (δ_H_ 1.78) to C-5, C-6, C-8 and C-15 (δ_C_ 18.4), from H-7b (δ_H_ 1.08) to C-9 (δ_C_ 77.9), from H-8 (δ_H_ 1.41) to C-7, C-9 and C-10, from H_3_-15 (δ_H_ 1.03) to C-7, C-8 and C-9, from H-9 (δ_H_ 2.92) to C-10, C-11 (δ_C_ 130.1), C-15, from H-10 (δ_H_ 1.65) to C-11, C-12 (δ_C_ 131.6), from H-11 (δ_H_ 6.24) to C-13, from H-12 (δ_H_ 5.69) to C-4, C-13, and C-14 revealed the presence of a decalin moiety and established a Δ^11^ double bond and the location of methyl groups at C-8 and C-13 in **1**.

HMBC correlations from H-1a (δ_H_ 4.11) to C-2 (δ_C_ 39.9), C-3 (δ_C_ 210.2) and C-13, from H-2a (δ_H_ 2.64) to C-1 (δ_C_ 60.9), C-3, from H-2b (δ_H_ 2.21) to C-3, C-4 established a saturated γ-pyrone derivative moiety in **1**. NOESY cross-peaks H_3_-14 (δ_H_ 1.26)/H-4 (δ_H_ 2.09), H-1β (δ_H_ 3.89); H_3_-15 (δ_H_ 1.03)/H-9 (δ_H_ 2.92); H-5/H-2β (δ_H_ 2.64), H-6β (δ_H_ 1.28), H-7β (δ_H_ 1.08), H-9; H-10 (δ_H_ 1.65)/H-6α (δ_H_ 1.14), H-8 (δ_H_ 1.41), H-4; H-9/H_3_-15, H-5 and H-4/H-6α, H-10, H_3_-14 indicated a *trans*-ring fusion of the A and B rings, *cis*-ring fusion of the B and C rings, the α-orientation of H_3_-14 and 9-OH, and the β-orientation of H_3_-15. The planar structure and relative configuration of **1** were unequivocally confirmed by X-ray analysis, which was carried out for a single crystal obtained by recrystallization from acetonitrile–water (Figure 2 and Figure S9).

**Figure 3:**
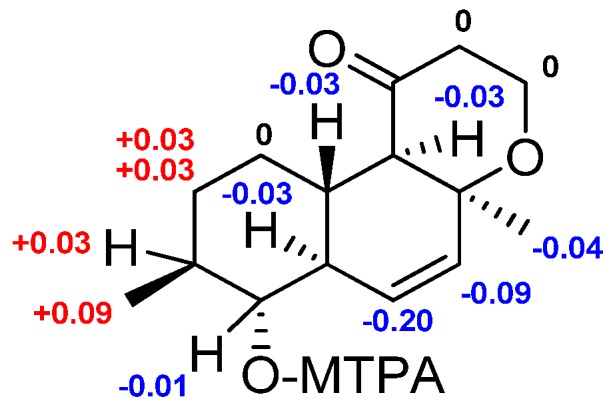
Δδ (δ_S_ − δ_R_) values (in ppm) for the (*S*)- and (*R*)-MPTA esters of **1**.

Esterification of **1** with (*R*)- and (*S*)-MTPA chloride occurred at the C-9 hydroxy group to give the (*S*)- and (*R*)-MTPA esters **1a** and **1b**, respectively. The observed chemical shift differences Δδ (δ*_S_* − δ*_R_*) (Figure 3) indicated the 9*R* configuration. These data and observed NOESY correlations determined the absolute stereostructure of **1** as 4*R*, 5*S*, 8*S*, 9*R*, 10*R*, and 13*S*. Compound **1** was named zosteropenilline A.

The molecular formula of **2** was established to be C_15_H_22_O_3_ from a HRESIMS peak at *m*/*z* 273.1474 [M + Na]^+^ and by ^13^C NMR analyses. The general features of the ^13^C NMR of **2** resembled those of **1** with the exception of the C-9–C-11 and C-15 carbon signals. COSY and HSQC spectra of **2** revealed the partially connectivity sequence of the protons in the A ring as CH_2_(15)-CH(8)-CH_2_(9)-CH(10). These data and HMBC correlations from H-9b (δ_H_ 0.93) to C-7 (δ_C_ 29.1), C-8 (δ_C_ 40.5), C-10 (δ_C_ 41.4), C-11 (δ_C_ 134.4), and C-15 (δ_C_ 68.1); and from H_2_-15 (δ_H_ 3.50, 3.47) to C-7, C-8 and C-9, established the A ring structure, lacking the hydroxy group at C-9, and indicated the presence of a hydroxymethyl group at C-8 in **2**. The NOESY cross-peaks H-4 (δ_H_ 2.07)/H-10 (δ_H_ 1.74), H_3_-14 (δ_H_ 1.26); H-8 (δ_H_ 1.63)/H-9α (δ_H_ 1.91), H-10; H-5 (δ_H_ 1.78)/H-9*β* (δ_H_ 0.93); H-9β/H_2_-15 (δ_H_ 3.50, 3.47) indicated the *trans*-ring fusion of the A and B ring, *cis*-ring fusion of the B and C ring, and the β-orientation of hydroxymethyl group at C-8. Compound **2** was named zosteropenilline B.

The molecular formula of **3** was established to be C_15_H_22_O_3_ from a HRESIMS peak at *m*/*z* 273.1463 [M + Na]^+^ and by ^13^C NMR analyses. The ^1^H and ^13^C NMR spectra (Table 1 and Table 2) for this compound were very similar to those obtained for zosteropenilline B (**2**) with the exception of the C-6–C-10 and C-15 carbon and proton signals. The HMBC correlations H-9a (δ_H_ 1.75)/C-8 (δ_C_ 69.7), C-10 (δ_C_ 36.9); H_3_-15 (δ_H_ 1.25)/C-7 (δ_C_ 24.9), C-8, C-9 (δ_C_ 44.5) established the structure of **3** including the hydroxy and methyl groups at the C-8 position. The relative configuration of **3** was defined based on the observed NOESY correlations (Figure S27). Compound **3** was named zosteropenilline C. 

The molecular formula of **4** was established to be C_15_H_22_O_2_ from a HRESIMS peak at *m*/*z* 257.1510 [M + Na]^+^ and by ^13^C NMR analyses. The ^1^H and ^13^C NMR data (Table 1 and Table 2) observed for **4** closely resembled those obtained for zosteropenilline B (**2**) with the exception of the C-7–C-9 and C-15 carbon and proton signals. The mutual correlations from H_3_-15 to C-7 and C-9 established the structure of the A ring and location methyl group at C-8. Compound **4** was named zosteropenilline D.

**Figure 4:**
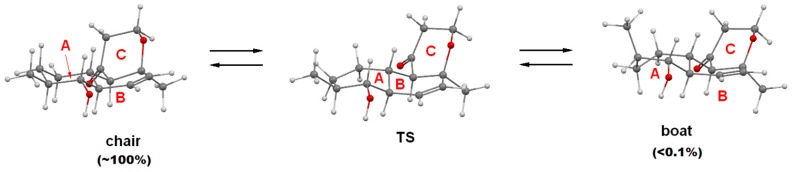
The inversion of A-ring 4*R*, 5*S*, 8*S*, 9*R*, 10*R*, 13*S* of **1**.

**Figure 5:**
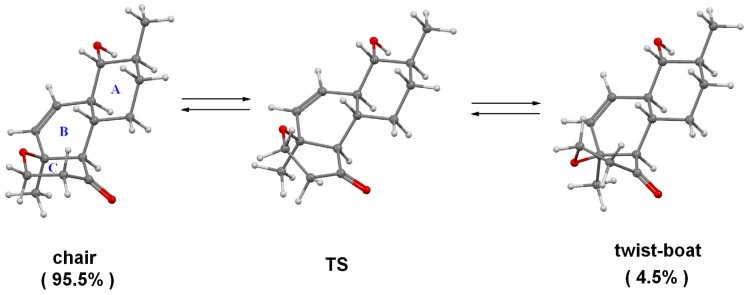
The inversion of C-ring for 4*R*, 5*S*, 8*S*, 9*R*, 10*R*, 13*S* of **1**.

The absolute configurations of zosteropenillines B–D (**2**–**4**) were determined on the basis of the ECD spectroscopy data. The geometry of the stable conformations of compounds **1**–**4** were optimized using general procedure, described in the experimental section of the paper. The internal rotations of the hydroxyl groups as well as the inversions of the six-membered rings A and C were accounted for during fulfilled conformational analysis. We found that for all these compounds ring C can exist in two stable conformations: “chair” and “boat”. The “chair” conformation is more stable for about ΔE^#^ (chair → boat) ≈ 2 kcal·mol^−1^ and the barrier for interconversion between conformations is ΔE^#^ (chair → boat) ≈ 6 kcal·mol^−1^. Analogously, ring A can also exist in “chair” and “boat” conformations. The “chair” conformation is more stable for about ΔE^#^ (chair → boat) ≈ 10 kcal·mol^−1^ and the barrier for interconversion between conformations is ΔE^#^ (chair → boat) ≈ 14 kcal·mol^−1^ (Figure 4 and Figure 5).

**Figure 6:**
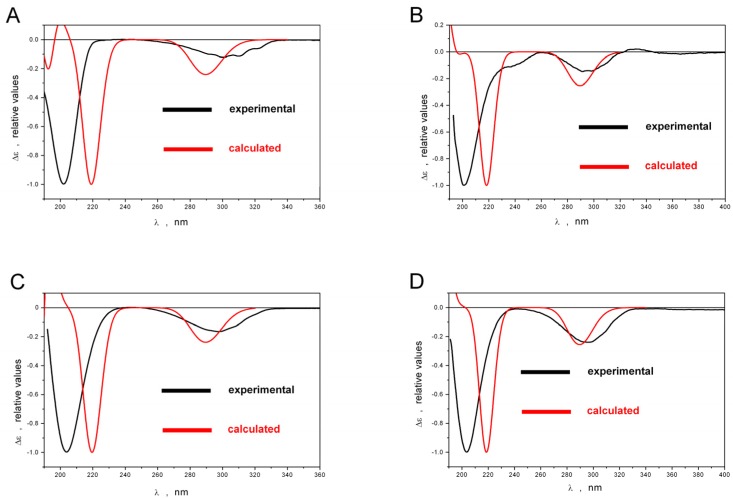
The normalized experimental and statistically averaged ECD spectra. (**A**) 4*R*, 5*S*, 8*S*, 9*R*, 10*R*, 13*S*-**1**; (**B**) 4*R*, 5*S*, 8*S*, 10*R*, 13*S*-**2**; (**C**) 4*R*, 5*S*, 8*R*, 10*R*, 13*S*-**3**; (**D**) 4*R*, 5*S*, 8*S*, 10*R*, 13*S*-**4**. The half-width bands ΔE_excitation_ = 0.20 eV were used for the simulations of individual bands in theoretical spectra for each conformer.

To obtain theoretical ECD spectra the excitation energies and the rotatory strengths for **1**–**4** were calculated using time-dependent density functional theory (TDDFT) with PBE1PBE exchange-correlation functional and cc-pvTz basis set [8]. A comparison of statistically averaged ECD spectra for **1**–**4** with corresponding experimental spectra is presented in Figure 6. The comparison of all theoretical spectra with the experimental ones showed that all spectra are qualitatively similar in the region λ ≥ 200 nm, where the pronounced Cotton effects occur, thus proving 4*R*, 5*S*, 8*S*, 9*R*, 10*R*, 13*S* absolute configuration for **1**; 4*R*, 5*S*, 8*S*, 10*R*, 13*S* for **2**; 4*R*, 5*S*, 8*R*, 10*R*, 13*S* for **3**; 4*R*, 5*S*, 8*S*, 10*R*, 13*S* for **4**.

**Figure 7:**
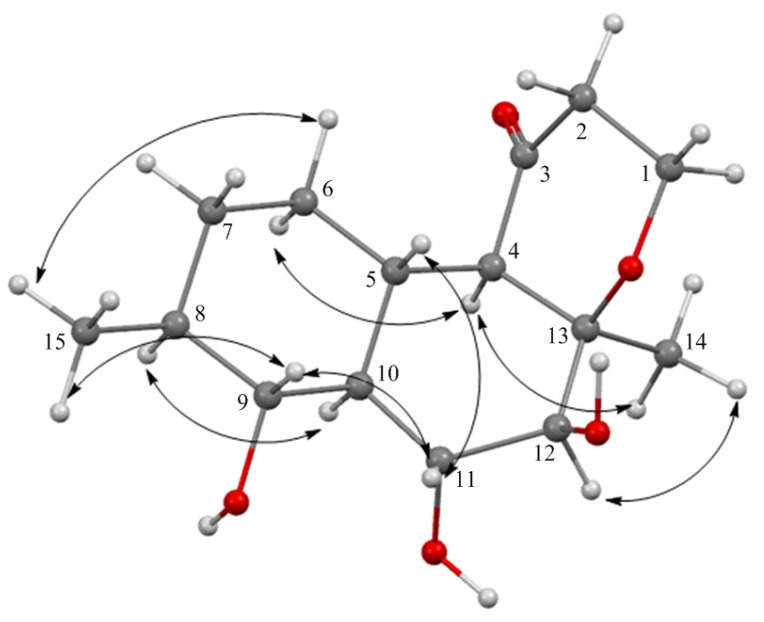
Energy-minimized 3D model of **5** with key NOESY correlations.

The molecular formula of **5** was established to be C_15_H_24_O_3_ from a HRESIMS peak at *m*/*z* 283.1538 [M − H]^−^ and by ^13^C NMR analyses. The COSY-45 data and HMBC correlations from H-4 (δ_H_ 2.11) to C-5 (δ_C_ 38.5), C-6 (δ_C_ 29.0), C-10 (δ_C_ 53.5), from H-5 (δ_H_ 1.95) to C-6, C-10, from H-6a (δ_H_ 1.33) to C-4 (δ_C_ 63.7), C-7 (δ_C_ 31.8) and C-10, from H-7a (δ_H_ 1.73) to C-5, C-9 (δ_C_ 77.2), from H_3_-15 (δ_H_ 1.06) to C-7, C-8 (δ_C_ 39.2), C-9, from H-9 (δ_H_ 3.29) to C-5, C-11 (δ_C_ 67.3), from H-10 (δ_H_ 1.61) to C-5, C-9, C-11, from H-11 (δ_H_ 4.38) to C-12 (δ_C_ 72.7), and from H_3_-14 (δ_H_ 1.36) to C-4, C-12, C-13 (δ_C_ 77.5) revealed the presence of a decalin moiety in **1** and established the location of methyl groups at C-8 and C-13 and hydroxy groups at C-9, C-11 and C-12. HMBC correlations from H-1a (δ_H_ 4.22) to C-2 (δ_C_ 37.9), C-3 (δ_C_ 206.0) and C-13, from H-2a (δ_H_ 2.67) to C-1 (δ_C_ 60.9), C-3, from H-4 (δ_H_ 2.11) to C-2 and C-3 established a saturated γ-pyrone derivative in **5** (Figure S40). The relative configuration of **5** was assigned based on NOESY cross-peaks (Figure 7) and ^1^H-^1^H coupling constants (Table 2). Observed NOESY correlations and magnitudes of the vicinal coupling constants between H-4 and H-5; H-5 and H-10; H-9 and H-10; H-10 and H-11; H-11 and H-12 indicated a *trans*-ring fusion of the A and B rings, *cis*-ring fusion of the B and C rings, the *α*-orientation of H_3_-14, 9-OH, 11-OH groups and the β-orientation of H_3_-15 and 12-OH groups. Compound **5** was named zosteropenilline E.

The molecular formula of **6** was established to be C_15_H_24_O_4_ from a HRESIMS peak at *m*/*z* 291.1579 [M + Na]^+^ and by ^13^C NMR analyses. The ^13^C NMR data of **6** matched those for **1** with the exception of the C-1–C-3 and C-13–C-14 carbon signals. The structure of the decalin moiety and location of the methyl groups at C-8 and C-13 and oxygen functions at C-9 and C-13 were established as for zosteropenilline A (**1**) based on COSY and HMBC correlations. The COSY-45 data and HMBC correlations from H_2_-1 (δ_H_ 3.91) to C-2 (δ_C_ 48.6) and C-3 (δ_C_ 217.6), from H_2_-2 (δ_H_ 2.86, 2.76) to C-1 (δ_C_ 57.6) and C-3, and from H-4 (δ_H_ 2.59) to C-3 indicated the presence of a 3-hydroxy-1-oxopropyl residue (C-1–C-3 residue numbering) at C-4. The relative configuration of **6** was assigned on the basis of a NOESY experiment and ^1^H-^1^H coupling constants (Table 2). Compound **6** was named zosteropenilline F. Zosteropenilline F is an epimer of the pallidopenilline A (**13**) which was earlier isolated from the *P. thomii* associated with the brown alga *Sargassum pallidum* [7]. The ^1^H and ^13^C NMR data observed for **6** closely resembled those obtained for pallidopenilline A (measured in CDCl_3_) (Figures S58–S68) with the exception of the C-4, C-13 and C-14 carbon and proton signals The molecular formula of **7** was established to be C_15_H_24_O_3_ from a HRESIMS peak at *m*/*z* 275.1621 [M + Na]^+^ and by ^13^C NMR analyses. The ^13^C NMR data (Table 3) observed for **7** closely resembled those obtained for zosteropenilline D (**4**) with the exception of the C-1–C-3 and C-13–C-14 carbon signals. The structure of the decalin moiety, location of the methyl groups at C-8 and C-13, oxygen functions at C-13 and 3-hydroxy-1-oxopropyl residue at C-4 were established by HMBC and COSY correlations (Figures S74 and S75). The relative configuration of **7** was assigned based on NOESY correlations H-4 (δ_H_ 2.89)/H-10 (δ_H_ 1.84); H-10/H-8 (δ_H_ 1.46) and H-5 (δ_H_ 1.48)/H_3_-14 (δ_H_ 1.20) and H_3_-15 (δ_H_ 0.89) recorded in CDCl_3_ and 13-OH (δ_H_ 4.95)/H-4 correlation in spectrum of **7** recorded in DMSO-*d*_6_ (Figure S77). Compound **7** was named zosteropenilline G.

The molecular formula of **8** was established to be C_15_H_24_O_4_ from a HRESIMS peak at *m*/*z* 291.1571 [M + Na]^+^ and by ^13^C NMR analyses. The ^1^H NMR, ^13^C NMR, DEPT and HSQC spectra of **8** (Table 3 and Table 4) displayed obvious signals for two methyl (δ_H_ 1.60, δ_C_ 20.9 and δ_H_ 1.02, δ_C_ 17.8) groups, four methylenes (δ_C_ 43.6, 30.5, 31.9) including one oxygen-bearing (δ_H_ 3.85, 2H, δ_C_ 57.9), seven methines (δ_H_ 1.69, δ_C_ 37.9, δ_H_ 1.47, δ_C_ 39.2, δ_H_ 1.28, δ_C_ 49.6, δ_H_ 2.92, δ_C_ 61.7, δ_H_ 3.25, δ_C_ 82.3, δ_H_ 4.32, δ_C_ 73.2, δ_H_ 5.53, δ_C_ 129.0,) including two oxygen-bearing, one carbonyl carbon (δ_C_ 214.0) and one *sp^3^* quaternary carbon (δ_C_ 131.5). The structure of a 1,2,3,4-tetrasubstituted cyclohexane ring and side chain in **8** were found by extensive NMR spectroscopy (^1^H and ^13^C NMR, COSY, HSQC and HMBC) as for zosteropenillines E (**5**) and F (**6**). HMBC correlations from H-4 (δ_H_ 2.92) to C-5 (δ_C_ 37.9), C-12 (δ_C_ 129.0), C-13 (δ_C_ 131.5), from H-10 (δ_H_ 1.28) to C-5, C-11 (δ_C_ 73.2), C-12, from H-11 (δ_H_ 4.32) to C-12, C-13, and from H_3_-14 (δ_H_ 1.60) to C-4 (δ_C_ 61.7), C-12, C-13 established a B ring structure, Δ^12^ double bond and the location of the hydroxy group at C-11. The relative configuration of **8** was assigned based on NOESY cross-peaks H-4/H-10; H-5 (δ_H_ 1.69)/H-9 (δ_H_ 3.25), H-11, H_3_-15 (δ_H_ 1.02); H-8 (δ_H_ 1.47)/H-10; H-9/H-11 and ^1^H-^1^H coupling constants (Table 4). Compound **8** was named zosteropenilline H.

The molecular formula of **9** was established to be C_15_H_22_O_3_ from a HRESIMS peak at *m*/*z* 273.1462 [M + Na]^+^ and by ^13^C NMR analyses. Structures of an A ring and side chain in **9** were established as for zosteropenillines E (**5**) and F (**6**). Observed long-range COSY correlations H-4 (δ_H_ 3.15)/H_2_-14 (δ_H_ 4.99, 4.59), H-12 (δ_H_ 6.20)/H-14b. and HMBC correlations from H-4 (δ_H_ 3.15) to C-13 (δ_C_ 140.8), C-14 (δ_C_ 112.8), from H-9 (δ_H_ 2.89) and H-10 (δ_H_ 1.85) to C-11 (δ_C_ 130.6) and from H_2_-14 (δ_H_ 4.99, 4.59) to C-4 (δ_C_ 59.6), C-12 (δ_C_ 129.3), C-13 indicated the presence of a diene system at the C-11 and C-13(14) in **9**. The relative configuration of **9** was assigned based on the NOESY correlations H-4/H-10; H-8 (δ_H_ 1.43)/H-10 and H-5 (δ_H_ 1.68)/H-9, H_3_-15 (δ_H_ 1.05). Compound **9** was named zosteropenilline I.

The molecular formula of **10** was established to be C_15_H_24_O_4_ from a HRESIMS peak at *m*/*z* 291.1573 [M + Na]^+^ and by ^13^C NMR analyses. The ^13^C NMR data (Table 3) observed for **10** matched the data reported for zosteropenilline G (**7**) with the exception of the C-7–C-9 and C-15 carbon signals. These data, together with the molecular mass difference of 16 mass units between **7** and **10**, indicated the presence of the hydroxymethyl group at C-8 in **10** instead of a methyl group. NOESY cross-peaks H-4 (δ_H_ 2.92)/H-6b (δ_H_ 0.95), H-10 (δ_H_ 1.87); H-5 (δ_H_ 1.52)/H-7b (δ_H_ 1.02), H-9b (δ_H_ 0.82), H_3_-14 (δ_H_ 1.20); H-7b/H-9b, H_2_-15 (δ_H_ 3.47, 3.45); H-8 (δ_H_ 1.61)/H-6b, H-10 indicated the *trans*-ring fusion of the A and B rings, a β-orientation of the side chain, H_3_-14 and hydroxymethyl group at C-8. Compound **10** was named zosteropenilline J.

The molecular formula of **11** was established to be C_15_H_22_O_5_ from a HRESIMS peak at *m*/*z* 305.1370 [M + Na]^+^ and by ^13^C NMR analyses. The UV spectrum exhibits a λ_max_ at 242 nm (log ε 3.46), consistent with the enone system in **11**. The COSY and HSQC spectra of **11** revealed the partial connectivity sequences of the protons in the A ring as CH(5)-CH_2_(6)-CH_2_(7)-CH(8) and CH_2_(15)-CH(8)-CH_2_(9). These data and HMBC correlations from H-9a (δ_H_ 2.60) to C-7 (δ_C_ 27.9), C-8 (δ_C_ 41.1) and C-15 (δ_C_ 67.4), from H-9b (δ_H_ 2.01) to C-10 (δ_C_ 166.3), from H_2_-15 (δ_H_ 3.58, 3.54) to C-7, C-8 and C-9 (δ_C_ 38.3) established the structure of A ring. Long range correlations from H-4 (δ_H_ 3.01) to C-5 (δ_C_ 39.4), C-10, C-12 (δ_C_ 200.3) and C-13 (δ_C_ 74.2), from H-11 (δ_H_ 5.90) to C-5 and C-13 and from H_3_-14 (δ_H_ 1.19) to C-4 (δ_C_ 61.6), C-12, C-13 determined the structure of B ring and indicated the 10-en-12-one position for the enone chromophore in **11**. The structure of side chain in **11** was found by NMR spectroscopy as for zosteropenilline F (**6**). Observed NOESY correlations H-5 (δ_H_ 2.89)/H-9b (δ_H_ 2.01), H_3_-14; H_2_-15/H-9b indicated these protons to be on the same side of the molecule. These data and the magnitude of the coupling constant of the H-4 signal (δ_H_ 3.01, *J* = 9.8 Hz) indicated H-4 and 13-OH to be on the other side of molecule. Compound **11** was named zosteropenilline K.

The molecular formula of **12** was established to be C_15_H_24_O_5_ from a HRESIMS peak at *m*/*z* 307.1501 [M + Na]^+^ and by ^13^C NMR analyses. The structure of the A ring in **12** was established based on COSY and HMBC correlations as for zosteropenilline K (**11**). HMBC correlations from H-11 (δ_H_ 5.60) to C-5 (δ_C_ 40.3), C-9 (δ_C_ 37.2), C-12 (δ_C_ 68.0), C-13 (δ_C_ 72.4), from H-12 (δ_H_ 4.36) to C-4 (δ_C_ 57.7), C-10 (δ_C_ 144.5), C-11 (δ_C_ 118.8), C-13, and from H_3_-14 (δ_H_ 1.20) to C-4, C-12, C-13 established the structure of B ring, location oxygenated functions at C-12, C-13 and methyl group at C-13 in **12**. The relative configuration of **12** was defined based on NOESY correlations H-5/H_3_-14; H_3_-14/H-12; H-4/H-6b; H-6b/H-8 and the magnitude of the coupling constant of the H-4 signal (δ_H_ 3.09, *J* = 10.0 Hz). Compound **12** was named zosteropenilline L. Besides the new zosteropenillines (**1**–**12**), a known pallidopenilline A (**13**) was also isolated from this fungus. For the first time, pallidopenilline A was found in fungus *P. thomii* KMM 4675 [7].

### 2.2. Bioassay Results

**Figure 8:**
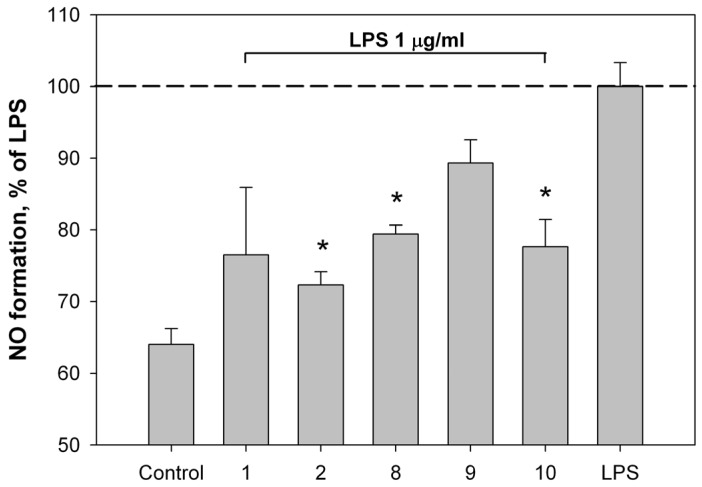
Effect of compounds **1**, **2**, and **8**–**10** on NO level in RAW 264.7 murine macrophages co-incubated with LPS from *E. coli*. The compounds were tested at a concentration of 10 µM. Time of cell incubation with compounds is 24 h at 37 °C. * *p* < 0.01.

Compounds **2**, **8**, and **10** induced a moderate downregulation of NO production in LPS-stimulated macrophages at non-cytotoxic concentration of 10.0 µM. Respectively, NO levels in these cells were decreased by 27.7 ± 1.8, 20.6 ± 1.2, and 22.3 ± 3.8 percent, in comparison with LPS-pretreated control cells. The most effective substance was compound **2**, which exhibited the maximal pronounced inhibition of NO formation in lipopolysaccharide-stimulated RAW 264.7 cells (Figure 8). 

**Figure 9:**
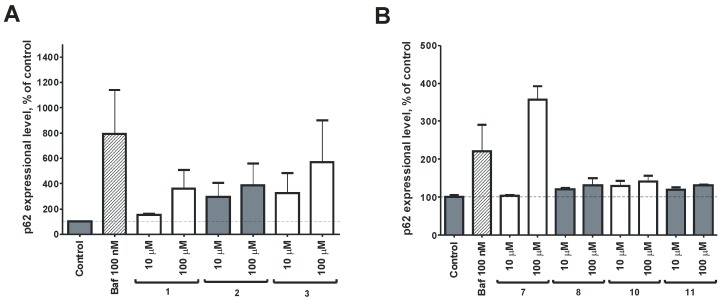
The effect on p62 expression. The expression of p62 in PC3 cells treated with the compounds **1**–**3** (**A**) and **7**, **8**, **10**, **11** (**B**). Cells were treated for 48 h, then proteins were extracted and both p62 and α-tubulin levels were detected by Western blotting. The signals’ intensity was quantified with Quantity One. Cells treated with the autophagy inhibitors bafilomycin A1 (Baf, 100 nM) were used as a positive control. The values are presented as mean ± SEM.

We have investigated the effect of the isolated zosteropenillines **1**–**3**, **7**, **8**, **10**, and **11** on the viability of human drug-resistant prostate cancer cells PC3 as well as on autophagy in these cancer cells. Induction of autophagy is a well-characterized phenomenon in prostate cancer, frequently associated with cancer cell survival and resistance to therapy [9,10]. Therefore, novel compounds that effectively inhibit prosurvival autophagy are of high interest [10,11]. p62 (or SQSTM) is a cargo protein that targets and binds other proteins to deliver them to autophagosomes for selective autophagy. During autophagy autophagosomes fuse with lysosomes and intra-autophagosomal components are degraded by lysosomal hydrolases. Therefore, an increased level of p62 has previously been associated with inhibition of autophagy flux. In our experiments MTT analysis revealed all compounds to be non-cytotoxic up to 100 µM (data not shown). Consequently, we investigated the effect of the zosteropenillines on the expression of autophagy-related cargo protein p62. Western blotting analysis revealed a slight increase in p62 levels in PC3 cells treated with all the investigated substances (Figure 9), suggesting the drug-induced inhibition of autophagy. The effect on p62 expression was similar to the effect of Bafilomycin A1 (Baf)—a well-established autophagy inhibitor. These results suggest that the investigated compounds are able to inhibit autophagy at non-cytotoxic concentrations and may sensitize human cancer cells to cytotoxic anticancer drugs.

## 3. Materials and Methods

### 3.1. General Experimental Procedures

Optical rotations were measured on a Perkin-Elmer 343 polarimeter (Perkin Elmer, Waltham, MA, USA) in MeOH. UV spectra were recorded on a Specord UV VIS spectrometer (Carl Zeiss, Jena, Germany) in MeOH and in *n*-hexane. IR spectra were determined on a Specord M 82 (Carl Zeiss) in CHCl_3_. ECD spectra were measured with a Chirascan-Plus CD Spectrometer (Leatherhead, UK) in MeOH in *n*-hexane. X-ray crystallographic analysis was carried out on a Bruker Kappa APEX2 diffractometer with graphic-monochromated Mo K_α_ radiation. ^1^H and ^13^C NMR spectra were recorded in CDCl_3_ and in DMSO-*d*_6_ on a Bruker Avance-500 (Bruker BioSpin GmbH, Rheinstetten, Germany) and Avance III-700 (Bruker BioSpin GmbH, Rheinstetten, Germany) spectrometers operating at 500.13 MHz and 125.77 MHz and 700.13 and 176.04 MHz, respectively, using TMS as an internal standard. HRESIMS spectra were obtained on an Agilent 6510 Q-TOF LC mass spectrometer (Agilent Technologies, Santa Clara, CA, USA). Low-pressure liquid column chromatography was performed using Si gel L (50/100 μm, Sorbpolimer, Imid, Krasnodar, Russia). Plates (4.5 × 6.0 cm) pre-coated with Si gel (5–17 μm, Sorbfil, Imid, Krasnodar, Russia) were used for thin layer chromatography. Preparative HPLC was carried out on a Shimadzu LC-20 (Shimadzu, USA Manufacturing, Canby, OR, USA) and Agilent 1100 (Agilent Technologies, Ratingen, Germany) chromatographs using an YMC ODS-AM (YMC Co, Ishikawa, Japan) (5 μm, 10 × 250 mm), YMC Si (YMC Co, Ishikawa, Japan) (5 μm, 10 × 250 mm), Supelco Discovery C-18 (Supelco Analytical, Bellefonte, PA, USA) (5 μm, 4.6 × 250 mm), Ultrasphere Si (Beckman Coulter Inc., Brea CA, USA) (5 μm, 4.6 × 250 mm) and Kromasil 3-CelluCoat (Kromasil, Bohus, Sweden) (4.6 × 150 mm) columns and Shimadzu RID-20A (Shimadzu Corporation, Kyoto, Japan) and Agilent 1100 (Agilent Technologies, Ratingen, Germany) refractometers.

### 3.2. Fungal Strain

The strain of the fungus *Penicillium thomii* KMM 4674 was isolated from superficial mycobiota of the rhizome sea-grass *Zostera marina* (Sea of Japan) and was identified on the basis of morphological evaluation. 

### 3.3. Cultivation of P. thomii

The fungus was grown stationary at 22 °C for 21 days in 20 Erlenmeyer flasks (500 mL), each containing 20 g of rice, 20 mg of yeast extract, 10 mg of KH_2_PO_4_, and 40 mL of natural sea water (Marine Experimental Station of G.B. Elyakov Pacific Institute of Bioorganic Chemistry, Troitsa (Trinity) Bay, Sea of Japan) by Dr. Natalya Kirichuk. 

### 3.4. Extraction and Isolation

At the end of the incubation period, the mycelia and medium were homogenized and extracted with EtOAc (1 L). The obtained extract was concentrated to dryness. The residue (2.46 g) was dissolved in H_2_O–EtOH (4:1) (100 mL) and was extracted with *n*-hexane (0.2 L × 3) and EtOAc (0.2 L × 3). After evaporation of the EtOAc layer, the residual material (2.26 g) was passed over a silica column (3 × 14 cm), which was eluted first with *n*-hexane (200 mL) followed by a step gradient from 5% to 50% EtOAc in *n*-hexane (total volume: 5 L). Fractions of 250 mL were collected and combined on the basis of TLC (Si gel, toluene–isopropanol 6:1 and 3:1, *v*/*v*).

The *n*-hexane–EtOAc (75:25) eluate (146 mg) was purified on an YMC ODS-AM column eluting with MeOH–H_2_O (80:20) to yield fractions A (15.4 mg), B (10 mg), C (2.3 mg), D (3.3 mg), and individual compound **7** (8.4 mg). Fraction A was purified on a Supelco C-18 eluting with CH_3_CN–H_2_O (25:75) to yield **3** (2.3 mg). Fraction B was purified on a Kromasil 3-CelluCoat column eluting with CH_3_CN–H_2_O (25:75) to yield **4** (3.0 mg). Fraction C was purified on an Ultrasphere Si column eluting with EtOAc–*n*-hexane–EtOH (50:50:5) to yield **9** (1.8 mg). Fraction D was purified on an Ultrasphere Si eluting with EtOAc–*n*-hexane–EtOH (100:100:5) to yield **5** (0.5 mg). The *n*-hexane–EtOAc (65:35) eluate (283 mg) was purified on a YMC ODS-AM column eluting with MeOH–H_2_O (65:35) to yield **1** (11 mg), **6** (3.0 mg), **8** (6.1 mg), **10** (34.6 mg), **11** (4.6 mg) and **13** (100 mg). The *n*-hexane–EtOAc (60:40) eluate (200 mg) was purified on a YMC ODS-AM column eluting with MeOH–H_2_O (65:35) to yield **2** (83 mg) and on a Supelco C-18 eluting with CH_3_CN–H_2_O (80:20) to yield **12** (1.6 mg).

### 3.5. Spectral Data

*Zosteropenilline A* (**1**): colorless crystals; ${\left[\alpha \right]}_{D}^{20}$ −125.0 (*c* 0.1, MeOH); UV (*n*-hexane) low intensity band; CD (*c* 0.05, *n*-hexane), λ_max_ (∆ε) 202 (−6.31), 300 (−0.77), 310 (−0.73), 321 (−0.40) nm; IR (CHCl_3_) ν_max_ 3462, 2958, 2928, 1703, 1648, 1601, 1444, 1397, 1120, 1034 cm^−1^; ^1^H and ^13^C NMR data, see Table 1 and Table 2, Supplementary Figures S1–S8; HRESIMS [M + Na]^+^ *m*/*z* 273.1461 (calcd. for C_15_H_22_O_3_Na 273.1461), [M + H]^+^ *m*/*z* 251.1636 (calcd. for C_15_H_23_O_3_ 251.1642).

*Zosteropenilline B* (**2**): colorless crystals; ${\left[\alpha \right]}_{D}^{20}$ −161.6 (*c* 0.12, MeOH); UV (MeOH low intensity band; CD (*c* 0.07, MeOH), λmax (∆ε) 201 (−1.55), 291 (−0.23), 332 (+0.03), 365 (−0.03) nm; IR (CHCl_3_) ν_max_ 3460, 2957, 2919, 1701, 1602, 1455, 1385, 1108, 1055 cm^−1^; ^1^H and ^13^C NMR data, see Table 1 and Table 2, Supplementary Figures S11–S19; HRESIMS [M + Na]^+^ *m*/*z* 273.1474 (calcd. for C_15_H_22_O_3_Na 273.1461), [M − H]^−^ *m*/*z* 249.1496 (calcd. for C_15_H_21_O_3_ 249.1496).

*Zosteropenilline C* (**3**): white solid; ${\left[\alpha \right]}_{D}^{20}$ −170.0 (*c* 0.1, MeOH); UV (MeOH) low intensity band; CD (*c* 0.07, MeOH), λ_max_ (∆ε) 204 (−9.86), 297 (−1.63) nm; IR (CHCl_3_) ν_max_ 3604, 2973, 2927, 1705, 1602, 1453, 1375, 1140, 1044 cm^−1^; ^1^H and ^13^C NMR data, see Table 1 and Table 2, Supplementary Figures S20–S27; HRESIMS [M + Na]^+^ *m*/*z* 273.1463 (calcd. for C_15_H_22_O_3_Na 273.1461), [M − H]^−^ *m*/*z* 249.1499 (calcd. for C_15_H_21_O_3_ 249.1496).

*Zosteropenilline D* (**4**): amorphous; ${\left[\alpha \right]}_{D}^{20}$ −56.0 (*c* 0.06, MeOH); UV (MeOH) low intensity band; CD (*c* 0.06, MeOH), λ_max_ (∆ε) 204 (−4.01), 296 (−0.96) nm; IR (CHCl_3_) ν_max_ 2956, 2929, 1706, 1602, 1456, 1376, 1121, 1069 cm^−1^; ^1^H and ^13^C NMR data, see Table 1 and Table 2, Supplementary Figures S28–S35; HRESIMS [M + Na]^+^ *m*/*z* 257.1510 (calcd. for C_15_H_22_O_2_Na 257.1512).

*Zosteropenilline E* (**5**): amorphous; ${\left[\alpha \right]}_{D}^{20}$ −16.0 (*c* 0.05, MeOH); UV (MeOH) low intensity band; CD (*c* 0.05, MeOH), λ_max_ (∆ε) 206 (−0.27), 293 (−0.21), 299 (−0.22), 305 (−0.19), 339 (−0.03); ^1^H and ^13^C NMR data, see Table 1 and Table 2, Supplementary Figures S36–S42; HRESIMS [M − H]^−^ *m*/*z* 283.1538 (calcd. for C_15_H_23_O_5_ 283.1551).

*Zosteropenilline F* (**6**): white solid; ${\left[\alpha \right]}_{D}^{20}$ +9.1 (*c* 0.11, MeOH); UV (MeOH) low intensity band; CD (*c* 0.07, MeOH), λ_max_ (∆ε) 194 (+0.70), 204 (−1.14), 230 (−0.12), 293 (+0.08), 319 (−0.02) nm; IR (CHCl_3_) ν_max_ 3621, 2976, 2928, 1702, 1602, 1450, 1390, 1046 cm^−1^; ^1^H and ^13^C NMR data, see Table 1 and Table 2, Supplementary Figures S43–S50; HRESIMS [M + Na]^+^ *m*/*z* 291.1579 (calcd. for C_15_H_24_O_4_Na 291.1567), [2M + Na]^+^ *m*/*z* 559.3242 (calcd. for 2C_15_H_24_O_4_Na 559.3241).

*Zosteropenilline G* (**7**): white solid; ${\left[\alpha \right]}_{D}^{20}$ −15.3 (*c* 0.034, MeOH); UV (MeOH) λ_max_ (log ε) low intensity band; CD (*c* 0.05, MeOH), λ_max_ (∆ε) 199 (−1.85), 218 (−1.07), 244 (+0.11), 287 (+0.57) nm; IR (CHCl_3_) ν_max_ 3623, 3459, 2884, 1704, 1670, 1602, 1455, 1391, 1056 cm^−1^; ^1^H and ^13^C NMR data, see Table 3 and Table 4, Supplementary Figures S69–S77; HRESIMS [M + Na]^+^ *m*/*z* 275.1621 (calcd. for C_15_H_24_O_3_Na 275.1618), [M − H]^−^ *m*/*z* 251.1646 (calcd. for C_15_H_23_O_3_ 251.1653).

*Zosteropenilline H* (**8**): amorphous; ${\left[\alpha \right]}_{D}^{20}$ +83.4 (*c* 0.11, MeOH); UV (MeOH) low intensity band; CD (*c* 0.06, MeOH), λ_max_ (∆ε) 209 (+3.62), 295 (+1.98) nm; IR (CHCl_3_) ν_max_ 3617, 3489, 2959, 2929, 1703, 1602, 1456, 1383, 1055 cm^−1^; ^1^H and ^13^C NMR data, see Table 3 and Table 4, Supplementary Figures S78–S85; HRESIMS [M + Na]^+^ *m*/*z* 291.1571 (calcd. for C_15_H_24_O_4_Na 291.1567), [2M + Na]^+^ *m*/*z* 559.3236 (calcd. for 2C_15_H_24_O_4_Na 559.3241).

*Zosteropenilline I* (**9**): white solid; ${\left[\alpha \right]}_{D}^{20}$ +80.0 (*c* 0.07, MeOH); UV (MeOH) low intensity band; CD (*c* 0.04, MeOH), λ_max_ (∆ε) 204 (−6.61), 247 (−1.13) nm; ^1^H and ^13^C NMR data, see Table 3 and Table 4, Supplementary Figures S86–S93; HRESIMS [M + Na]^+^ *m*/*z* 273.1462 (calcd. for C_15_H_22_O_3_Na 273.1461), [2M + Na]^+^ *m*/*z* 523.3029 (calcd. for 2C_15_H_22_O_3_Na 523.3030).

*Zosteropenilline J* (**10**): colorless crystals; ${\left[\alpha \right]}_{D}^{20}$ −52.9 (*c* 0.71, MeOH); UV (MeOH) low intensity band; CD (*c* 0.06, MeOH), λ_max_ (∆ε) 196 (−3.81), 213 (+0.35), 238 (+0.05), 295 (−2.15) nm; IR (CHCl_3_) ν_max_ 3605, 3497, 2959, 2929, 1702, 1602, 1456, 1383, 1061 cm^−1^; ^1^H and ^13^C NMR data, see Table 3 and Table 4, Supplementary Figures S94–S101; HRESIMS [M + Na]^+^ *m*/*z* 291.1573 (calcd. for C_15_H_24_O_4_Na 291.1567), [2M + Na]^+^ *m*/*z* 559.3236 (calcd. for 2C_15_H_24_O_4_Na 559.3241).

*Zosteropenilline K* (**11**): amorphous; ${\left[\alpha \right]}_{D}^{20}$ +11.1 (*c* 0.09, MeOH); UV (MeOH) λ_max_ (log ε) 242 (3.46) nm; CD (*c* 0.06, MeOH), λ_max_ (∆ε) 193 (0.75), 219 (−4.62), 244 (+0.65), 258 (−0.13), 279 (0.46), 294 (0.41), 318 (0.35) nm; IR (CHCl_3_) ν_max_ 3617, 3464, 2958, 2927, 1702, 1671, 1653, 1604, 1456, 1390, 1059 cm^−1^; ^1^H and ^13^C NMR data, see Table 3 and Table 4, Supplementary Figures S102–S109; HRESIMS [M + Na]^+^ *m*/*z* 305.1370 (calcd. for C_15_H_22_O_5_Na 305.1359).

*Zosteropenilline L* (**12**): amorphous; ${\left[\alpha \right]}_{D}^{20}$ −54 (*c* 0.05, MeOH); UV (MeOH) low intensity band; CD (*c* 0.05, MeOH), λ_max_ (∆ε) 196 (−1.29), 210 (+0.14), 298 (−1.12), 349 (+0.04) nm; ^1^H and ^13^C NMR data, see Table 3 and Table 4, Supplementary Figures S110–S117; HRESIMS [M + Na]^+^ *m*/*z* 307.1501 (calcd. for C_15_H_24_O_5_Na 307.1516).

*Pallidopenilline A* (**13**): colorless crystals; ${\left[\alpha \right]}_{D}^{20}$ −21. (*c* 0.1, MeOH); UV (MeOH) λ_max_ (log ε) low intensity band; CD (*c* 0.1, MeOH), λ_max_ (∆ε) 196 (−9.89), 213 (+1.14), 294 (−1.78) nm; ^1^H and ^13^C NMR data, Supplementary Figures S51–S65; HRESIMS [M + Na]^+^ *m*/*z* 291.1575 (calcd. for C_15_H_24_O_5_Na 291.1567).

### 3.6. Preparation of (S)-MTPA and (R)-MTPA Esters of ***1***

To a solution of **1** (1.75 mg) in pyridine were added 4-dimethylaminopyridine (a few crystals) and (*R*)-MTPA-Cl (1.2 μL) at room temperature, and the mixture was stirred for 1.5 h. After evaporation of the solvent, the residue was purified by HPLC on an YMC Si (5 μm, 10 × 250 mm) column eluted with *n*-hexane–acetone (85:15) to afford the (*S*)-MTPA ester of **1** (**1a**). The (*R*)-MTPA ester of **1** (**1b**) was prepared similarly manner using (*S*)-MTPA-Cl. NMR data of (*R*, *S*)-MTPA esters of **1** (Figure S10). HRESIMS (**1a**) *m*/*z* 489.1864 [M + Na]^+^ (calcd. for C_25_H_29_F_3_O_5_Na, 489.1859), *m*/*z* 955.3830 [2M + Na]^+^ (calcd for C_50_H_58_F_6_O_10_Na, 955.3826); HRESIMS (**1b**) *m*/*z* 489.1858 [M + Na]^+^ (calcd. for C_25_H_29_F_3_O_5_Na, 489.1859), *m*/*z* 955.3815 [2M + Na]^+^ (calcd for C_50_H_58_F_6_O_10_Na, 955.3826).

### 3.7. X-ray Crystal Data for ***1***

Experimental intensity data for C_15_H_22_O_3_ were collected at T = 296(2) K (α-C_15_H_22_O_3_) on a BRUKER Kappa APEX2 diffractometer with graphite monochromated Mo K_α_ radiation (λ = 0.71073 Å). Intensity data were corrected for absorption using the multi-scan method. The structures were solved using direct methods and refined by least-squares calculation in anisotropic approximation for non-hydrogen atoms. Hydrogen atoms were placed at idealized positions and refined using a riding model. Data collection, reduction, and refinement of the lattice parameters were performed using the Apex II software package [12]. All calculations were performed with SHELXL/PC program [13]. Selected bond lengths and hydrogen bonds are listed in the Supplementary Materials, Figure S8. Supplementary crystallographic data (accession numbers CCDC 1522593) can be obtained free of charge from the Cambridge Crystallographic Data Center via http://www.ccdc.cam.ac. uk/data_request/cif (or from the Cambridge Crystallographic Data Centre, 12 Union Road, Cambridge, UK; fax: + 44 1223 336 033 or email: deposit@ccdc.cam.uk).

### 3.8. Quantum Chemical Modeling

The conformational analyses of **1** in a hexane solvent and for **2**–**4** in a CH_3_OH solvent were done using density functional theory (DFT) with the exchange-correlation functional PBE1PBE [14], the polarization continuum model (PCM) [15] and 6-311+G(d) split-valence basis set, implemented in the Gaussian 03 package of programs [16]. The molecular cavity was modeled according to unified force field (*radii = UFF*). The statistical weights (*g_im_*) of different conformations of **1**–**4** were obtained according to Equation (1):

(1) $$g_{im}=\frac{e^{-\Delta G_{im}/RT}}{\sum_{i}e^{-\Delta G_{im}/RT}}$$

where the summation was done over all possible conformations of **1**–**4** and ∆*G_im_* = *G_i_* − *G_m_*; *G* = *E*_el_ + *G*_tr,T_ + *G*_rot,T_ + *G*_vib,T_ is a sum of electronic, translational, rotational, and vibrational contributions to the Gibbs free energy, calculated at temperature T = 298.15 K; the subscript “m” denotes conformation, for which *G* is minimal. The excitation energies and the rotatory strengths were calculated using time-dependent density functional theory (TDDFT). Theoretical ECD spectra (∆*ε_calc_*(λ)) were simulated as a superposition of Gauss-type functions, individual for each transition from electronic ground state to the *i*-th calculated excited electronic state (1 ≤ *i* ≤ 50). The same value ξ = 0.20 eV for the bandwidths at 1/*e* peak heights was used. The total theoretical ECD spectra were obtained after statistical averaging:

(2) $$\varepsilon_{calc}\left(\lambda \right)=\underset{i}{\sum }g_{i}\cdot \Delta \varepsilon_{i,calc}\left(\lambda \right)$$

The scaled theoretical and experimental ECD spectra were obtained according to Equation (3):

(3) $$\varepsilon_{scaled}\left(\lambda \right)=\frac{\Delta \varepsilon \left(\lambda \right)}{\left|\Delta \varepsilon \left(\lambda_{peak}\right)\right|}$$

where the denominator |∆*ε*(*λ_peak_*)| is a modulo of the peak value for the chosen characteristic band in the corresponding ECD spectrum.

### 3.9. Measurement of Nitric Oxide Content

RAW 264.7 murine macrophages were plated into 96-well microplates and incubated at 37 °C with 5% CO_2_ for 2 h. After adhesion, cells were incubated with tested compounds (10.0 µM) for 24 h. For endogenously generated NO detection studies, the cells were coincubated with 10 µM of FA–OMe fluorescent probe for 8 h [17]. Prior to fluorescence registration, the cells were washed three times with PBS and then bathed in 200 µL/well of PBS. Green fluorescence of cells was registered at λ_ex_ = 460 nm and λ_em_ = 524 nm. In each experiment LPS from *E. coli* serotype 055:B5 (1.0 µg/mL, Sigma-Aldrich, St. Louis, MO, USA) were used as a positive control. Fluorescent intensity was measured using plate reader PHERAstar FS (BMG Labtech GmbH, Ortenberg, Germany).

### 3.10. Statistical Analysis

All assays were performed at least in triplicate. The results are expressed as the mean ± standard error (SD). A Student’s *t*-test was used to evaluate the data with the significance level of *p* < 0.05. The means and standard errors for each treatment were calculated and plotted using SigmaPlot 3.02 software (Jandel Scientific, San Rafael, CA, USA).

### 3.11. Cell Lines and Culture Conditions

The human prostate cancer cells line PC-3 was purchased from ATCC. Cells were cultured according to the manufacturer’s instructions in 10% FBS/RPMI media (Invitrogen, Paisley, UK) containing penicillin/streptomycin (Invitrogen). Cells were continuously kept in culture for a maximum of three months, and were routinely inspected microscopically for stable phenotype and regularly checked for contamination with mycoplasma. Cell line authentication was recently performed by DSMZ (Braunschweig, Germany) using highly polymorphic short tandem repeat loci [18].

### 3.12. In Vitro MTT-Based Drug Sensitivity Assay

The in vitro cytotoxicity of individual substances was evaluated using the MTT (3-(4,5-dimethylthiazol-2-yl)-2,5-diphenyltetrazolium bromide) assay, which was performed as previously described [19].

### 3.13. Western Blotting

Western blotting was performed as described before [19]. For detection of the proteins following primary and secondary antibody were used: anti-SQSTM/p62 (#5114, 1:1000, Cell Signaling Technology, Danvers, MA, USA), anti-α-tubulin (T5168, 1:10,000, Sigma-Aldrich, St. Louis, MO, USA), anti-mouse IgG-HRP (NXA931, 1:10,000, GE Healthcare, Little Chalfont, UK), anti-rabbit IgG-HRP (Cell Signaling Technology, Danvers, MA, USA, #7074, 1:5000). Signals were detected using the ECL chemiluminescence system (Thermo Scientific, Rockford, IL, USA) according to the manufacturer’s protocol. Relative optical density of the signal intensity of the bands was quantified with Quantity One 4.6 software (Bio-Rad, Hercules, CA, USA) and normalized first, against corresponding signal of α-tubulin (loading control), and second, against the signal of control sample. Mean values of two independent experiments ± SEM are presented. SEM values were calculated using GraphPad Prism software v. 5.01 (GraphPad Prism software Inc., La Jolla, CA, USA).

## 4. Conclusions

Zosteropenillines A–L (**1**–**12**) belong to the series of lovastatin-related polyketides that are formed by an enzymatic intramolecular Diels–Alder cycloaddition of a linear hexaketide precursor [20,21,22]. Polyketides containing “decalin” moiety exhibit diverse biological properties such as anticancer, antimicrobial, neurotrophic, and growth-stimulating activity [23,24,25,26,27]. Twelve new polyketides, named zosteropenillines A–L (**1**–**12**), have been isolated from the extract of the marine-derived fungus *Penicillium thomii*. The absolute configuration zosteropenilline A (**1**) was determined by a combination of the modified Mosher’s method, X-ray analysis, and NOESY data. Absolute configurations of zosteropenillines B–D (**2**–**4**) were determined by time-dependent density functional theory (TD-DFT) calculations of ECD spectra. Zosteropenillines B, G, and I at a non-toxic concentration of 10.0 µM induced a moderate downregulation of NO production in macrophages stimulated with LPS.

## Figures and Tables

**Table 1:** ^13^C NMR spectroscopic data (δ in ppm) for zosteropenillines A–F (**1**–**6**).

Position	1 *	2 *	3 *	4 **	5 **	6 *
1	60.9, CH_2_	60.9, CH_2_	60.9, CH_2_	60.9, CH_2_	60.9, CH_2_	57.6, CH_2_
2	39.9, CH_2_	39.9, CH_2_	39.9, CH_2_	39.9, CH_2_	37.9, CH_2_	48.6, CH_2_
3	210.2, C	210.4, C	210.3, C	210.9, C	206.0, C	217.6, C
4	62.8, CH	62.9, CH	62.7, CH	63.0, CH	63.7, CH	61.8, CH
5	37.5, CH	39.5, CH	38.9, CH	39.3. CH	38.5, CH	37.4, CH
6	28.7, CH_2_	28.8, CH_2_	38.7, CH_2_	29.3, CH_2_	29.0, CH_2_	29.4, CH_2_
7	32.7, CH_2_	29.1, CH_2_	24.9, CH_2_	34.9, CH_2_	31.8, CH_2_	32.9, CH_2_
8	40.2, CH	40.5, CH	69.7, C	32.8, CH	39.2, CH	40.6, CH
9	77.9, CH	35.2, CH_2_	44.5, CH_2_	40.9, CH_2_	79.2, CH	77.8, CH
10	48.6, CH	41.4, CH	36.9, CH	41.9, CH	53.5, CH	49.1, CH
11	130.1, CH	134.4, CH	134.4, CH	130.5, CH	67.3, CH	128.6, CH
12	131.6, CH	130.9, CH	131.1, CH	134.8, CH	72.7, CH	133.0, CH
13	74.7, C	75.0, C	75.0, C	75.2, C	77.5, C	69.1, C
14	23.2, CH_3_	23.3, CH_3_	23.3, CH_3_	23.3, CH_3_	22.0, CH_3_	28.8, CH_3_
15	18.4, CH_3_	68.1, CH_2_	31.5, CH_3_	22.3, CH_3_	19.1, CH_3_	18.4, CH_3_

* Chemical shifts were measured at 125.77 MHz in CDCl_3_; ** Chemical shifts were measured at 176.04 MHz in CDCl_3_.

**Table 2:** ^1^H NMR spectroscopic data (δ, *J* in Hz) for zosteropenillines A–F (**1**–**6**).

Position	1 **	2 *	3 *	4 **	5 **	6 *
1	a: 4.11, ddd (12.0, 8.0, 1.2) b: 3.89, td (12.0, 3.0)	a: 4.11, ddd (11.8, 8.0, 1.0) b: 3.89 td (12.0, 3.0)	a: 4.11, ddd (11.8, 8.0, 1.0) b: 3.89, td (12.0, 2.9)	a: 4.11, ddd (11.7, 8.0, 1.4) b: 3.89, td (12.0, 3.0)	a: 4.22, dd (12.0, 9.0) b: 3.91, td (12.0, 4.0)	a: 3.91, ddd (11.7, 6.2, 4.2) b: 3.87, ddd (11.7, 6.2, 4.2)
2	a: 2.64, ddd (14.0, 12.0, 8.0) b: 2.21, ddt (14.0, 3.0, 1.2)	a: 2.66, ddd (14.1, 12.1, 8.0) b: 2.21, ddt (14.1, 3.0, 1.3)	a: 2.66, ddd (14.0, 12.1, 8.0) b: 2.21, ddt (13.9, 2.9, 1.3)	a: 2.68, ddd (14.0, 12.0, 3.0) b: 2.21, ddt (14.0, 3.0, 1.5)	a: 2.67, ddd (15.0, 12.0, 9.0) b: 2.21, dd (15.0, 4.0)	a: 2.86, ddd (18.4, 6.2, 4.2) b: 2.76, ddd (18.4, 6.2, 4.4)
4	2.09, dd (11.8, 1.2)	2.07, dd (11.3, 1.2)	2.10, dd (11.3, 1.2)	2.04, dd (11.5, 1.6)	2.11, d (11.7)	2.59, d (11.4)
5	1.89, tdd (11.8, 10.6, 3.2)	1.78, m	1.75, m	1.75, m	1.95, m	1.93, tdd (11.4, 10.4, 3.0)
6	a: 1.28, m b: 1.14, qd (13.0, 3.3)	a: 1.41, dq (13.2, 3.2) b: 1.14, qd (13.2, 3.5)	a: 1.69, m b: 1.41, m	a: 1.34, dq (13.3, 3.6) b: 1.11, qd (13.0, 3.8)	a: 1.33, m b: 1.11, m	a: 1.50, m b: 1.15, qd (12.7, 3.2)
7	a: 1.78, dq (12.7, 3.3) b: 1.08, qd (12.7, 3.6)	a: 1.85, dq (12.9, 3.4) b: 1.01, qd (12.9, 3.7)	a: 1.45, m b: 1.19, m	a: 1.75, m b: 0.96, m	a: 1.73, m b: 1.03, m	a: 1.77, m b: 1.12, qd (13.0, 3.3)
8	1.41, m	1.63, m		1.48, m	1.48, m	1.42, m
9	2.92, td (9.9, 5.8)	a: 1.91, dq (12.6, 3.5) b: 0.93, q (12.3)	a: 1.75, m b: 1.28, m	a: 1.77, m b: 0.87, q (12.2)	3.29, t (9.0)	2.92, t (9.9)
10	1.65, tt (10.4, 2.3)	1.74, m	2.15, ddd (13.3, 10.2, 3.5)	1.71, m	1.61, td (10.8, 9.0)	1.73, tt (10.3, 2.3)
11	6.24, dd (10.0, 2.4)	5.67, dd (9.8, 1.5)	5.63, brs	5.59, dd (9.9, 2.5)	4.38, t (10.5)	6.11, dd (10.0, 2.0)
12	5.69, dd (10.0, 2.6)	5.61, dd (9.8, 2.5)	5.63, brs	5.65, dd (9.8, 1.0)	3.78, d (10.5)	5.64, dd (10.0, 2.6)
14	1.26, s	1.26, s	1.26, s	1.25, s	1.36, s	1.30, s
15	1.03, d (6.5)	a: 3.50, dd (10.3, 6.3) b: 3.47, dd (10.3, 6.3)	1.25, s	0.91, d (6.6)	1.06, d (6.5)	1.03, d (6.5)
9-OH	1.55, d (6.3)					

* Chemical shifts were measured at 500.13 MHz in CDCl_3_; ** Chemical shifts were measured at 700.13 MHz in CDCl_3_.

**Table 3:** ^13^C NMR spectroscopic data (δ in ppm) for zosteropenillines G–L (**7**–**12**).

Position	7 *	8 **	9 *	10 **	11 *	12 **
1	58.1, CH_2_	57.9, CH_2_	58.0, CH_2_	58.0, CH_2_	57.9, CH_2_	58.0, CH_2_
2	49.3, CH_2_	43.6, CH_2_	42.1, CH_2_	49.2, CH_2_	48.9, CH_2_	49.5, CH_2_
3	214.3, C	214.0, C	213.5, C	214.1, C	211.2, C	213.2, C
4	63.5, CH	61.7, CH	59.6, CH	63.4, CH	61.6, CH	57.7, CH
5	41.3, CH	37.9, CH	40.3, CH	41.5, CH	39.4, CH	40.3, CH
6	29.4, CH_2_	30.5, CH_2_	29.9, CH_2_	28.7, CH_2_	32.4, CH_2_	32.1, CH_2_
7	34.8, CH_2_	31.9, CH_2_	32.6, CH_2_	28.9, CH_2_	27.9, CH_2_	28.1, CH_2_
8	32.9, CH	39.2, CH	40.4, CH	40.6, CH	41.1, CH	41.1, CH
9	40.9, CH_2_	82.3, CH	78.3, CH	35.2, CH_2_	38.3, CH_2_	37.2, CH_2_
10	42.1, CH	49.6, CH	47.2, CH	41.5, CH	166.3, C	144.5, C
11	131.9, CH	73.2, CH	130.6, CH	131.5, CH	120.0, CH	118.8, CH
12	133.7, CH	129.0, CH	129.3, CH	134.0, CH	200.3, C	68.0, CH
13	73.0, C	131.5, C	140.8, C	72.9, C	74.2, C	72.4, C
14	25.9, CH_3_	20.9, CH_3_	112.8, CH_2_	25.9, CH_3_	21.8, CH_3_	20.5, CH_3_
15	22.3, CH_3_	17.8, CH_3_	18.4, CH_3_	68.1, CH_2_	67.4, CH_2_	67.8, CH_2_

* Chemical shifts were measured at 125.77 MHz in CDCl_3_; ** Chemical shifts were measured at 176.04 MHz in CDCl_3_.

**Table 4:** ^1^H NMR spectroscopic data (δ, *J* in Hz) for zosteropenillines G–L (**7**–**12**).

Position	7 *	8 **	9 *	10 **	11 *	12 ***
1	a: 3.90, ddd (11.7, 7.1, 3.7) b: 3.86, ddd (11.7, 6.6, 3.7)	3.85, m (2H)	a: 3.91, ddd (11.5, 7.1, 3.9) b: 3.85, ddd (11.5, 6.8, 4.2)	a: 3.91, ddd (11.2, 6.9, 3.6) b: 3.87, ddd (11.2, 6.7, 3.7)	a: 3.96, ddd (11.5, 7.7, 3.7) b: 3.82, ddd (11.5, 6.7, 3.9)	a: 3.95, ddd (11.3, 7.5, 3.5) b: 3.82, ddd (11.3, 6.4, 3.6)
2	a: 3.04, ddd (18.0, 6.8, 3.8) b: 2.60, ddd (18.0, 7.1, 3.8)	a: 2.69, ddd (18.2, 6.6, 4.3) b: 2.64, ddd (18.2, 6.3, 4.3)	a: 2.83, ddd (18.3, 6.8, 3.8) b: 2.65, ddd (18.3, 6.8, 4.0)	a: 3.06, ddd (18.0, 7.1, 3.7) b: 2.61, ddd (18.0, 7.0, 3.7)	a: 3.14, ddd (18.0, 6.5, 3.6) b: 2.66, ddd (18.0, 7.6, 3.9)	a: 3.05, ddd (18.1, 6.5, 3.6) b: 2.69, ddd (18.0, 7.6, 3.7)
4	2.89, d (11.5)	2.92, m	3.15, dtd (12.1, 2.6, 0.9)	2.92, d (11.6)	3.01, d (9.8)	3.09, d (10.0)
5	1.48, m	1.69, m	1.68, m	1.52, dq (11.7, 2.7)	2.89, m	2.55, m
6	a: 1.70, m b: 0.93, m	a: 1.57, dq (13.2, 3.5) b: 1.17, qd (13.1, 3.5)	a: 1.53, m b: 1.14, m	a: 1.79, m b: 0.95, qd (12.3, 3.6)	a: 1.91, dq (12.9, 3.0) b: 1.15, qd (13.1, 3.3)	a: 1.74, m b: 1.12, m
7	a: 1.71, m b: 0.96, m	a: 1.70, m b: 1.03, qd (13.2, 3.5)	a: 1.77, m b: 1.10, m	a: 1.79, m b: 1.02, qd (12.4, 3.6)	a: 1.84, dq (13.1, 3.2) b: 1.29, m	a: 1.79, m b: 1.12, m
8	1.46, m	1.47, m	1.43, m	1.61, m	1.74, m	1.64, m
9	a: 1.73, m b: 0.77, q (12.4)	3.25, t (9.3)	2.89, t (9.9)	a: 1.87, m b: 0.82, q (13.0)	a: 2.60, dq (14.0, 2.5) b: 2.01, m	a: 2.39, dq (13.5, 2.5) b: 1.77, m
10	1.84, m	1.28, dt (11.9, 9.3)	1.85, t (10.0)	1.87, m		
11	5.42, brs	4.32, dq (8.9, 2.1)	6.20, brs	5.44, brs	5.90, brs	5.60, dt (5.9, 2.1)
12	5.42, brs	5.53, q (1.8)	6.20, brs	5.44, brs		4.36, dd (5.9, 1.7)
14	1.20, s	1.60, brs	a: 4.99, dd (2.8, 1.3) b: 4.59, dt (2.3, 0.9)	1.20, s	1.19, s	1.20, s
15	0.89, d (6.5)	1.02, d (6.5)	1.05, d (6.5)	a: 3.47, dd (10.6, 6.6) b: 3.45, dd (10.6, 6.6)	a: 3.58, dd (10.5, 5.8) b: 3.54, dd (10.5, 5.8)	a: 3.50, dd (10.5, 5.9) b: 3.48, dd (10.4, 6.2)

* Chemical shifts were measured at 500.13 MHz in CDCl_3_; ** Chemical shifts were measured at 700.13 MHz in CDCl_3_.

## Supplementary Materials

^1^H, ^13^C, DEPT, COSY-45, HSQC, HMBC and NOESY spectra of all compounds are available online at www.mdpi.com/1660-3397/15/2/46/s1.
