# Supplementary material for: Zosteropenillines: Polyketides from the Marine-Derived Fungus Penicillium thomii
Source: Mar Drugs. 2017 Feb 17;15(2):46. doi: 10.3390/md15020046 (PMC5334626; doi:10.3390/md15020046)

Supplementary Meterials: Zosteropenillines: Polyketides from the Marine-derived Fungus *Penicillium thomii*

Shamil Sh. Afiyatullov, Elena V. Leshchenko, Dmitrii V. Berdyshev, Maria P. Sobolevskaya, Alexandr S. Antonov, Vladimir A. Denisenko, Roman S. Popov, Mikhail V. Pivkin, Anatoly A. Udovenko, Evgeny A. Pislyagin, Gunhild von Amsberg and Sergey A. Dyshlovoy

Contents

Figure S1. ^{1}H NMR spectrum (700.13 MHz) of **1** in $CDCl_3$

Figure S2. ^{13}C NMR spectrum (176.04 MHz) of **1** in $CDCl_3$

Figure S3. DEPT-135 spectrum (176.04 MHz) of **1** in $CDCl_3$

Figure S4. DEPT-90 spectrum (176.04) of **1** in $CDCl_3$

Figure S5. HSQC spectrum of **1** in $CDCl_3$

Figure S6. HMBC spectrum of **1** in $CDCl_3$

Figure S7. 1H-1H COSY spectrum of **1** in $CDCl_3$

Figure S8. NOESY spectrum of **1** in $CDCl_3$

Figure S9. X-Ray data for **1**

Figure S10. ^{1}H and ^{13}C NMR Spectroscopic data for S-MTPA ester of **1** (**1a**) and R-MTPA ester of **1** (**1b**)

Figure S11. ^{1}H NMR spectrum (500.13 MHz) of **2** in $CDCl_3$

Figure S12. ^{13}C NMR spectrum (125.77 MHz) of **2** in $CDCl_3$

Figure S13. DEPT-135 spectrum (125.77 MHz) of **2** in $CDCl_3$

Figure S14. DEPT-90 spectrum (125.77 MHz) of **2** in $CDCl_3$

Figure S15. HSQC spectrum of **2** in $CDCl_3$

Figure S16. HMBC spectrum of **2** in $CDCl_3$

Figure S17. 1H-1H COSY spectrum of **2** in $CDCl_3$

Figure S19. NOESY spectrum of **2** in $CDCl_3$

Figure S20. ^{1}H NMR spectrum (500.13 MHz) of **3** in $CDCl_3$

Figure S21. ^{13}C NMR spectrum (125.77 MHz) of **3** in $CDCl_3$

Figure S22. DEPT-135 spectrum (125.77 MHz) of **3** in $CDCl_3$

Figure S23. DEPT-90 spectrum (125.77 MHz) of **3** in $CDCl_3$

Figure S24. HSQC spectrum of **3** in $CDCl_3$

Figure S25. HMBC spectrum of **3** in $CDCl_3$

Figure S26. 1H-1H COSY spectrum of **3** in $CDCl_3$

Figure S27. NOESY spectrum of **3** in $CDCl_3$

Figure S28. ^{1}H NMR spectrum (700.13 MHz) of **4** in $CDCl_3$

Figure S29. ^{13}C NMR spectrum (176.04 MHz) of **4** in $CDCl_3$

Figure S30. DEPT-135 spectrum (176.04 MHz) of **4** in $CDCl_3$

Figure S31. DEPT-90 spectrum (176.04 MHz) of **4** in $CDCl_3$

Figure S32. HSQC spectrum of **4** in $CDCl_3$

Figure S33. HMBC spectrum of **4** in $CDCl_3$

Figure S34. 1H-1H COSY spectrum of **4** in $CDCl_3$

Figure S35. NOESY spectrum of **4** in $CDCl_3$

Figure S36. ^{1}H NMR spectrum (700.13 MHz) of **5** in $CDCl_3$

Figure S37. ^{13}C NMR spectrum (176.04 MHz) of **5** in $CDCl_3$

Figure S38. DEPT-135 spectrum (176.04 MHz) of **5** in $CDCl_3$

Figure S39. HSQC spectrum of **5** in $CDCl_3$

Figure S40. HMBC spectrum of **5** in $CDCl_3$

Figure S41. 1H-1H COSY spectrum of **5** in $CDCl_3$

Figure S42. NOESY spectrum of **5** in $CDCl_3$

Figure S43. ^{1}H NMR spectrum (500.13 MHz) of **6** in $CDCl_3$

Figure S44. ^{13}C NMR spectrum (125.77 MHz) of **6** in $CDCl_3$

Figure S45. DEPT-135 spectrum (125.77 MHz) of **6** in $CDCl_3$

Figure S46. DEPT-90 spectrum (125.77 MHz) of **6** in $CDCl_3$

Figure S47. HSQC spectrum of **6** in $CDCl_3$

Figure S48. HMBC spectrum of **6** in $CDCl_3$

Figure S49. 1H-1H COSY spectrum of **6** in $CDCl_3$

Figure S50. NOESY spectrum of **6** in $CDCl_3$

Figure S51. ^{1}H NMR spectrum (500.13 MHz) of **13** in DMSO-d6

Figure S52. ^{13}C NMR spectrum (125.77 MHz) of **13** in DMSO-d6

Figure S53. DEPT-135 spectrum (125.77 MHz) of **13** in DMSO-d6

Figure S54. HSQC spectrum of **13** in DMSO-d6

Figure S55. HMBC spectrum of **13** in DMSO-d6

Figure S56. 1H-1H COSY spectrum of **13** in DMSO-d6

Figure S57. NOESY spectrum of **13** in DMSO-d6

Figure S58. ^{1}H NMR spectrum (500.13 MHz) of **13** in $CDCl_3$

Figure S59. ^{13}C NMR spectrum (125.77 MHz) of **13** in $CDCl_3$

Figure S60. DEPT-135 spectrum (125.77 MHz) of **13** in $CDCl_3$

Figure S61. DEPT-90 spectrum (125.77 MHz) of **13** in $CDCl_3$

Figure S62. HSQC spectrum of **13** in $CDCl_3$

Figure S63. HMBC spectrum of **13** in $CDCl_3$

Figure S64. 1H-1H COSY spectrum of **13** in $CDCl_3$

Figure S65. NOESY spectrum of **13** in $CDCl_3$

Figure S66. ^{13}C NMR spectrum (125.77 MHz) of **13** and **6** in $CDCl_3$

Figure S67. DEPT-135 spectrum (125.77 MHz) of **13** and **6** in $CDCl_3$

Figure S68. ^{1}H NMR spectrum (125.77 MHz) of **13** and **6** in $CDCl_3$

Figure S69. ^{1}H NMR spectrum (500.13 MHz) of **7** in $CDCl_3$

Figure S70. ^{13}C NMR spectrum (125.77 MHz) of **7** in $CDCl_3$

Figure S71. DEPT-135 spectrum (125.77 MHz) of **7** in $CDCl_3$

Figure S72. DEPT-90 spectrum (125.77 MHz) of **7** in $CDCl_3$

Figure S73. HSQC spectrum of **7** in $CDCl_3$

Figure S74. HMBC spectrum of **7** in $CDCl_3$

Figure S75. 1H-1H COSY spectrum of **7** in $CDCl_3$

Figure S76. NOESY spectrum of **7** in $CDCl_3$

Figure S77. NOESY spectrum of **7** in DMSO-d6

Figure S78. ^{1}H NMR spectrum (700.13 MHz) of **8** in $CDCl_3$

Figure S79. ^{13}C NMR spectrum (176.04 MHz) of **8** in $CDCl_3$

Figure S80. DEPT-135 spectrum (176.04 MHz) of **8** in $CDCl_3$

Figure S81. DEPT-90 spectrum (176.04) of **8** in $CDCl_3$

Figure S82. HSQC spectrum of **8** in $CDCl_3$

Figure S83. HMBC spectrum of **8** in $CDCl_3$

Figure S84. 1H-1H COSY spectrum of **8** in $CDCl_3$

Figure S85. NOESY spectrum of **8** in $CDCl_3$

Figure S86. ^{1}H NMR spectrum (500.13 MHz) of **9** in $CDCl_3$

Figure S87. ^{13}C NMR spectrum (125.77 MHz) of **9** in $CDCl_3$

Figure S88. DEPT-135 spectrum (125.77 MHz) of **9** in $CDCl_3$

Figure S89. DEPT-90 spectrum (125.77 MHz) of **9** in $CDCl_3$

Figure S90. HSQC spectrum of **9** in $CDCl_3$

Figure S91. HMBC spectrum of **9** in $CDCl_3$

Figure S92. 1H-1H COSY spectrum of **9** in $CDCl_3$

Figure S93. NOESY spectrum of **9** in $CDCl_3$

Figure S94. ^{1}H NMR spectrum (700.13 MHz) of **10** in $CDCl_3$

Figure S95. ^{13}C NMR spectrum (176.04 MHz) of **10** in $CDCl_3$

Figure S96. DEPT-135 spectrum (176.04 MHz) of **10** in $CDCl_3$

Figure S97. DEPT-90 spectrum (176.04) of **10** in $CDCl_3$

Figure S98. HSQC spectrum of **10** in $CDCl_3$

Figure S99. HMBC spectrum of **10** in $CDCl_3$

Figure S100. 1H-1H COSY spectrum of **10** in $CDCl_3$

Figure S101. NOESY spectrum of **10** in $CDCl_3$

Figure S102. ^{1}H NMR spectrum (500.13 MHz) of **11** in $CDCl_3$

Figure S103. ^{13}C NMR spectrum (125.77 MHz) of **11** in $CDCl_3$

Figure S104. DEPT-135 spectrum (125.77 MHz) of **11** in $CDCl_3$

Figure S105. DEPT-90 spectrum (125.77 MHz) of **11** in $CDCl_3$

Figure S106. HSQC spectrum of **11** in $CDCl_3$

Figure S107. HMBC spectrum of **11** in $CDCl_3$

Figure S108. 1H-1H COSY spectrum of **11** in $CDCl_3$

Figure S109. NOESY spectrum of **11** in $CDCl_3$

Figure S110. ^{1}H NMR spectrum (500.13 MHz) of **12** in $CDCl_3$

Figure S111. ^{13}C NMR spectrum (125.77 MHz) of **12** in $CDCl_3$

Figure S112. DEPT-135 spectrum (125.77 MHz) of **12** in $CDCl_3$

Figure S113. DEPT-90 spectrum (125.77 MHz) of **12** in $CDCl_3$

Figure S114. HSQC spectrum of **12** in $CDCl_3$

Figure S115. HMBC spectrum of **12** in $CDCl_3$

Figure S116. 1H-1H COSY spectrum of **12** in $CDCl_3$

Figure S117. NOESY spectrum of **12** in $CDCl_3$

Figure S1. ^{1}H NMR spectrum (700.13 MHz) of **1** in $CDCl_3$

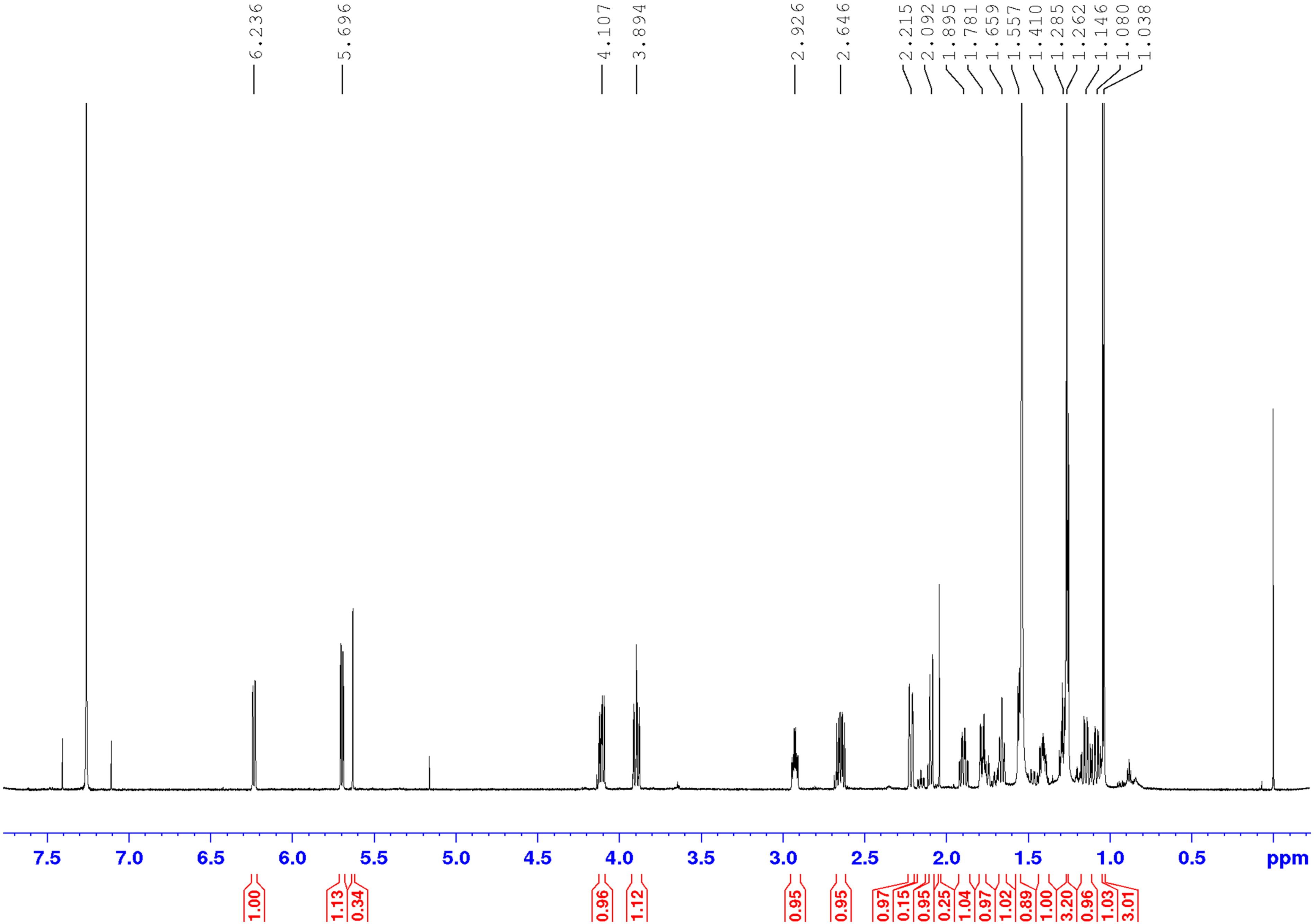

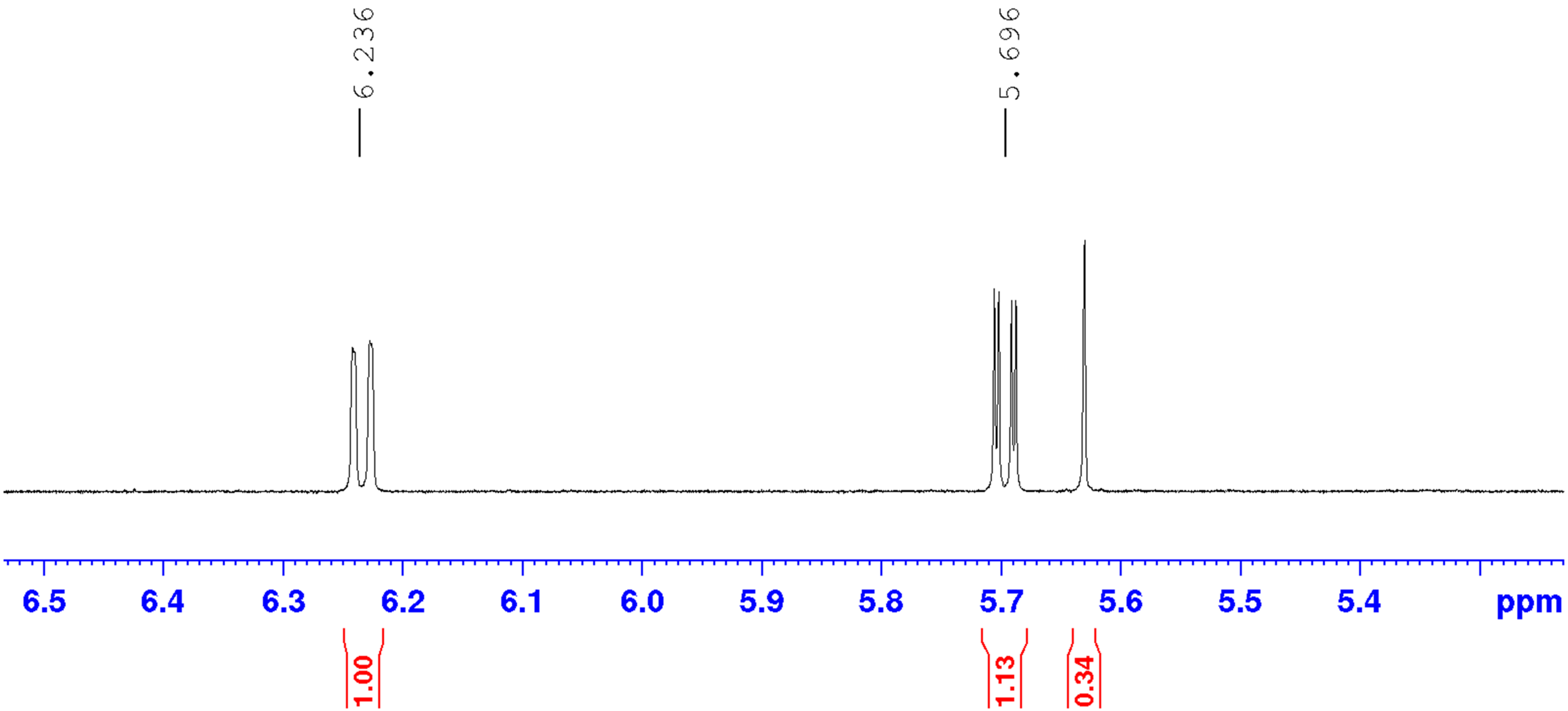

6.236
5.696
6.5
6.4
6.3
6.2
6.1
6.0
5.9
5.8
5.7
5.6
5.5
5.4
ppm
1.00
1.13
0.34

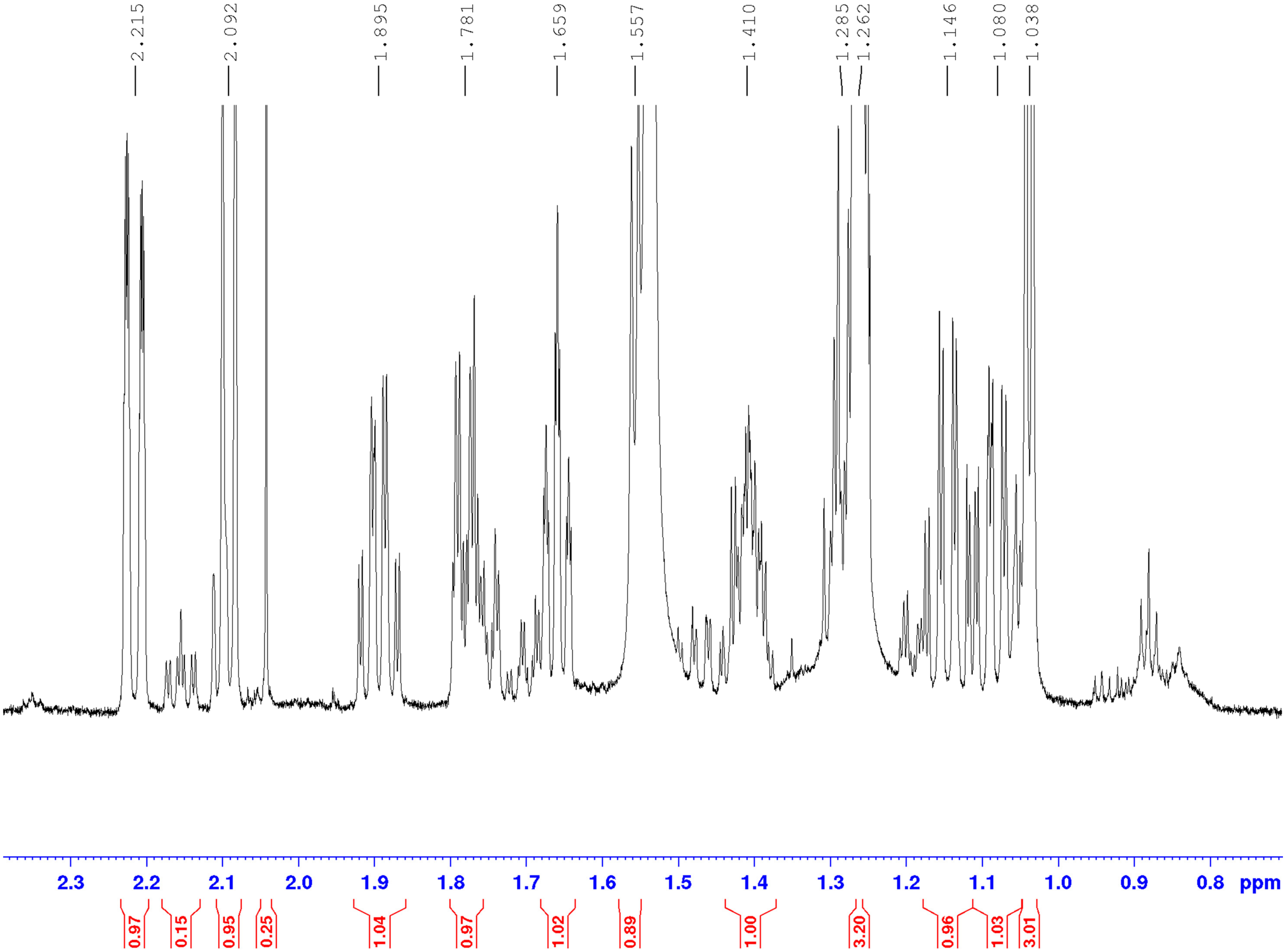

2.215
2.092
1.895
1.781
1.659
1.557
1.410
1.285
1.262
1.146
1.080
1.038
2.3
2.2
2.1
2.0
1.9
1.8
1.7
1.6
1.5
1.4
1.3
1.2
1.1
1.0
0.9
0.8
ppm
0.97
0.15
0.95
0.25
1.04
0.97
1.02
0.89
1.00
3.20
0.96
1.03
3.01

Figure S2. ^{13}C NMR spectrum (125.77 MHz) of **1** in $CDCl_3$

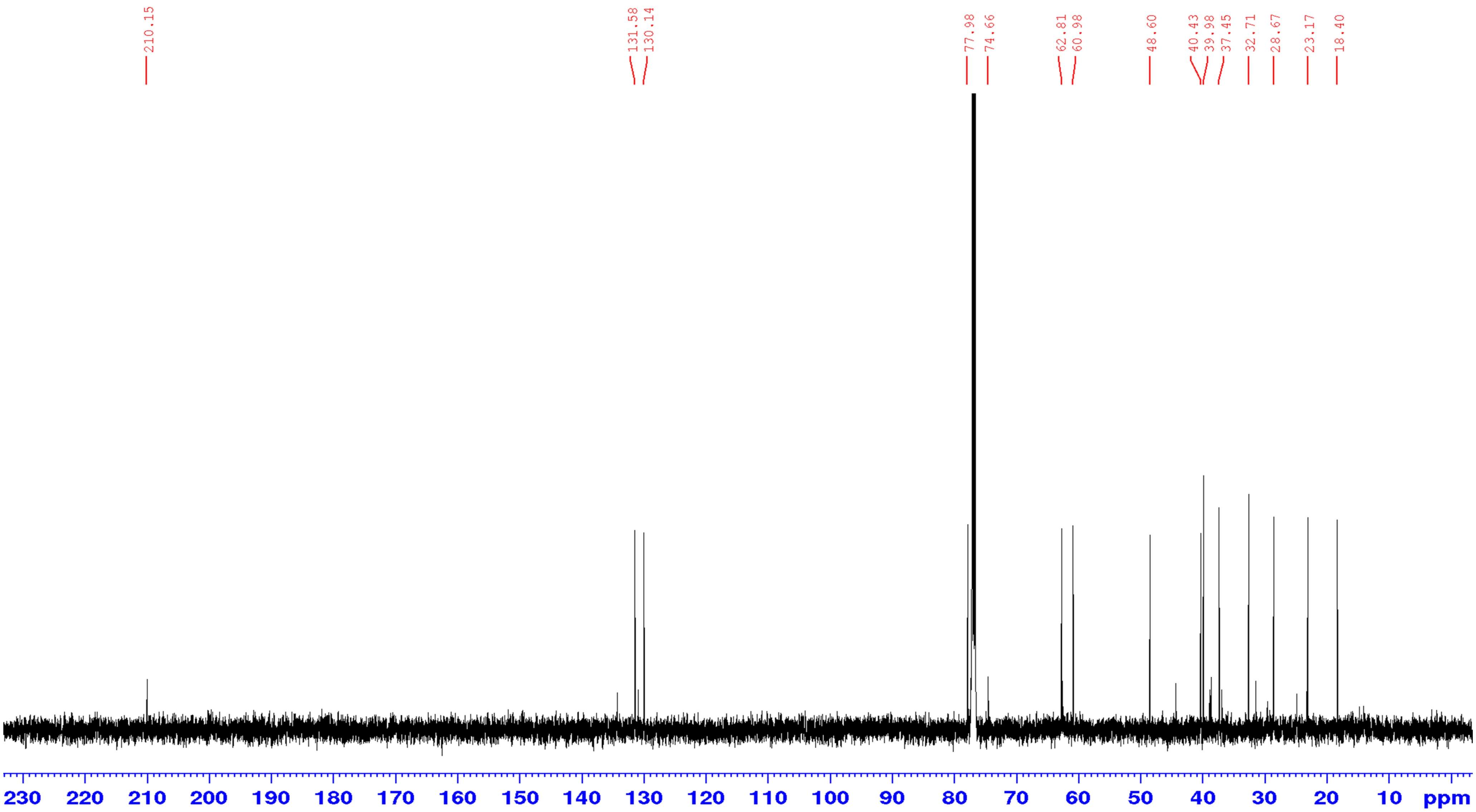

Figure S3. DEPT-135 spectrum (176.04 MHz) of **1** in $CDCl_3$

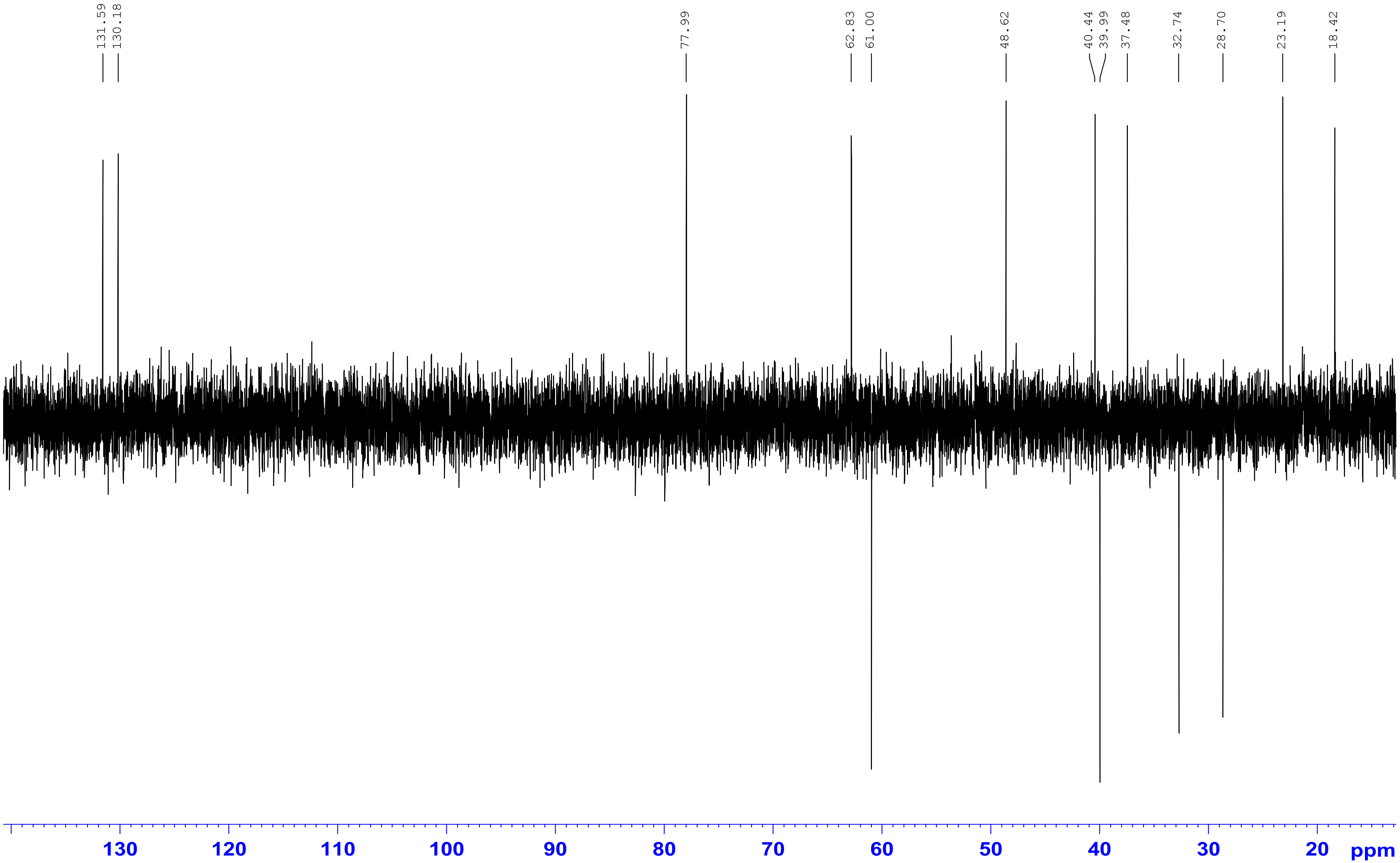

Figure S4. DEPT-90 spectrum (176.04) of **1** in $CDCl_3$

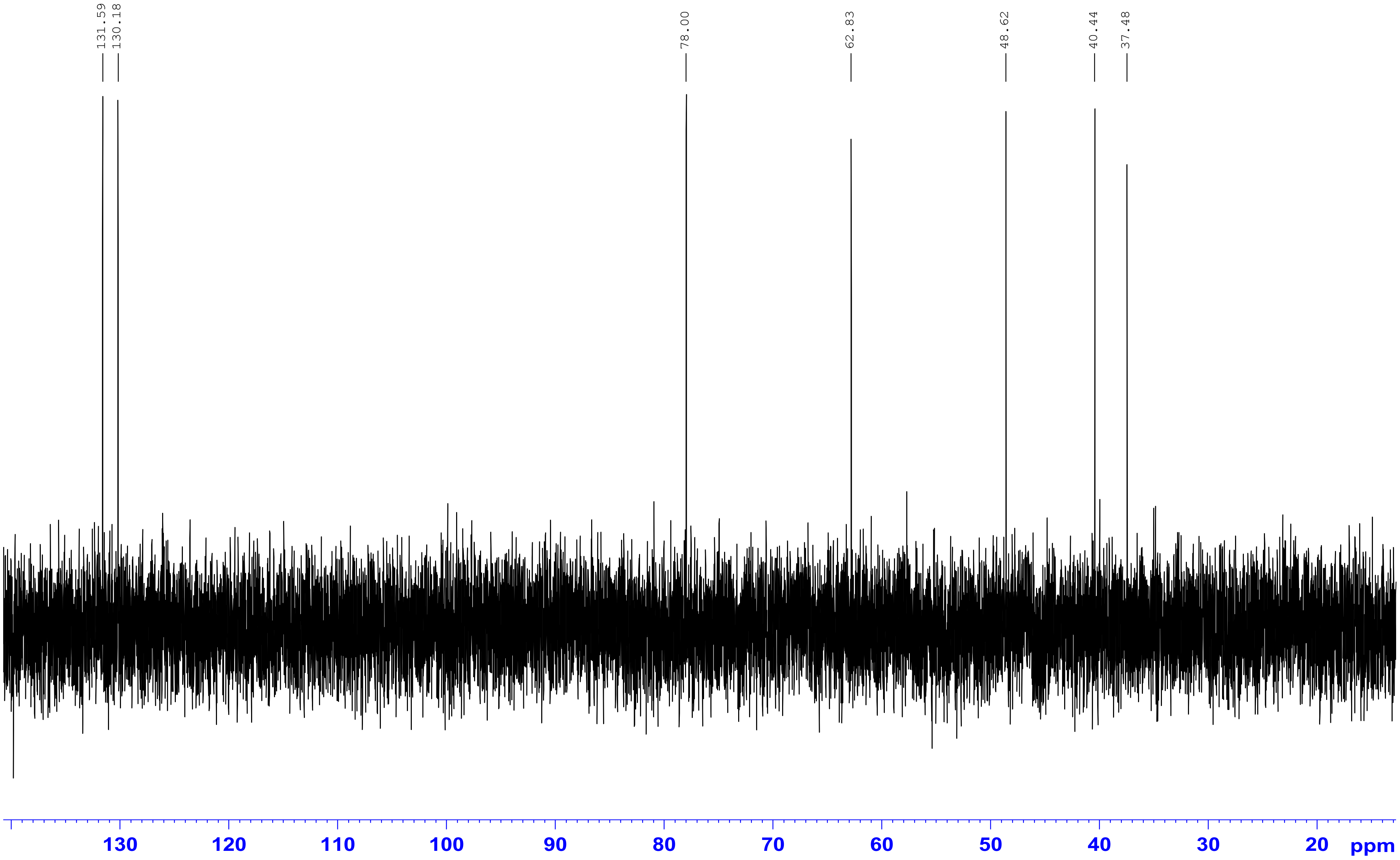

Figure S5. HSQC spectrum of **1** in $CDCl_3$

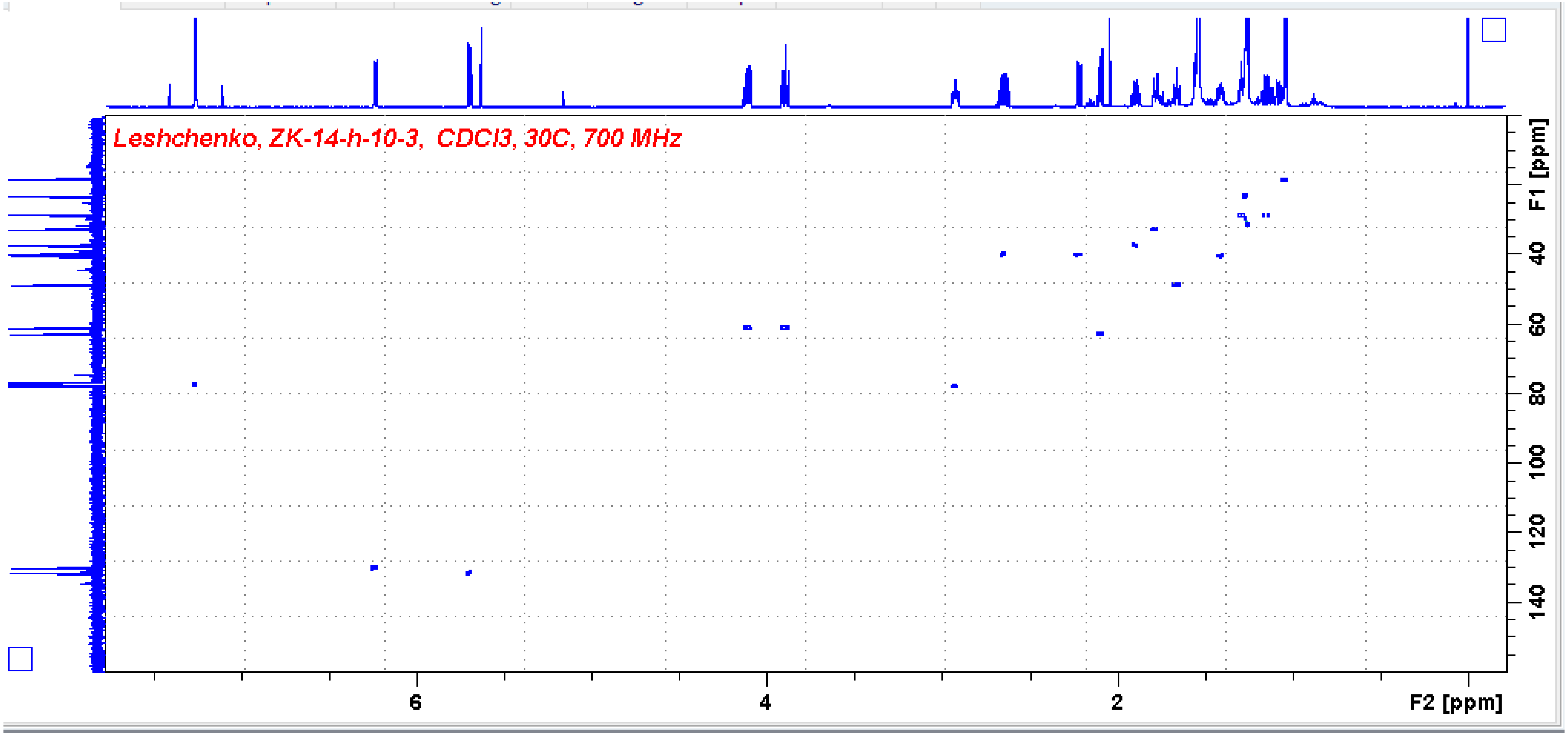

Figure S6. HMBC spectrum of **1** in $CDCl_3$

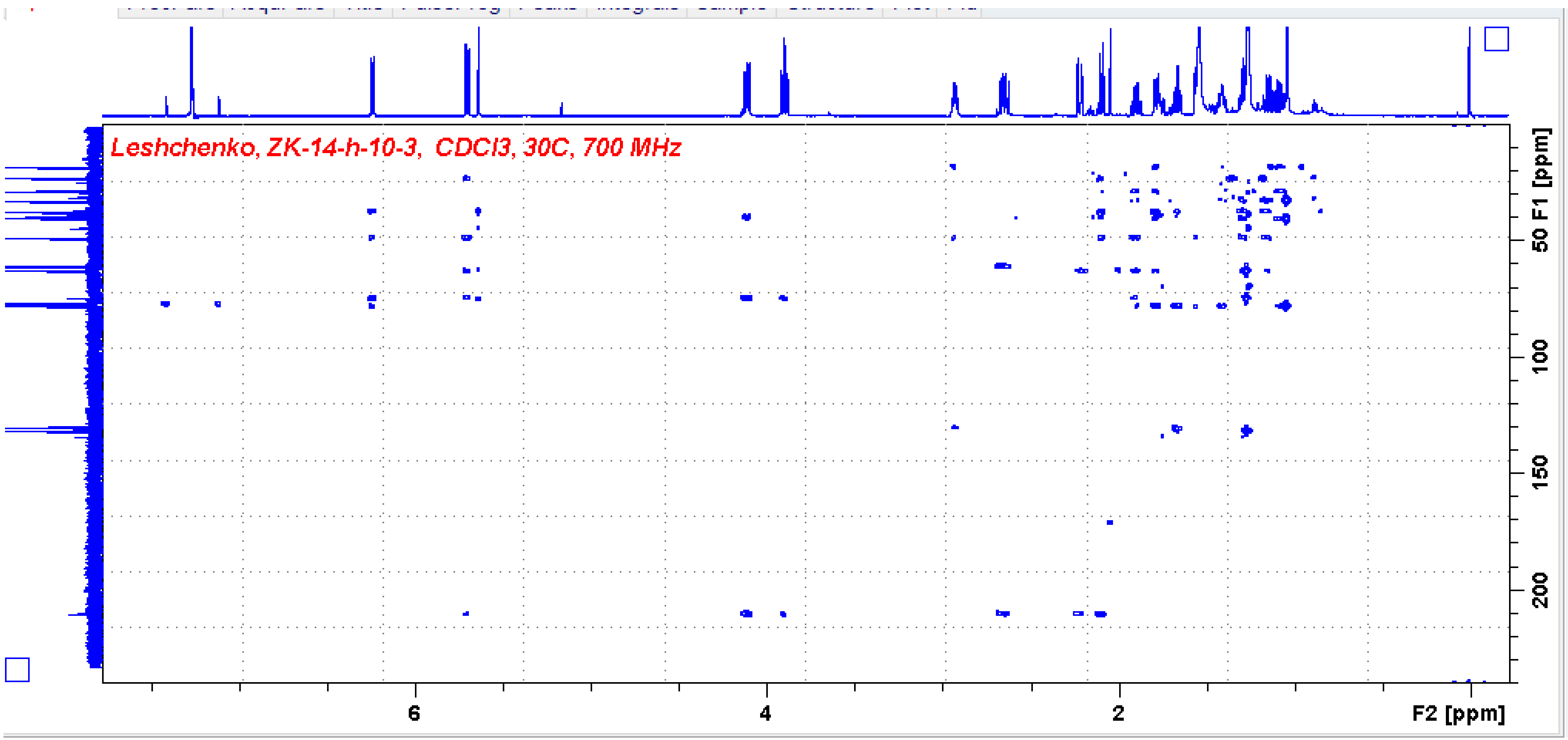

Figure S7. 1H-1H COSY spectrum of **1** in $CDCl_3$

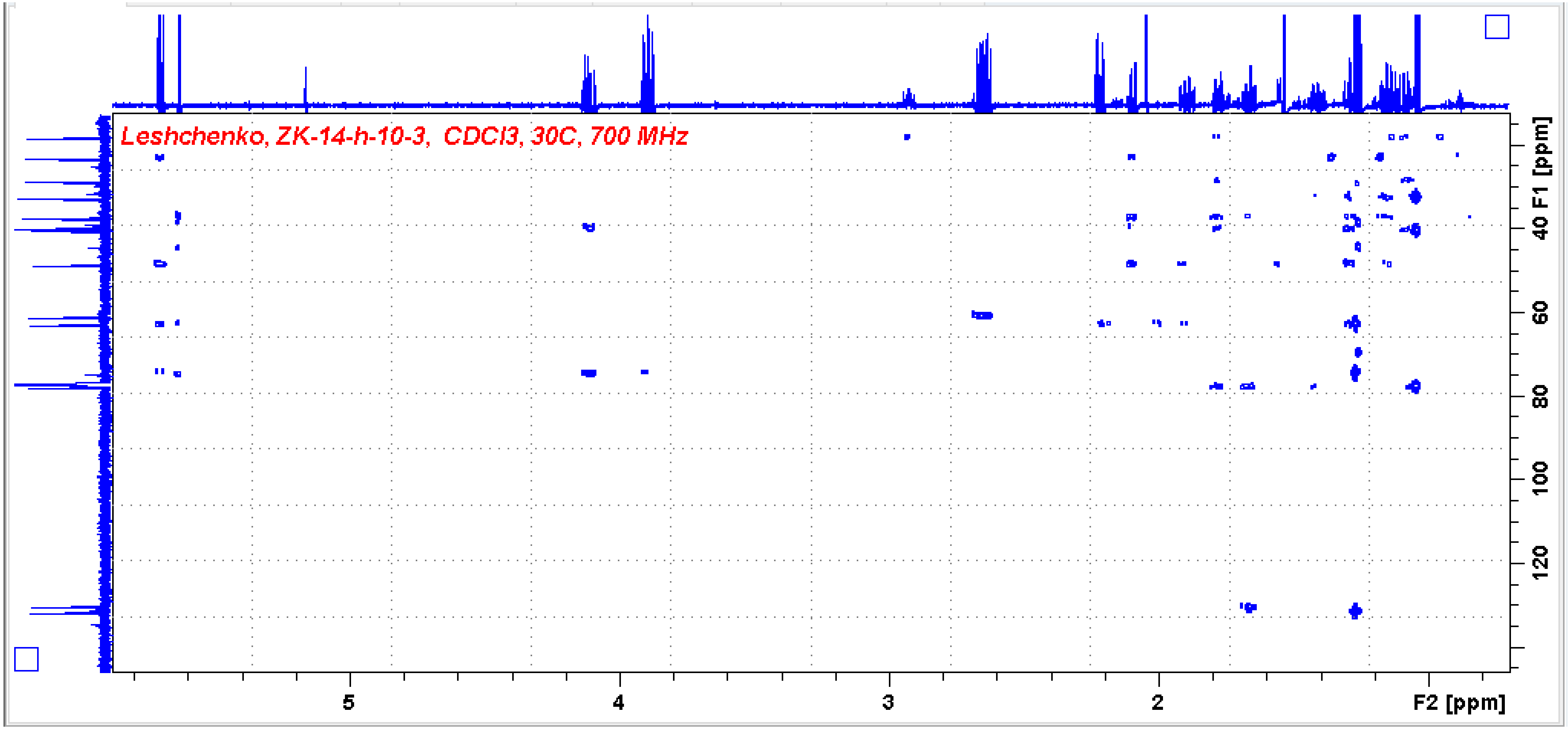

Figure S8. NOESY spectrum of **1** in $CDCl_3$

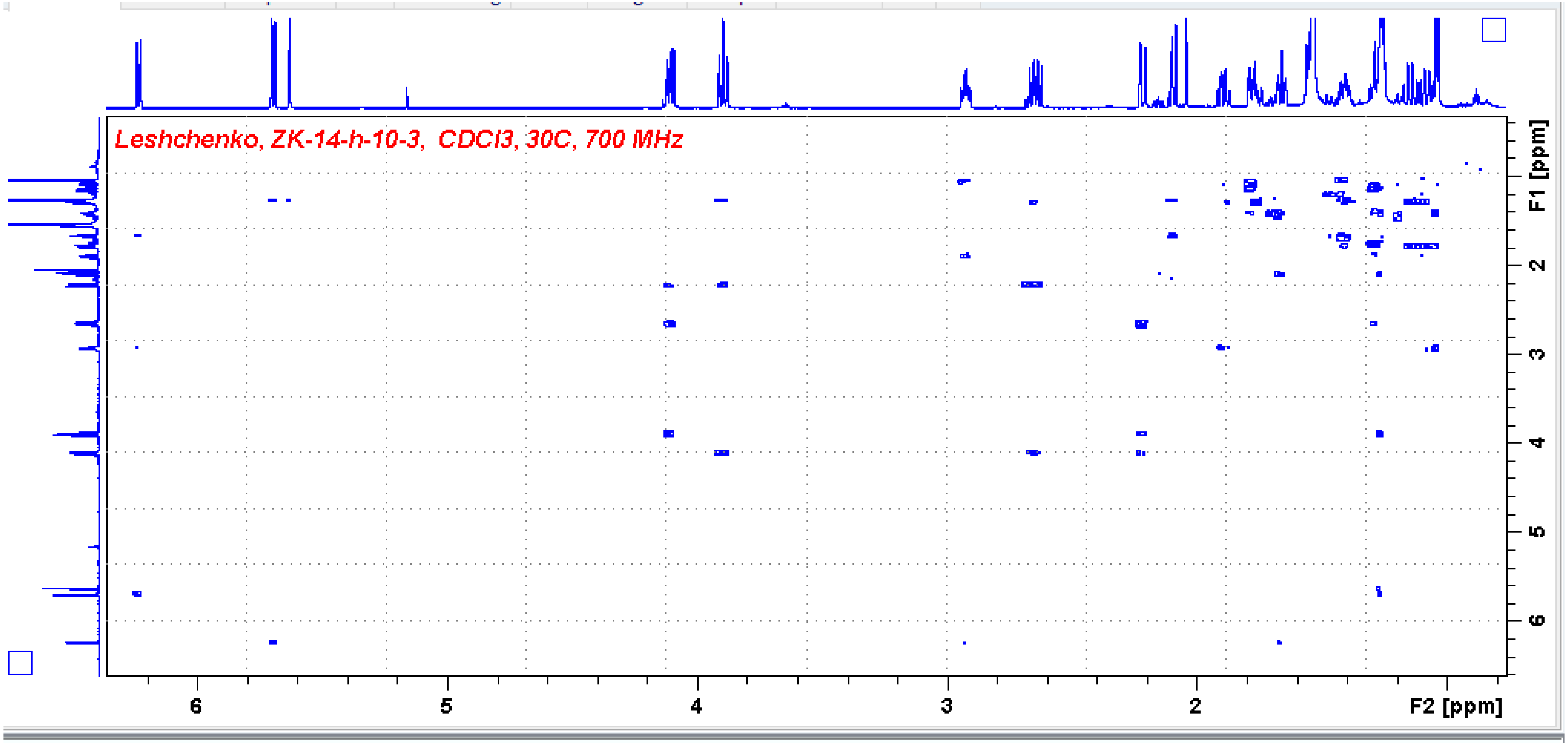

Figure S9. X-Ray data for **1** (Tables 1-6)

Table 1. Crystal data and structure refinement for zosteropenilline A (**1**).

Identification code	t19	
Empirical formula	C15 H22 O3	
Formula weight	250.32	
Temperature	296(2) K	
Wavelength	0.71073 E	
Crystal system	Orthorhombic	
Space group	$P2_12_12_1$	
Unit cell dimensions	a = 5.28750(10) E	α= 90°.
	b = 10.1068(2) E	β= 90°.
	c = 25.6939(5) E	γ = 90°.
Volume	1373.07(5) E^3	
Z	4	
Density (calculated)	1.211 Mg/m^3	
Absorption coefficient	0.083 mm^{-1}	
F(000)	544	
Crystal size	0.330 x 0.200 x 0.110 mm^3	
Theta range for data collection	2.165 to 28.516°.	
Index ranges	-7<=h<=7, -13<=k<=12, -34<=l<=28	
Reflections collected	11438	
Independent reflections	3461 [R(int) = 0.0252]	
Completeness to theta = 25.242°	99.9 %	
Absorption correction	None	
Refinement method	Full-matrix least-squares on F^2	
Data / restraints / parameters	3461 / 0 / 167	
Goodness-of-fit on F^2	1.012	
Final R indices [I>2sigma(I)]	R1 = 0.0371, wR2 = 0.0822	
R indices (all data)	R1 = 0.0590, wR2 = 0.0925	
Absolute structure parameter	0.2(5)	
Extinction coefficient	0.008(2)	
Largest diff. peak and hole	0.147 and -0.137 e.E^{-3}	

Table 2. Atomic coordinates (x 10^4) and equivalent isotropic displacement parameters (E^2x 10^3) for t19. U(eq) is defined as one third of the trace of the orthogonalized U^{ij} tensor.

	x	y	z	U(eq)
O(1)	3979(3)	2357(1)	8534(1)	56(1)
O(2)	-1785(3)	4243(2)	7843(1)	69(1)
O(3)	4966(3)	7079(1)	9853(1)	50(1)
C(1)	3237(5)	2008(3)	8020(1)	78(1)

C(2)	2187(4)	3185(3)	7730(1)	66(1)
C(3)	175(4)	3869(2)	8037(1)	50(1)
C(4)	806(3)	4108(2)	8600(1)	38(1)
C(5)	2613(3)	5297(2)	8654(1)	34(1)
C(6)	1790(4)	6507(2)	8345(1)	44(1)
C(7)	3626(4)	7647(2)	8431(1)	51(1)
C(8)	3950(4)	8001(2)	9000(1)	45(1)
C(9)	4718(3)	6781(2)	9312(1)	37(1)
C(10)	2880(3)	5633(2)	9229(1)	34(1)
C(11)	3615(4)	4422(2)	9531(1)	44(1)
C(12)	3258(4)	3208(2)	9357(1)	48(1)
C(13)	1977(4)	2866(2)	8858(1)	45(1)
C(14)	-40(4)	1816(2)	8952(1)	68(1)
C(15)	5838(5)	9125(2)	9068(1)	70(1)

Table 3. Bond lengths [E] and angles [°] for t19.

O(1)-C(1)	1.421(3)
O(1)-C(13)	1.442(2)
O(2)-C(3)	1.211(3)
O(3)-C(9)	1.428(2)
O(3)-H(3)	0.8200
C(1)-C(2)	1.509(4)
C(1)-H(1A)	0.9700
C(1)-H(1B)	0.9700
C(2)-C(3)	1.494(3)
C(2)-H(2A)	0.9700
C(2)-H(2B)	0.9700
C(3)-C(4)	1.503(3)
C(4)-C(5)	1.541(2)
C(4)-C(13)	1.549(3)
C(4)-H(4)	0.9800
C(5)-C(6)	1.521(2)
C(5)-C(10)	1.522(2)
C(5)-H(5)	0.9800
C(6)-C(7)	1.523(3)
C(6)-H(6A)	0.9700
C(6)-H(6B)	0.9700
C(7)-C(8)	1.515(3)
C(7)-H(7A)	0.9700
C(7)-H(7B)	0.9700
C(8)-C(15)	1.522(3)

C(8)-C(9)	1.525(3)
C(8)-H(8)	0.9800
C(9)-C(10)	1.529(2)
C(9)-H(9)	0.9800
C(10)-C(11)	1.501(2)
C(10)-H(10)	0.9800
C(11)-C(12)	1.319(3)
C(11)-H(11)	0.9300
C(12)-C(13)	1.492(3)
C(12)-H(12)	0.9300
C(13)-C(14)	1.524(3)
C(14)-H(14A)	0.9600
C(14)-H(14B)	0.9600
C(14)-H(14C)	0.9600
C(15)-H(15A)	0.9600
C(15)-H(15B)	0.9600
C(15)-H(15C)	0.9600

C(1)-O(1)-C(13)	114.97(16)
C(9)-O(3)-H(3)	109.5
O(1)-C(1)-C(2)	111.35(18)
O(1)-C(1)-H(1A)	109.4
C(2)-C(1)-H(1A)	109.4
O(1)-C(1)-H(1B)	109.4
C(2)-C(1)-H(1B)	109.4
H(1A)-C(1)-H(1B)	108.0
C(3)-C(2)-C(1)	111.5(2)
C(3)-C(2)-H(2A)	109.3
C(1)-C(2)-H(2A)	109.3
C(3)-C(2)-H(2B)	109.3
C(1)-C(2)-H(2B)	109.3
H(2A)-C(2)-H(2B)	108.0
O(2)-C(3)-C(2)	122.4(2)
O(2)-C(3)-C(4)	122.5(2)
C(2)-C(3)-C(4)	115.09(18)
C(3)-C(4)-C(5)	110.48(15)
C(3)-C(4)-C(13)	111.66(16)
C(5)-C(4)-C(13)	110.22(14)
C(3)-C(4)-H(4)	108.1
C(5)-C(4)-H(4)	108.1
C(13)-C(4)-H(4)	108.1
C(6)-C(5)-C(10)	110.68(14)

C(6)-C(5)-C(4)	113.72(15)
C(10)-C(5)-C(4)	108.59(14)
C(6)-C(5)-H(5)	107.9
C(10)-C(5)-H(5)	107.9
C(4)-C(5)-H(5)	107.9
C(5)-C(6)-C(7)	110.50(15)
C(5)-C(6)-H(6A)	109.6
C(7)-C(6)-H(6A)	109.6
C(5)-C(6)-H(6B)	109.6
C(7)-C(6)-H(6B)	109.6
H(6A)-C(6)-H(6B)	108.1
C(8)-C(7)-C(6)	113.00(16)
C(8)-C(7)-H(7A)	109.0
C(6)-C(7)-H(7A)	109.0
C(8)-C(7)-H(7B)	109.0
C(6)-C(7)-H(7B)	109.0
H(7A)-C(7)-H(7B)	107.8
C(7)-C(8)-C(15)	111.19(18)
C(7)-C(8)-C(9)	110.24(16)
C(15)-C(8)-C(9)	111.59(16)
C(7)-C(8)-H(8)	107.9
C(15)-C(8)-H(8)	107.9
C(9)-C(8)-H(8)	107.9
O(3)-C(9)-C(8)	111.42(15)
O(3)-C(9)-C(10)	110.78(15)
C(8)-C(9)-C(10)	111.76(14)
O(3)-C(9)-H(9)	107.6
C(8)-C(9)-H(9)	107.6
C(10)-C(9)-H(9)	107.6
C(11)-C(10)-C(5)	110.14(14)
C(11)-C(10)-C(9)	112.47(15)
C(5)-C(10)-C(9)	111.37(14)
C(11)-C(10)-H(10)	107.5
C(5)-C(10)-H(10)	107.5
C(9)-C(10)-H(10)	107.5
C(12)-C(11)-C(10)	123.14(17)
C(12)-C(11)-H(11)	118.4
C(10)-C(11)-H(11)	118.4
C(11)-C(12)-C(13)	124.84(18)
C(11)-C(12)-H(12)	117.6
C(13)-C(12)-H(12)	117.6
O(1)-C(13)-C(12)	104.26(15)

O(1)-C(13)-C(14)	110.89(16)
C(12)-C(13)-C(14)	110.03(18)
O(1)-C(13)-C(4)	109.65(16)
C(12)-C(13)-C(4)	111.19(15)
C(14)-C(13)-C(4)	110.66(16)
C(13)-C(14)-H(14A)	109.5
C(13)-C(14)-H(14B)	109.5
H(14A)-C(14)-H(14B)	109.5
C(13)-C(14)-H(14C)	109.5
H(14A)-C(14)-H(14C)	109.5
H(14B)-C(14)-H(14C)	109.5
C(8)-C(15)-H(15A)	109.5
C(8)-C(15)-H(15B)	109.5
H(15A)-C(15)-H(15B)	109.5
C(8)-C(15)-H(15C)	109.5
H(15A)-C(15)-H(15C)	109.5
H(15B)-C(15)-H(15C)	109.5

Symmetry transformations used to generate equivalent atoms:

Table 4. Anisotropic displacement parameters (E^2x 10^3) for t19. The anisotropic displacement factor exponent takes the form: $-2\pi^2[h^2 a^{*2}U^{11} + ... + 2 h k a^* b^* U^{12}]$

	U^{11}	U^{22}	U^{33}	U^{23}	U^{13}	U^{12}
O(1)	46(1)	50(1)	72(1)	-16(1)	2(1)	6(1)
O(2)	47(1)	100(1)	60(1)	-3(1)	-16(1)	-7(1)
O(3)	47(1)	59(1)	43(1)	-16(1)	-5(1)	-3(1)
C(1)	68(2)	78(2)	86(2)	-43(2)	4(2)	7(2)
C(2)	55(1)	92(2)	52(1)	-31(1)	-3(1)	-7(1)
C(3)	40(1)	58(1)	52(1)	-9(1)	-5(1)	-12(1)
C(4)	31(1)	43(1)	40(1)	-4(1)	0(1)	-1(1)
C(5)	32(1)	38(1)	33(1)	-1(1)	-2(1)	-1(1)
C(6)	44(1)	48(1)	40(1)	7(1)	-7(1)	-1(1)
C(7)	51(1)	48(1)	54(1)	17(1)	-5(1)	-5(1)
C(8)	37(1)	35(1)	63(1)	2(1)	-4(1)	0(1)
C(9)	33(1)	40(1)	39(1)	-6(1)	-2(1)	2(1)
C(10)	33(1)	36(1)	33(1)	-1(1)	0(1)	1(1)
C(11)	51(1)	47(1)	35(1)	5(1)	-6(1)	2(1)
C(12)	52(1)	41(1)	52(1)	11(1)	-4(1)	2(1)
C(13)	38(1)	38(1)	58(1)	-2(1)	2(1)	-3(1)
C(14)	53(1)	48(1)	102(2)	11(1)	0(1)	-13(1)

C(15)	64(1)	42(1)	103(2)	5(1)	-16(1)	-12(1)

Table 5. Hydrogen coordinates (x 10^4) and isotropic displacement parameters (E^2x 10^{3}) for t19.

	x	y	z	U(eq)
H(3)	3589	7305	9970	75
H(1A)	1964	1318	8036	93
H(1B)	4687	1661	7833	93
H(2A)	3545	3803	7657	79
H(2B)	1490	2893	7401	79
H(4)	-766	4322	8784	46
H(5)	4278	5024	8525	41
H(6A)	107	6772	8454	53
H(6B)	1726	6289	7978	53
H(7A)	5261	7409	8287	61
H(7B)	3020	8418	8244	61
H(8)	2310	8305	9132	54
H(9)	6381	6498	9186	45
H(10)	1217	5918	9354	41
H(11)	4357	4532	9856	53
H(12)	3848	2514	9561	58
H(14A)	740	1028	9085	102
H(14B)	-879	1616	8630	102
H(14C)	-1251	2141	9199	102
H(15A)	7459	8855	8938	105
H(15B)	5982	9340	9431	105
H(15C)	5264	9887	8880	105

Table 6. Hydrogen bonds for t19 [E and °].

D-H...A	d(D-H)	d(H...A)	d(D...A)	<(DHA)
O(3)-H(3)...O(3)#1	0.82	2.07	2.8785(10)	171.3
C(2)-H(2A)...O(2)#2	0.97	2.55	3.374(3)	142.3

Symmetry transformations used to generate equivalent atoms:

#1 x-1/2,-y+3/2,-z+2 #2 x+1,y,z

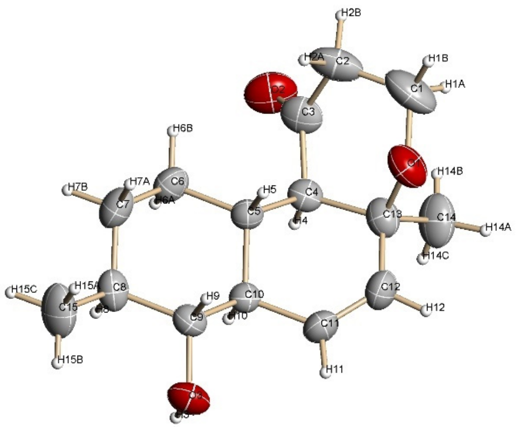

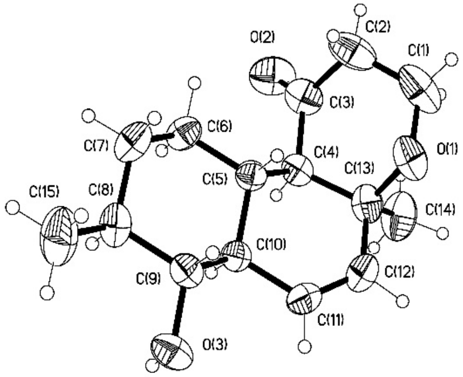

Crystal structure of **1**

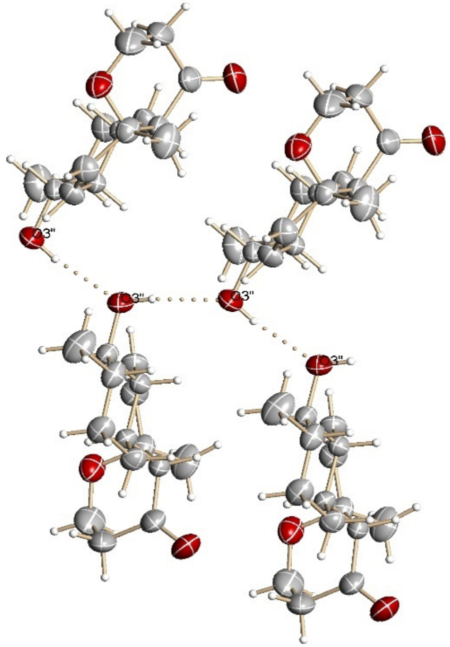

Polymer chain fragment of **1**

Figure S10. ^{1}H and ^{13}C NMR Spectroscopic for S-MTPA ester of 1 (1a) and R-MTPA ester of 1 (1b).

Table 1. Δδ(δ_S- δ_R) values (in ppm) for the (*S*)- and (*R*)-MPTA esters of **1**.

Position	δ_C (mult) at 176.4 in $CDCl_3$	(*S*)-MTPA ester (1a) at 700.13 in $CDCl_3$ δ_H (*J*, Hz)	(*R*)-MTPA ester (1b) at 700.13 in $CDCl_3$ δ_H (*J*, Hz)	Δδ(δS- δR) values (in ppm)
1	60.9, CH_2	4.10, dd (12.0, 8.1)	4.10, dd (11.7, 8.1)	0
		3.88, td (12.0, 2.9)	3.88, td (11.8, 2.8)	0
2	39.9, CH_2	2.64, ddd (13.8, 12.1, 8.0)	2.64, ddd (13.9, 12.1, 8.2)	0
		2.23, brd (13.8)	2.23, brd (13.8)	0
3	209.0, C			
4	62.3, CH	2.04, d (11.1)	2.07, d (10.8)	-0,03
5	37.2, CH	1.96, qd (11.5, 3.3)	1.99, qd (10.9, 3.1)	-0,03
6	28.3, CH_2	1.30, dq (13.1, 3.2)	1.30, m	0
		1.14, qd (11.3, 3.0)	1.14, m	0
7	32.7, CH_2	1.84, dq (13.8, 3.4)	1.81, m	+0,03
		1.20, m	1,17	+0,03
8	38.1, CH	1.69, m	1,66, m	+0,03
9	81.7, CH	4.69 t (10.4)	4.70, t (10.5)	-0,01
10	45.6, CH	1.92, tt (10.7, 1.8)	1.95, brt (10.5)	-0,03
11	127.9, CH	5.34 d (10.3)	5.54, d (10.1)	-0,20
12	132.3, CH	5.53 dd (10.3, 2.5)	5.62, dd (10.1, 2.1)	-0,09
13	74.4, C			
14	23.0, CH_3	1.20, s	1.24, s	-0,04
15	18.5, CH_3	0.90, d (6.3)	0.81, d (6.5)	+0,09

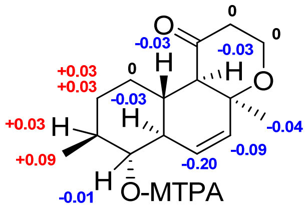

4*R*, 5*S*, 8*S*, 9*R*, 10*R*, 13*S* – **1**

^{1}H NMR spectrum (700.13 MHz) of (*S*)- and (*R*)-MPTA esters of **1** in $CDCl_3$

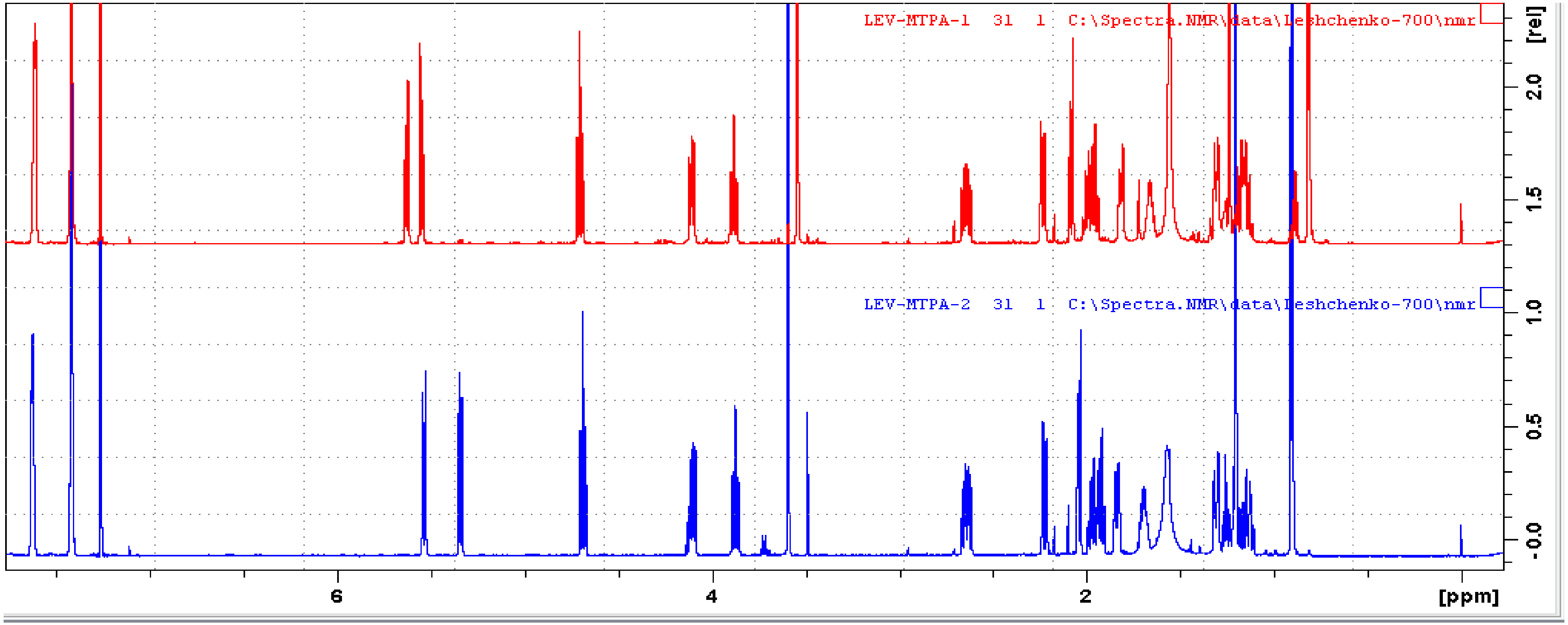

*LEV-MTPA-2 - (*S*)-MTPA ester (1a); LEV-MTPA-1 - (*R*)-MTPA ester (1b)

Figure S11. ^{1}H NMR spectrum (500.13 MHz) of **2** in $CDCl_3$

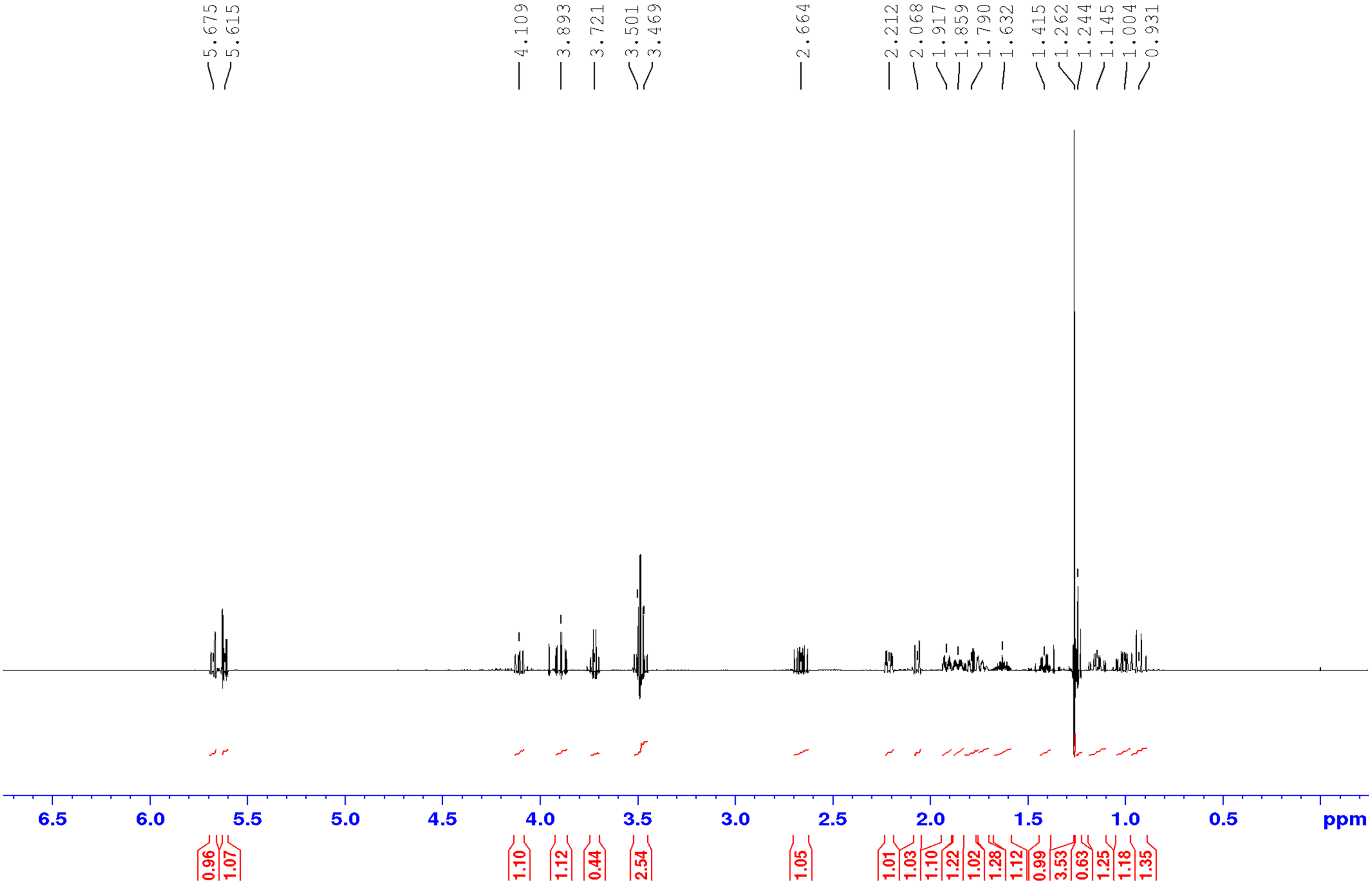

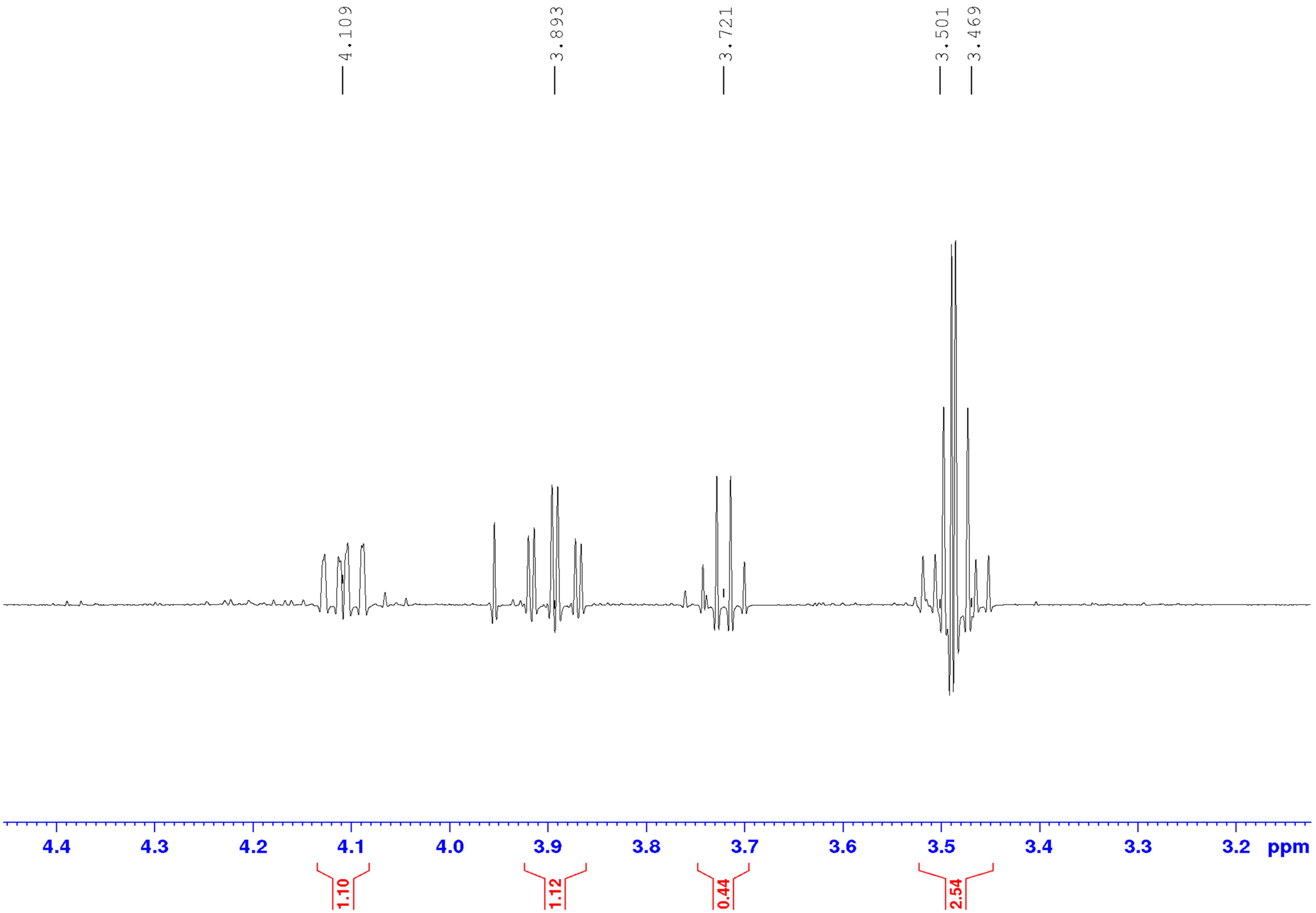
4.109
3.893
3.721
3.501
3.469
4.4
4.3
4.2
4.1
4.0
3.9
3.8
3.7
3.6
3.5
3.4
3.3
3.2
ppm
1.10
1.12
0.44
2.54

Figure S12. ^{13}C NMR spectrum (125.77 MHz) of **2** in $CDCl_3$

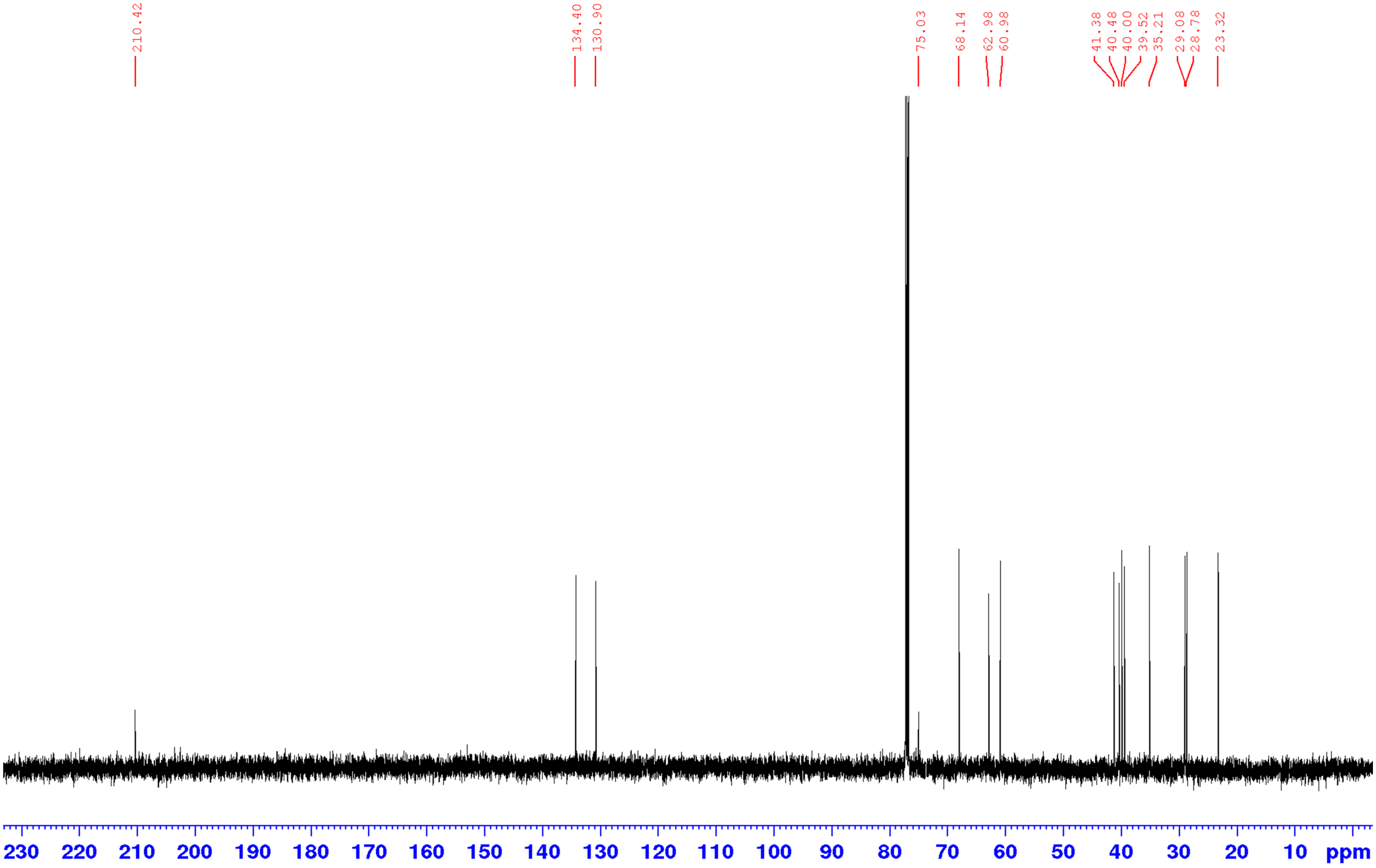

Figure S13. DEPT-135 spectrum (125.77 MHz) of **2** in $CDCl_3$

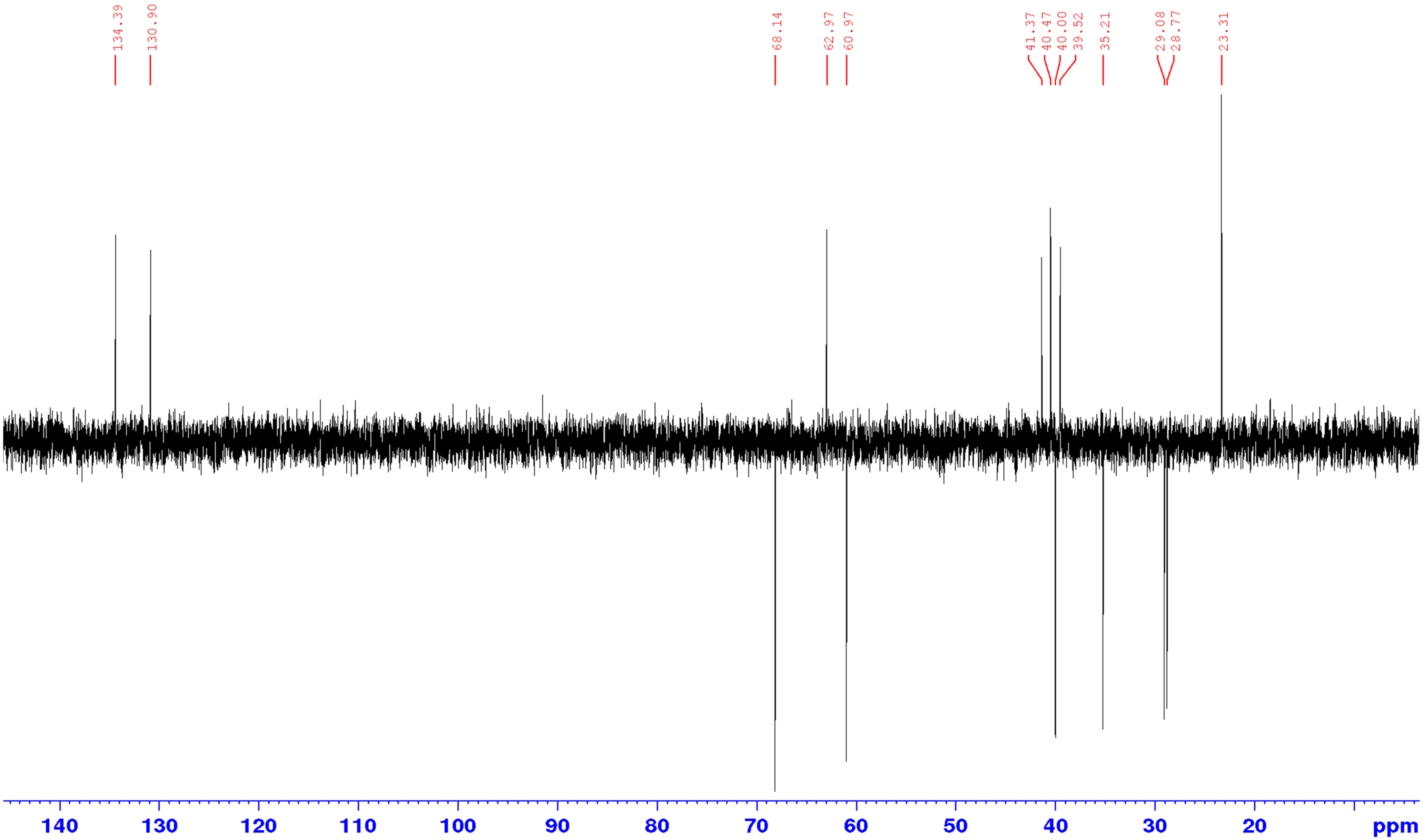

Figure S14. DEPT-90 spectrum (125.77 MHz) of **2** in $CDCl_3$

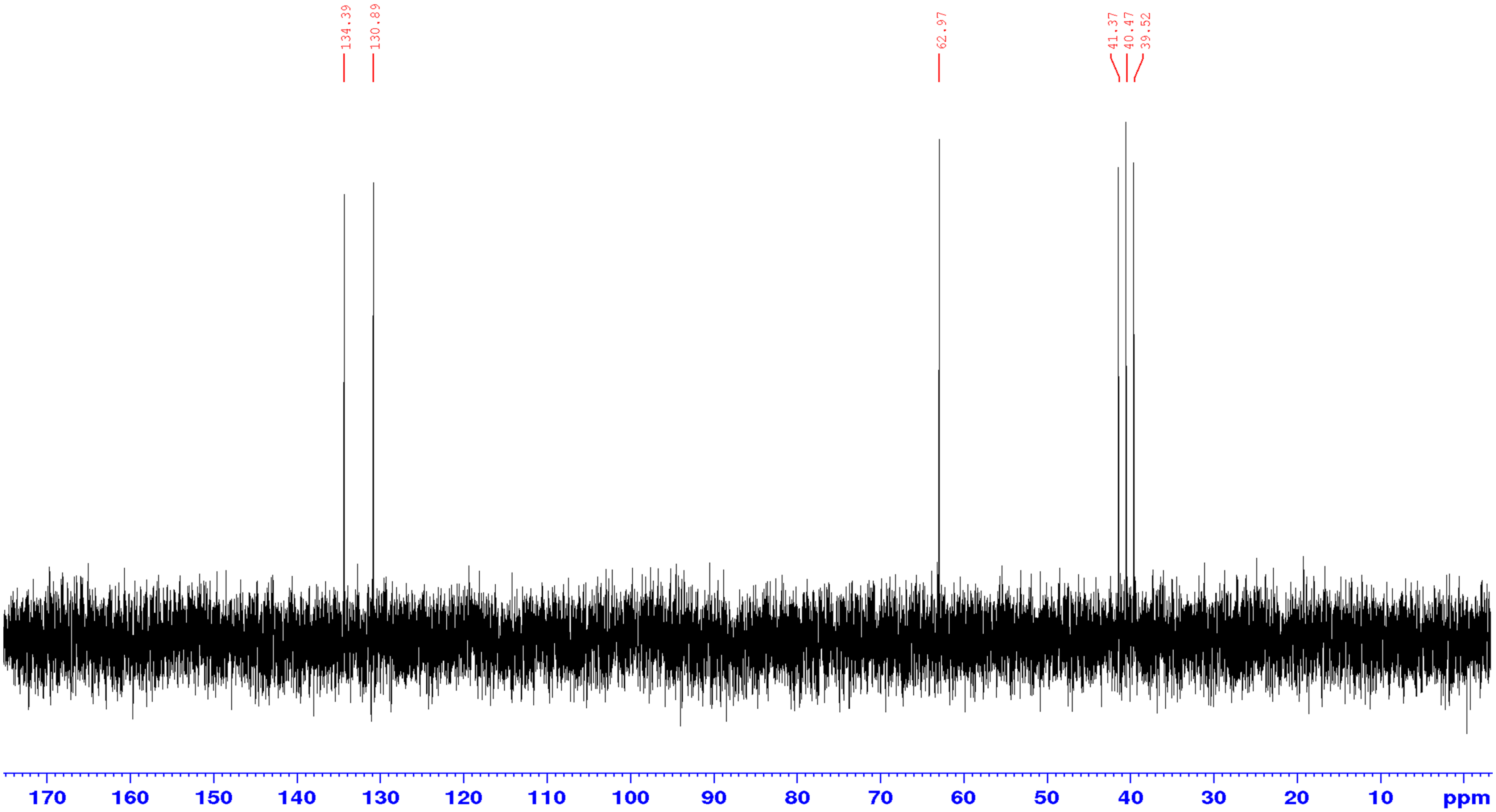

Figure S15. HSQC spectrum of **2** in $CDCl_3$

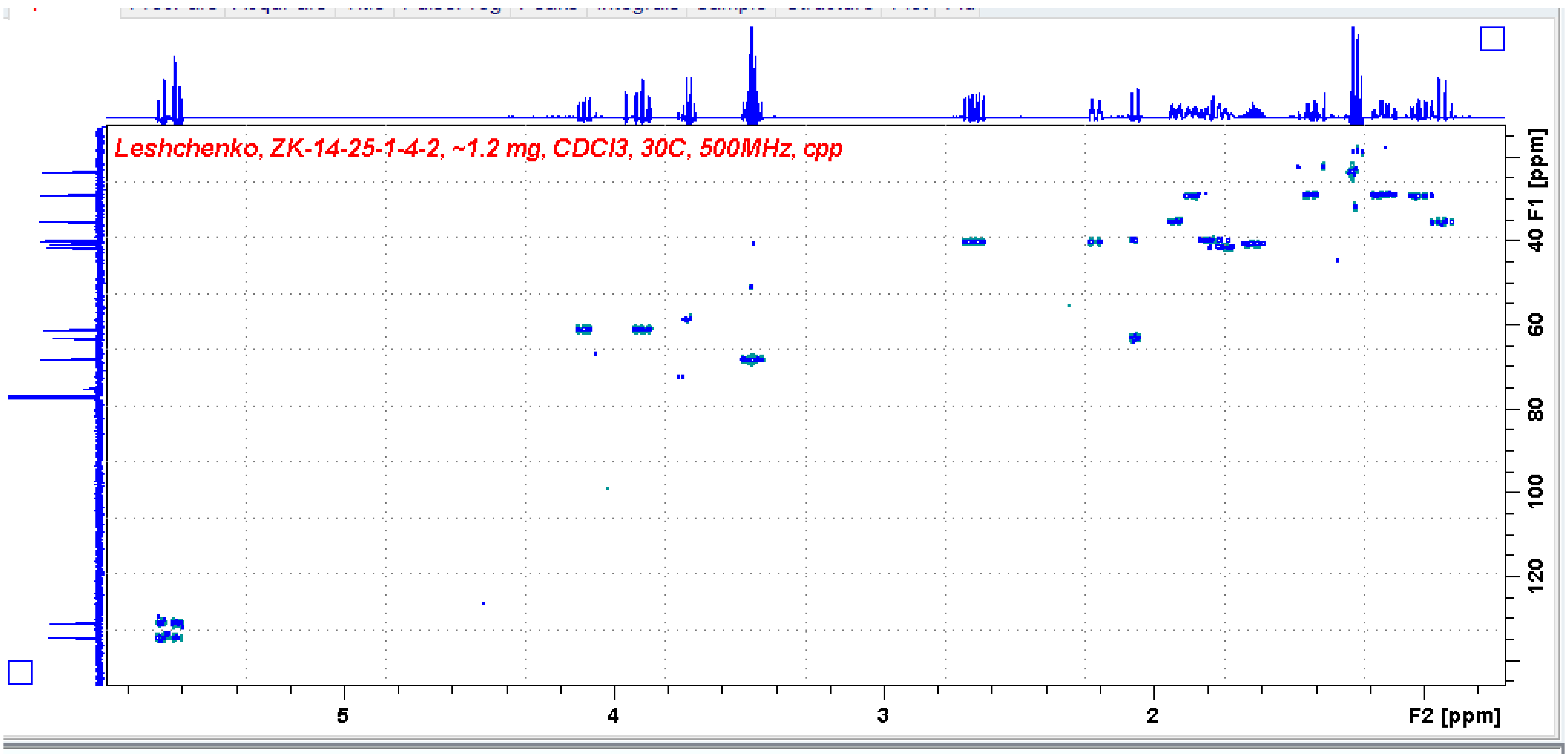

Figure S16. HMBC spectrum of **2** in $CDCl_3$

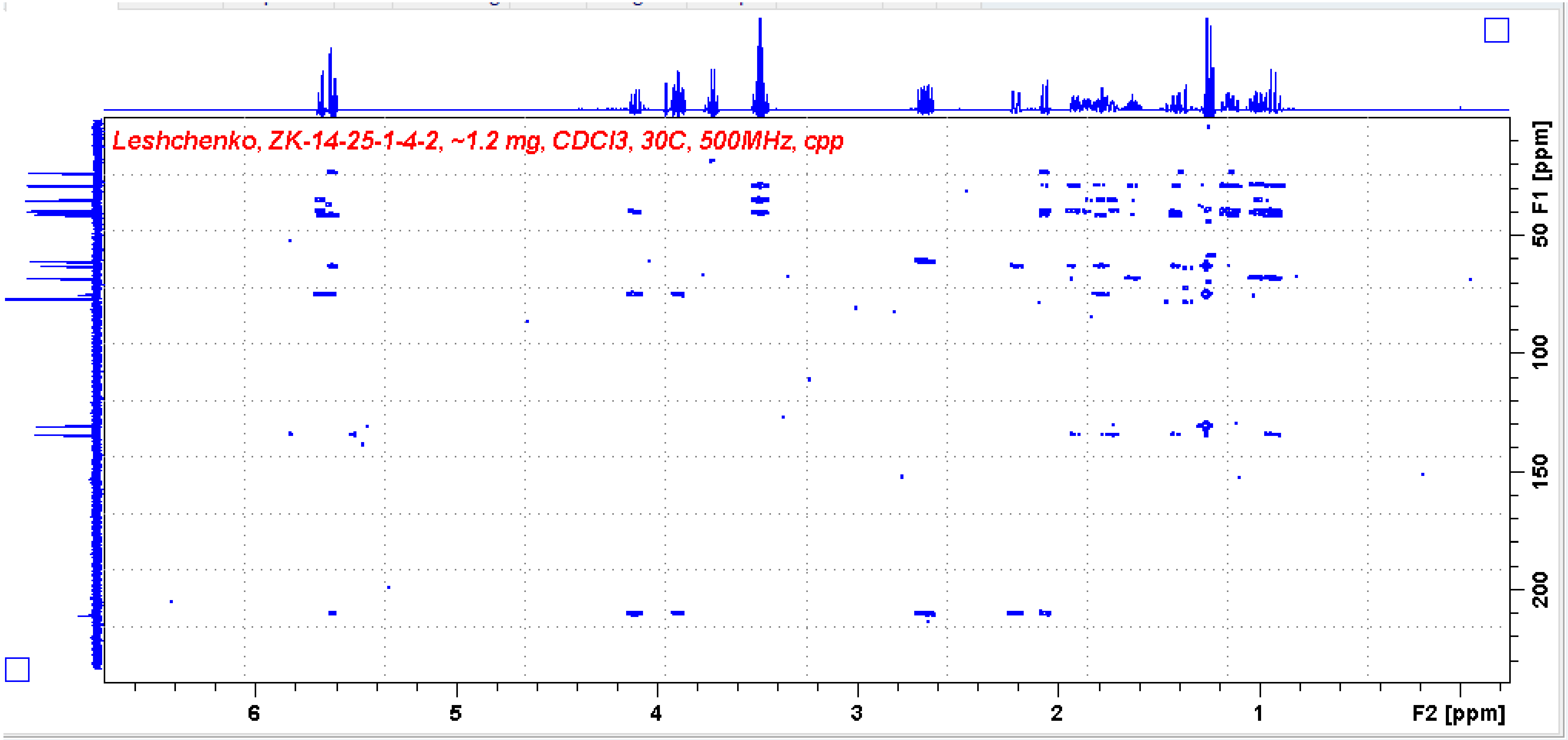

Figure S17. 1H-1H COSY spectrum of **2** in $CDCl_3$

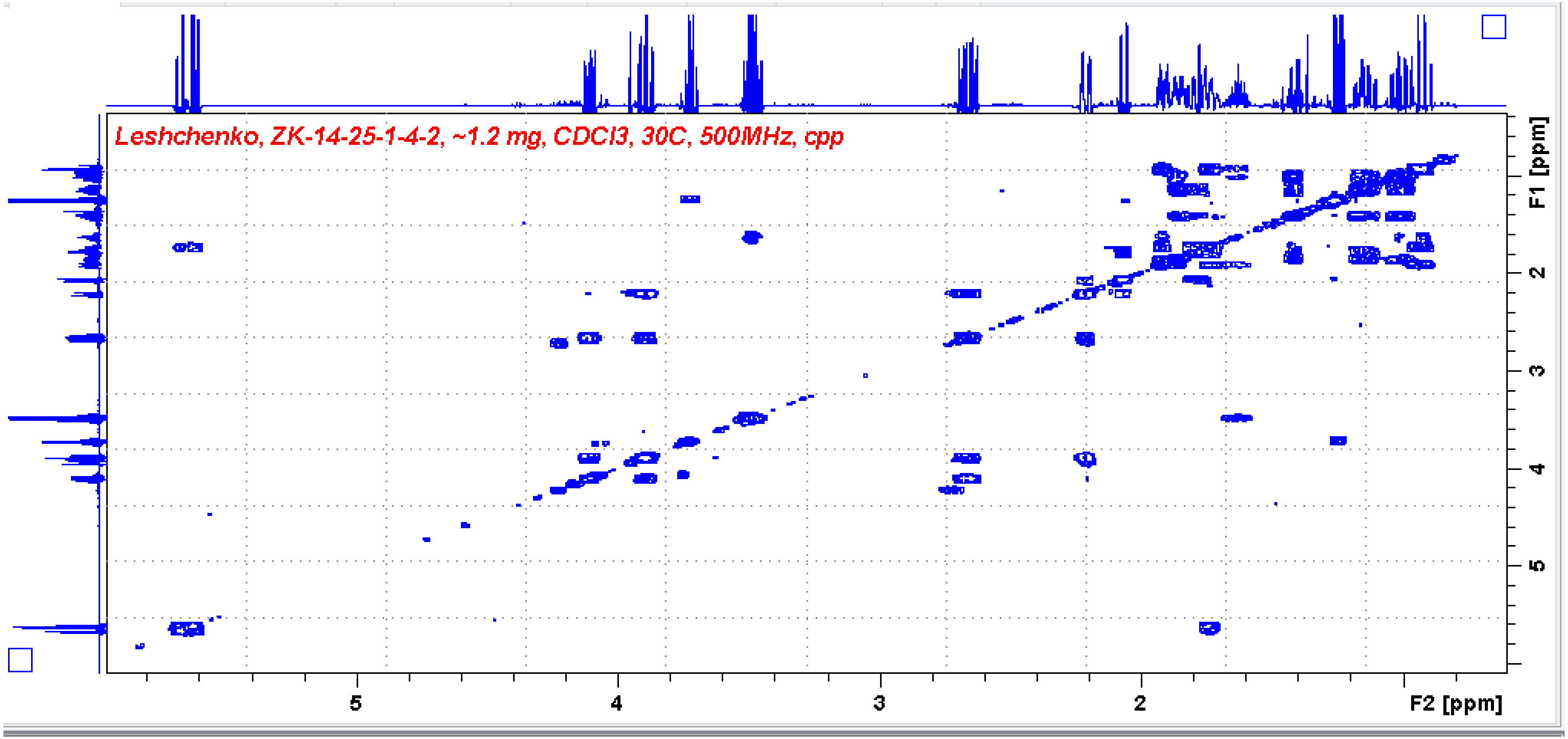

Figure S19. NOESY spectrum of **2** in $CDCl_3$

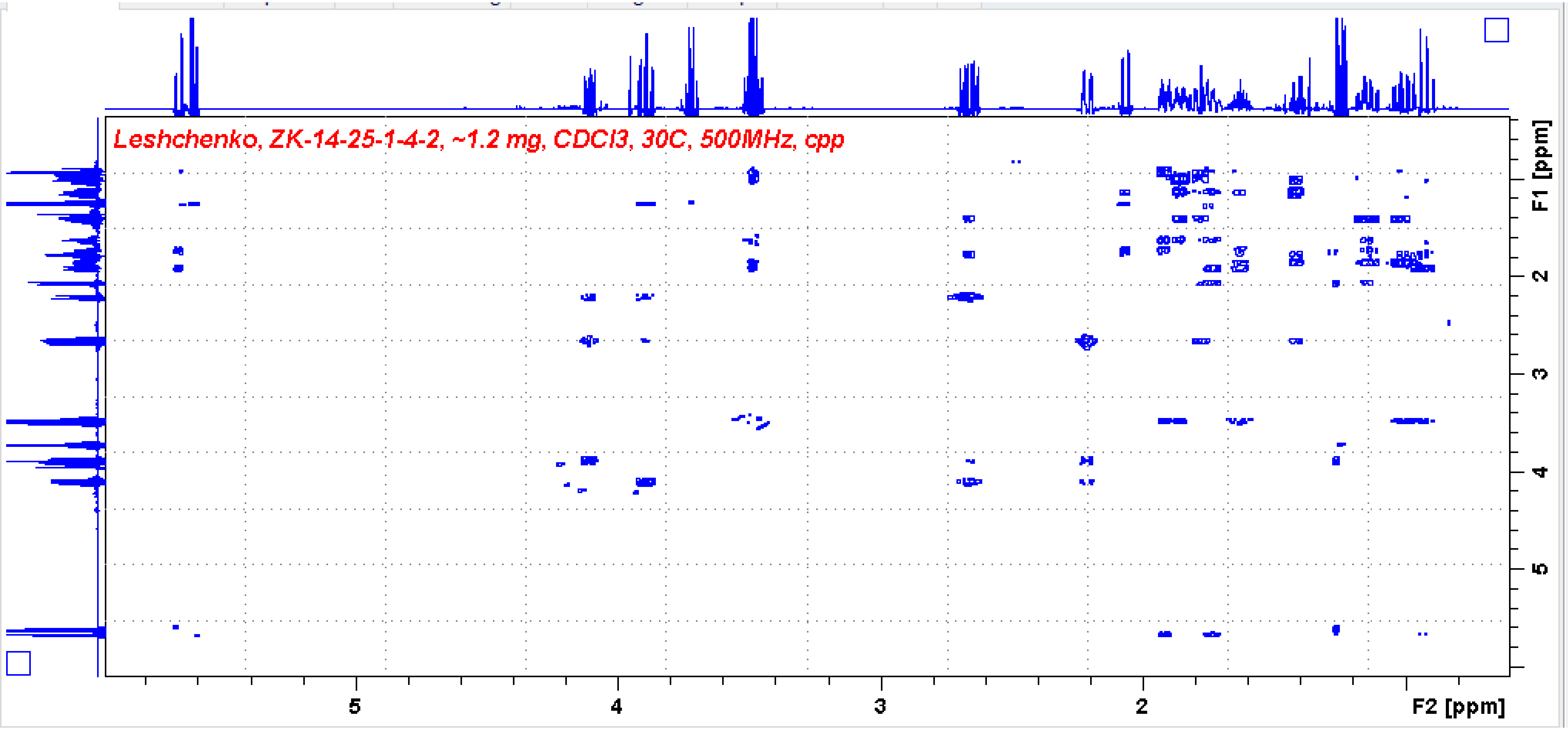

Figure S20. ^{1}H NMR spectrum (500.13 MHz) of **3** in $CDCl_3$

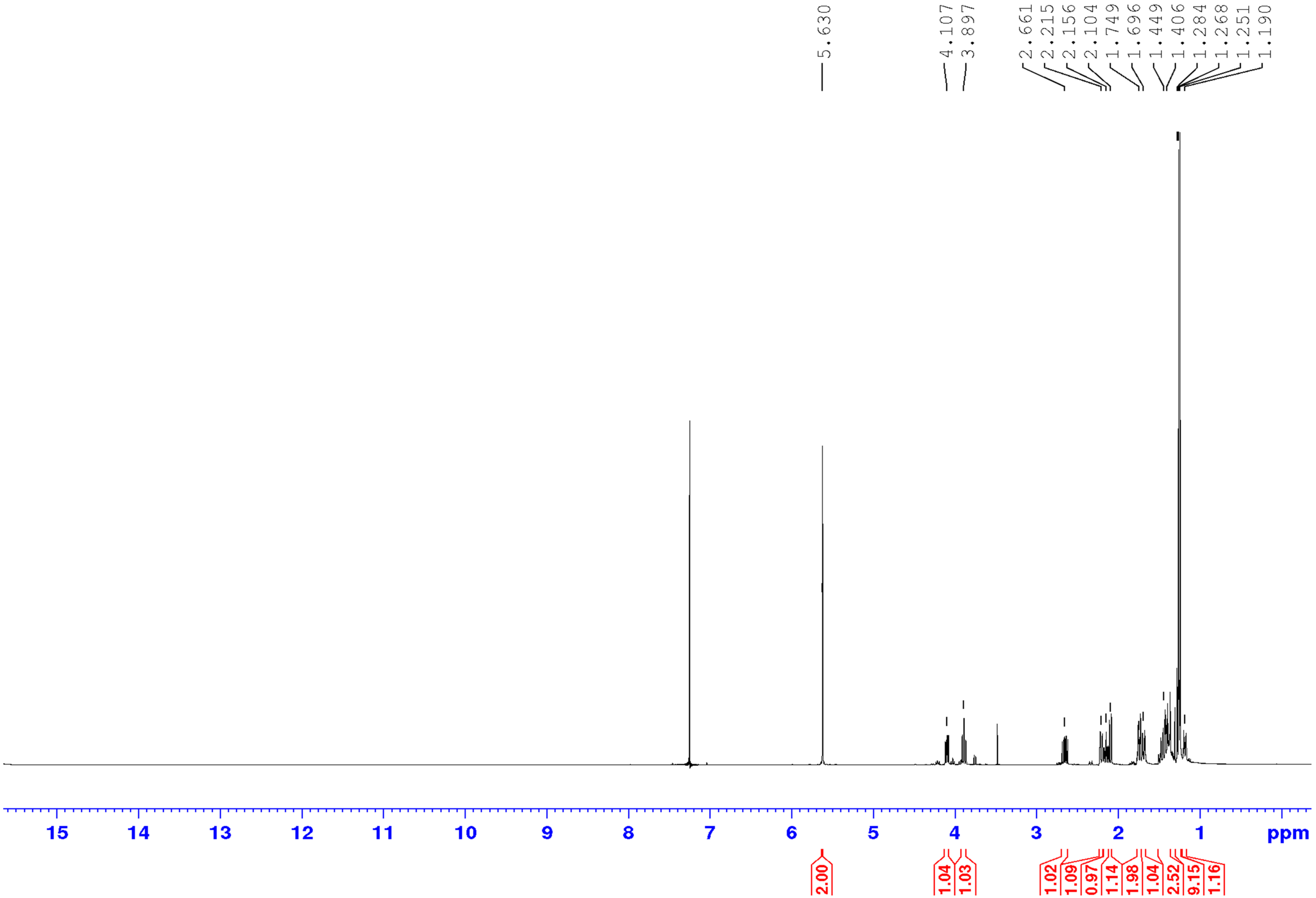

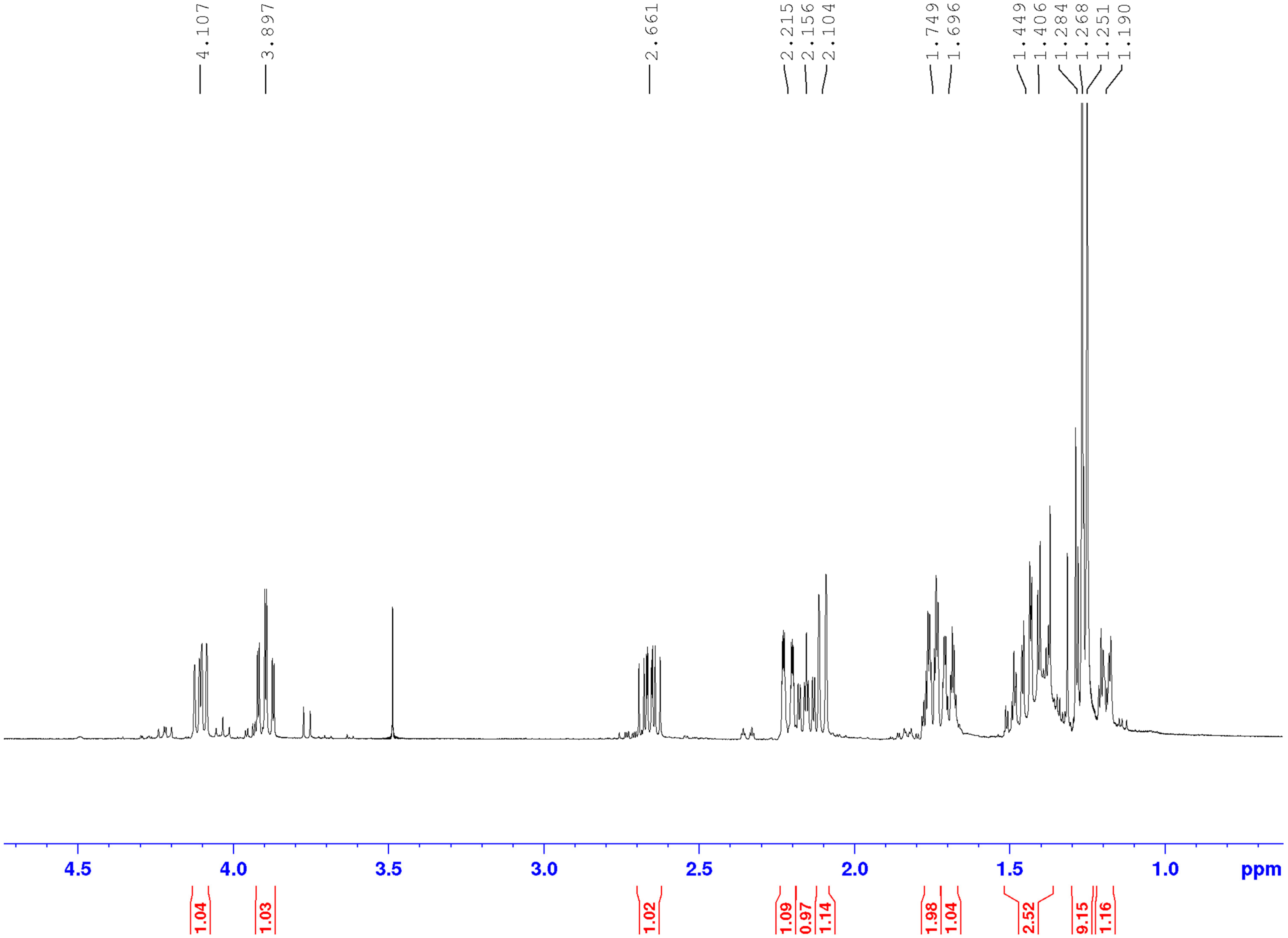
4.107
3.897
2.661
2.215
2.156
2.104
1.749
1.696
1.449
1.406
1.284
1.268
1.251
1.190
4.5
4.0
3.5
3.0
2.5
2.0
1.5
1.0
ppm
1.04
1.03
1.02
1.09
0.97
1.14
1.98
1.04
2.52
9.15
1.16

Figure S21. ^{13}C NMR spectrum (125.77 MHz) of **3** in $CDCl_3$

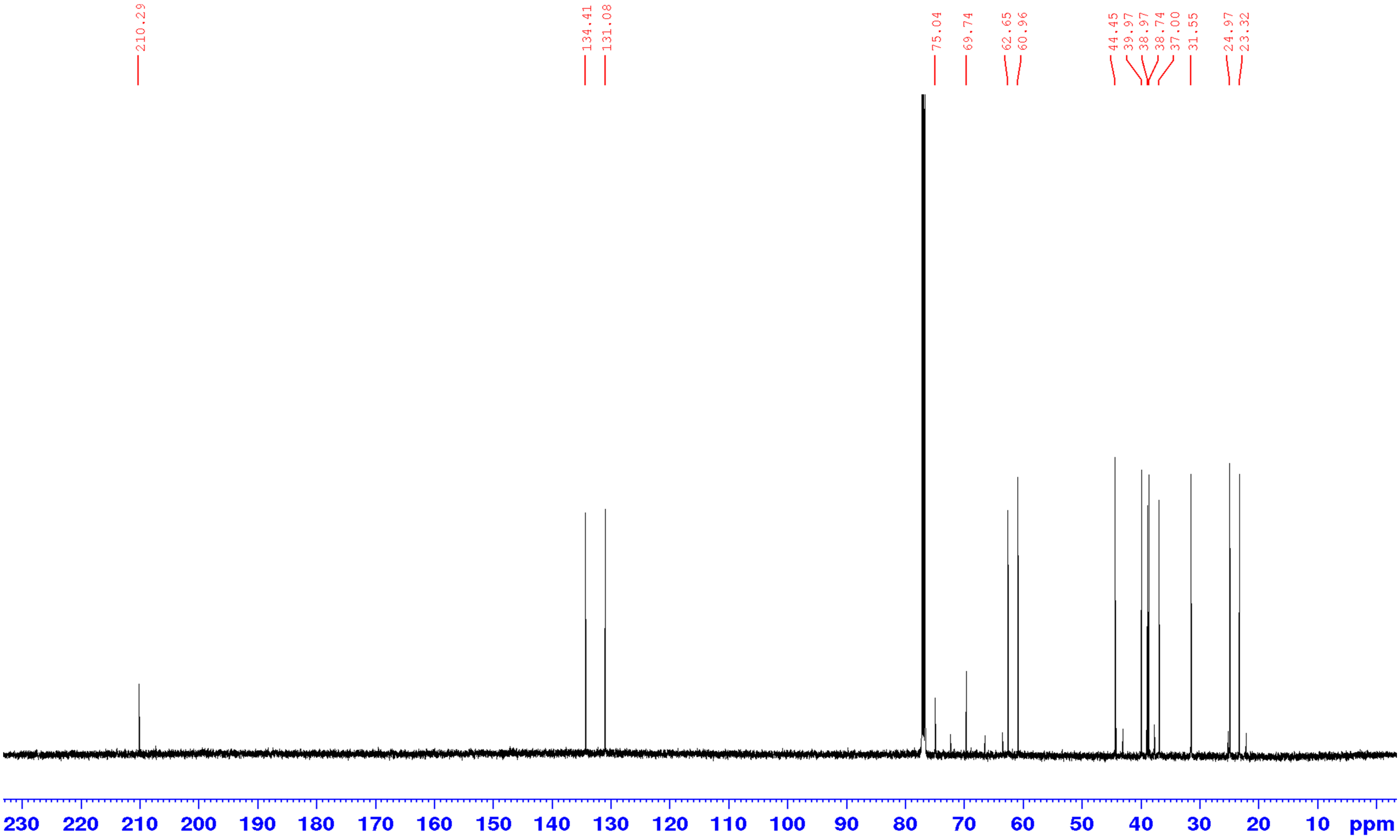

Figure S22. DEPT-135 spectrum (125.77 MHz) of **3** in $CDCl_3$

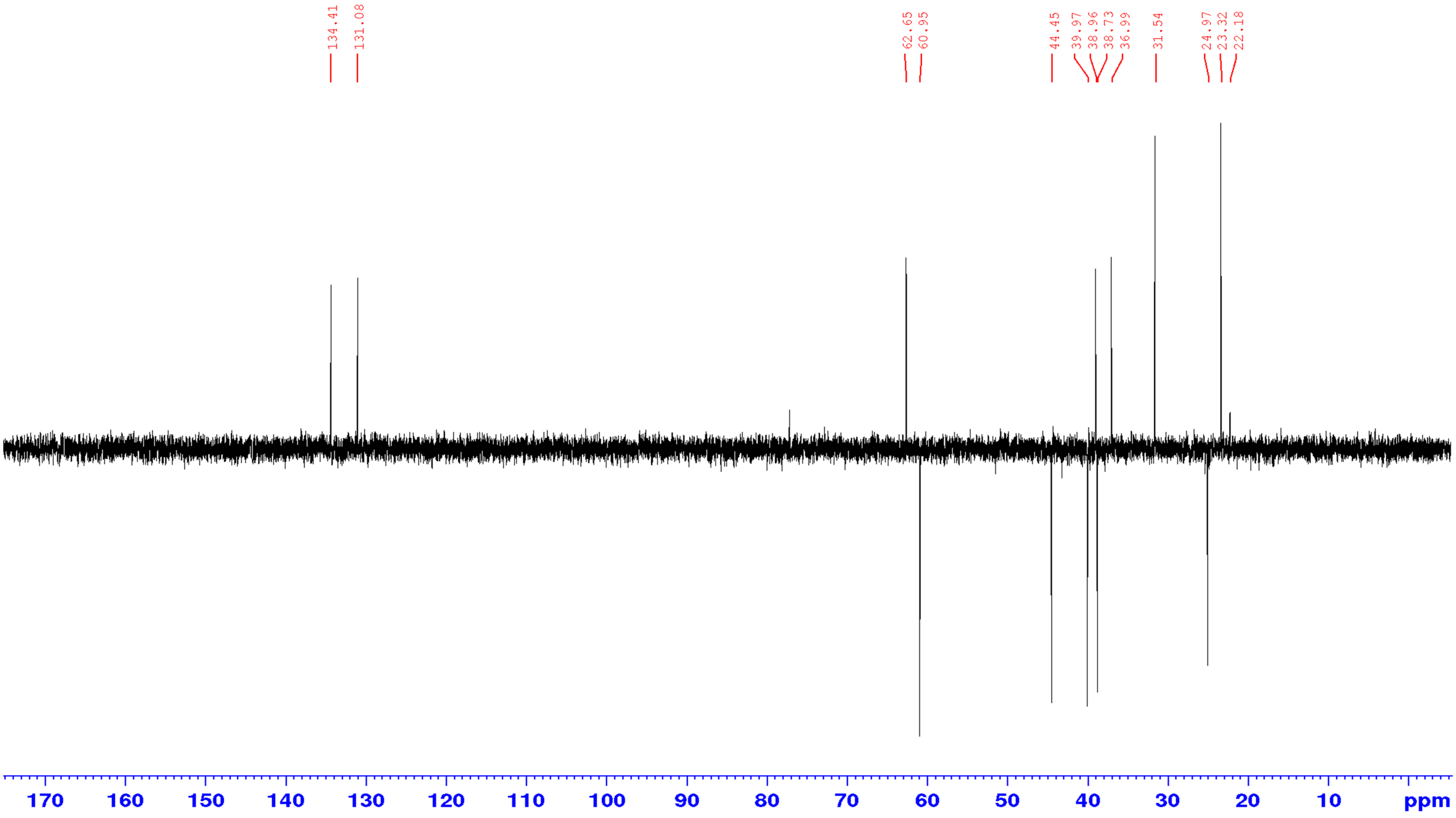

Figure S23. DEPT-90 spectrum (125.77 MHz) of **3** in $CDCl_3$

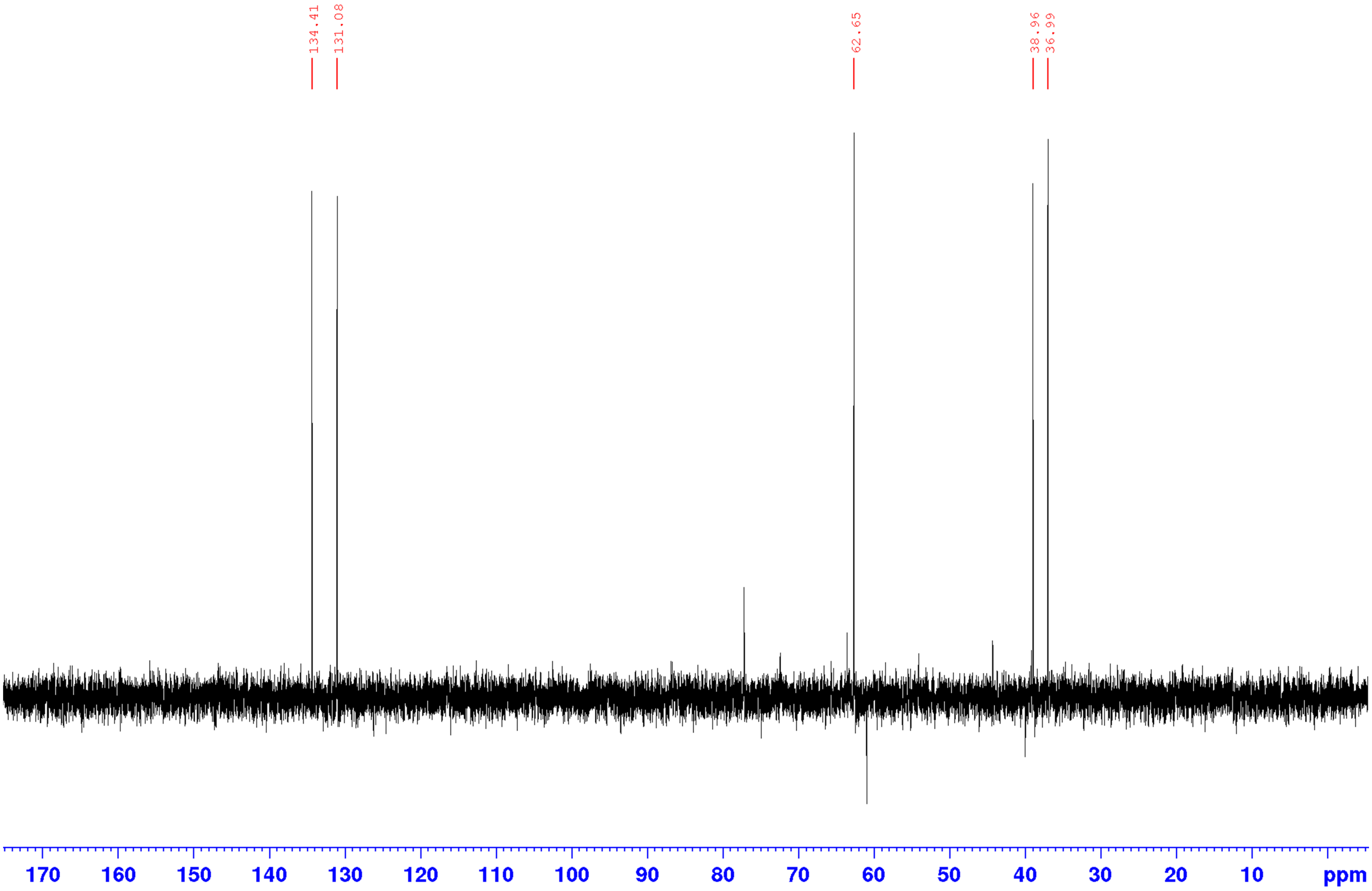

Figure S24. HSQC spectrum of **3** in $CDCl_3$

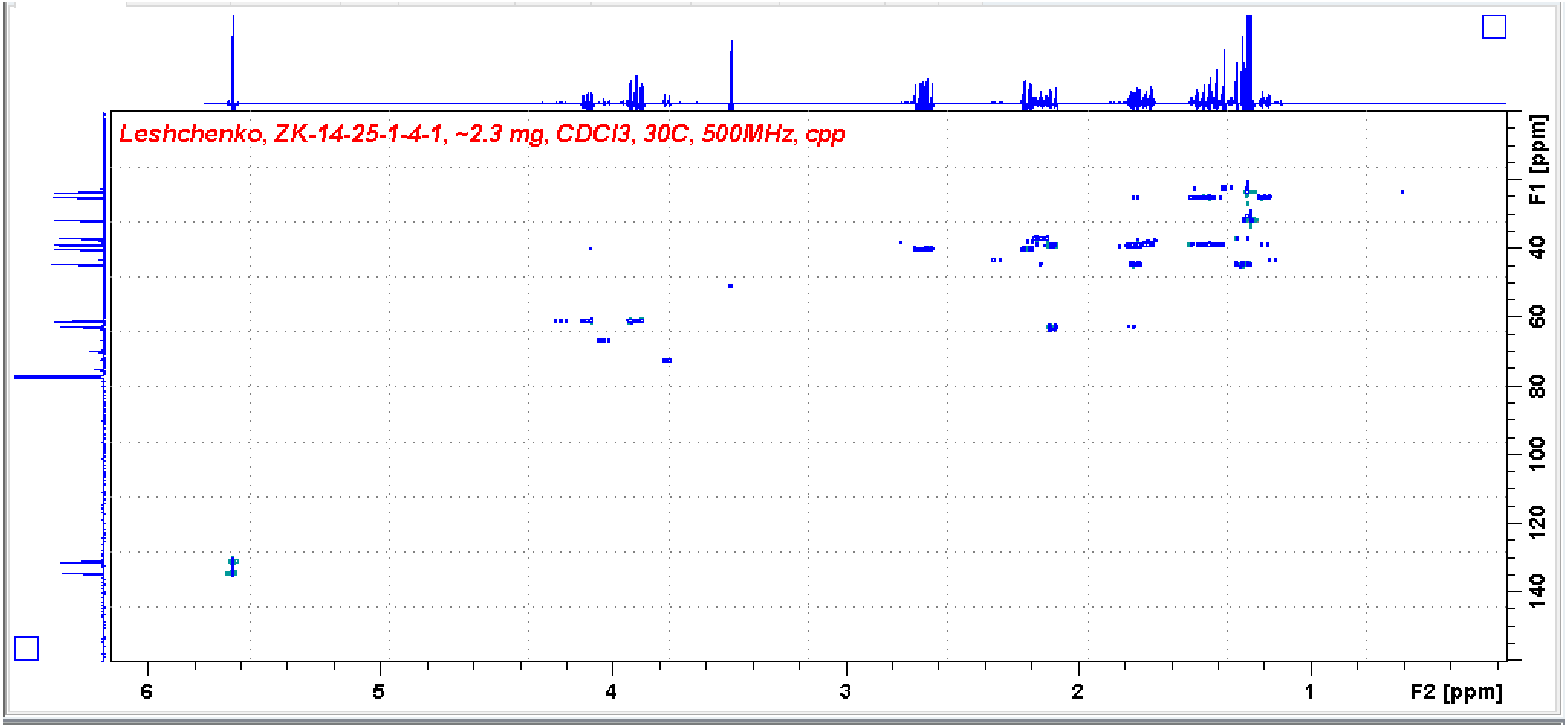

Figure S25. HMBC spectrum of **3** in $CDCl_3$

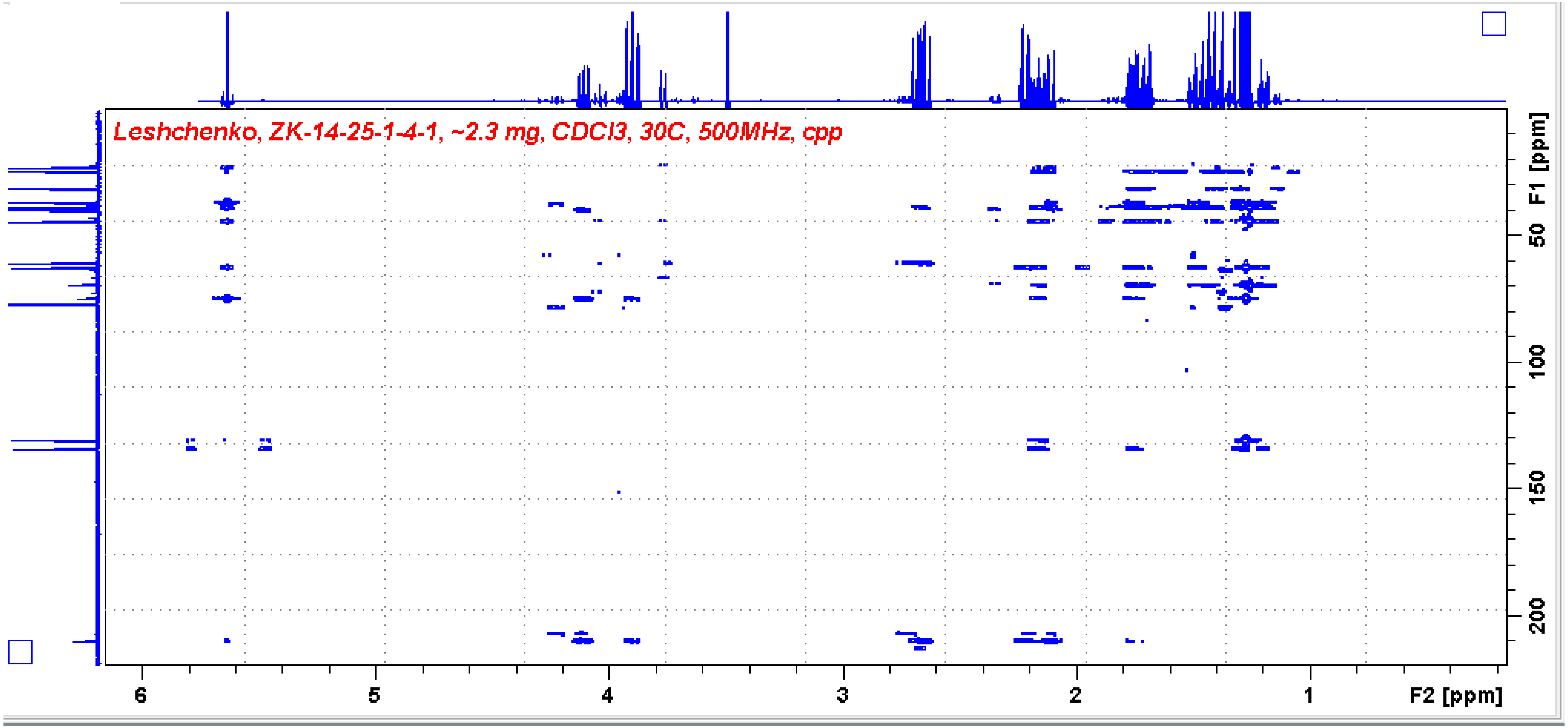

Figure S26. 1H-1H COSY spectrum of **3** in $CDCl_3$

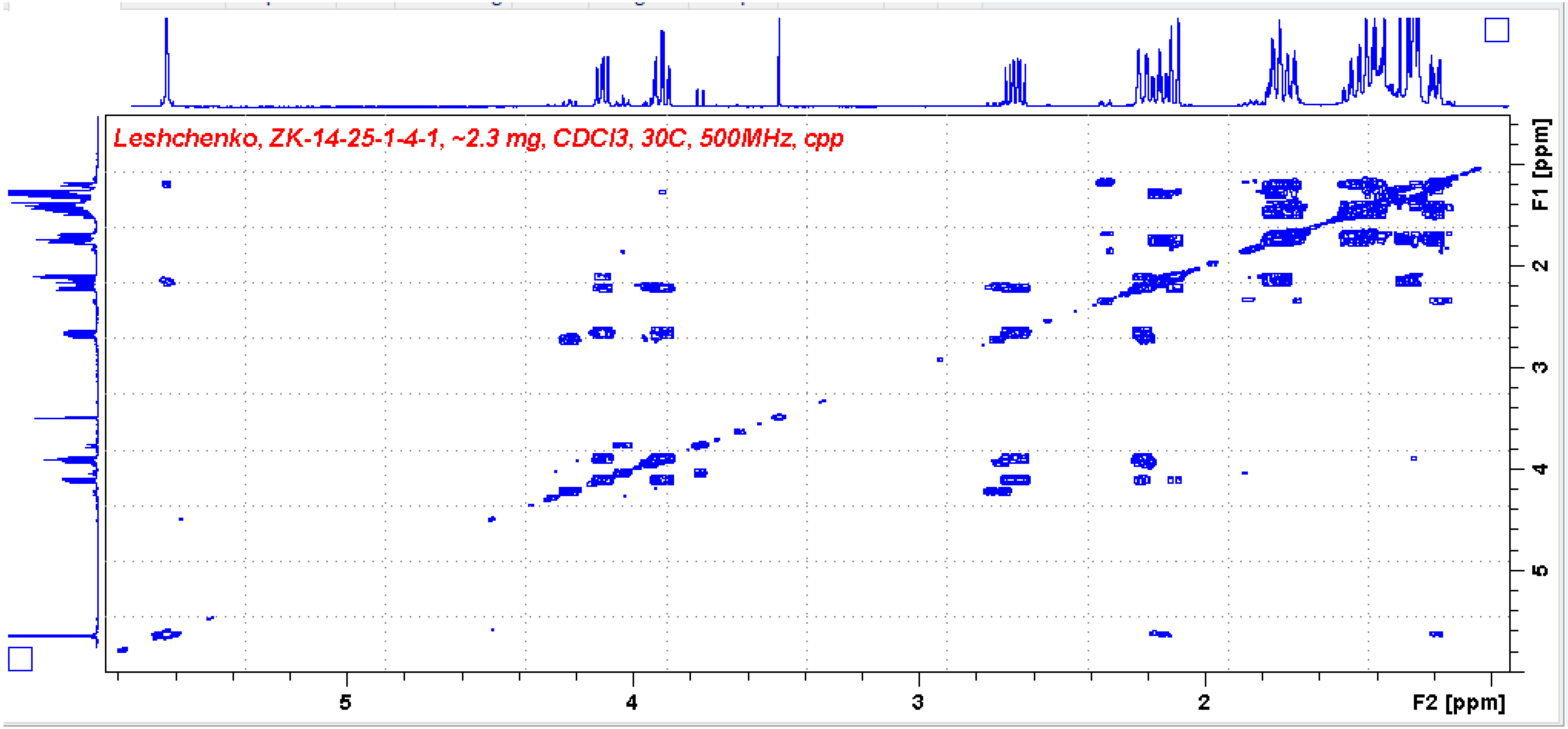

Figure S27. NOESY spectrum of **3** in $CDCl_3$

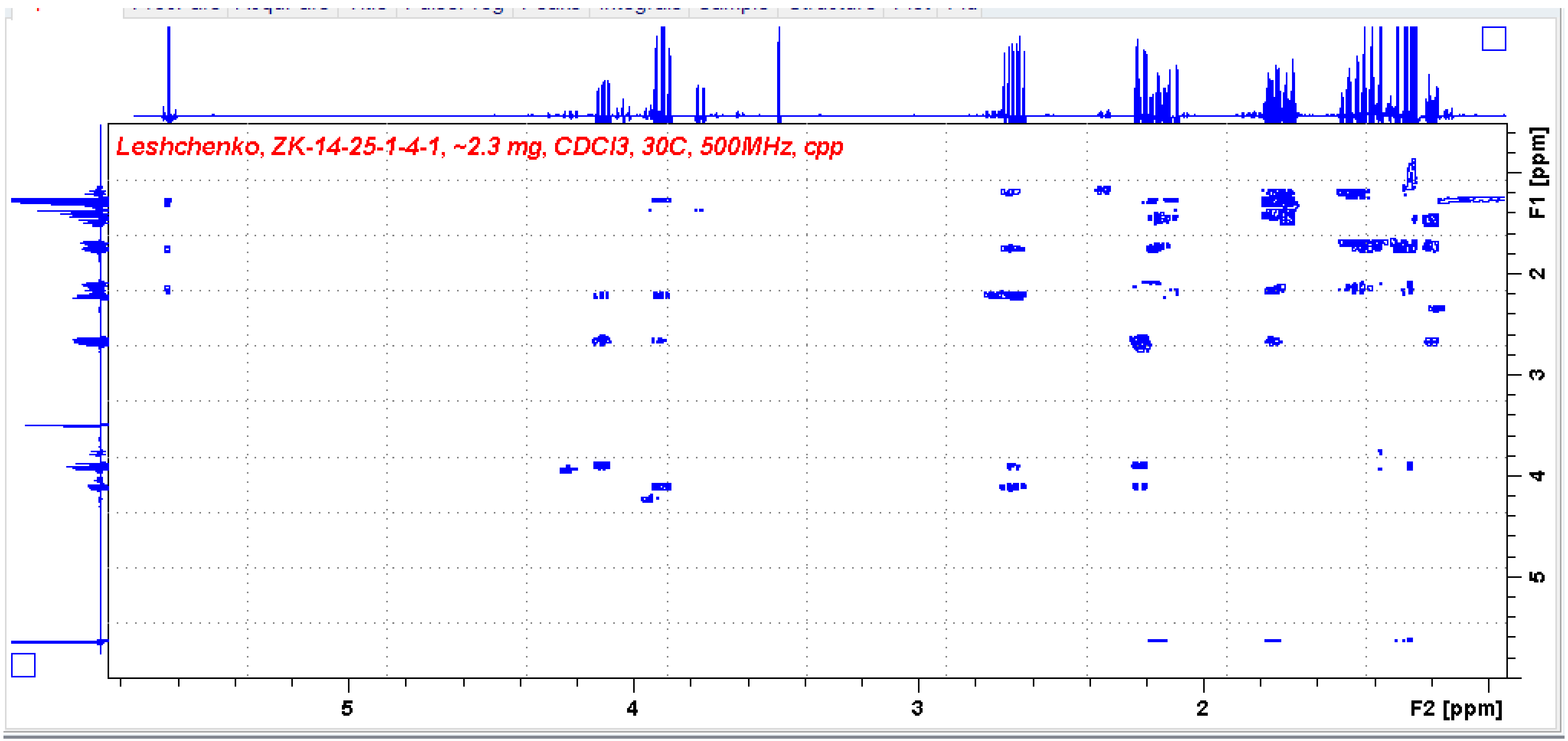

Figure S28. ^{1}H NMR spectrum (700.13 MHz) of **4** in $CDCl_3$

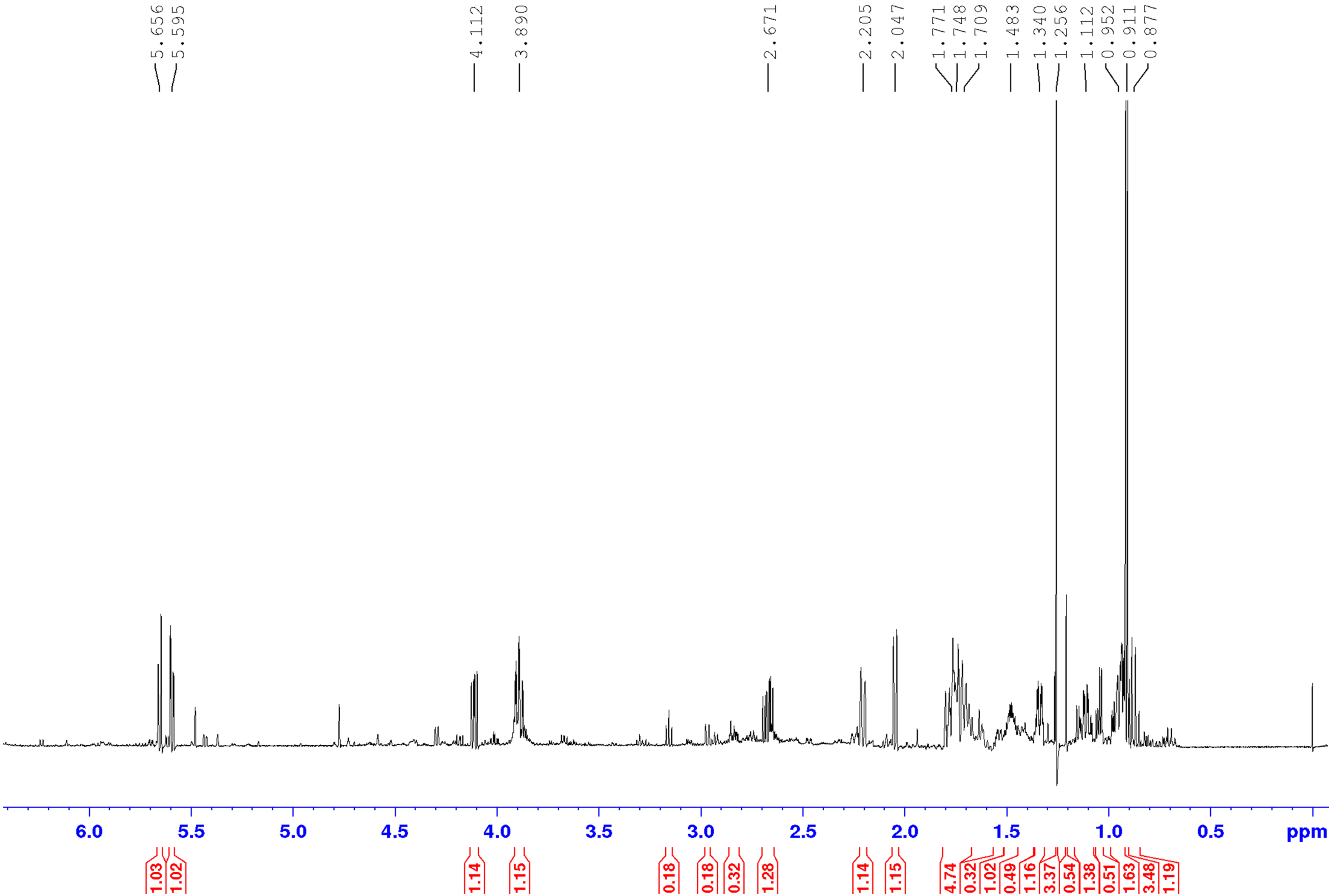

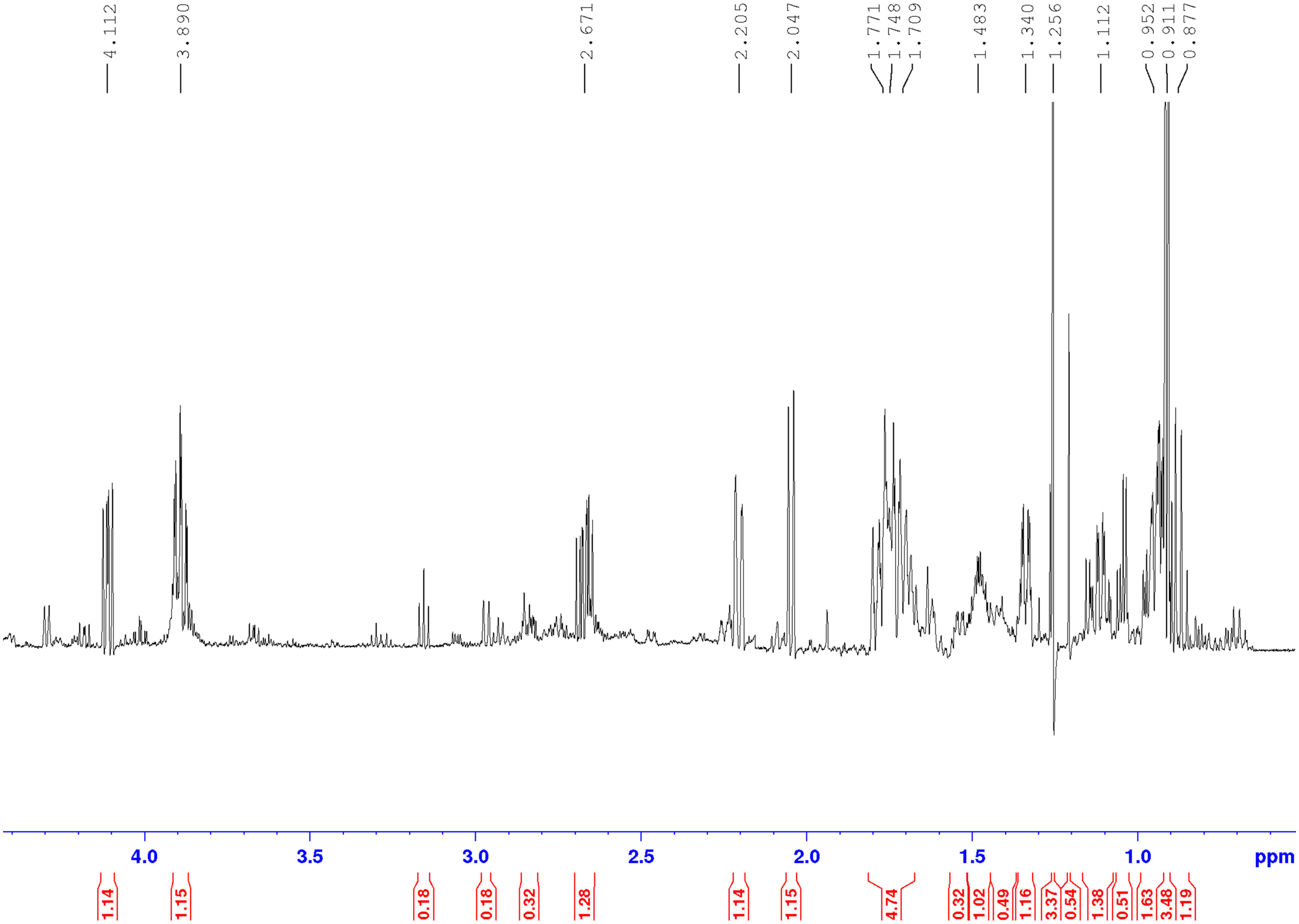

4.112
3.890
2.671
2.205
2.047
1.771
1.748
1.709
1.483
1.340
1.256
1.112
0.952
0.911
0.877
4.0
3.5
3.0
2.5
2.0
1.5
1.0
ppm
1.14
1.15
0.18
0.18
0.32
1.28
1.14
1.15
4.74
0.32
1.02
0.49
1.16
3.37
0.54
1.38
0.51
1.63
3.48
1.19

Figure S29. ^{13}C NMR spectrum (176.04 MHz) of **4** in $CDCl_3$

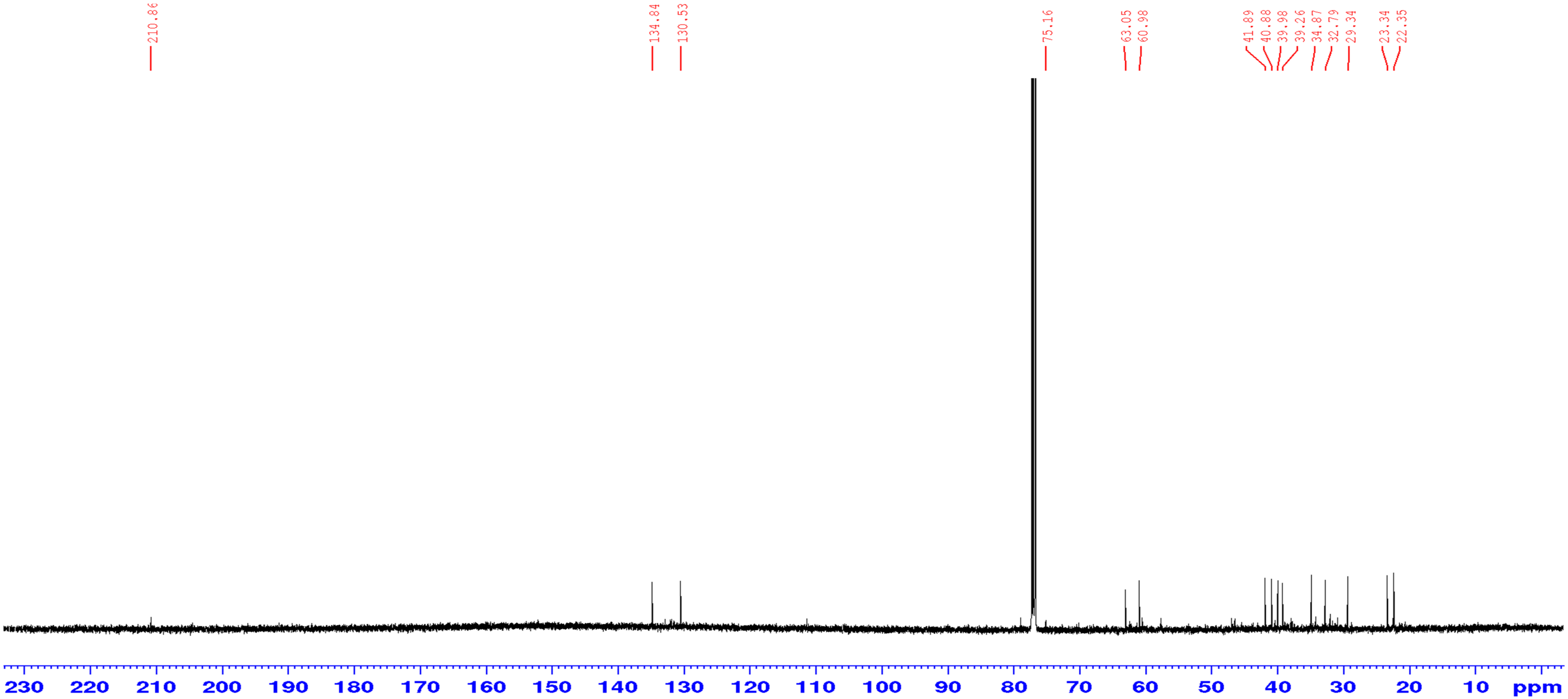

Figure S30. DEPT-135 spectrum (176.04 MHz) of **4** in $CDCl_3$

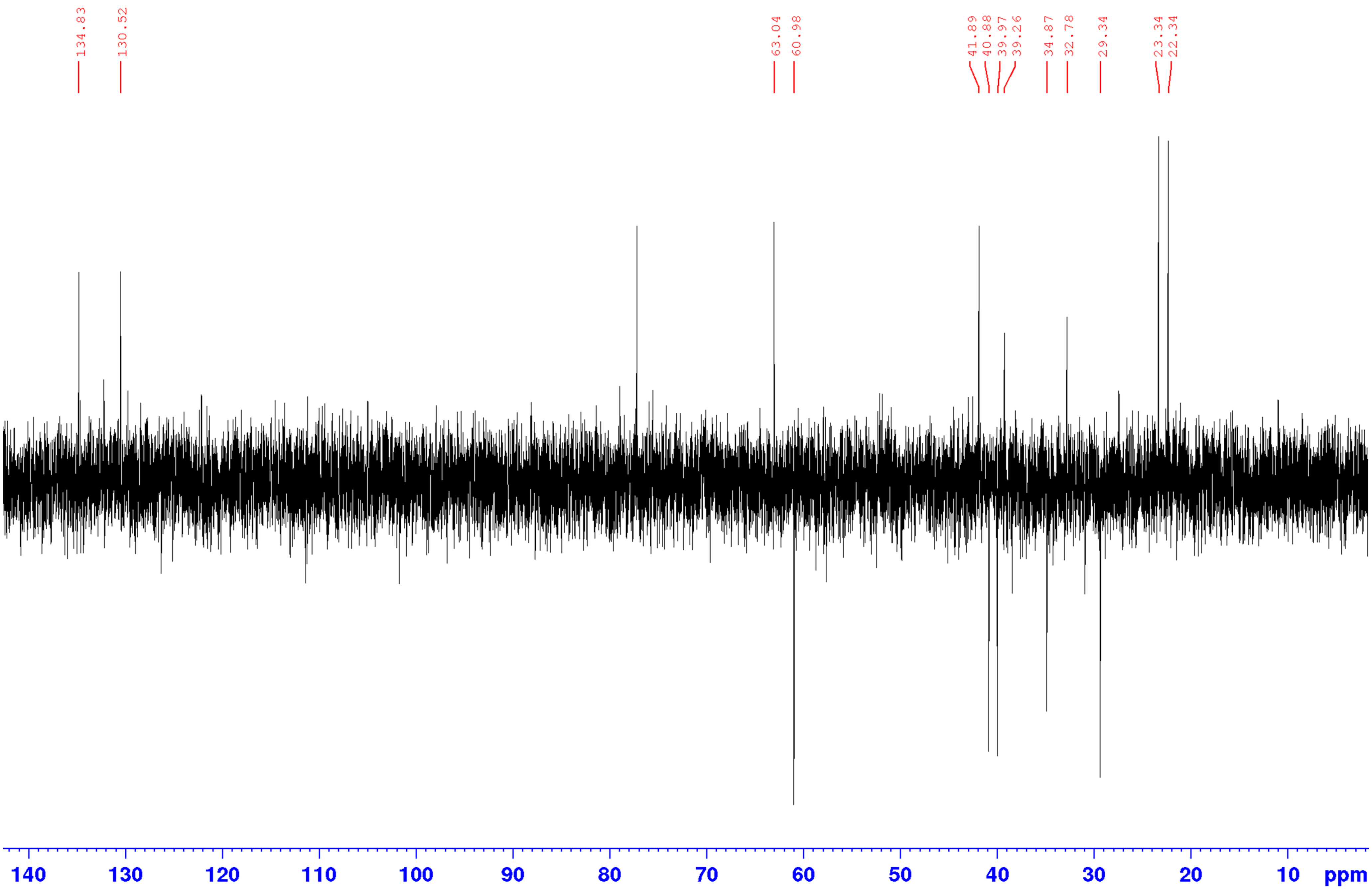

Figure S31. DEPT-90 spectrum (176.04 MHz) of **4** in $CDCl_3$

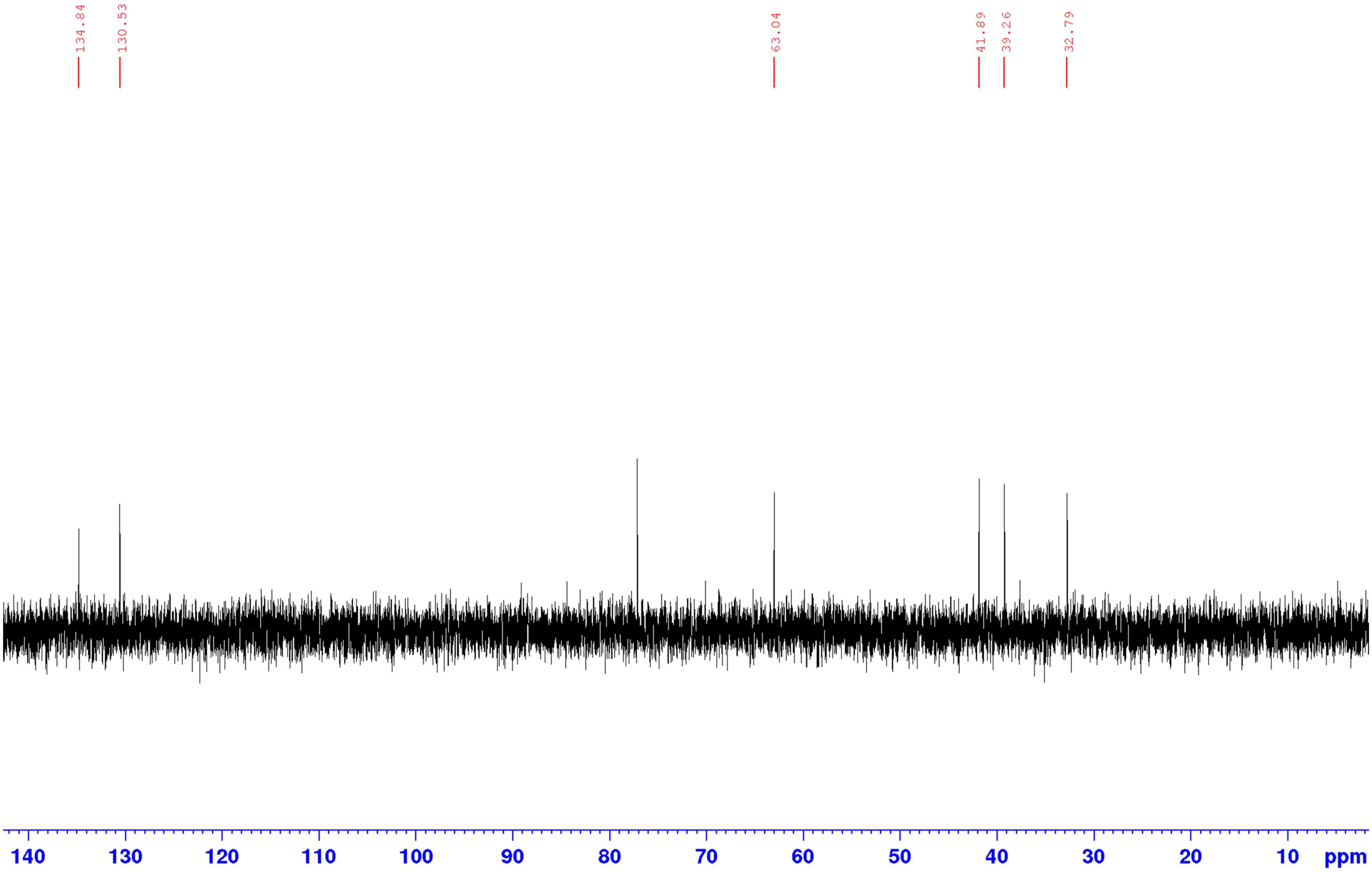

Figure S32. HSQC spectrum of **4** in $CDCl_3$

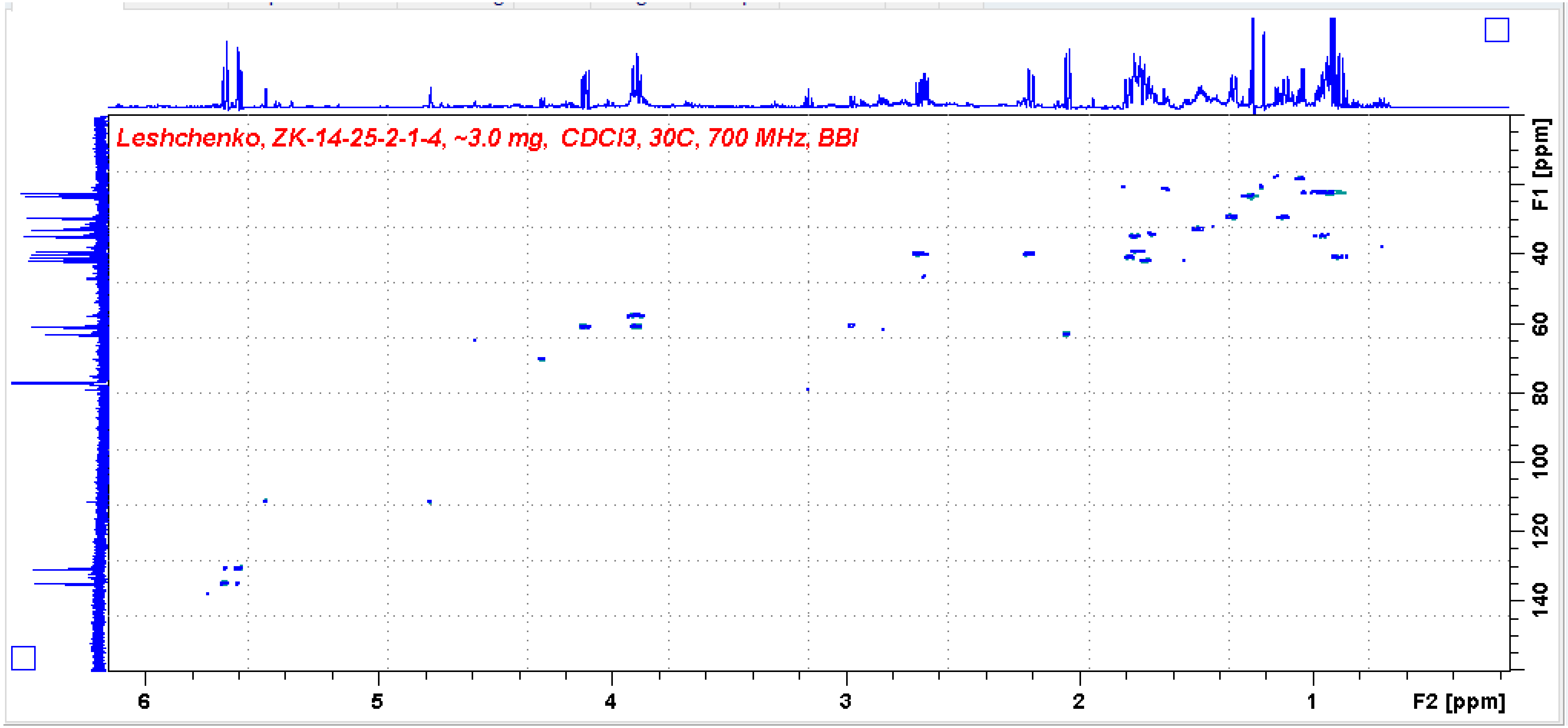

Figure S33. HMBC spectrum of **4** in $CDCl_3$

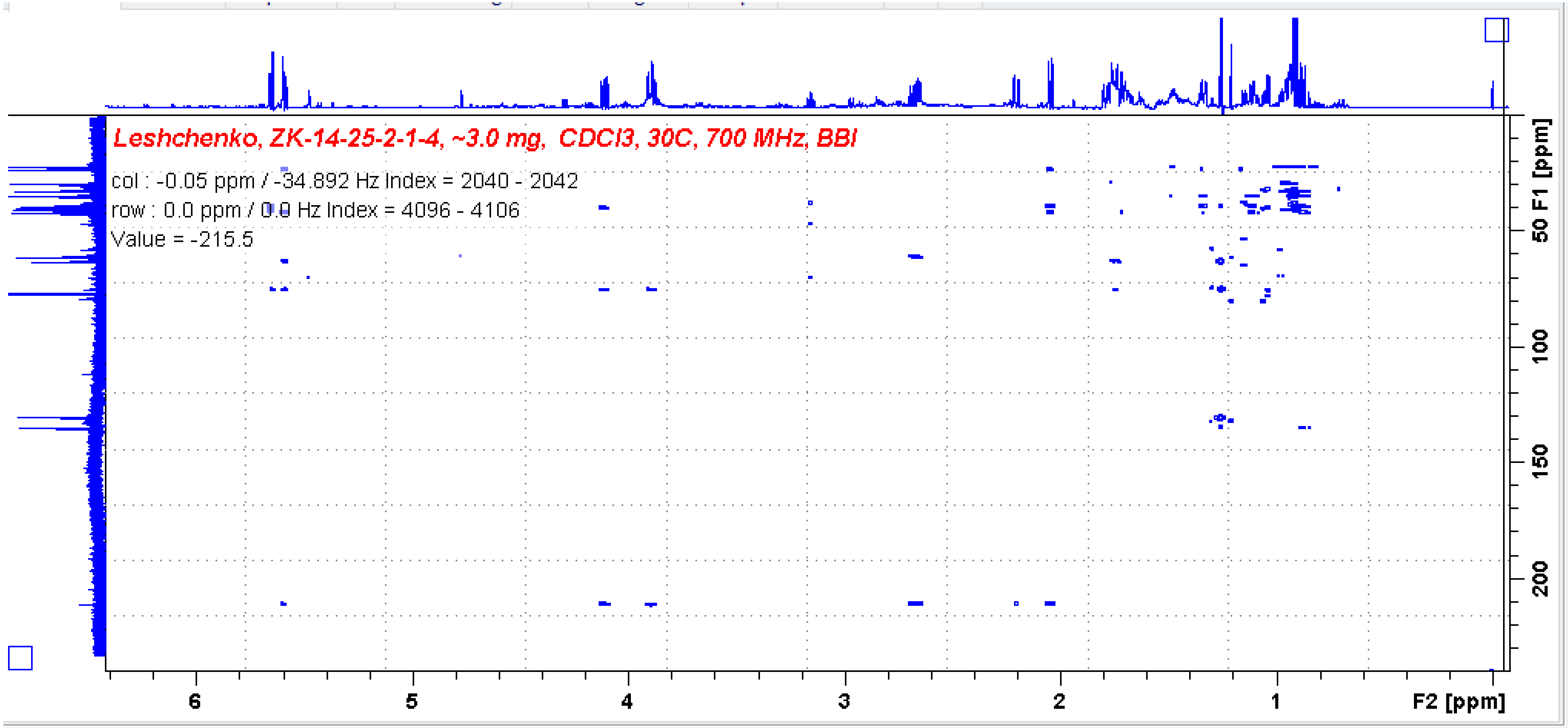

Figure S34. 1H-1H COSY spectrum of **4** in $CDCl_3$

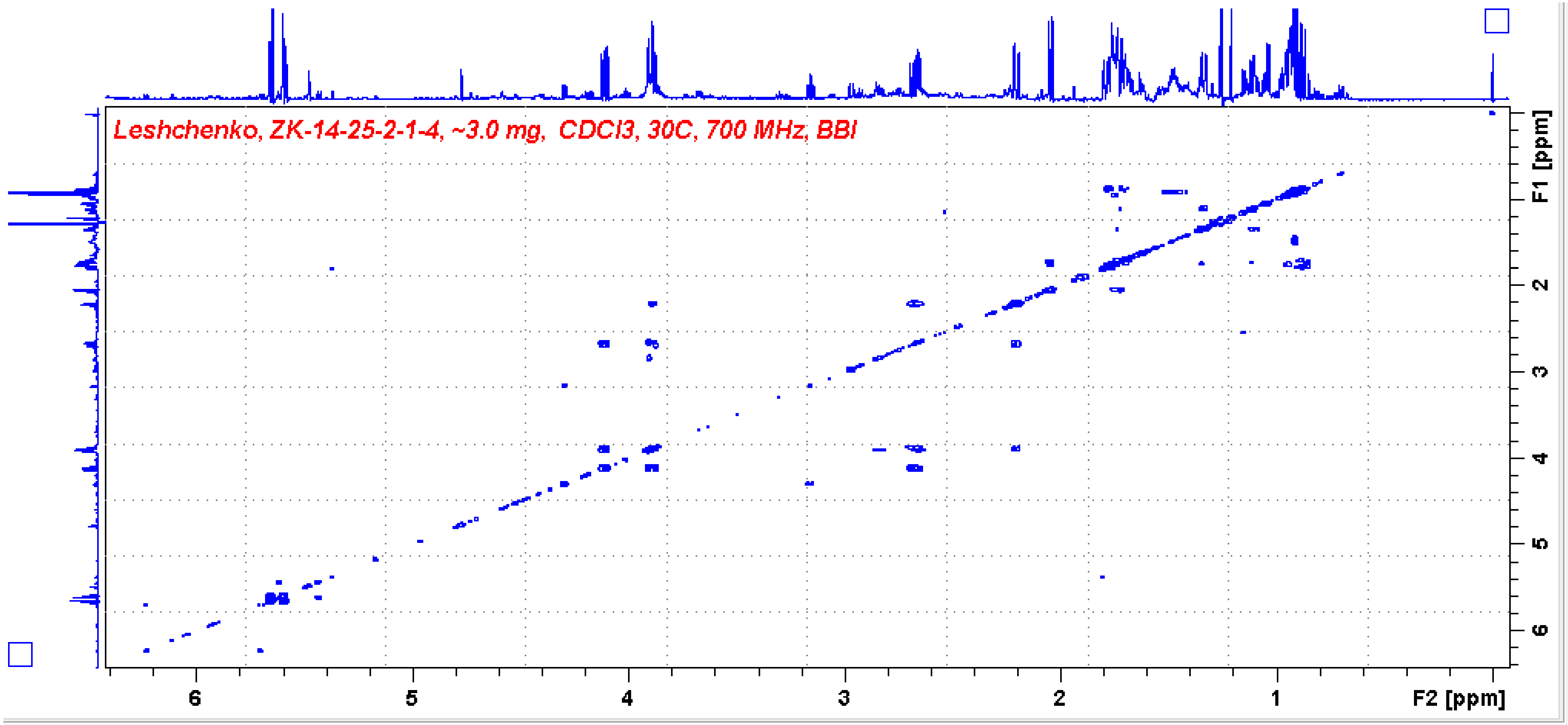

Figure S35. NOESY spectrum of **4** in $CDCl_3$

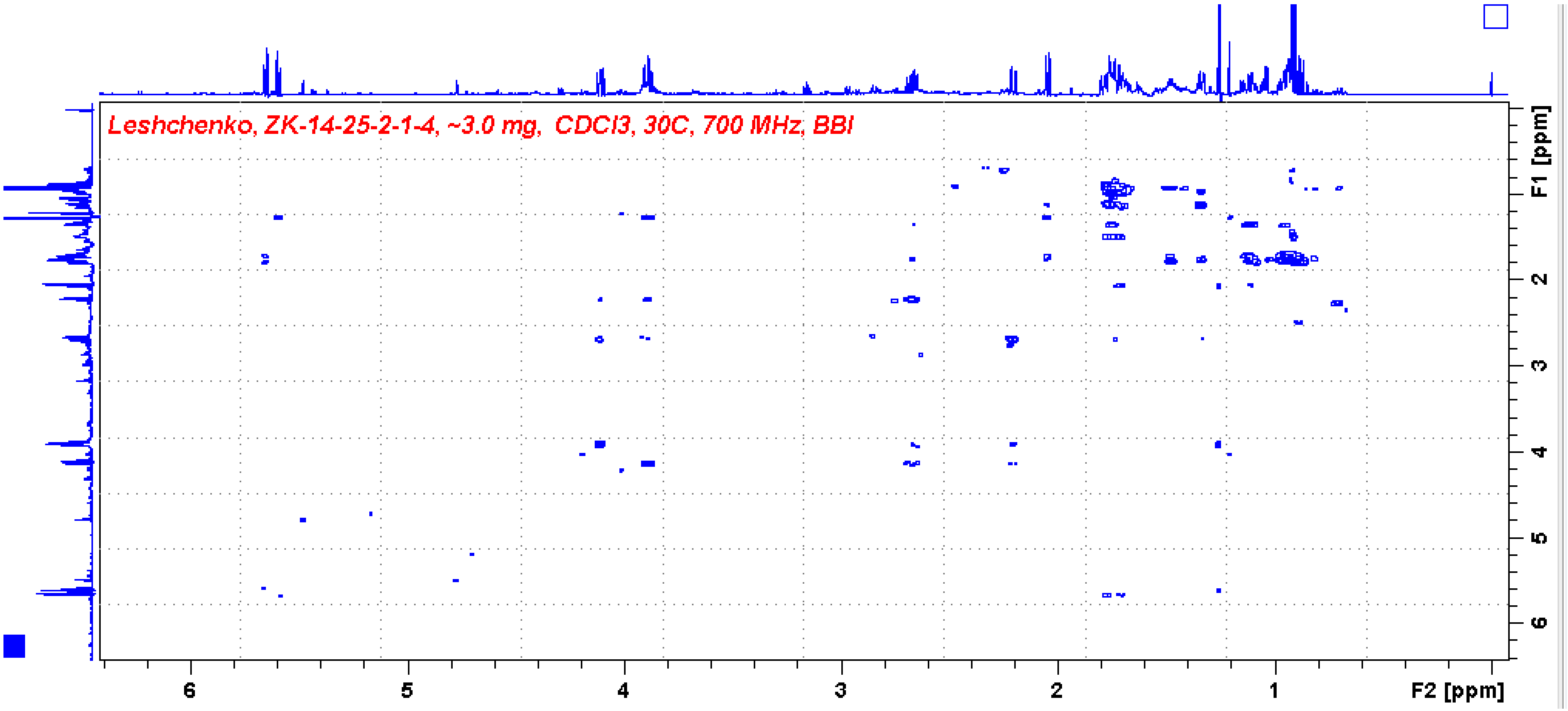

Figure S36. ^{1}H NMR spectrum (700.13 MHz) of **5** in $CDCl_3$

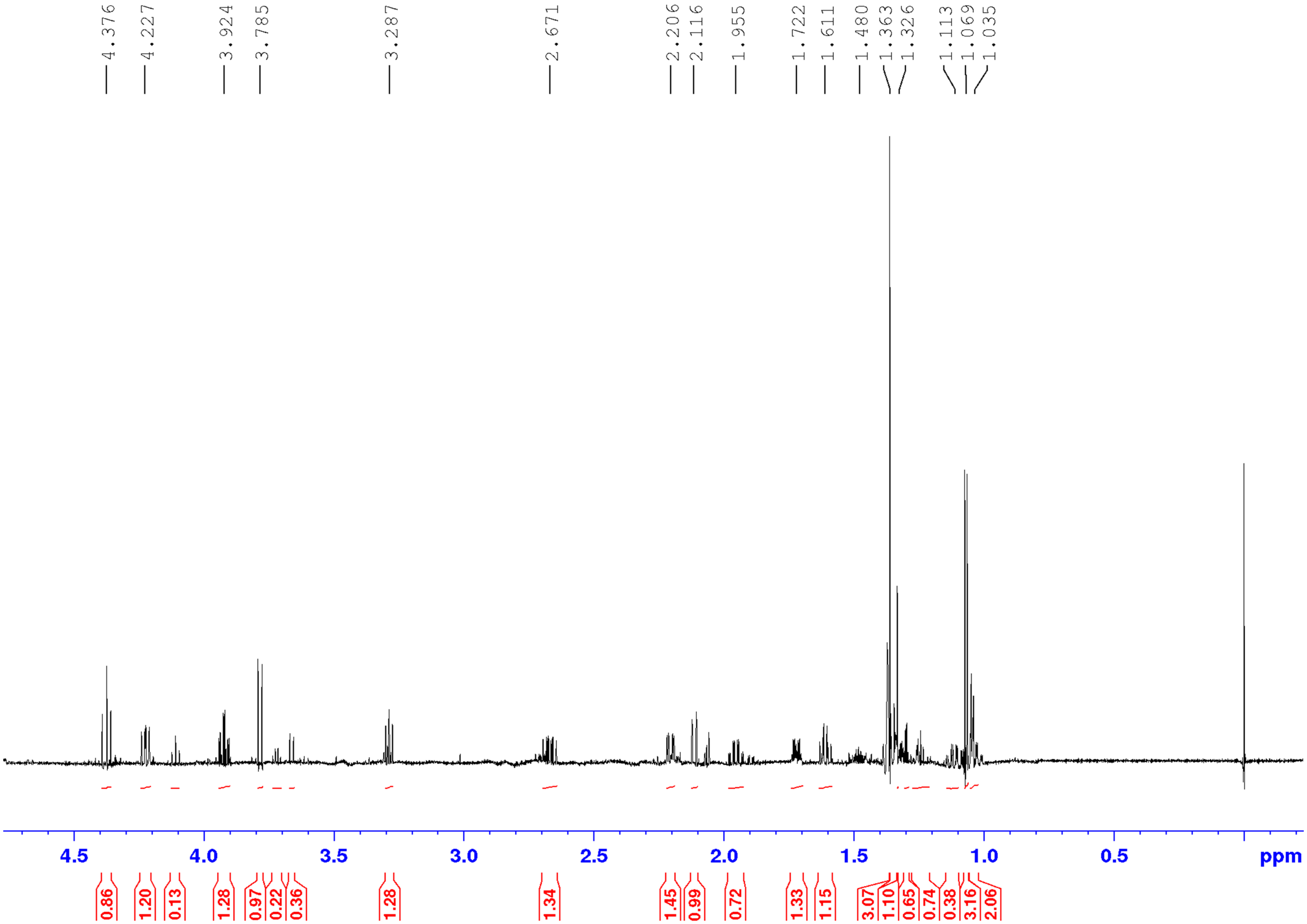

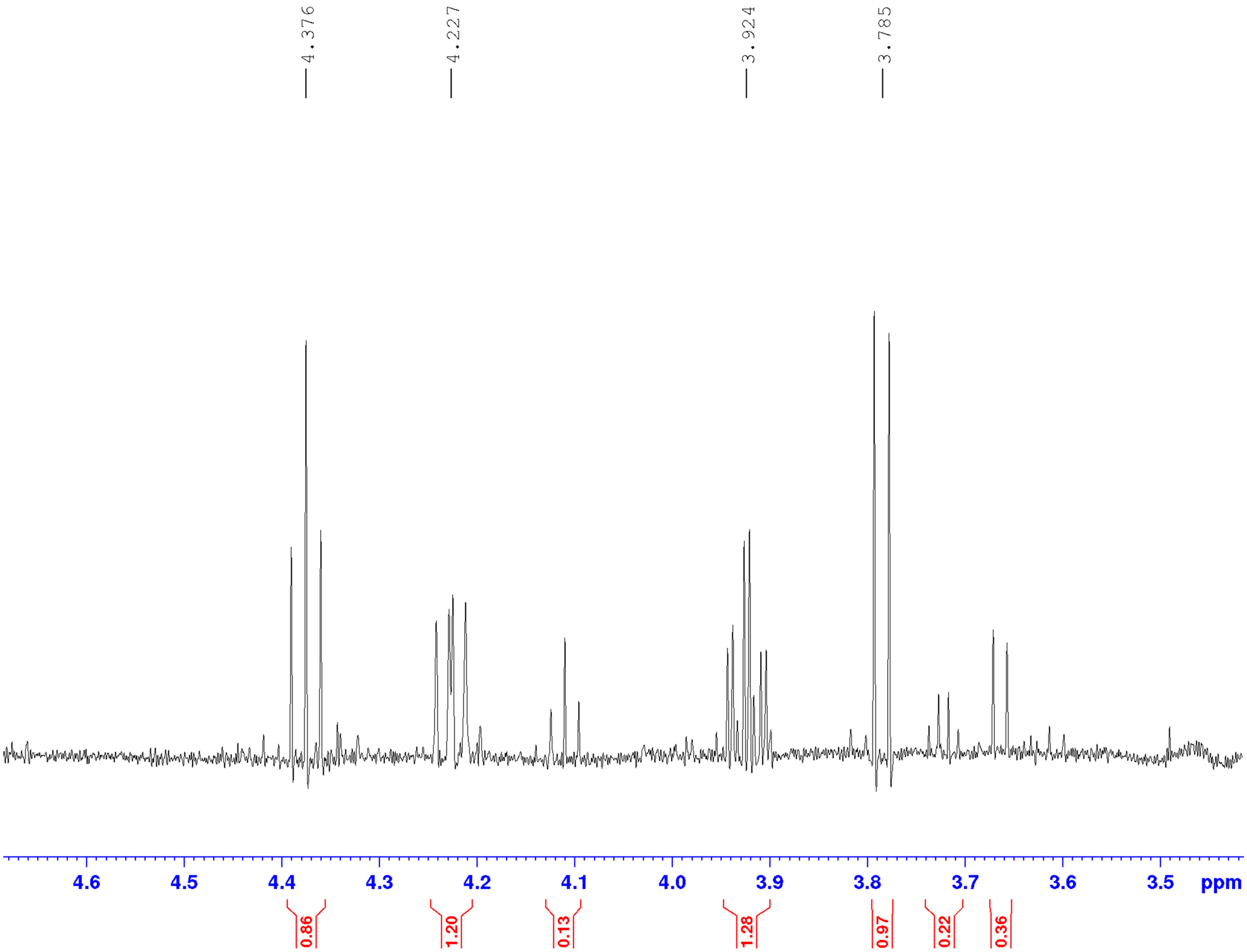
4.376
4.227
3.924
3.785
4.6
4.5
4.4
4.3
4.2
4.1
4.0
3.9
3.8
3.7
3.6
3.5
ppm
0.86
1.20
0.13
1.28
0.97
0.22
0.36

Figure S37. ^{13}C NMR spectrum (176.04 MHz) of **5** in $CDCl_3$

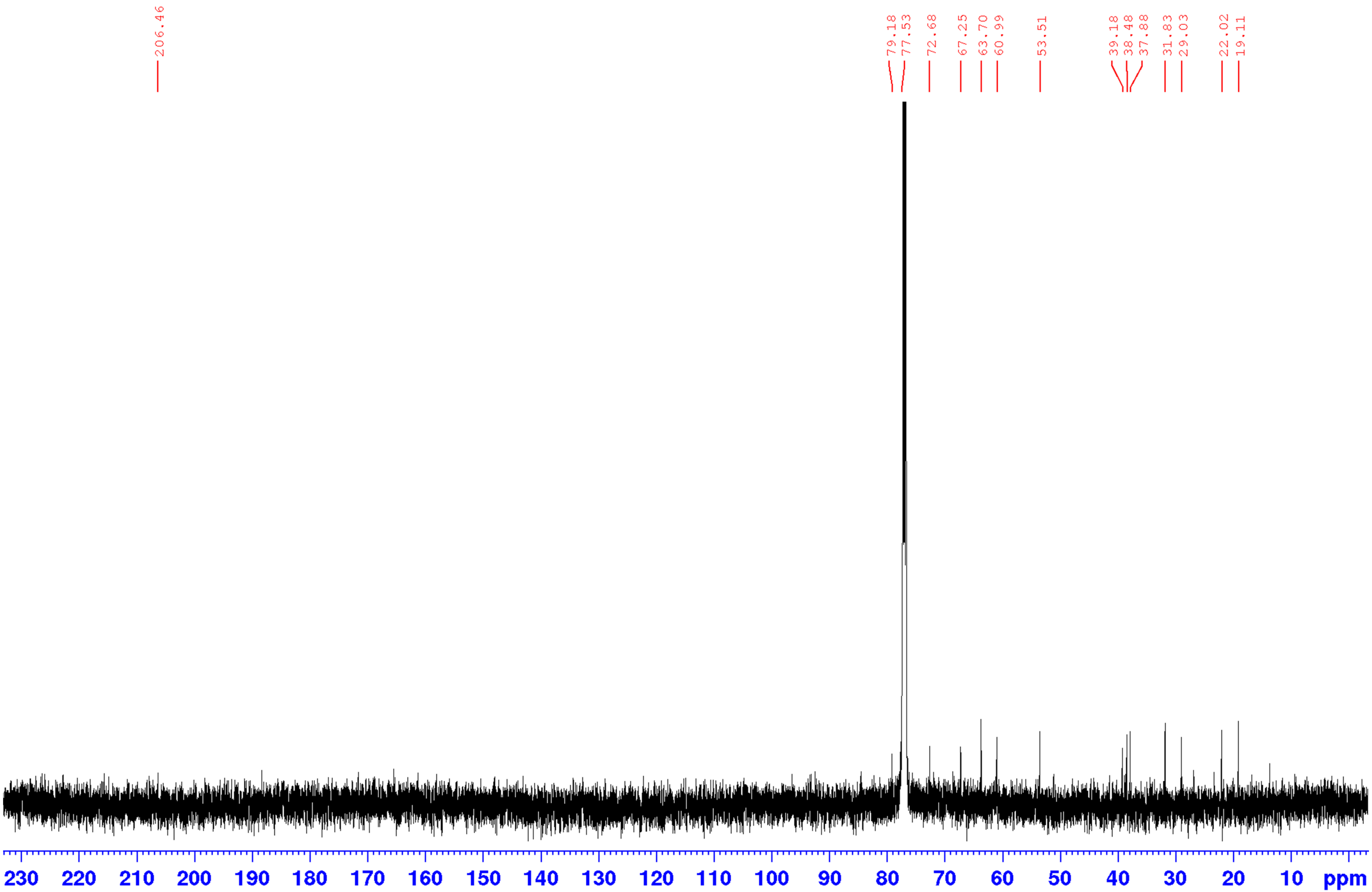

Figure S38. DEPT-135 spectrum (176.04 MHz) of **5** in $CDCl_3$

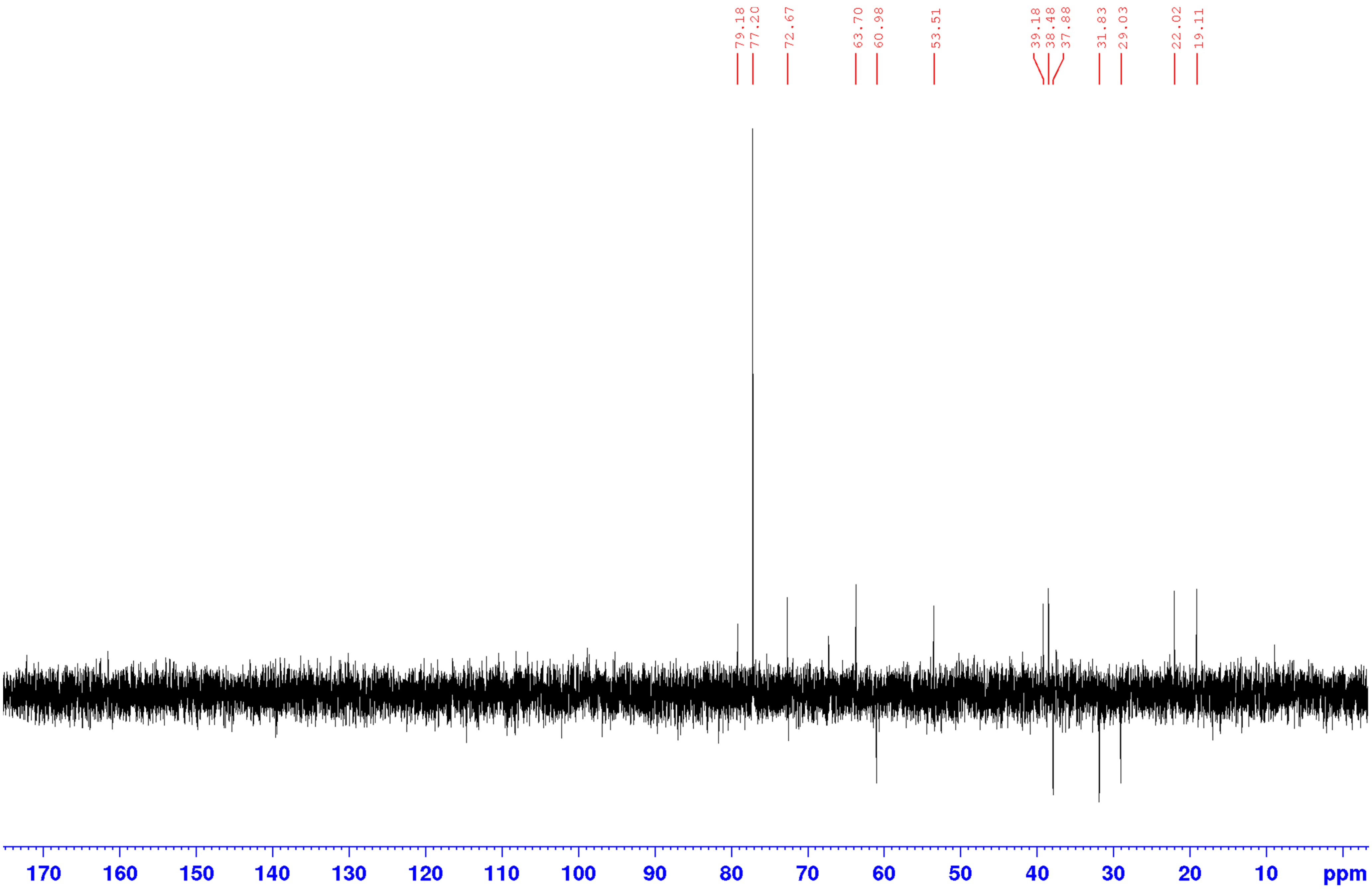

Figure S39. HSQC spectrum of **5** in $CDCl_3$

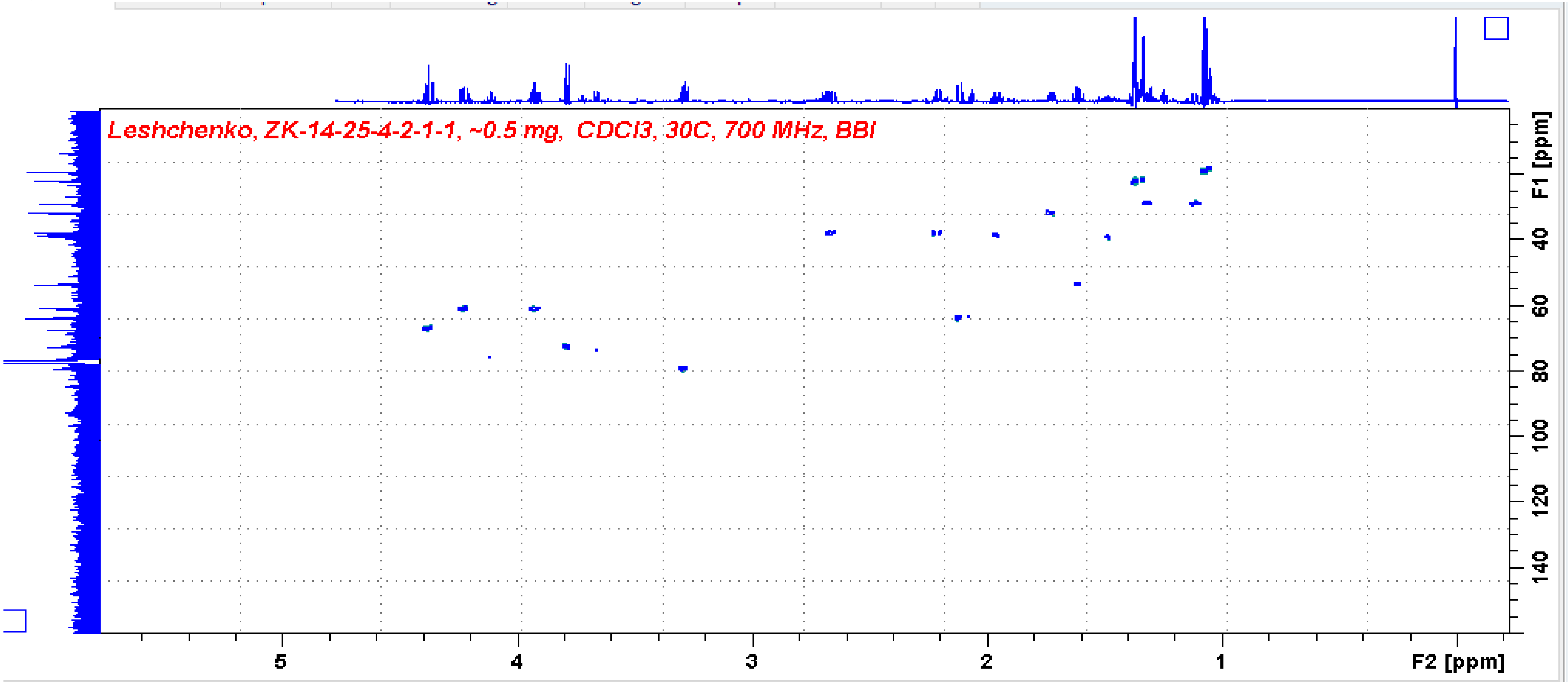

Figure S40. HMBC spectrum of **5** in $CDCl_3$

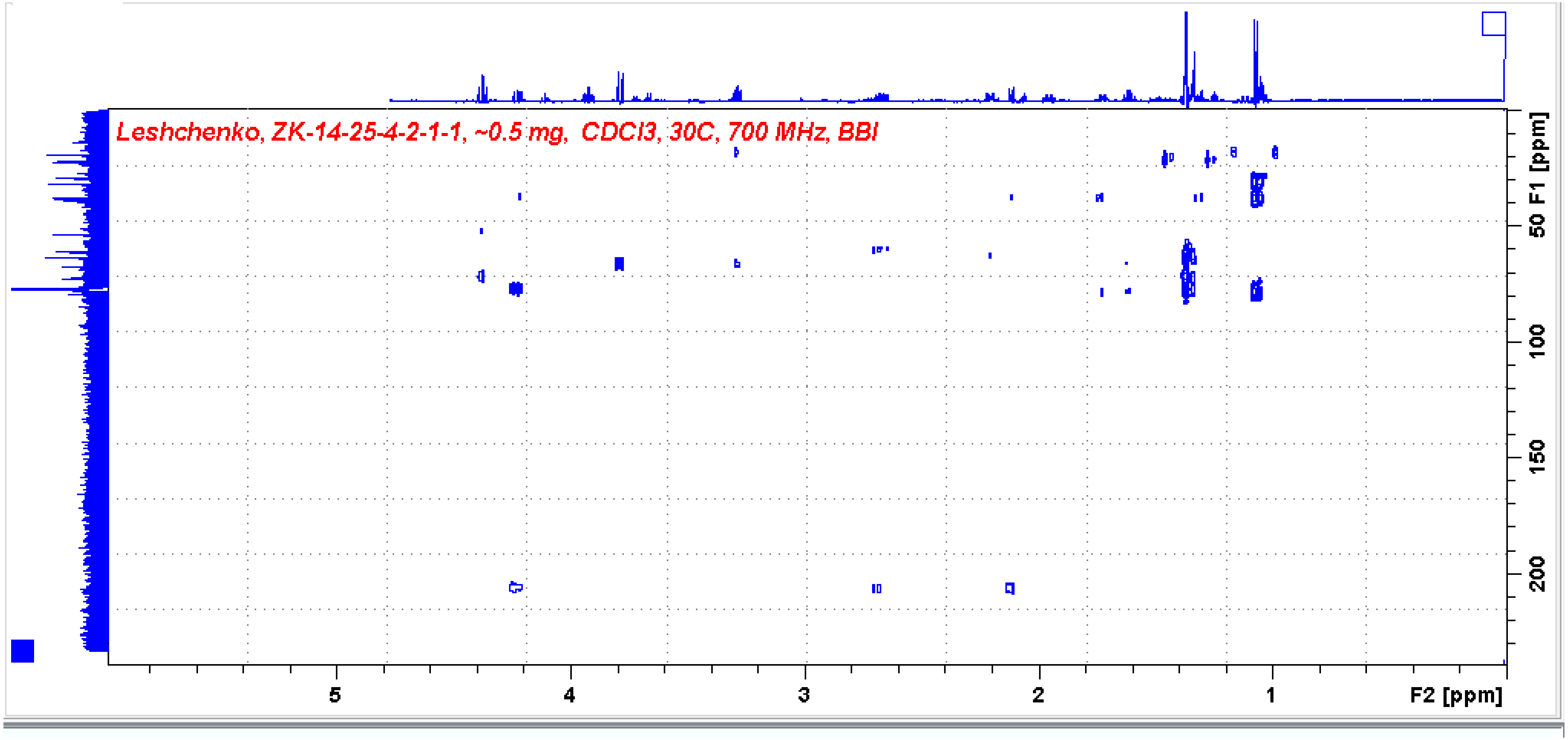

Figure S41. 1H-1H COSY spectrum of **5** in $CDCl_3$

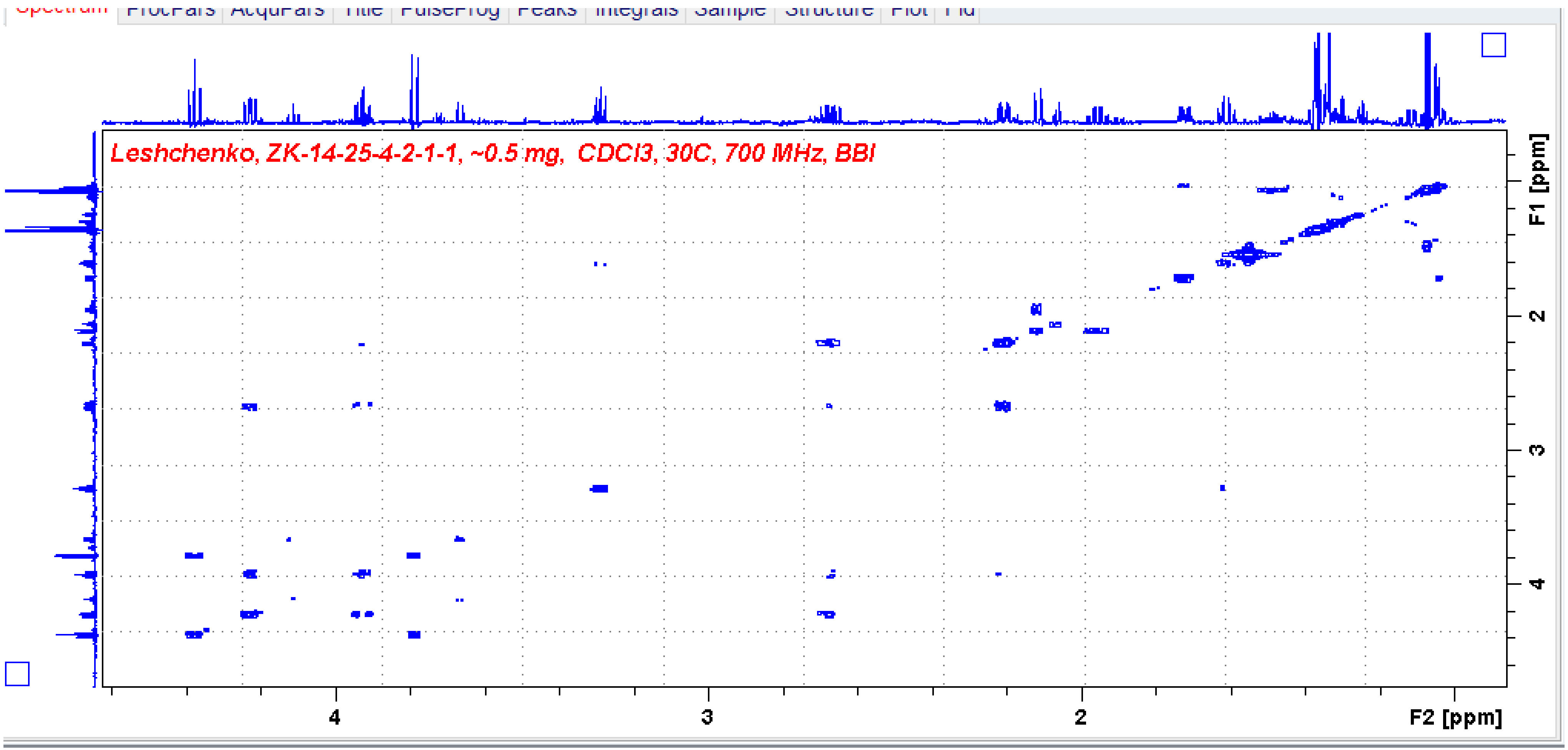

Figure S42. NOESY spectrum of **5** in CDCl3

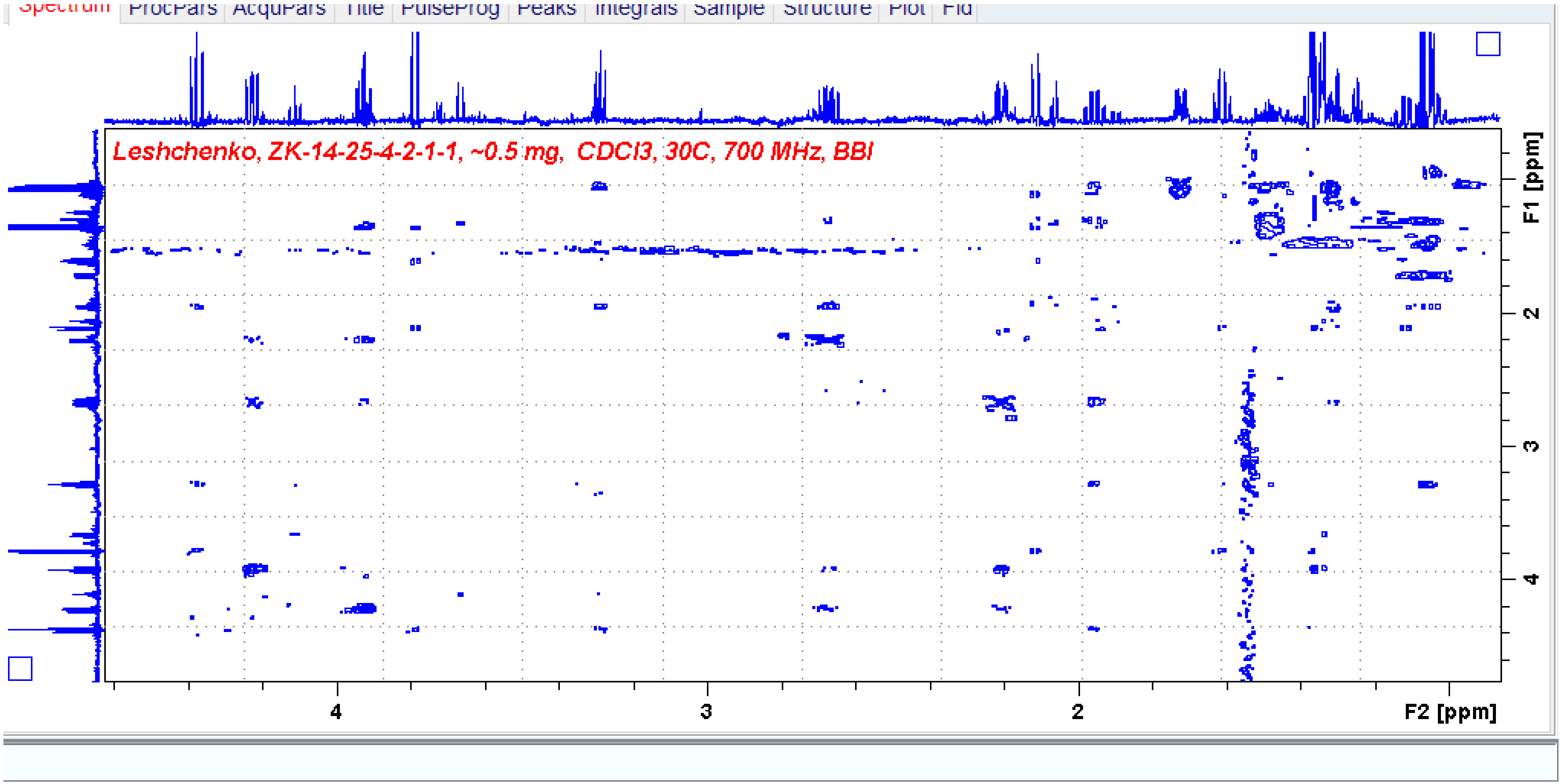

Figure S43. ^{1}H NMR spectrum (500.13 MHz) of **6** in $CDCl_3$

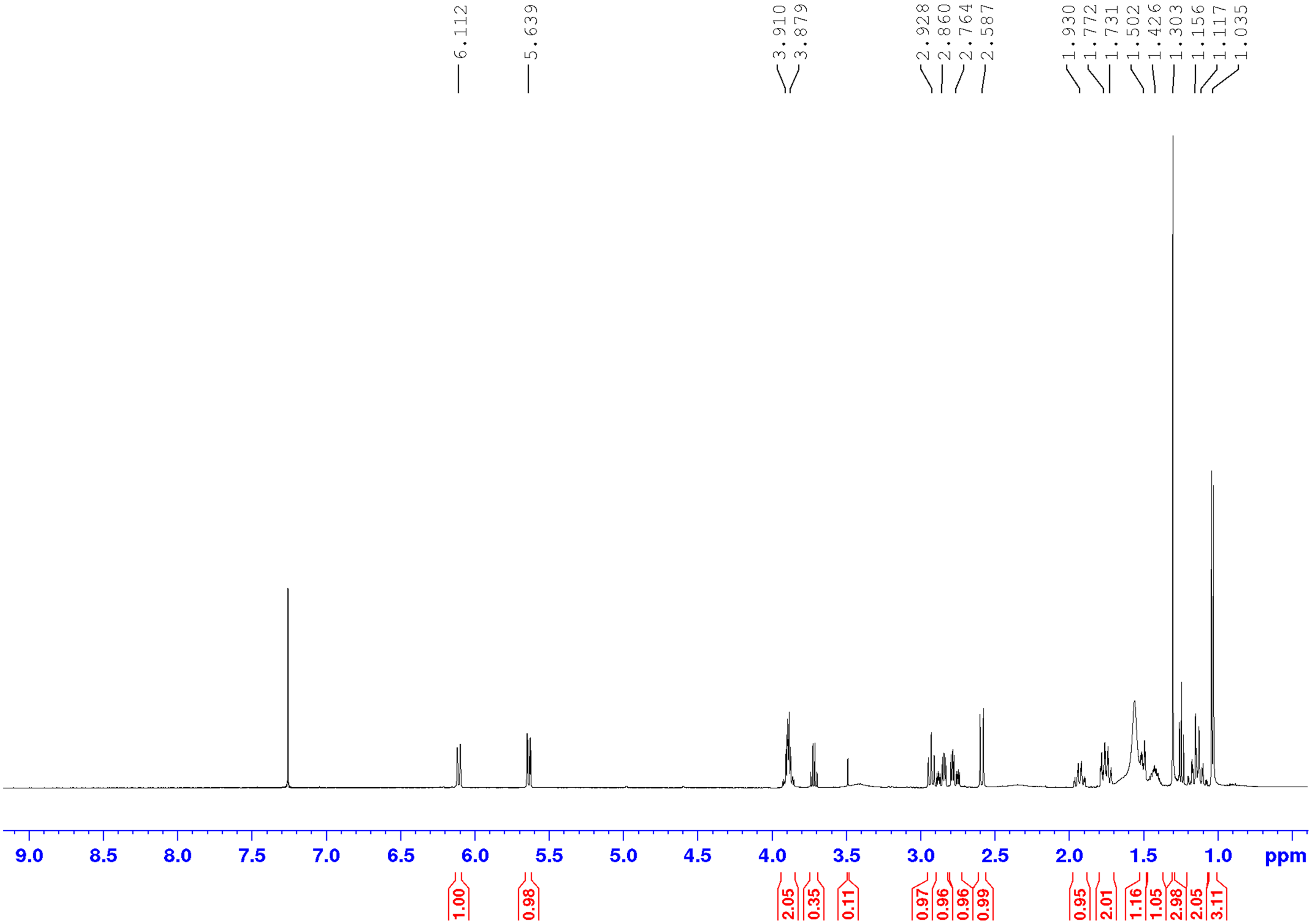

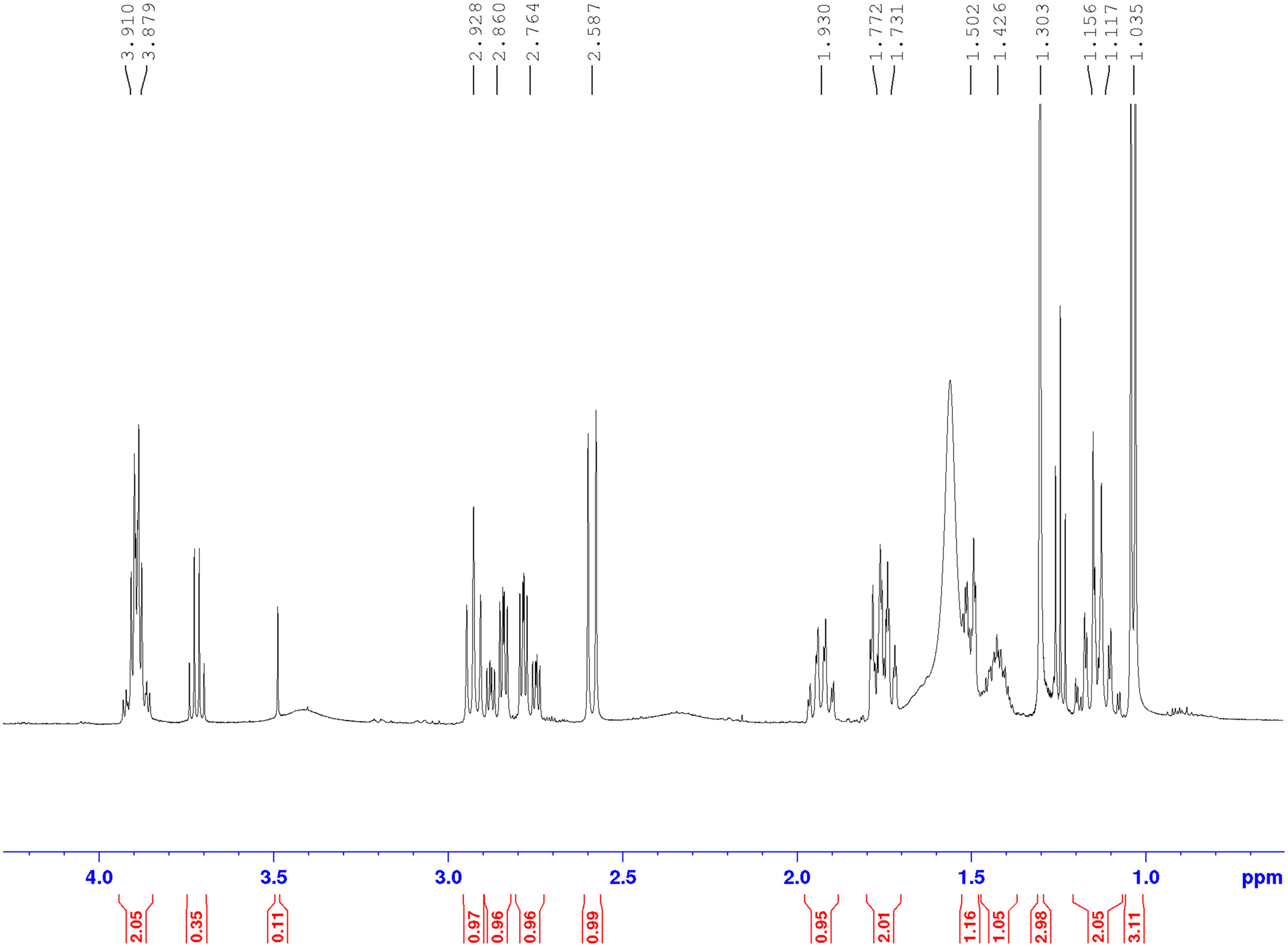

3.910
3.879
2.928
2.860
2.764
2.587
1.930
1.772
1.731
1.502
1.426
1.303
1.156
1.117
1.035
4.0
3.5
3.0
2.5
2.0
1.5
1.0
ppm
2.05
0.35
0.11
0.97
0.96
0.96
0.99
0.95
2.01
1.16
1.05
2.98
2.05
3.11

Figure S44. ^{13}C NMR spectrum (125.77 MHz) of **6** in $CDCl_3$

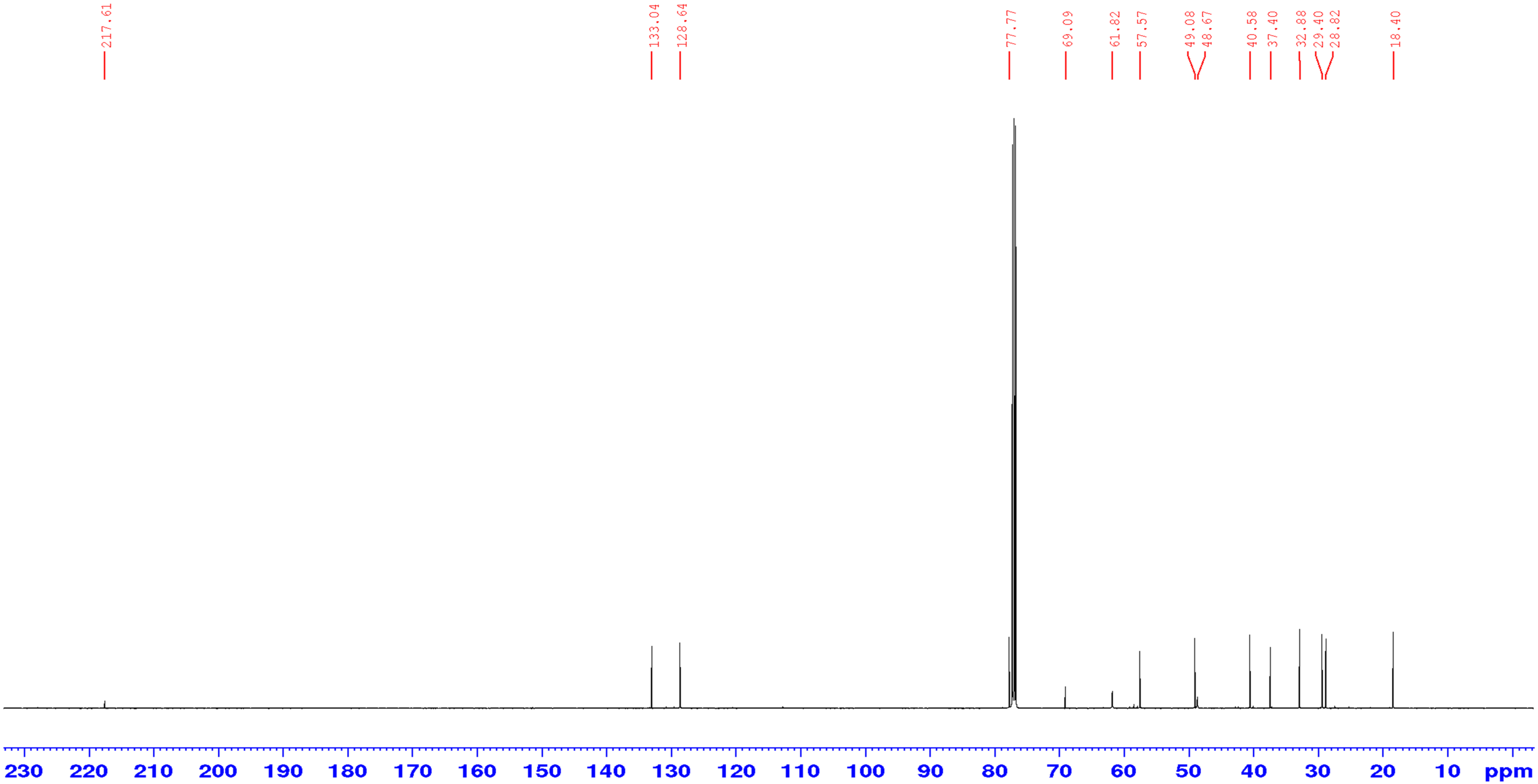

Figure S45. DEPT-135 spectrum (125.77 MHz) of **6** in $CDCl_3$

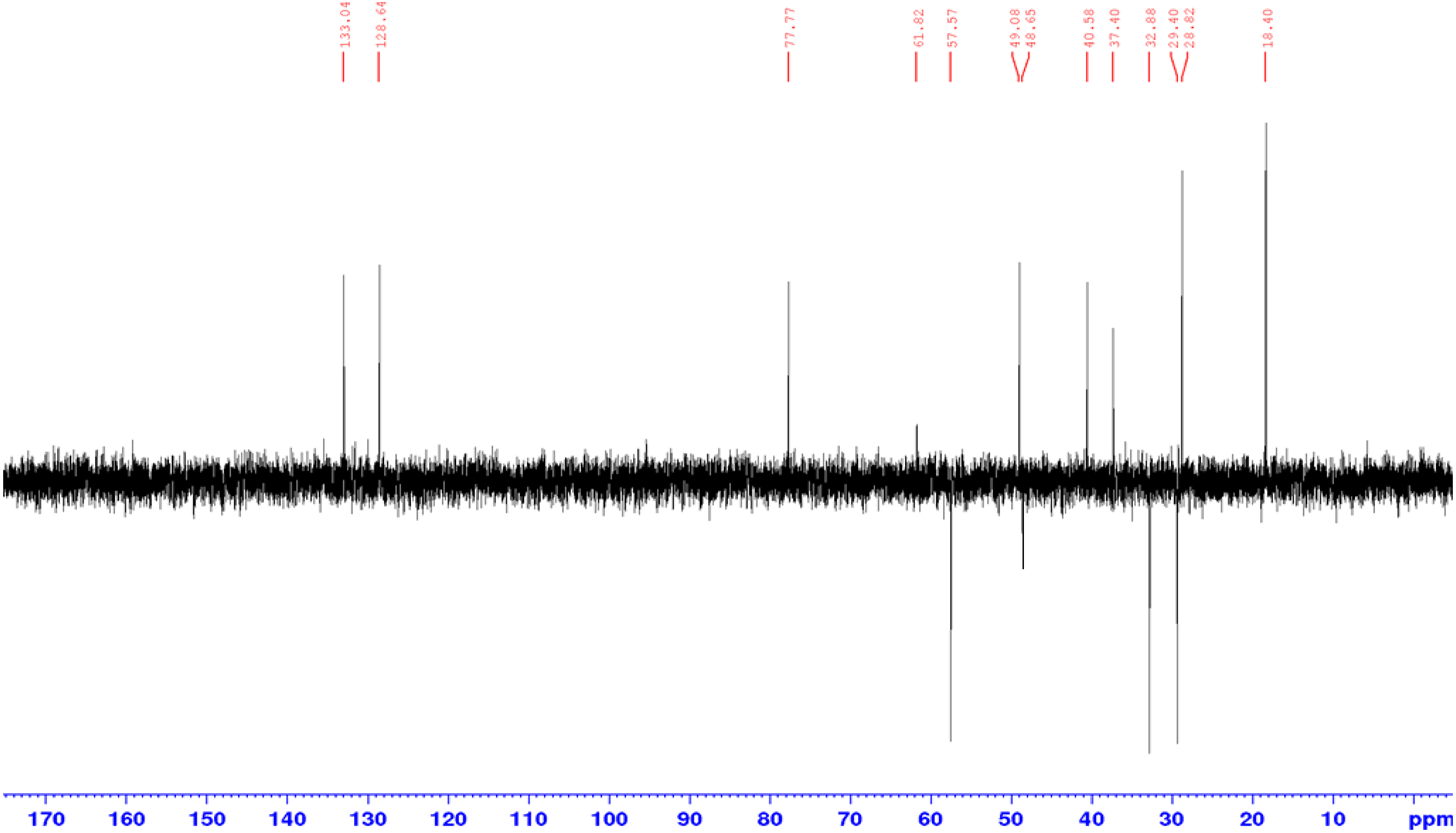

Figure S46. DEPT-90 spectrum (125.77 MHz) of **6** in $CDCl_3$

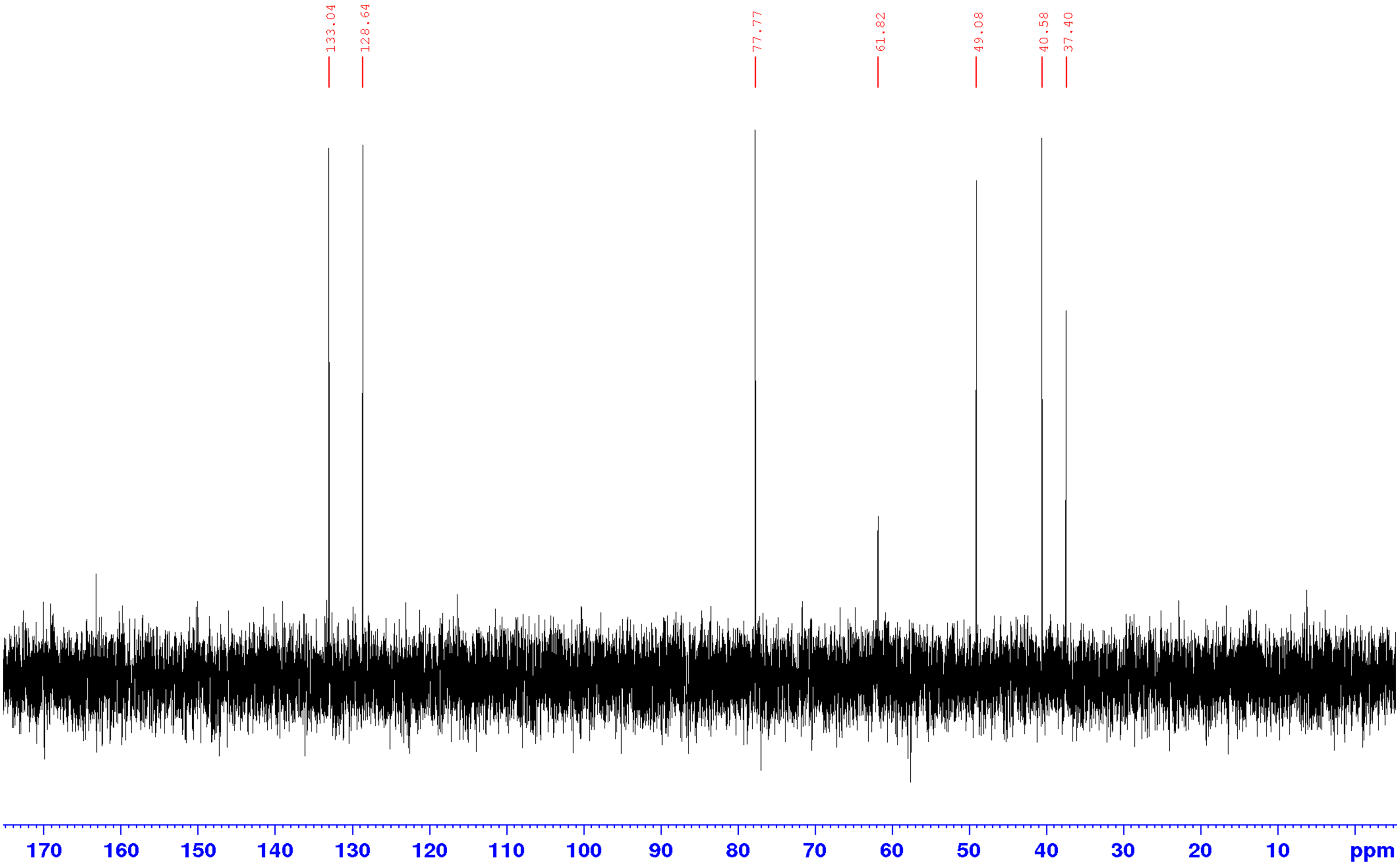

Figure S47. HSQC spectrum of **6** in $CDCl_3$

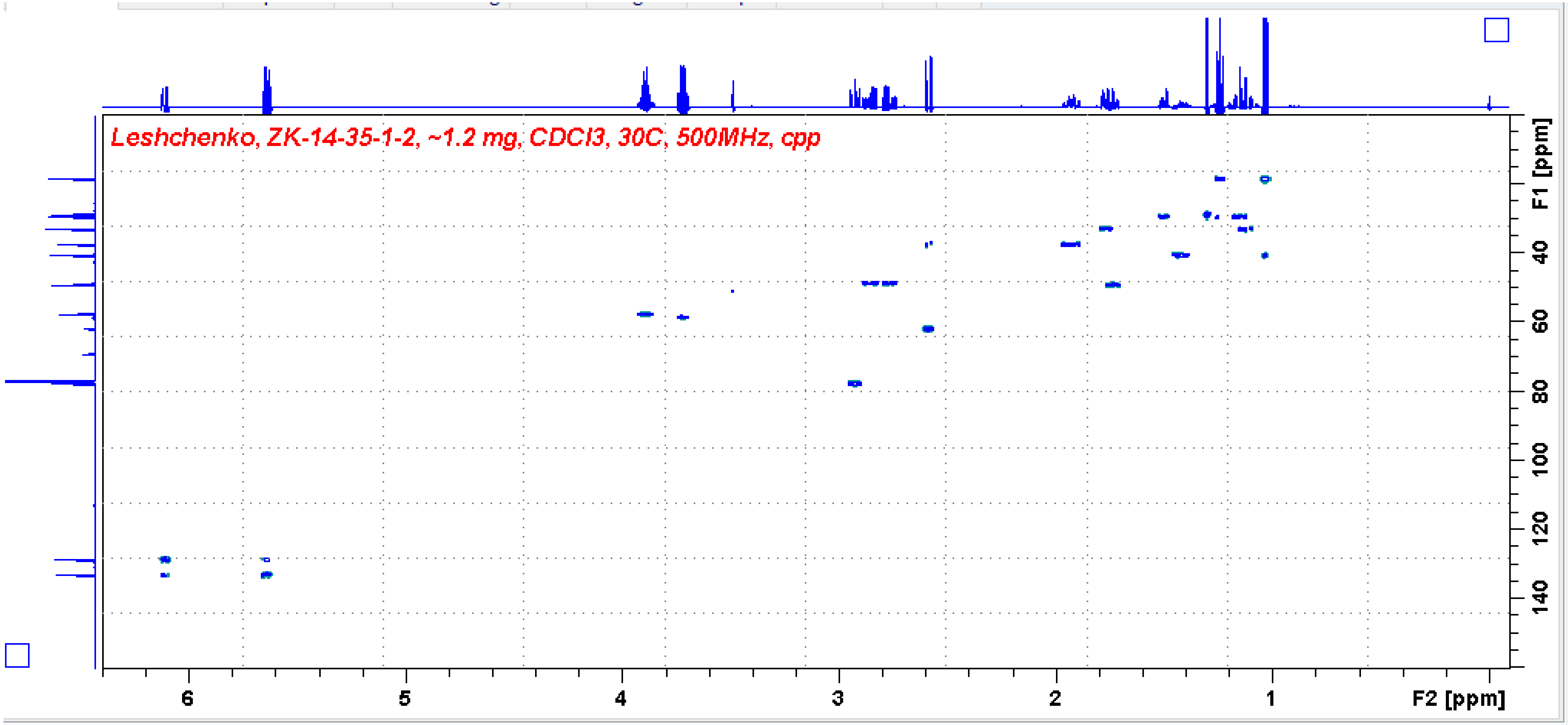

Figure S48. HMBC spectrum of **6** in $CDCl_3$

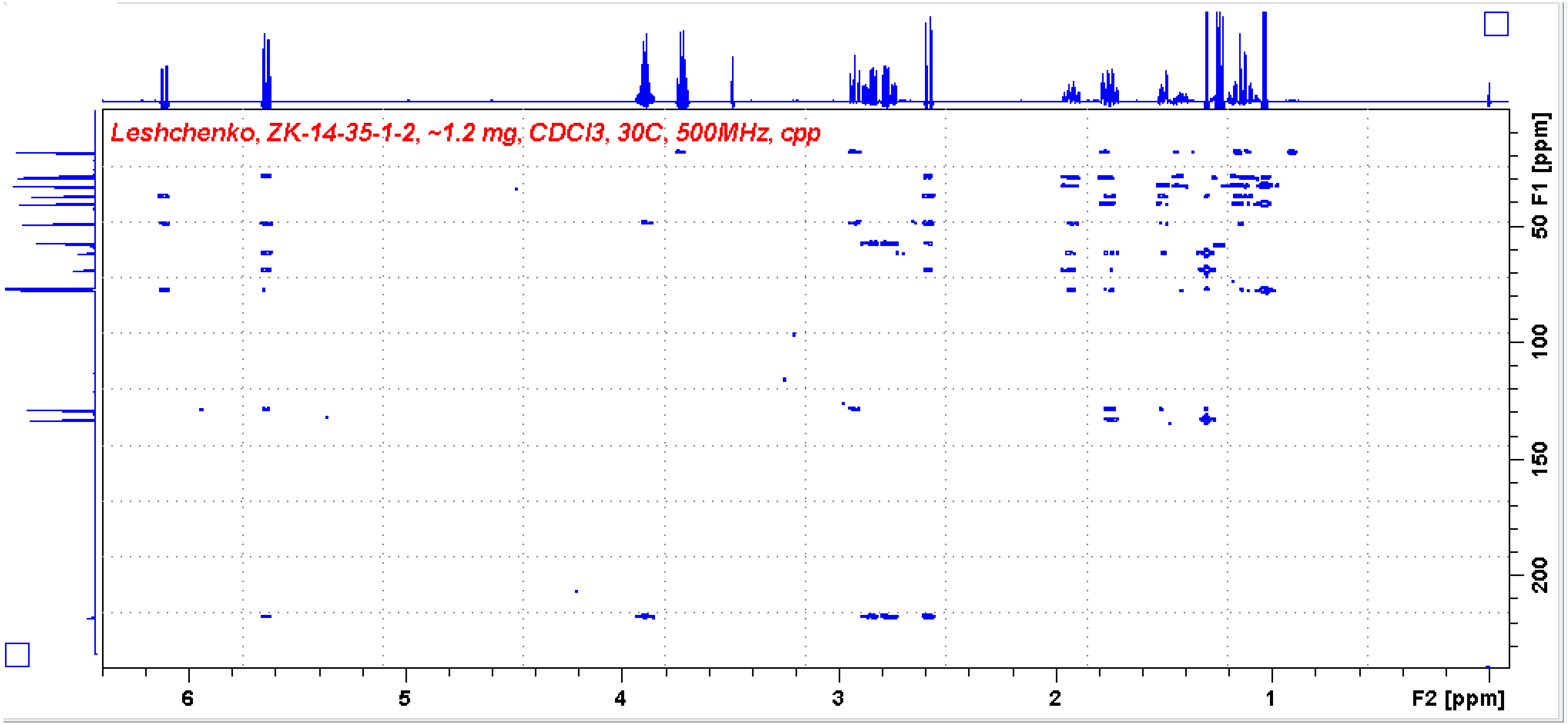

Figure S49. 1H-1H COSY spectrum of **6** in $CDCl_3$

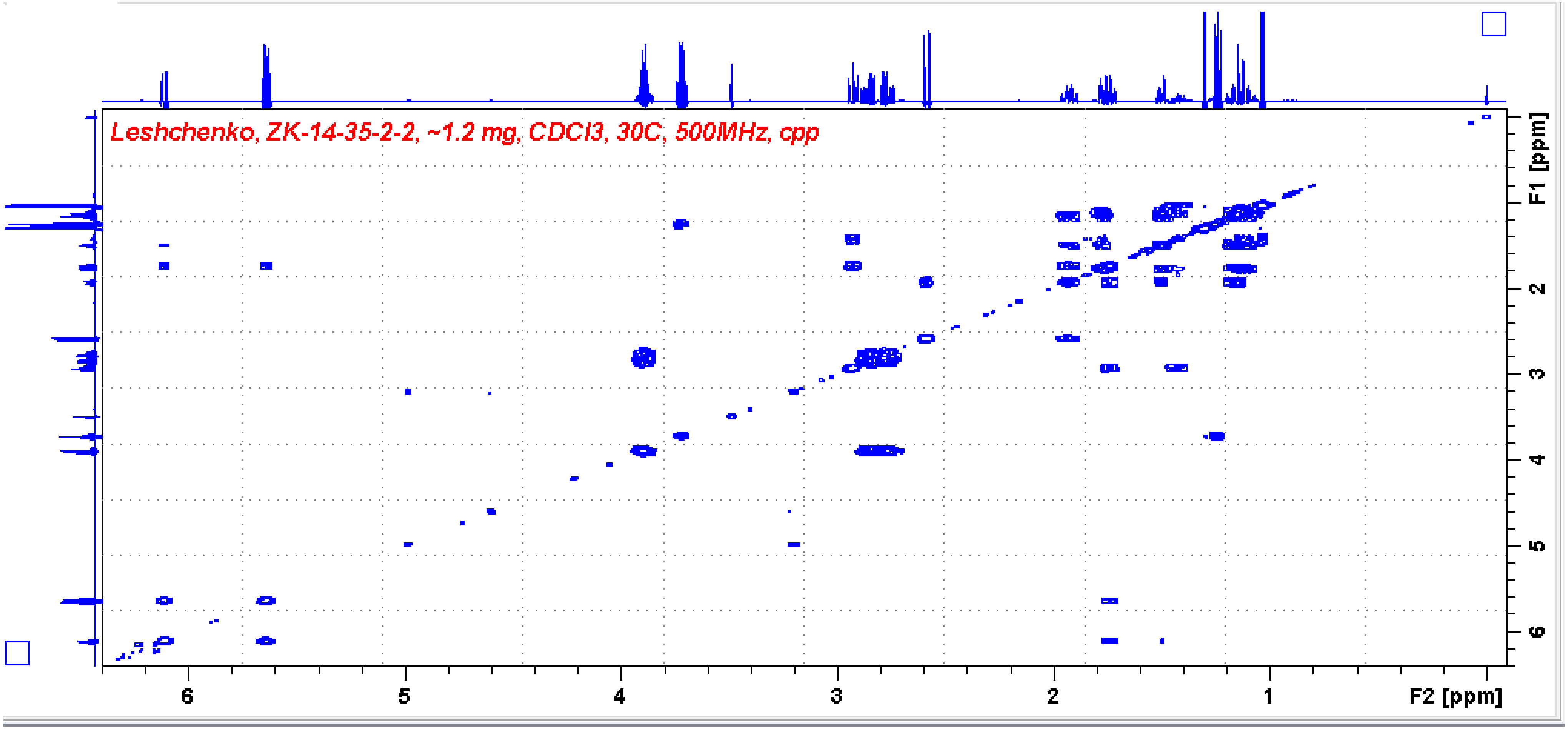

Figure S50. NOESY spectrum of **6** in $CDCl_3$

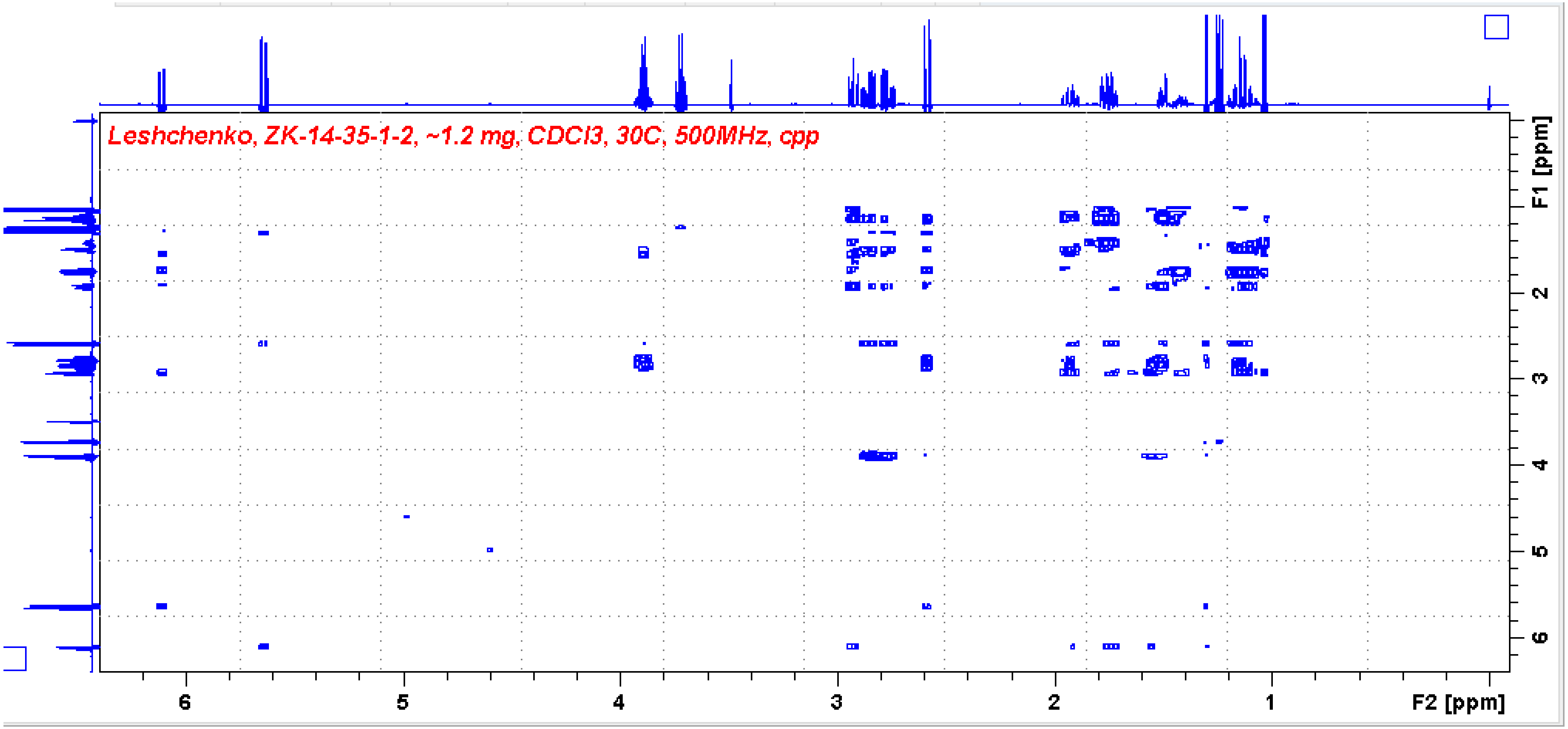

Figure S51. ^{1}H NMR spectrum (700.13 MHz) of **13** in DMSO-d6

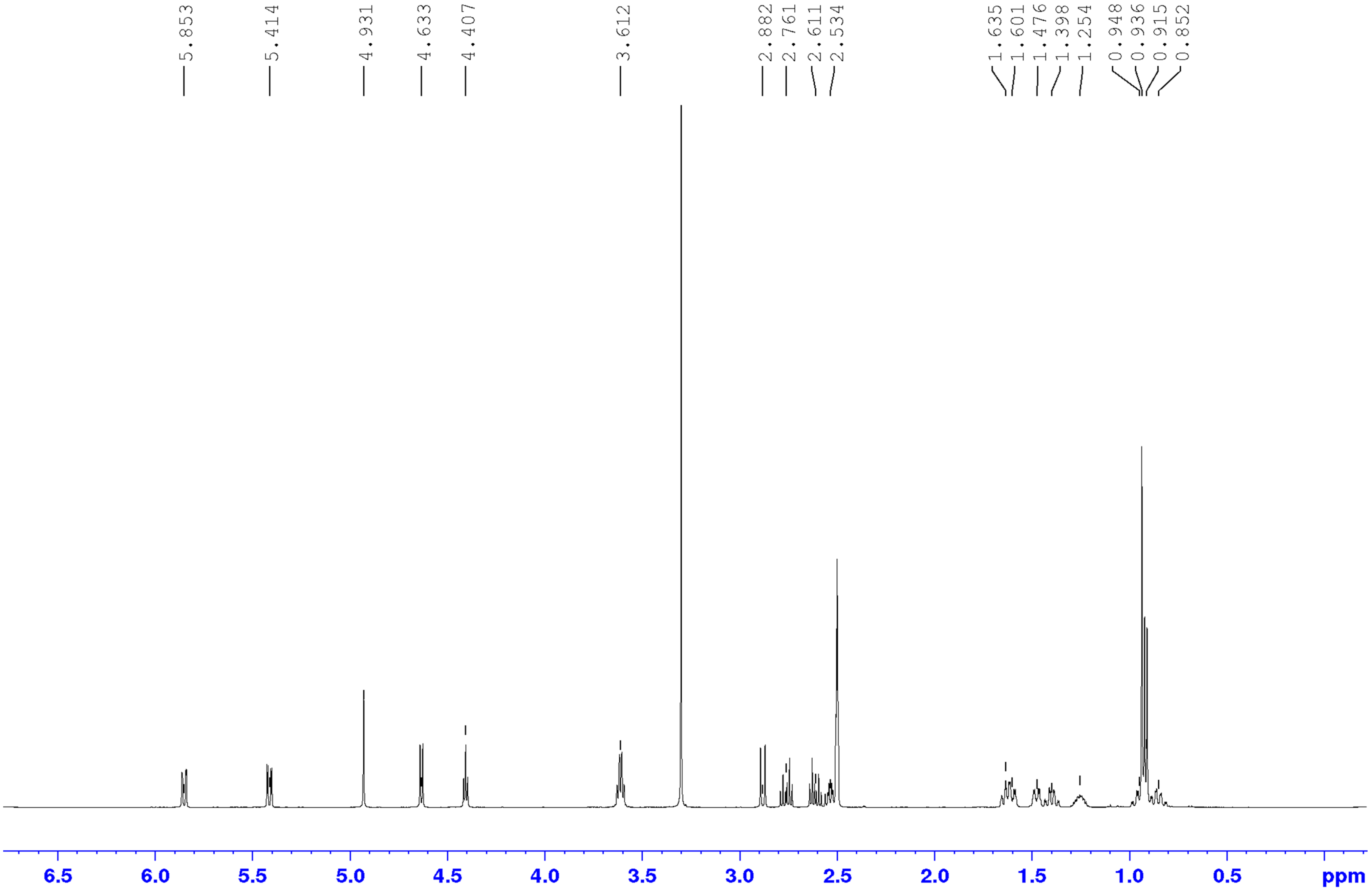

Figure S52. ^{13}C NMR spectrum (176.04 MHz) of **13** in DMSO-d6

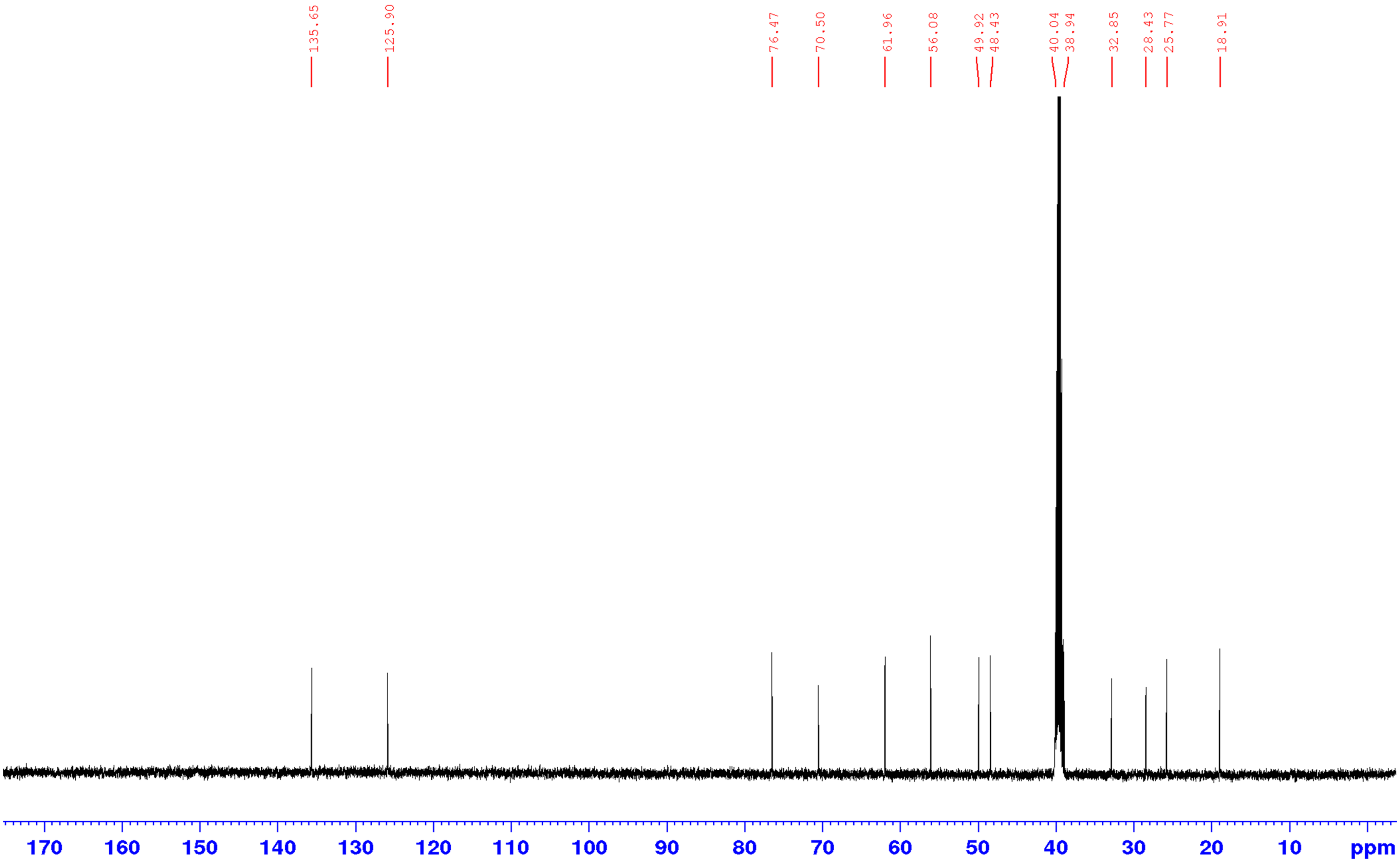

Figure S53. DEPT-135 spectrum (176.04 MHz) of **13** in DMSO-d6

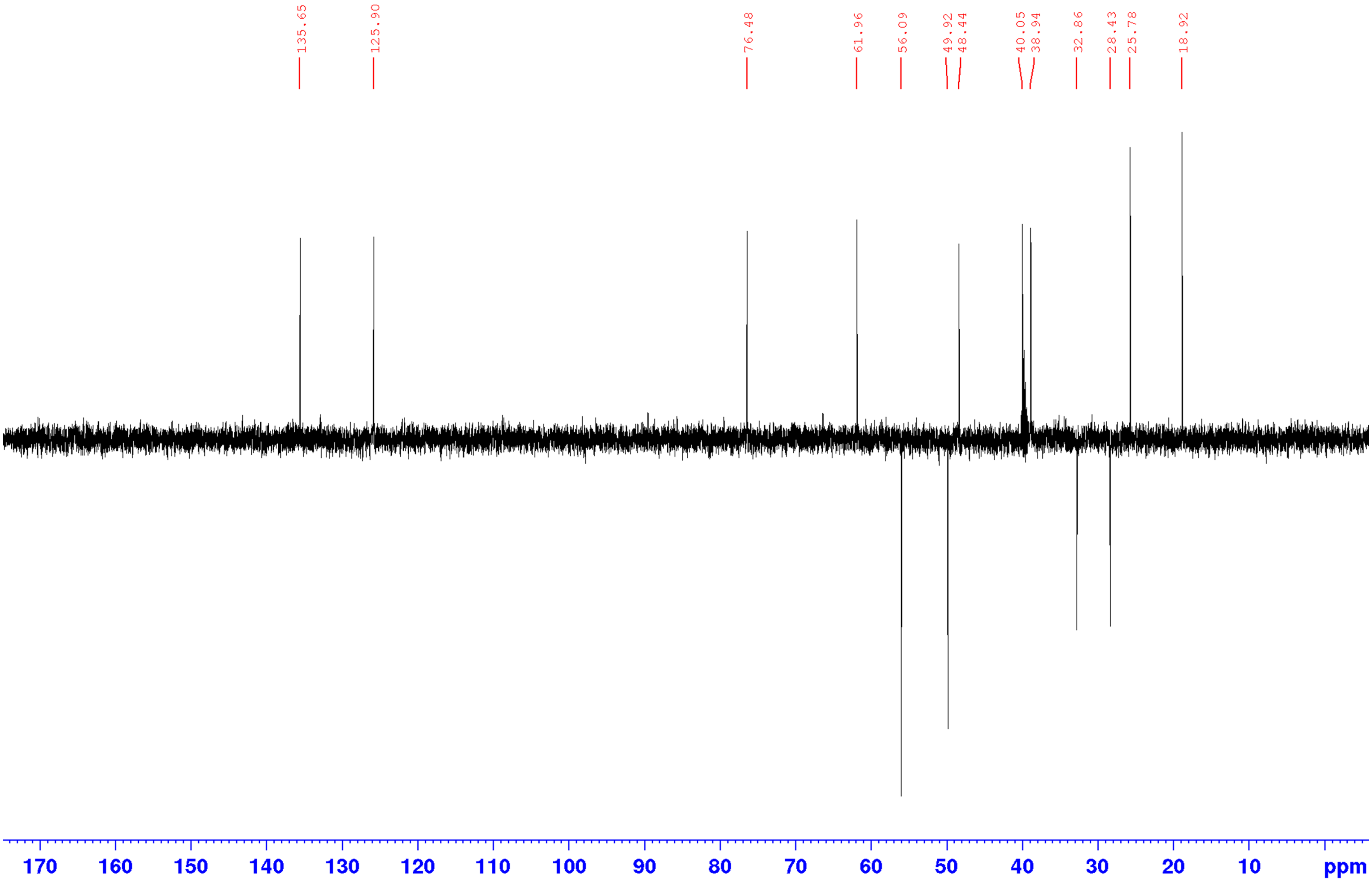

Figure S54. HSQC spectrum of **13** in DMSO-d6

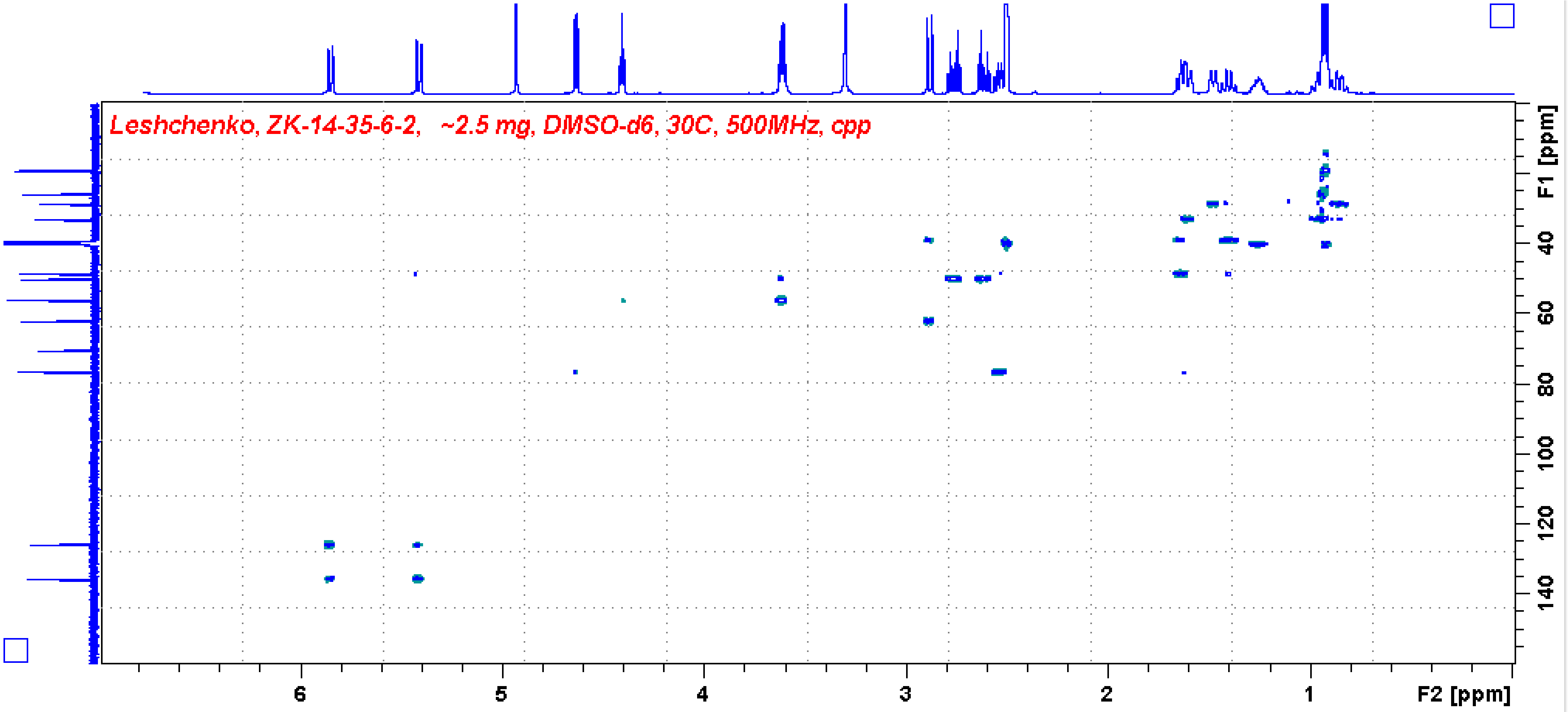

Figure S55. HMBC spectrum of **13** in DMSO-d6

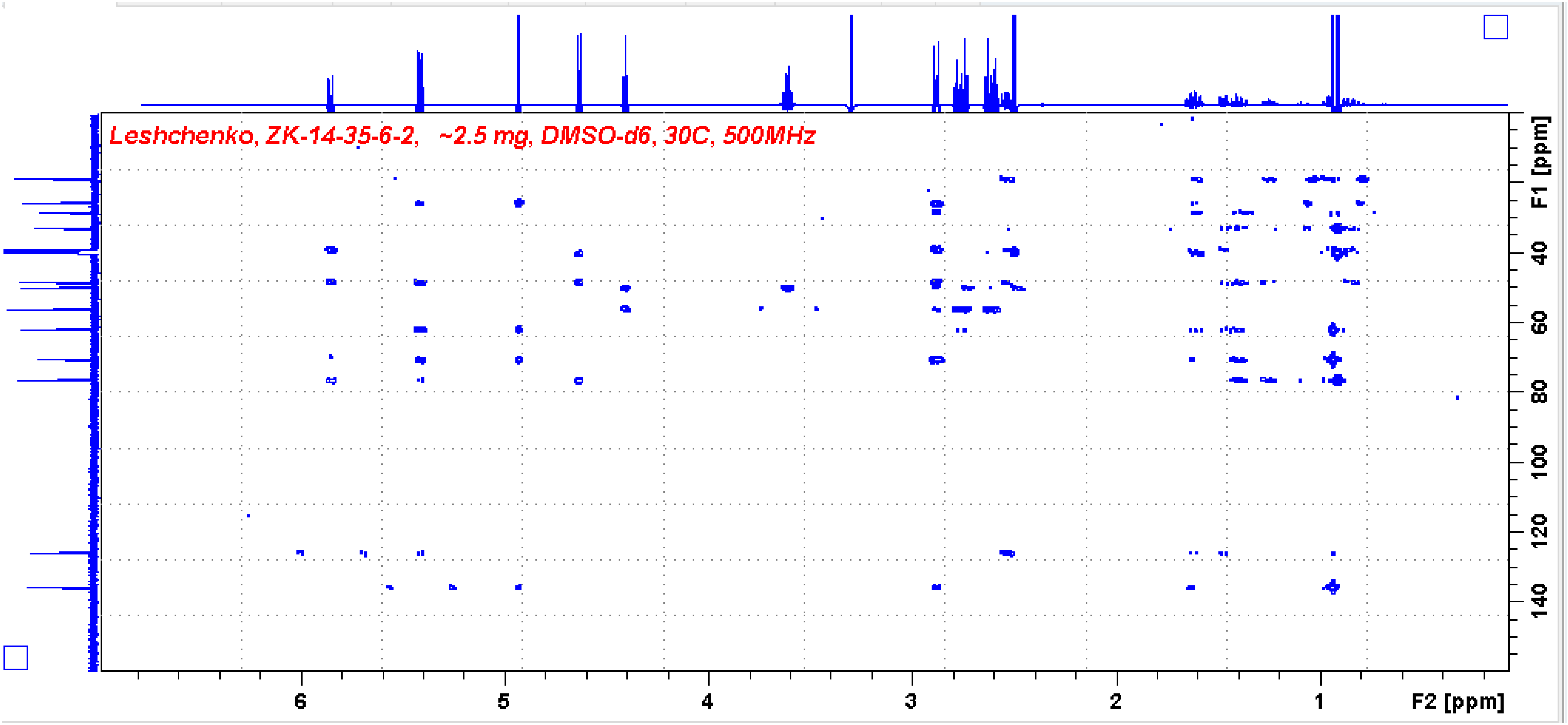

Figure S56. 1H-1H COSY spectrum of **13** in DMSO-d6

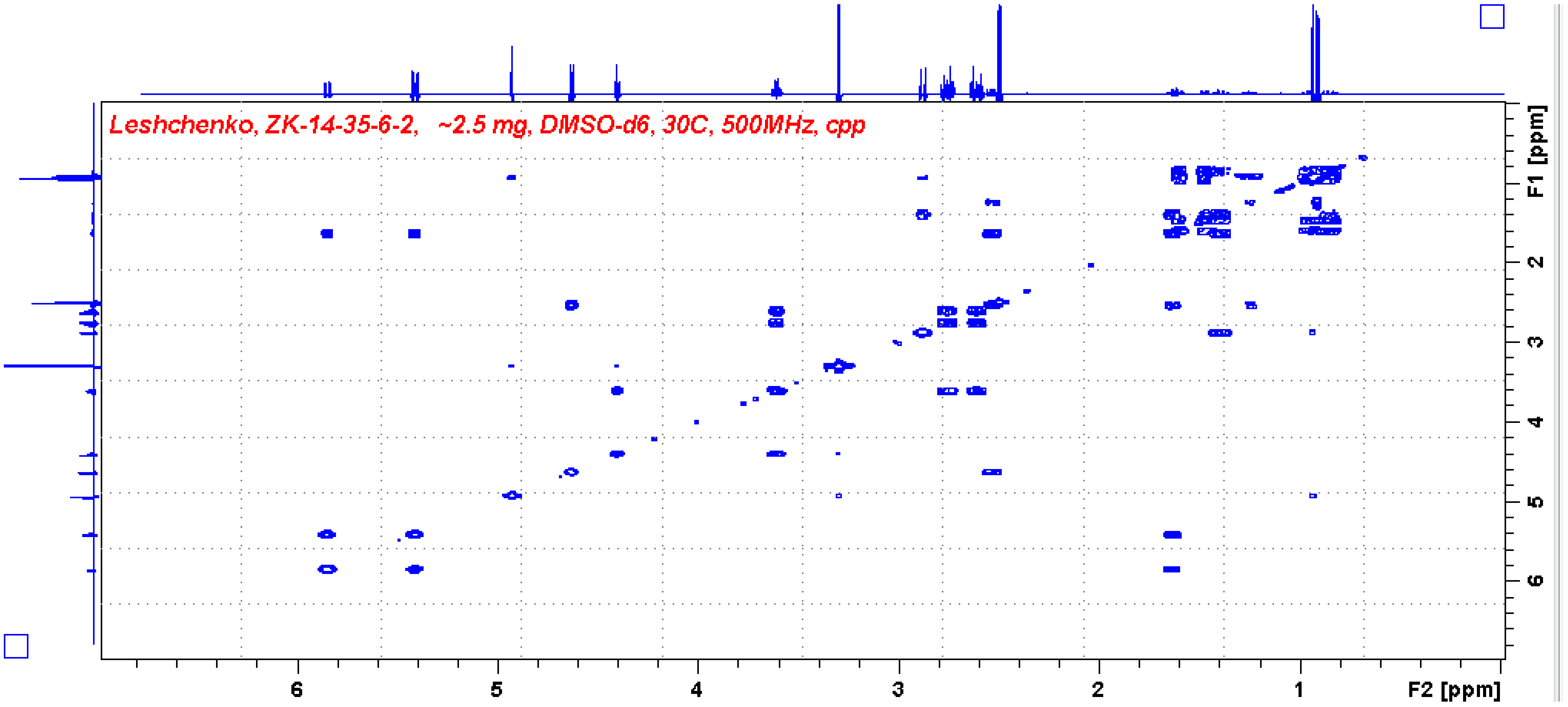

Figure S57. NOESY spectrum of **13** in DMSO-d6

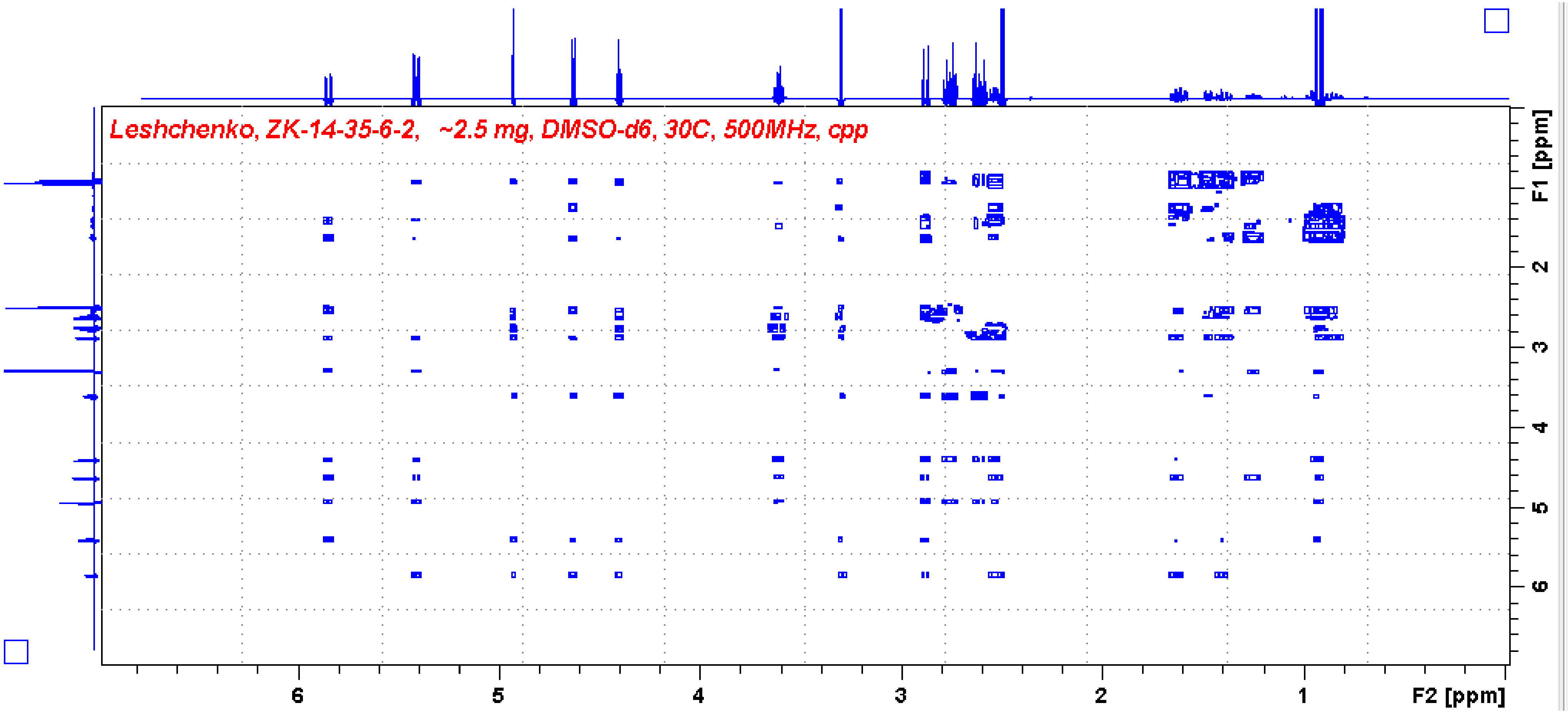

Figure S58. ^{1}H NMR spectrum (500.13 MHz) of **13** in $CDCl_3$

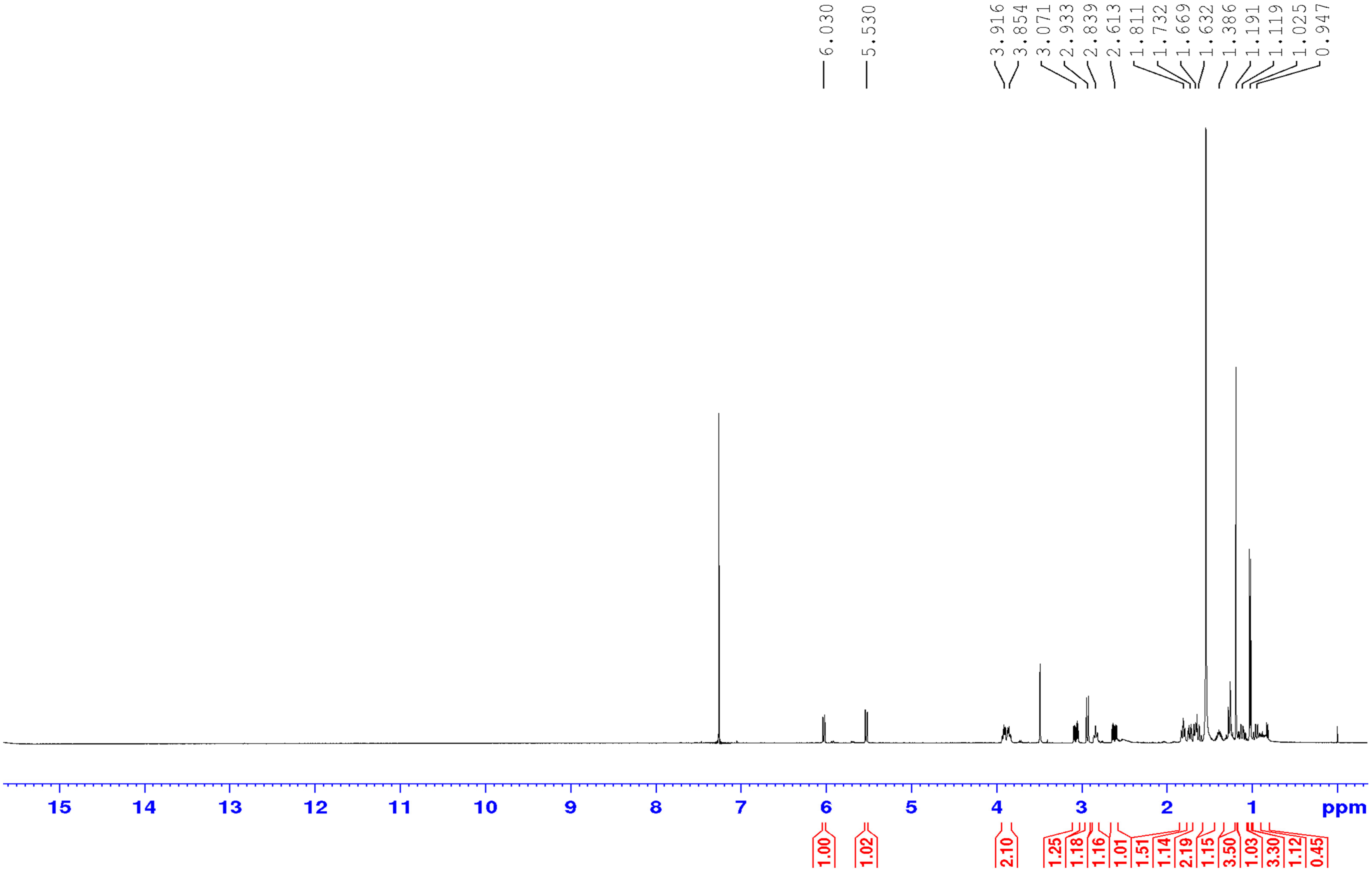

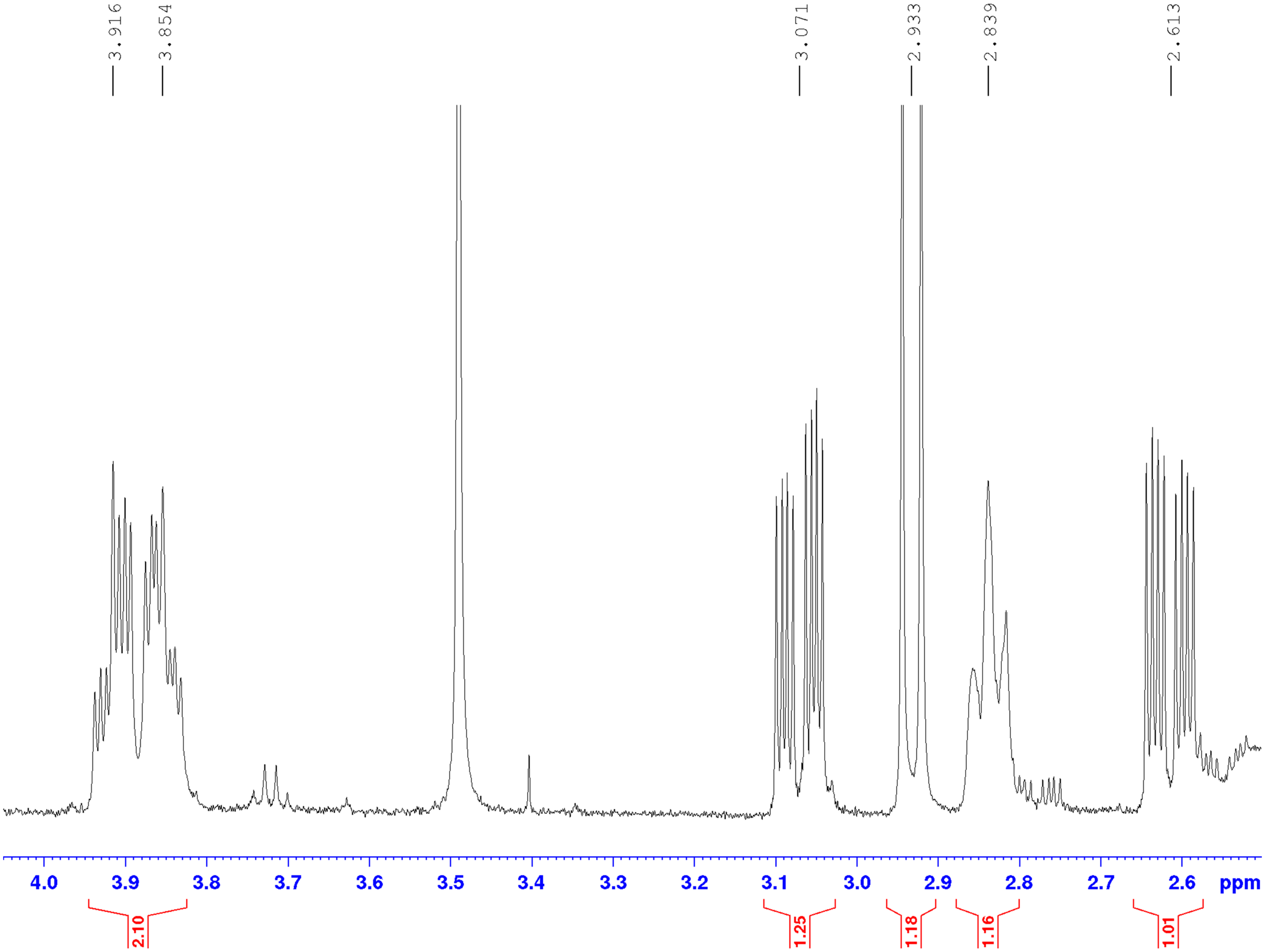

3.916
3.854
3.071
2.933
2.839
2.613
4.0
3.9
3.8
3.7
3.6
3.5
3.4
3.3
3.2
3.1
3.0
2.9
2.8
2.7
2.6
ppm
2.10
1.25
1.18
1.16
1.01

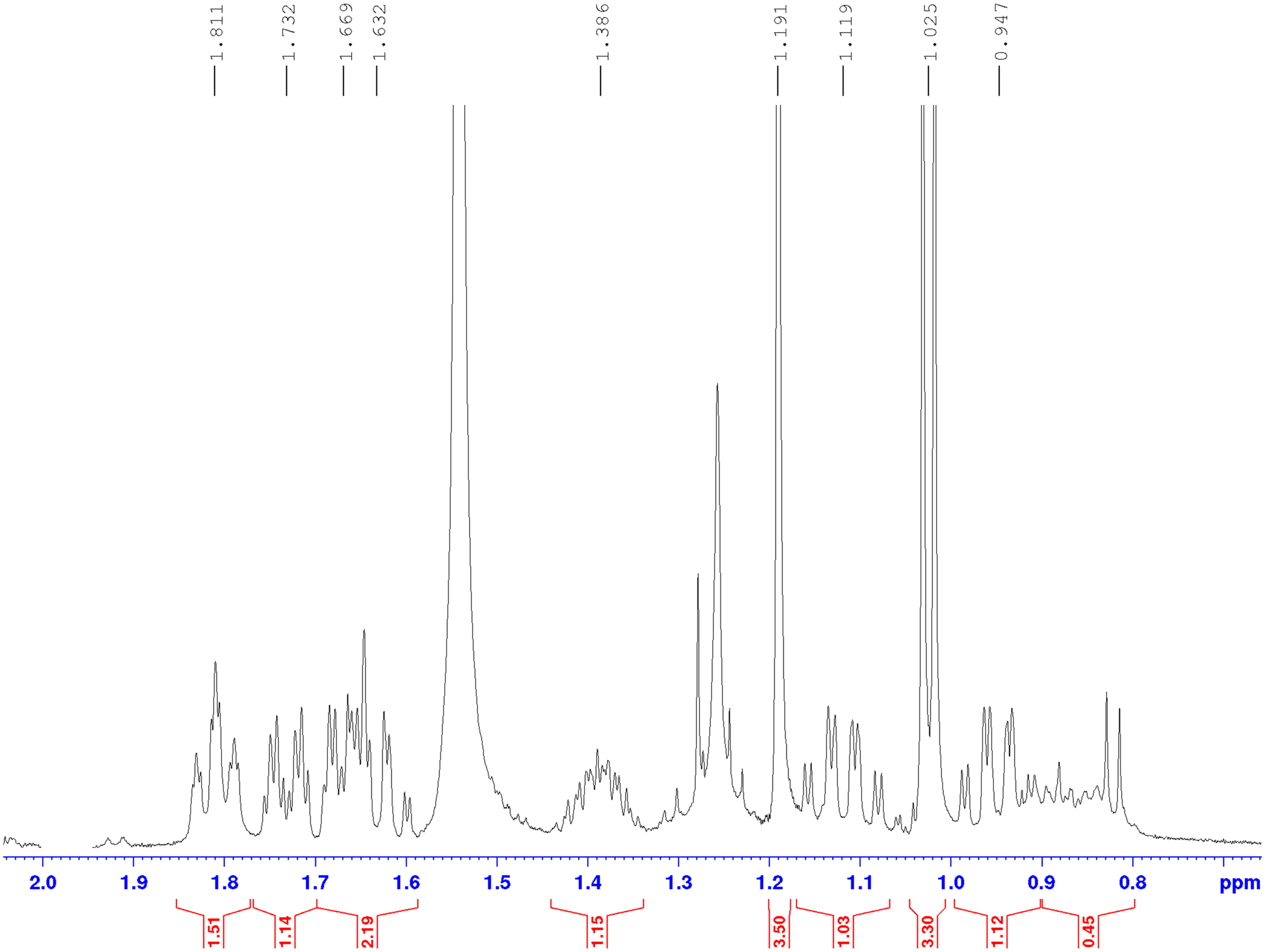

1.811
1.732
1.669
1.632
1.386
1.191
1.119
1.025
0.947
2.0
1.9
1.8
1.7
1.6
1.5
1.4
1.3
1.2
1.1
1.0
0.9
0.8
ppm
1.51
1.14
2.19
1.15
3.50
1.03
3.30
1.12
0.45

Figure S59. ^{13}C NMR spectrum (125.77 MHz) of **13** in $CDCl_3$

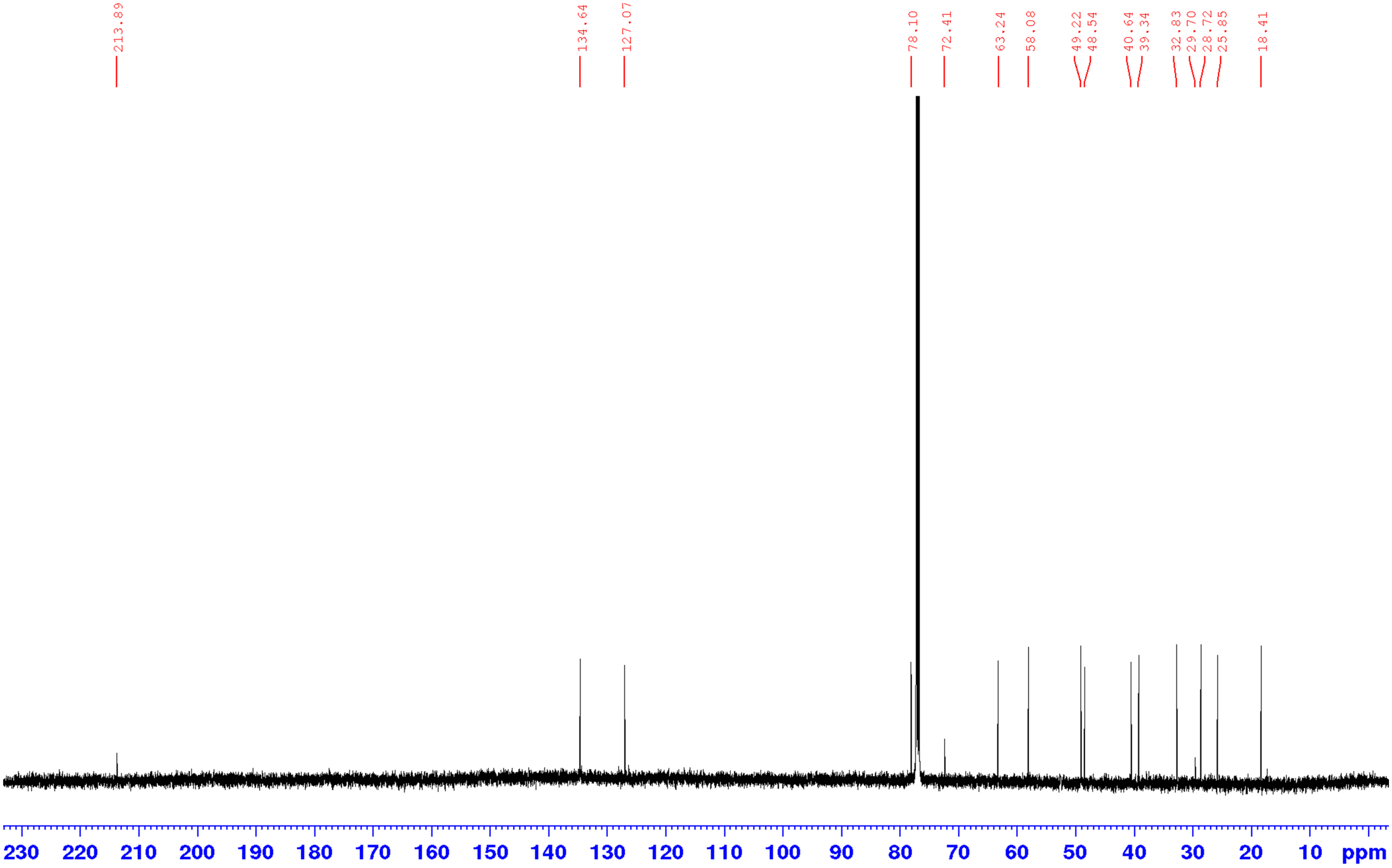

Figure S60. DEPT-135 spectrum (125.77 MHz) of **13** in $CDCl_3$

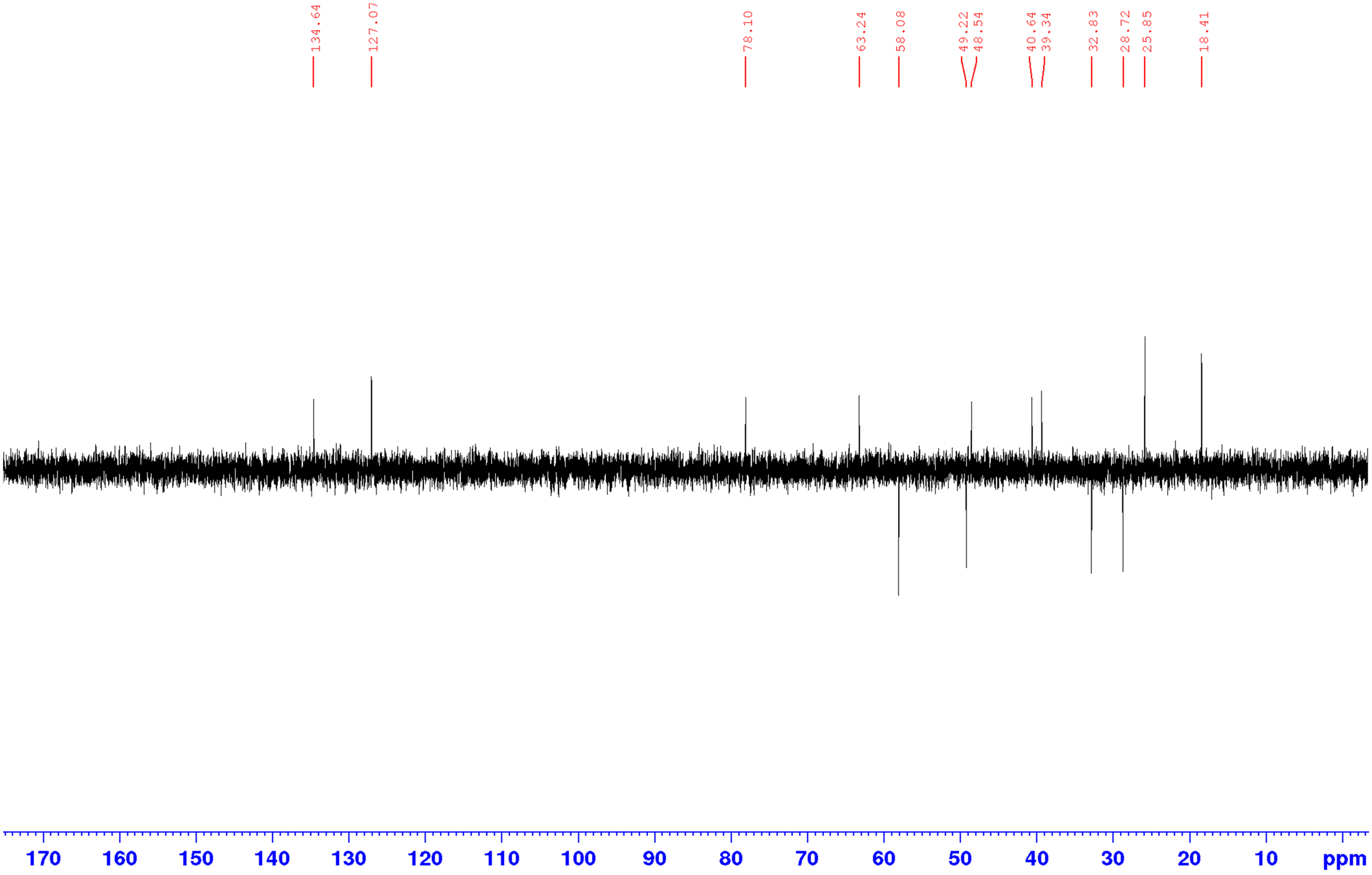

Figure S61. DEPT-90 spectrum (125.77 MHz) of **13** in $CDCl_3$

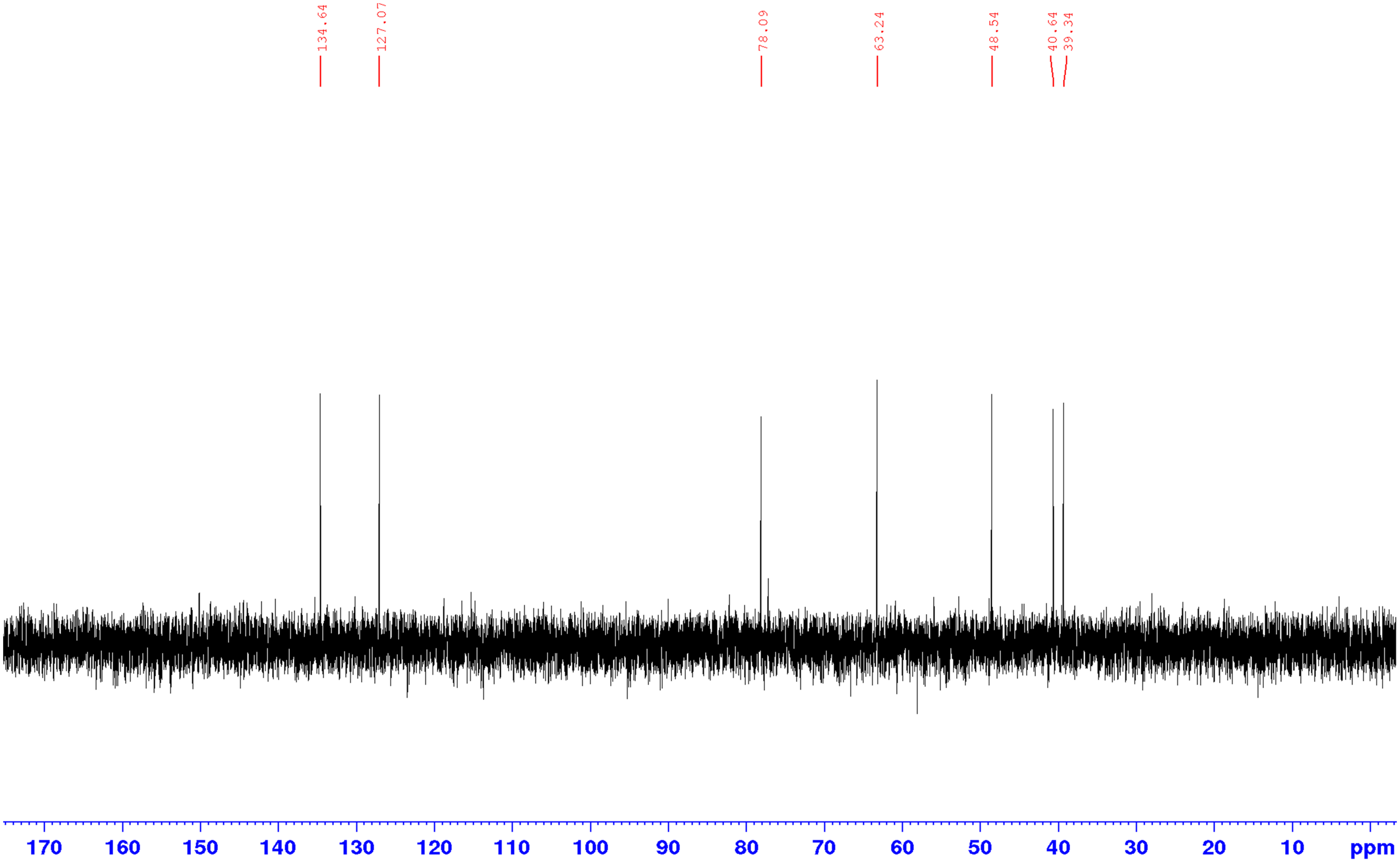

Figure S62. HSQC spectrum of **13** in $CDCl_3$

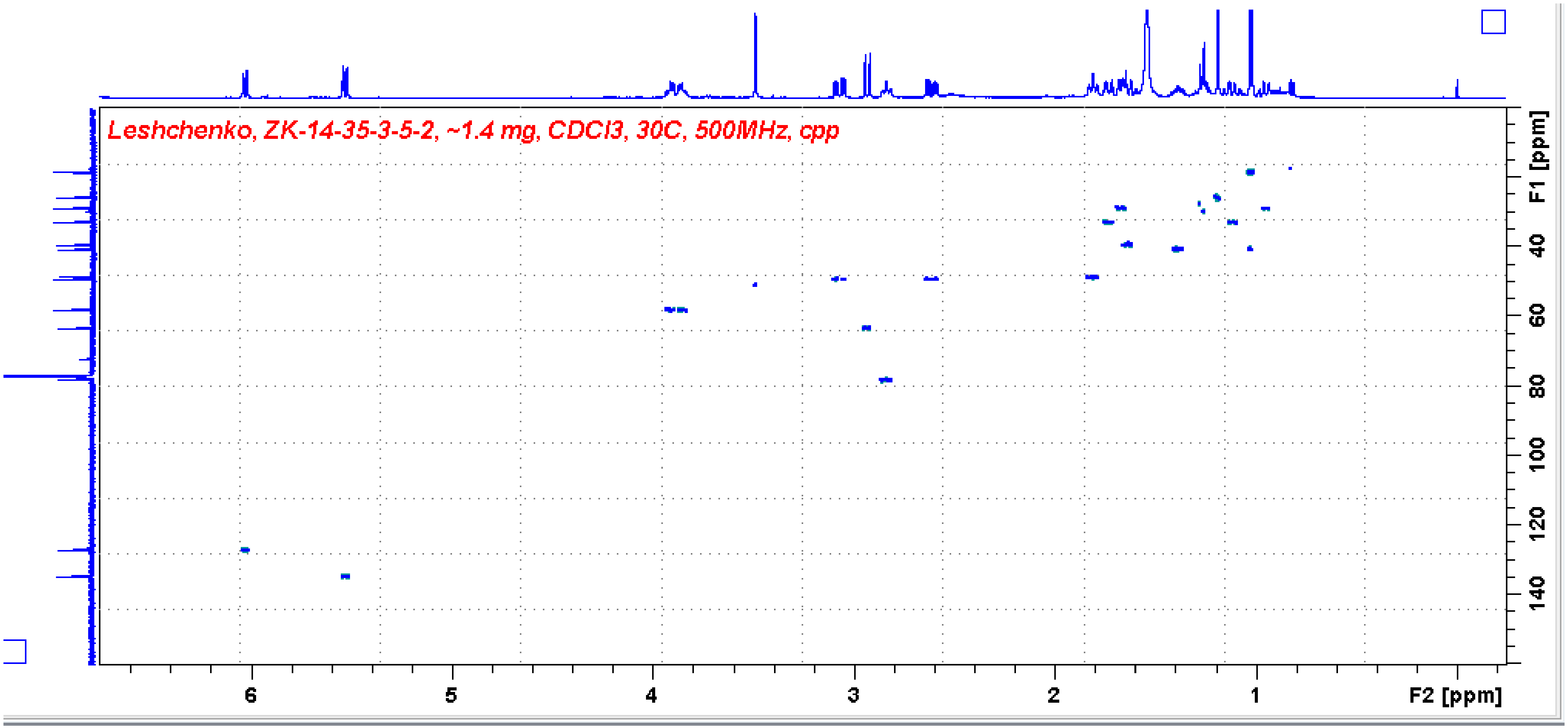

Figure S63. HMBC spectrum of **13** in $CDCl_3$

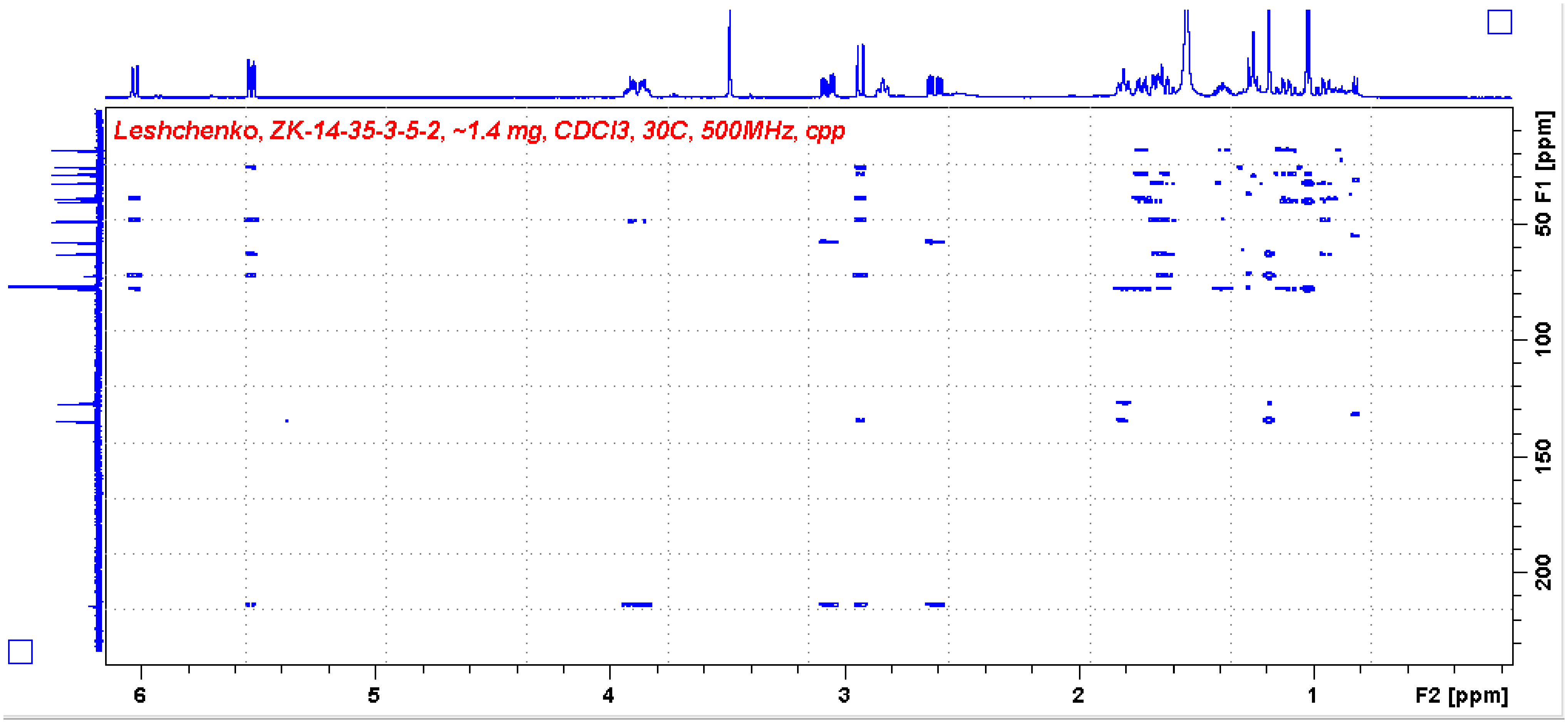

Figure S64. $^{1}H - ^{1}H$ COSY spectrum of **13** in $CDCl_3$

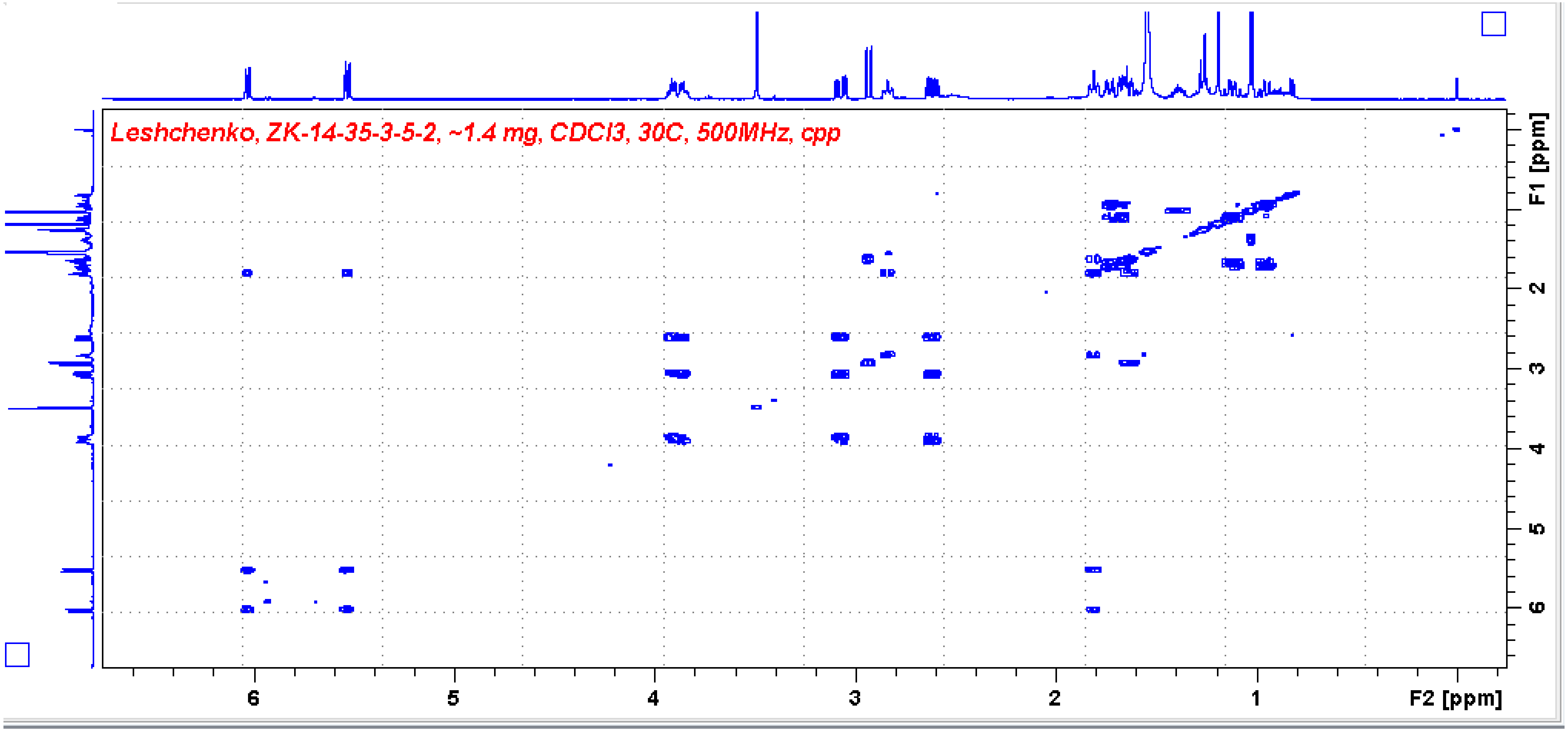

Figure S65. NOESY spectrum of **13** in $CDCl_3$

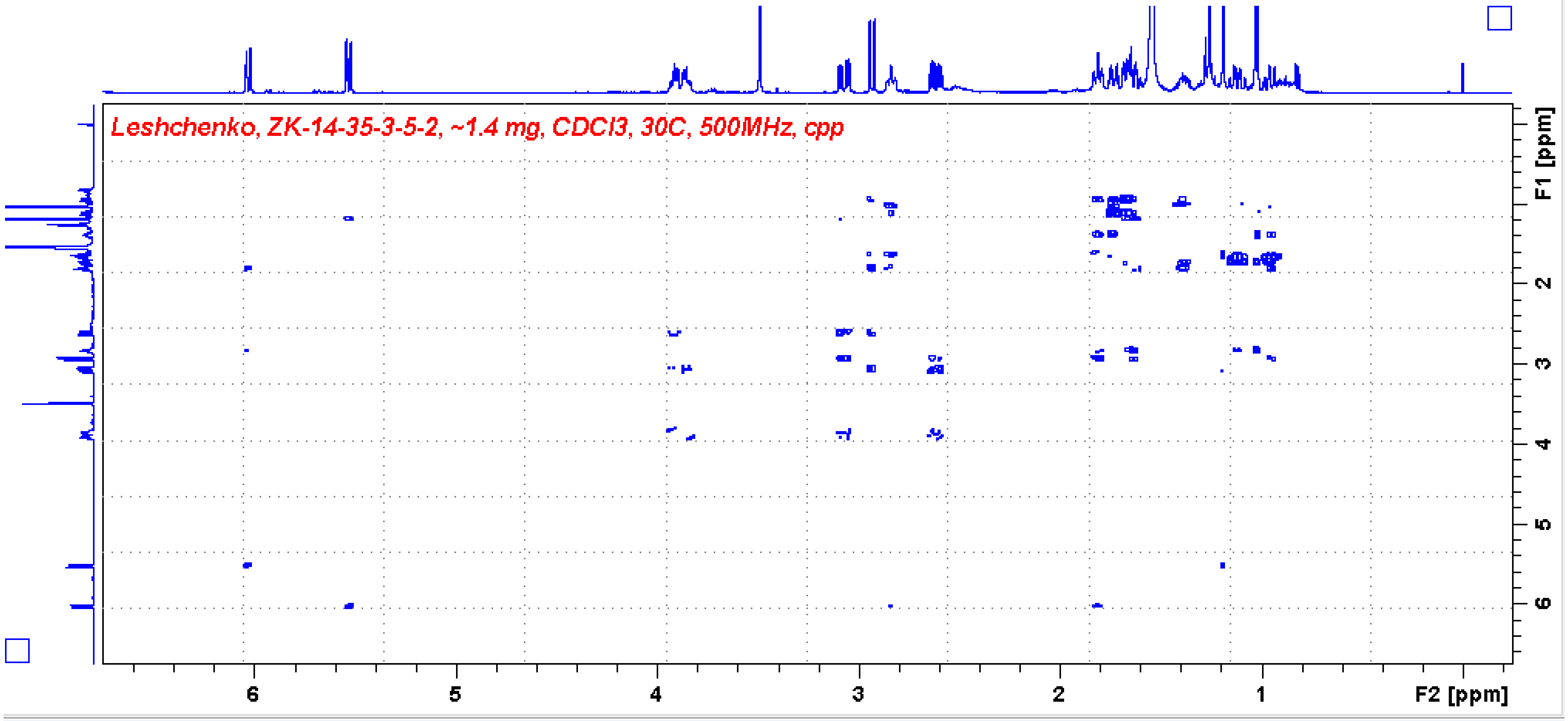

Figure S66. ^{13}C NMR spectrum of **13** and **6** in $CDCl_3$

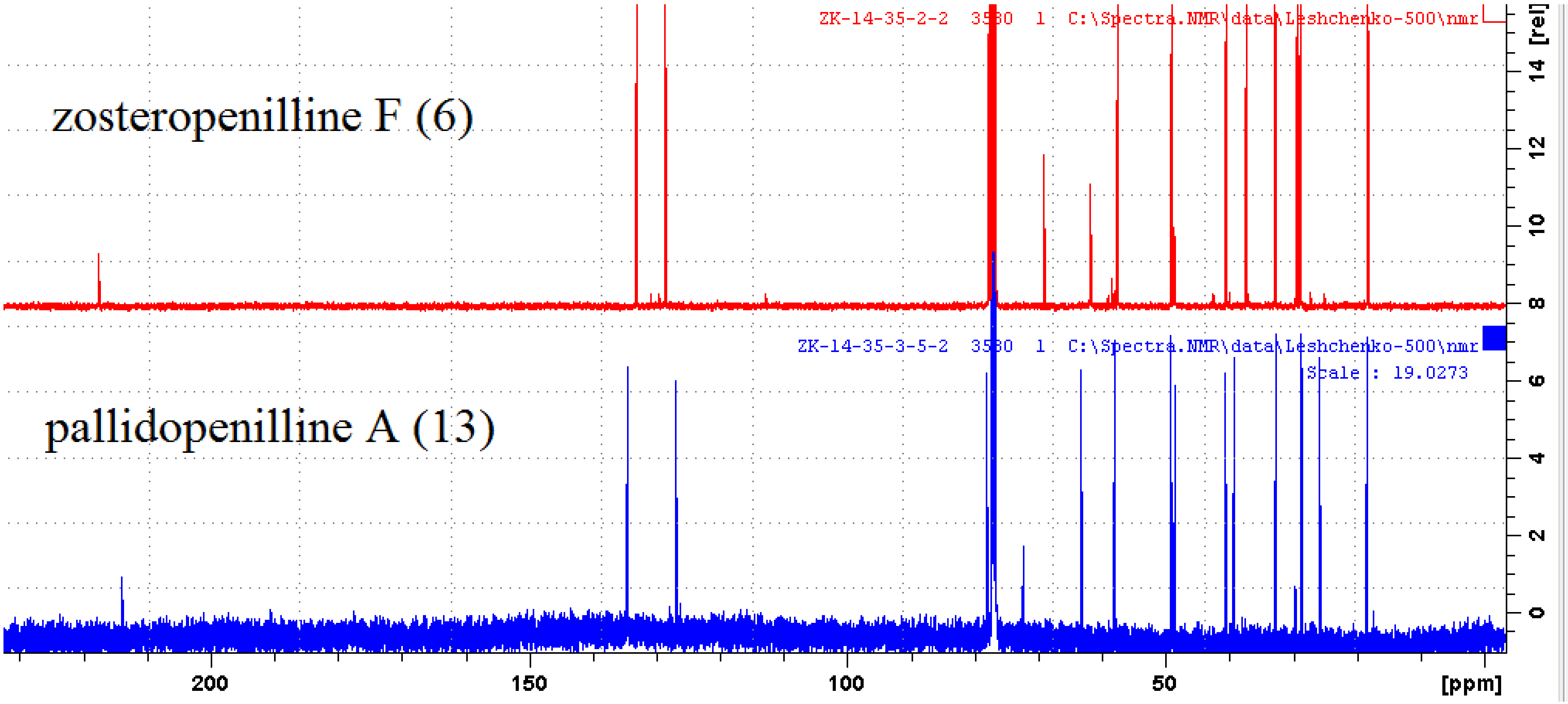

Figure S67. DEPT spectrum of **13** and **6** in $CDCl_3$

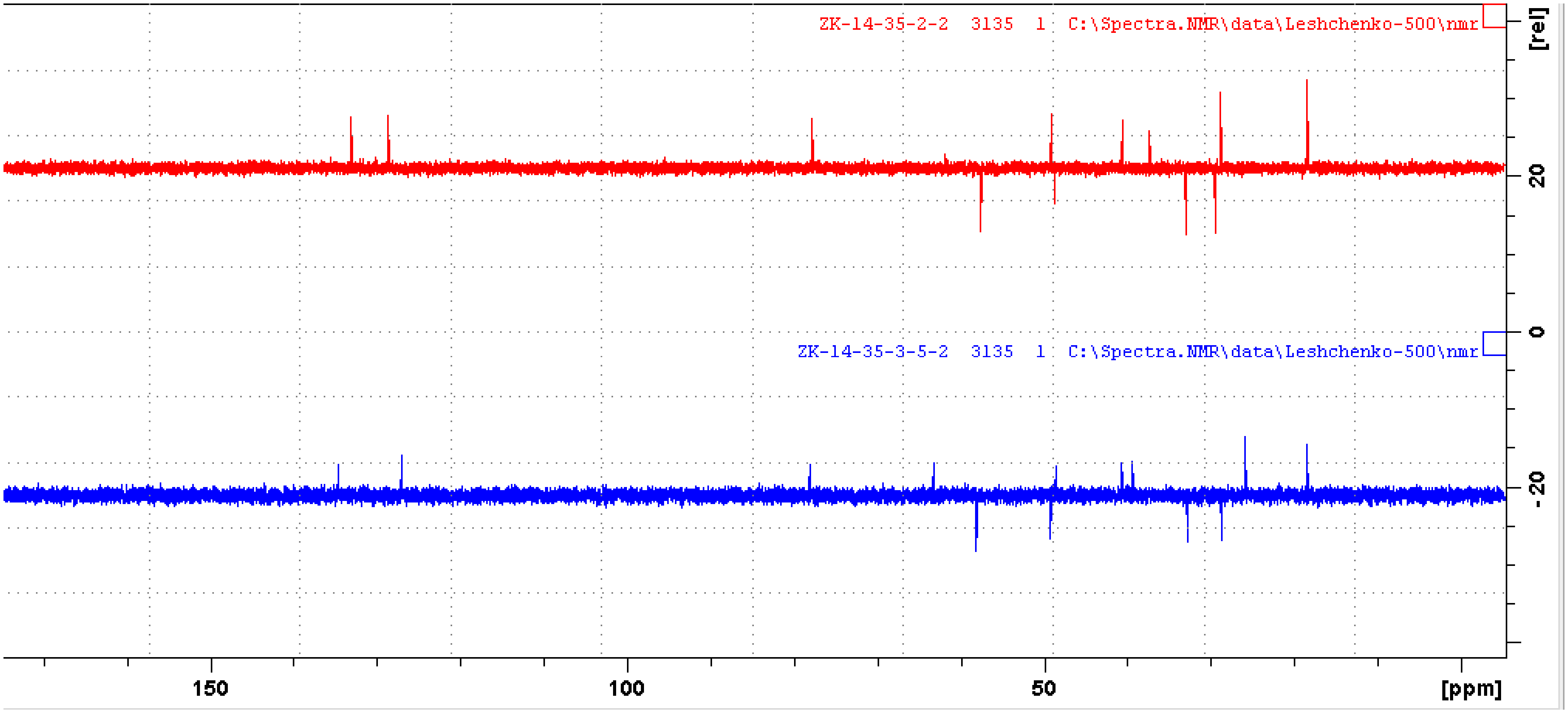

Figure S68. ^{1}H NMR spectrum of **13** and **6** in $CDCl_3$

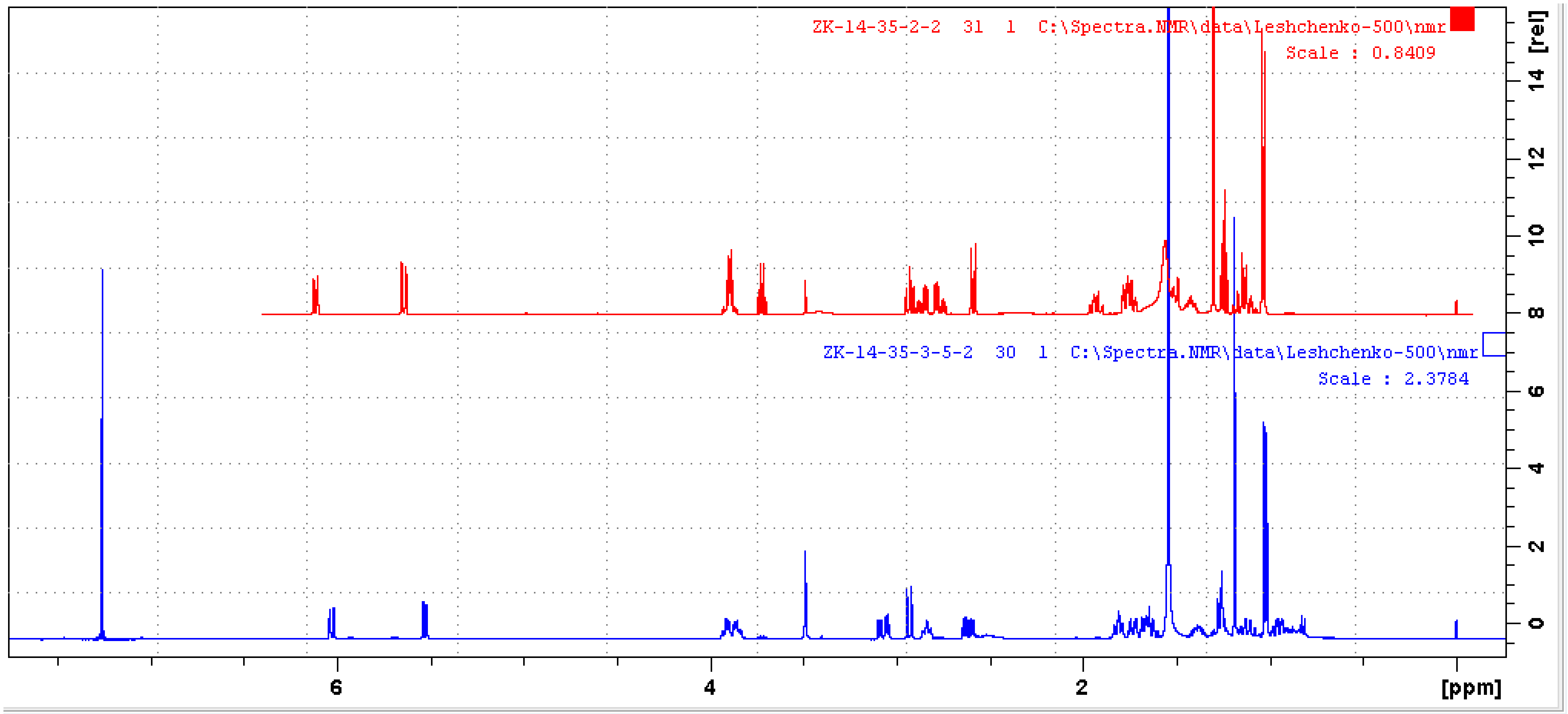

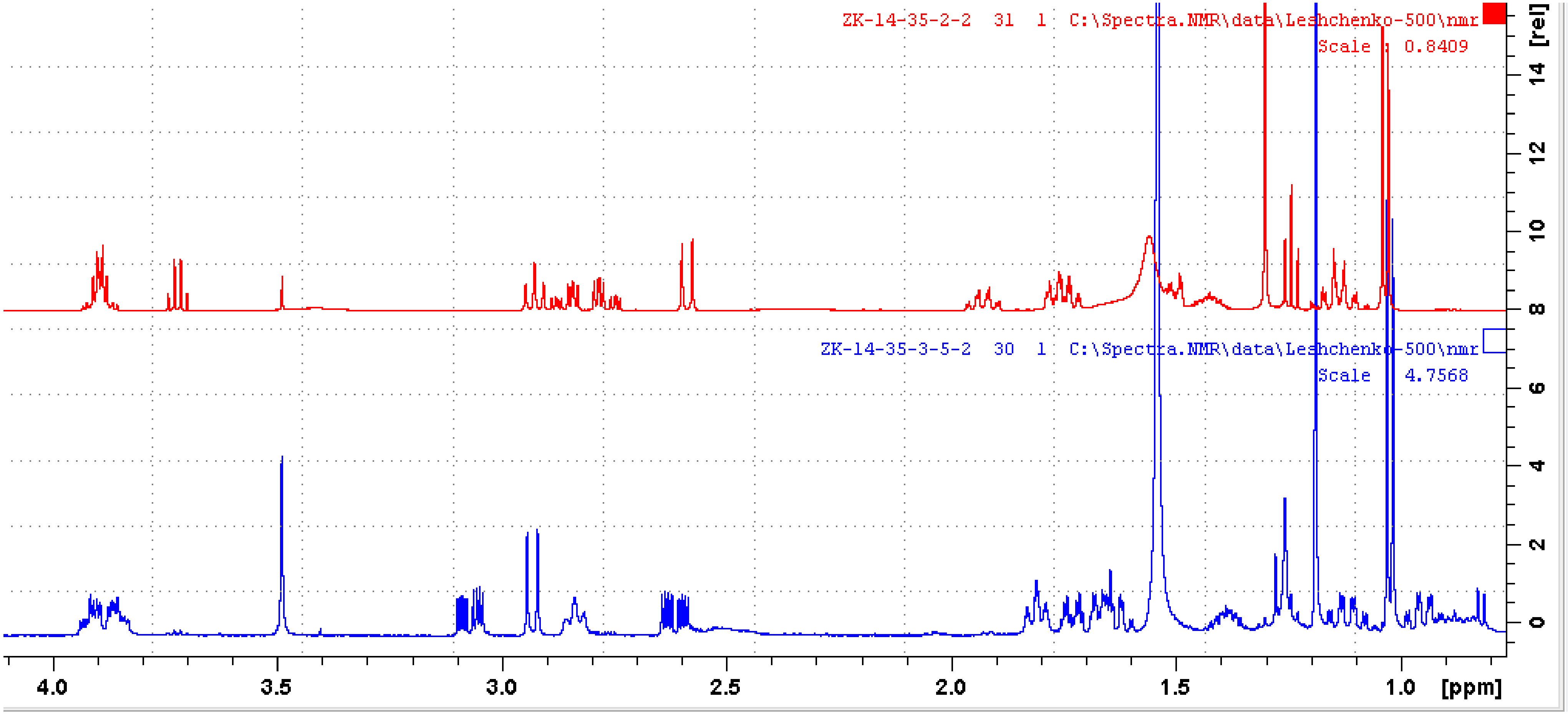
ZK-14-35-2-2 31 1 C:\Spectra.NMR\data\Leshchenko-500\nmr
Scale : 0.8409
ZK-14-35-3-5-2 30 1 C:\Spectra.NMR\data\Leshchenko-500\nmr
Scale : 4.7568
[rel]
[ppm]
4.0
3.5
3.0
2.5
2.0
1.5
1.0
14
12
10
8
6
4
2
0

Figure S69. ^{1}H NMR spectrum (500.13 MHz) of **7** in $CDCl_3$

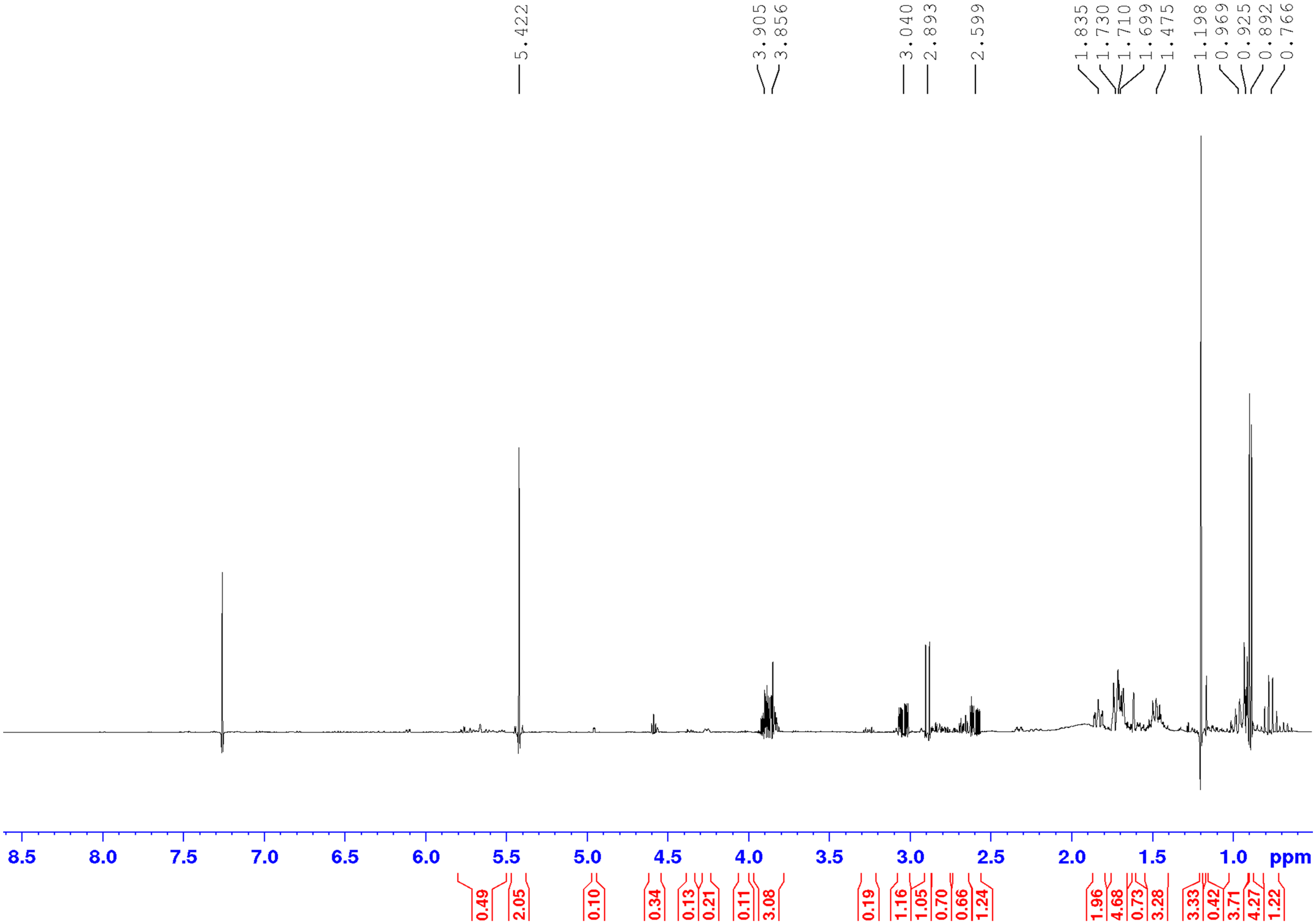

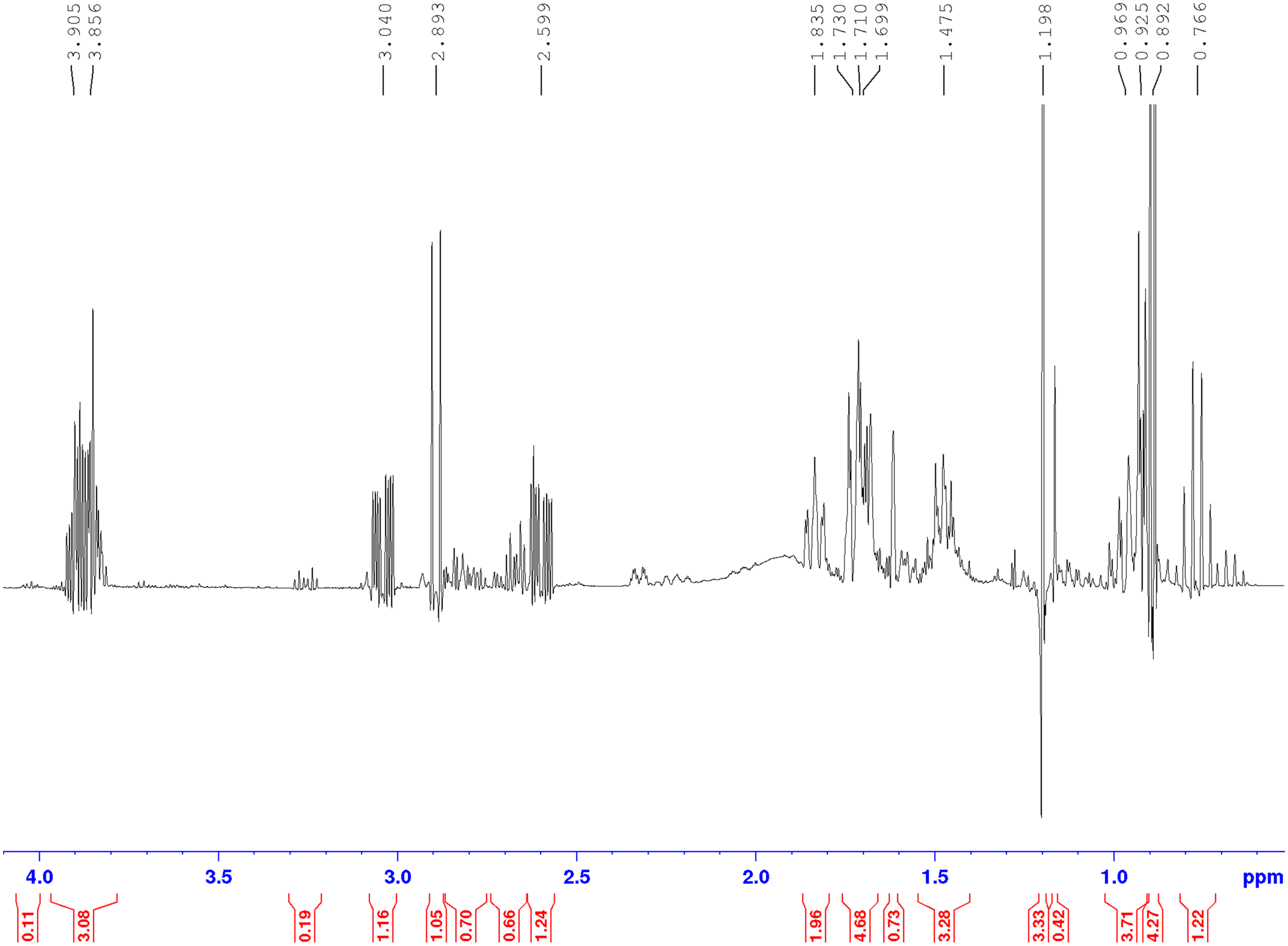

3.905
3.856
3.040
2.893
2.599
1.835
1.730
1.710
1.699
1.475
1.198
0.969
0.925
0.892
0.766
4.0
3.5
3.0
2.5
2.0
1.5
1.0
ppm
0.11
3.08
0.19
1.16
1.05
0.70
0.66
1.24
1.96
4.68
0.73
3.28
3.33
0.42
3.71
4.27
1.22

Figure S70. ^{13}C NMR spectrum (125.77 MHz) of **7** in $CDCl_3$

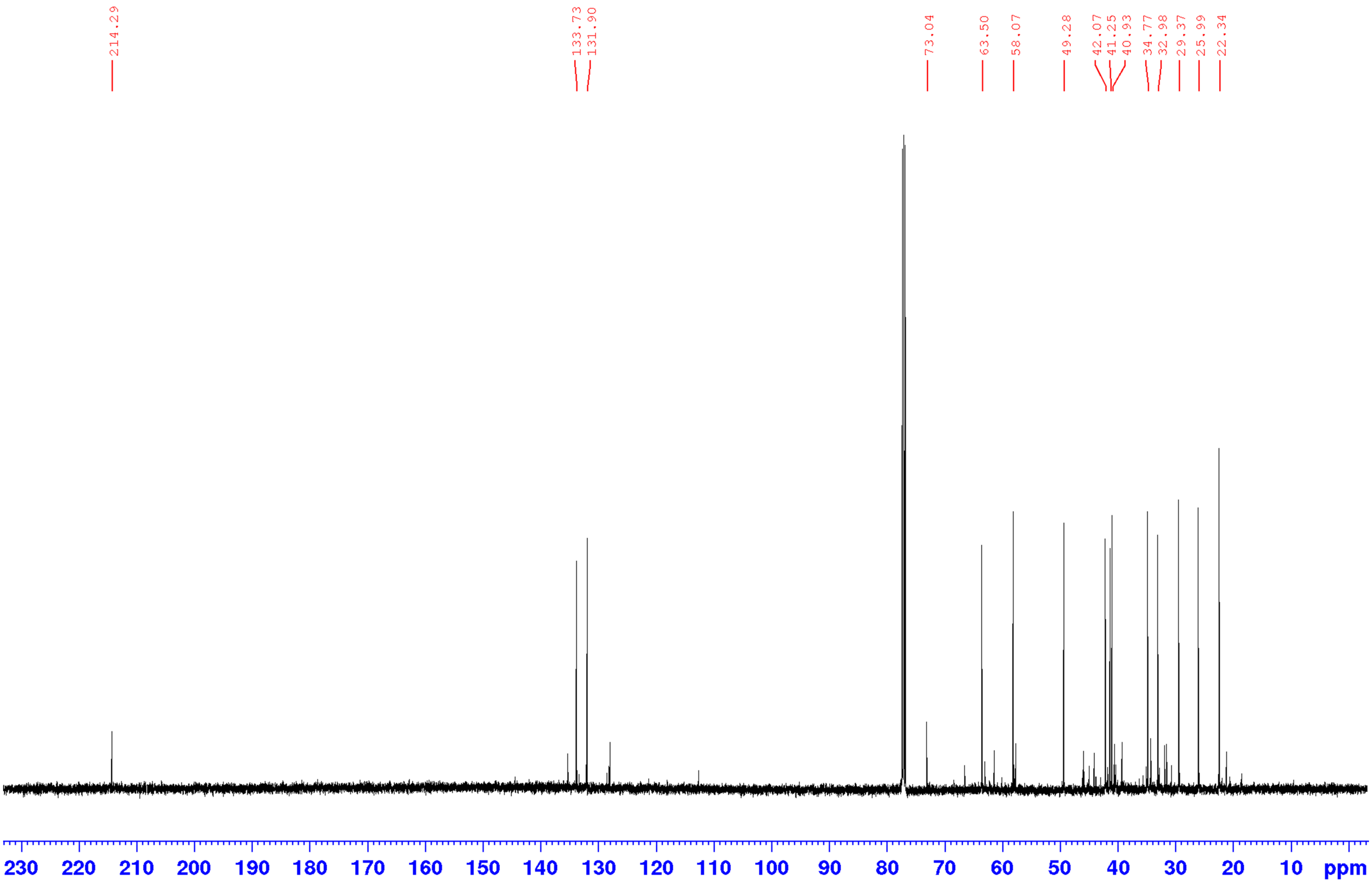

Figure S71. DEPT-135 spectrum (125.77 MHz) of **7** in $CDCl_3$

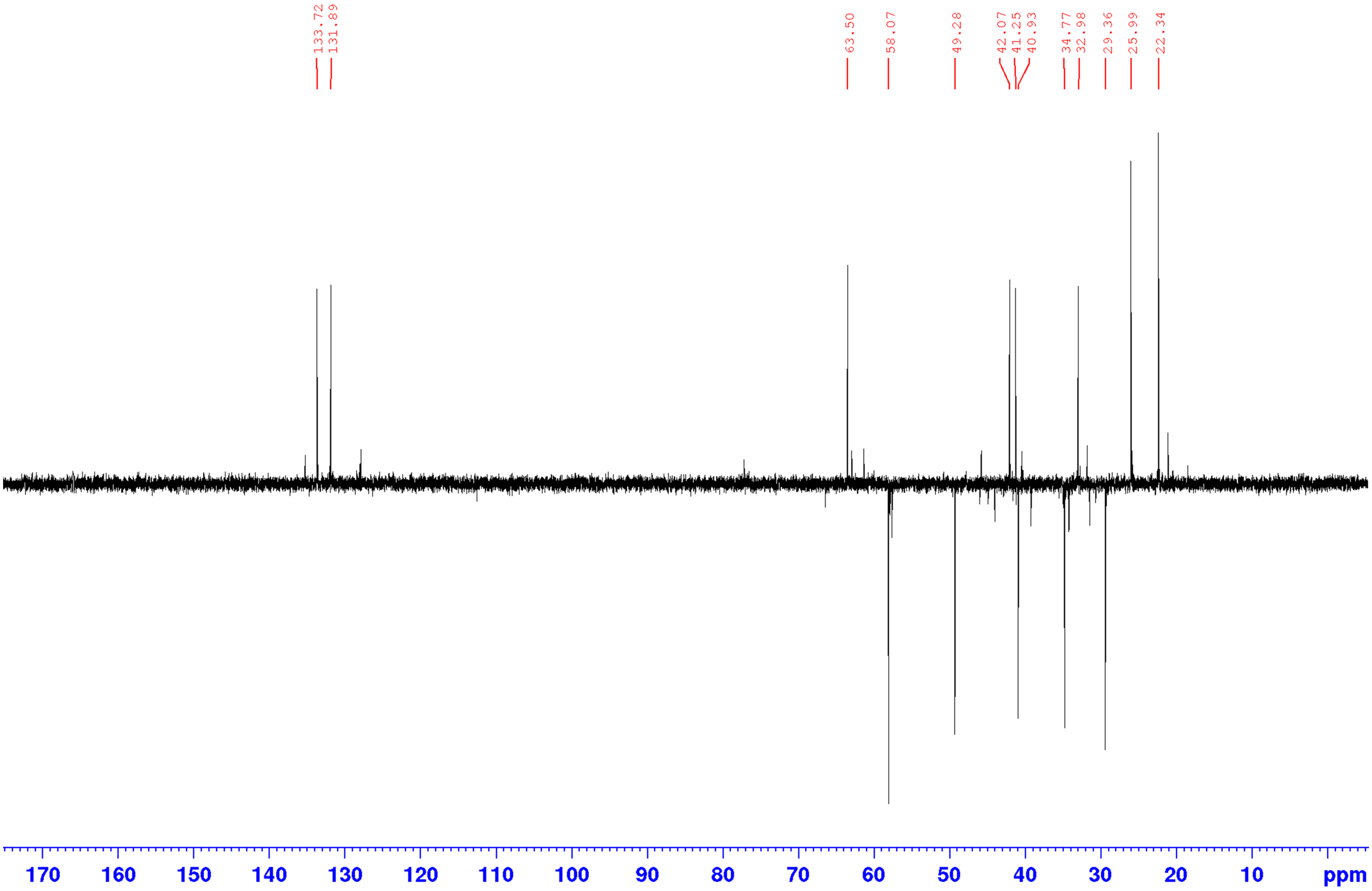

Figure S72. DEPT-90 spectrum (125.77 MHz) of **7** in $CDCl_3$

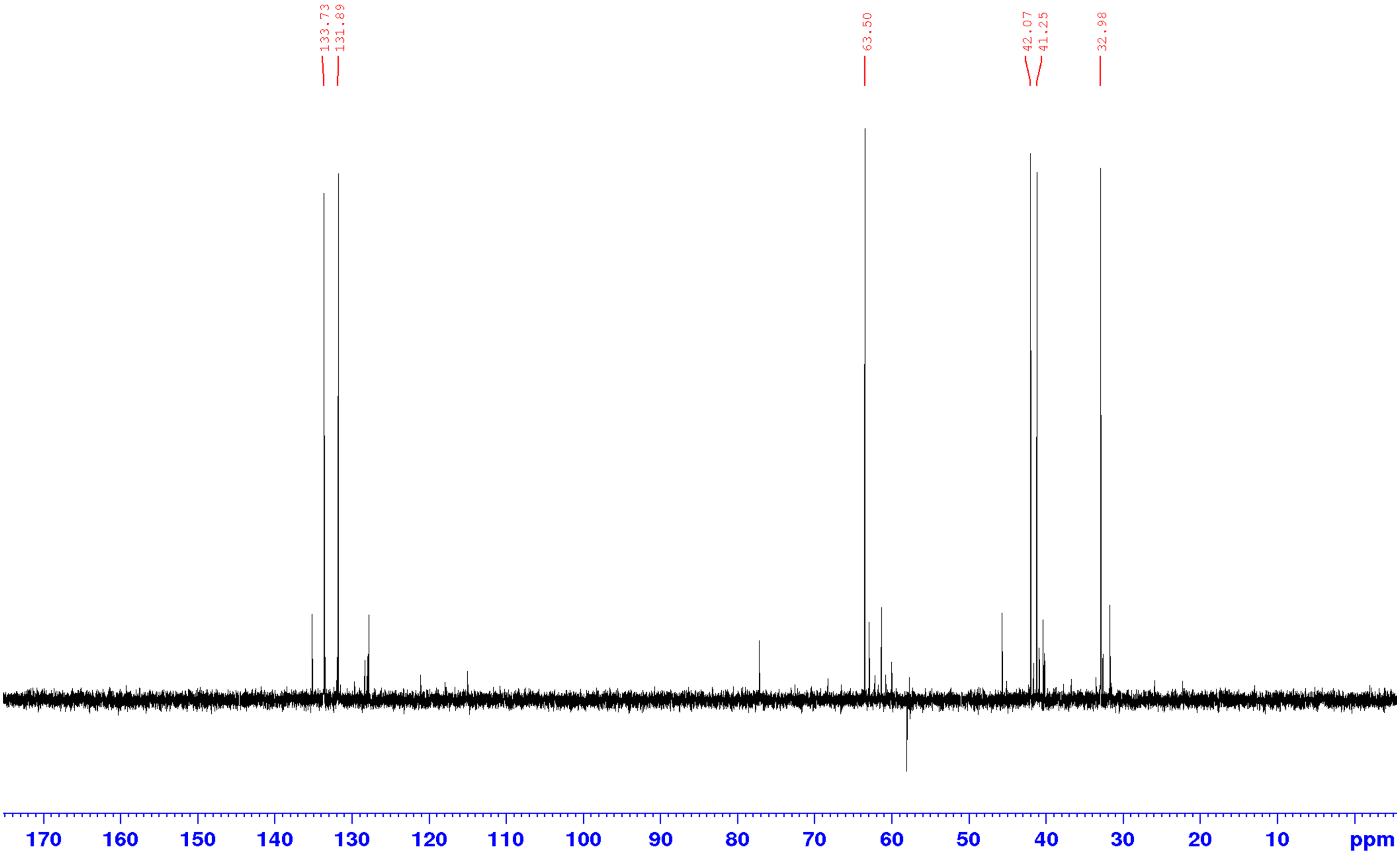

Figure S73. HSQC spectrum of **7** in $CDCl_3$

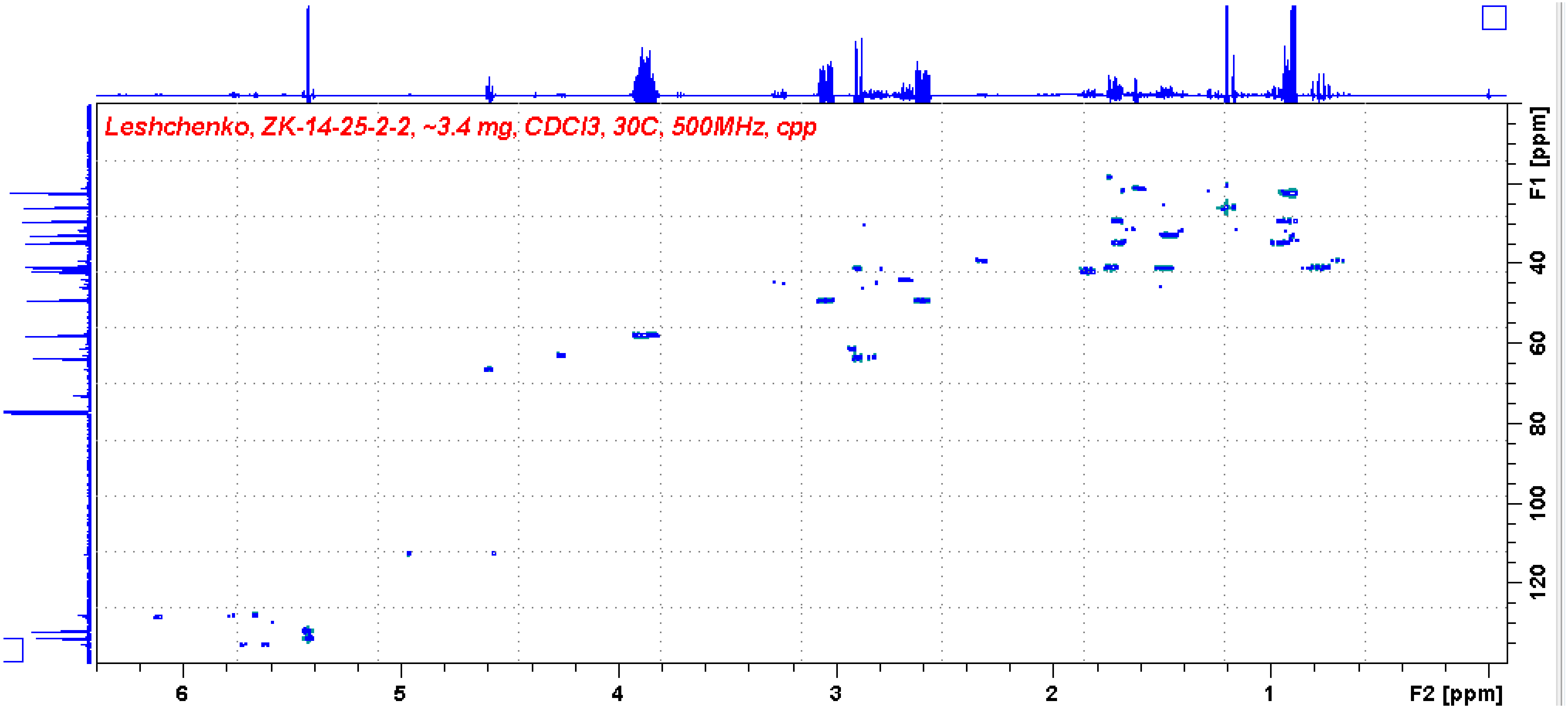

Figure S74. HMBC spectrum of **7** in $CDCl_3$

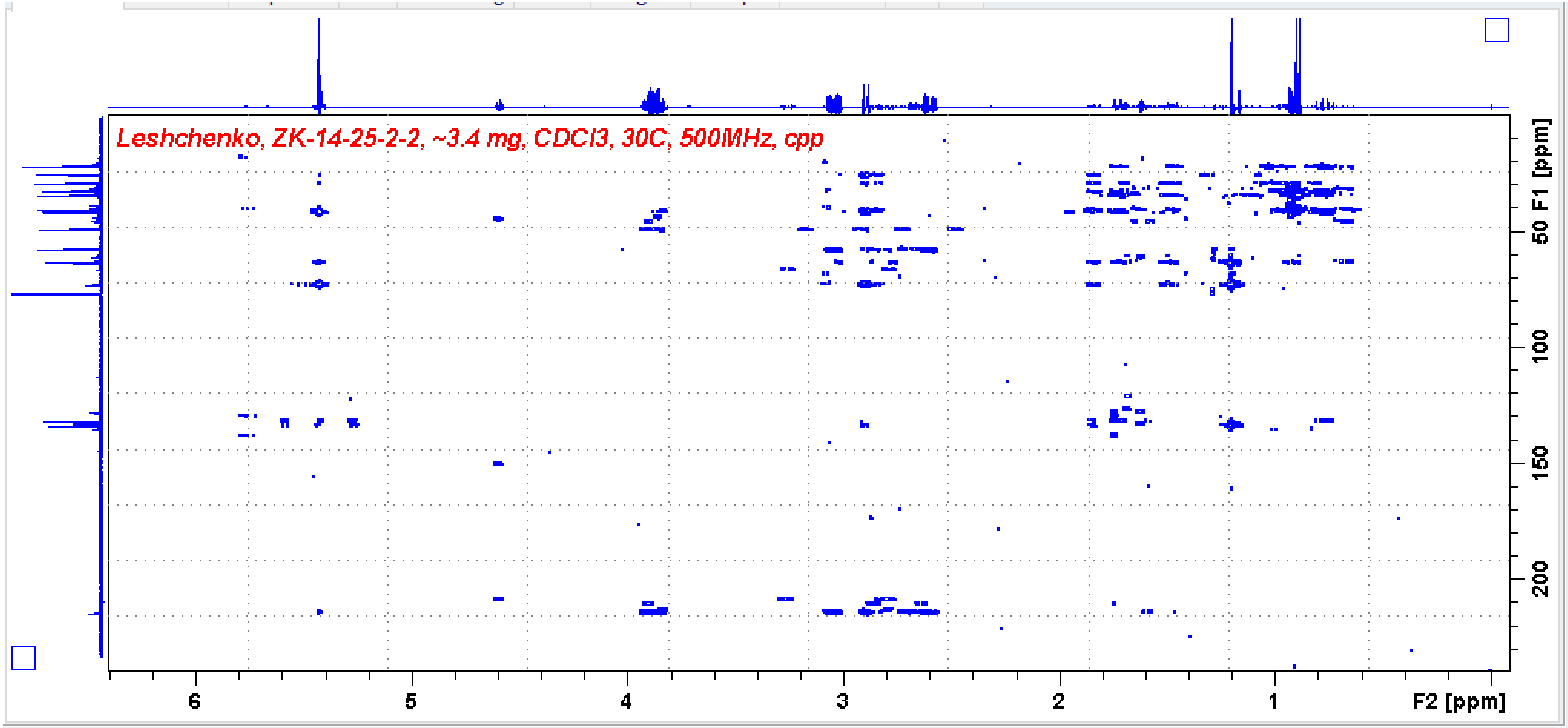

Figure S75. 1H-1H COSY spectrum of **7** in $CDCl_3$

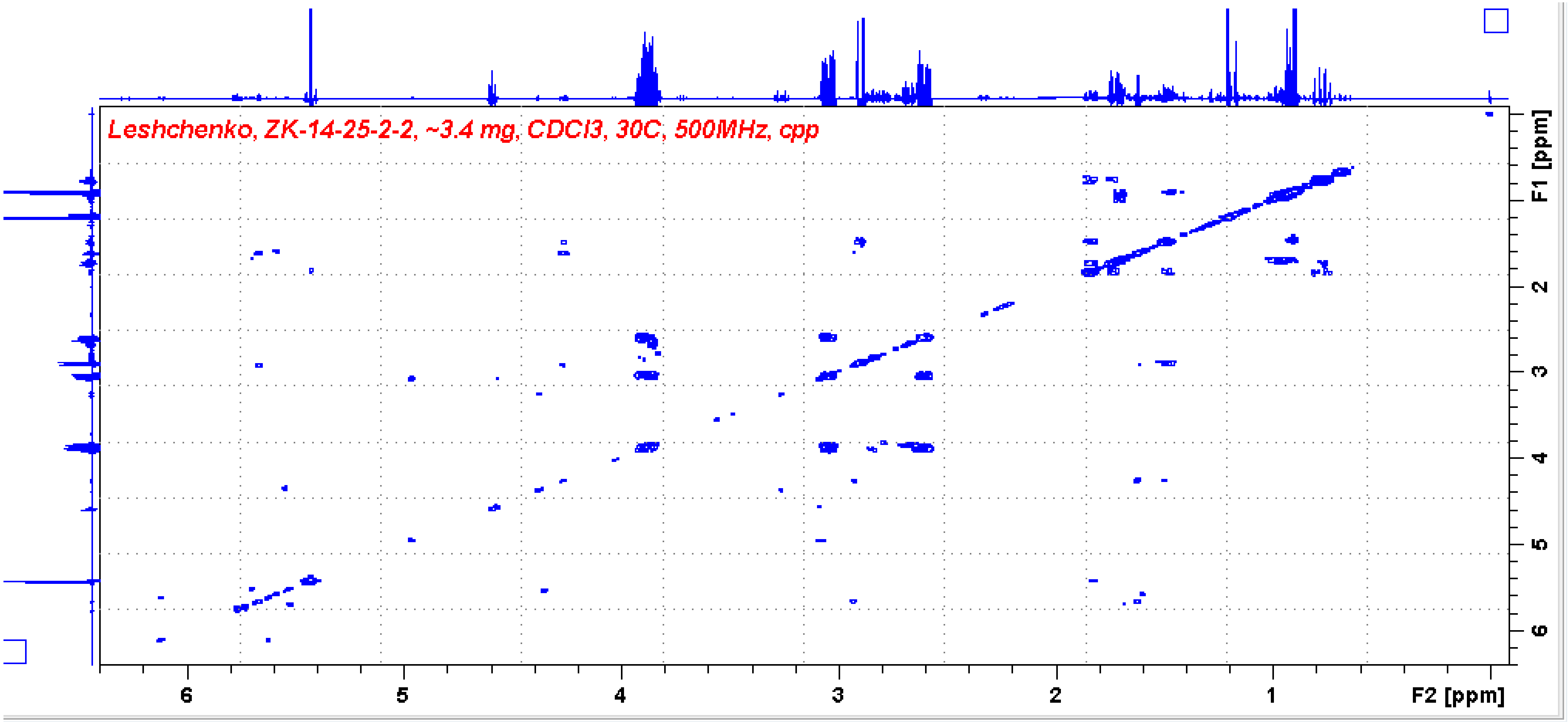

Figure S76. NOESY spectrum of **7** in $CDCl_3$

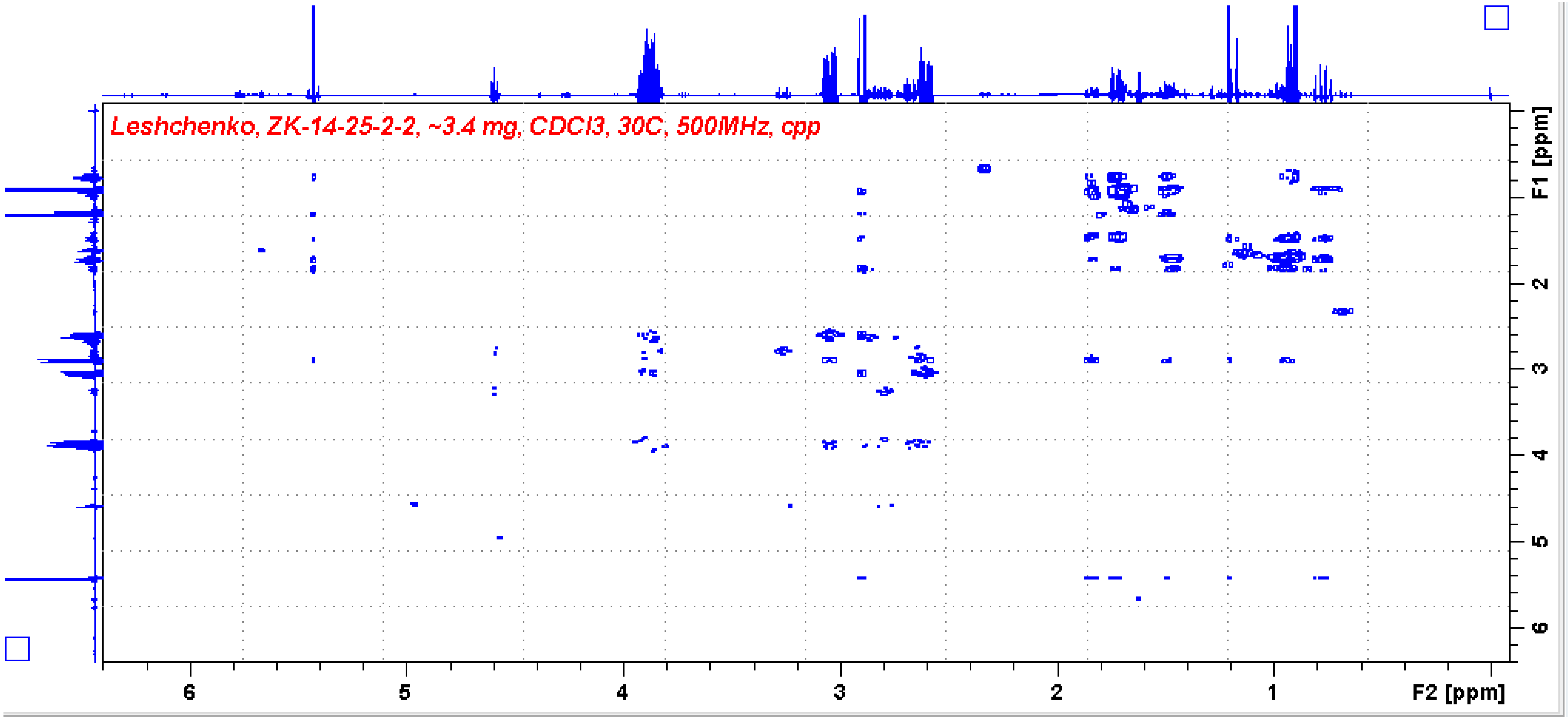

Figure S77. NOESY spectrum of **7** in Dmso-d6

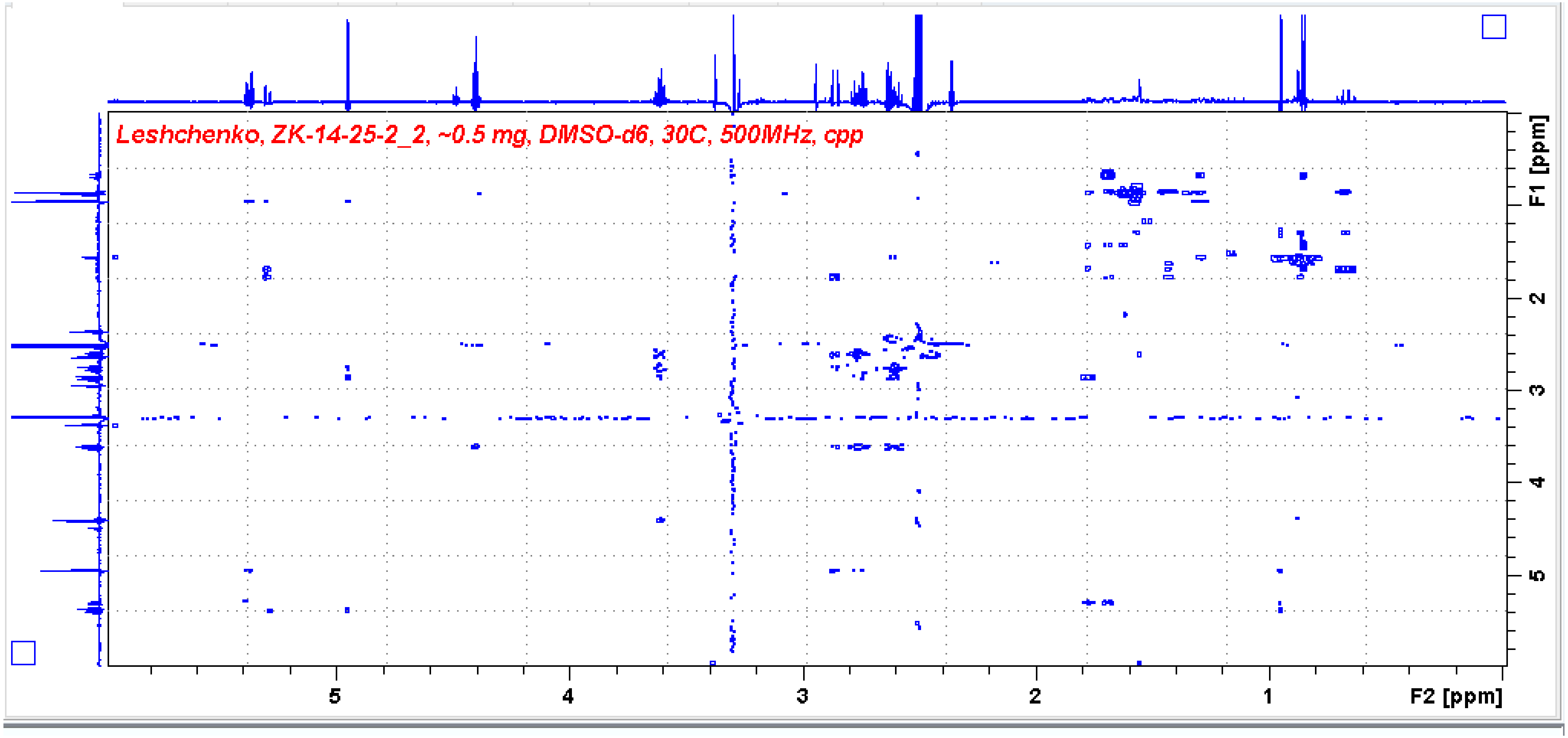

Figure S78. ^{1}H NMR spectrum (700.13 MHz) of **8** in $CDCl_3$

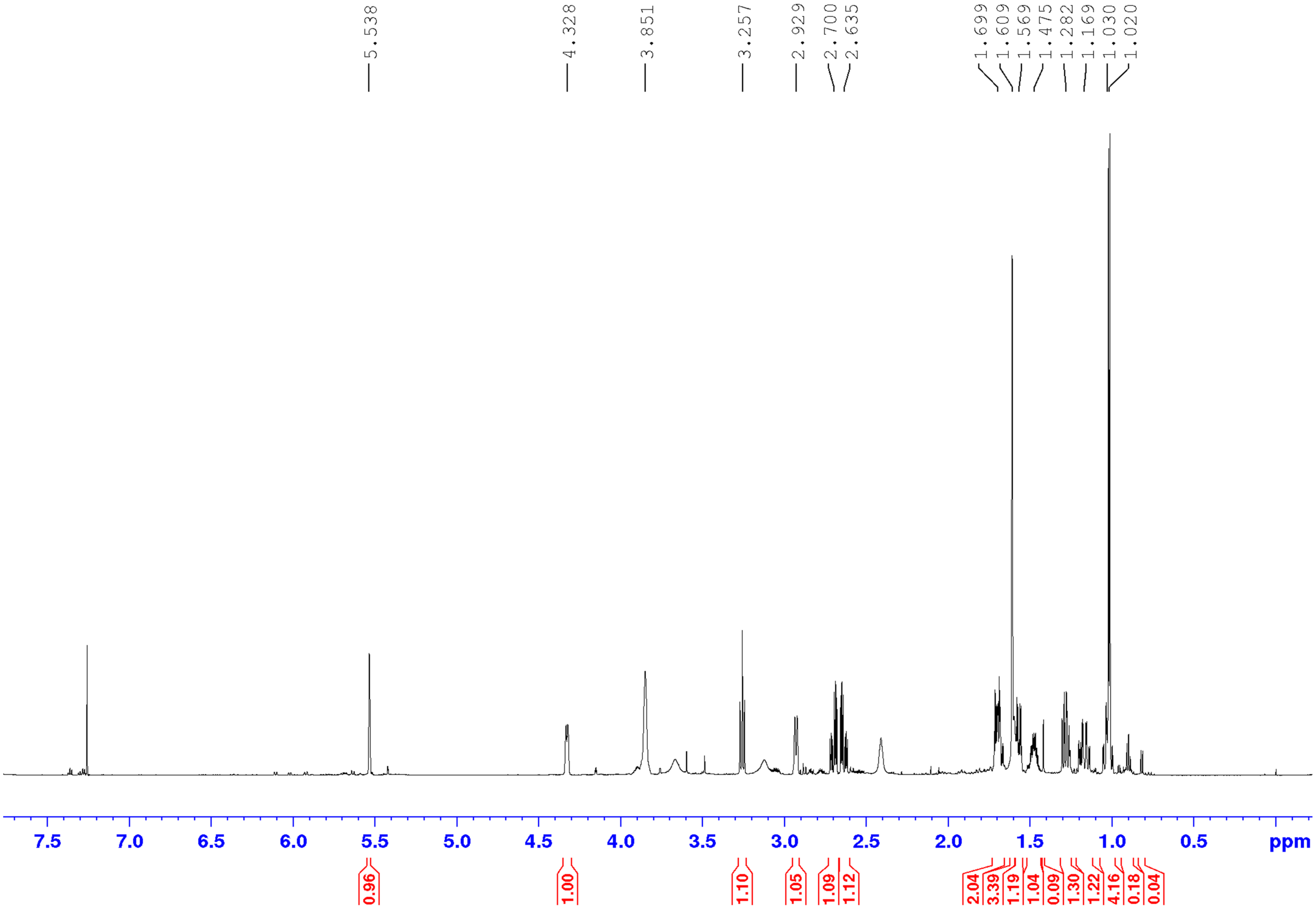

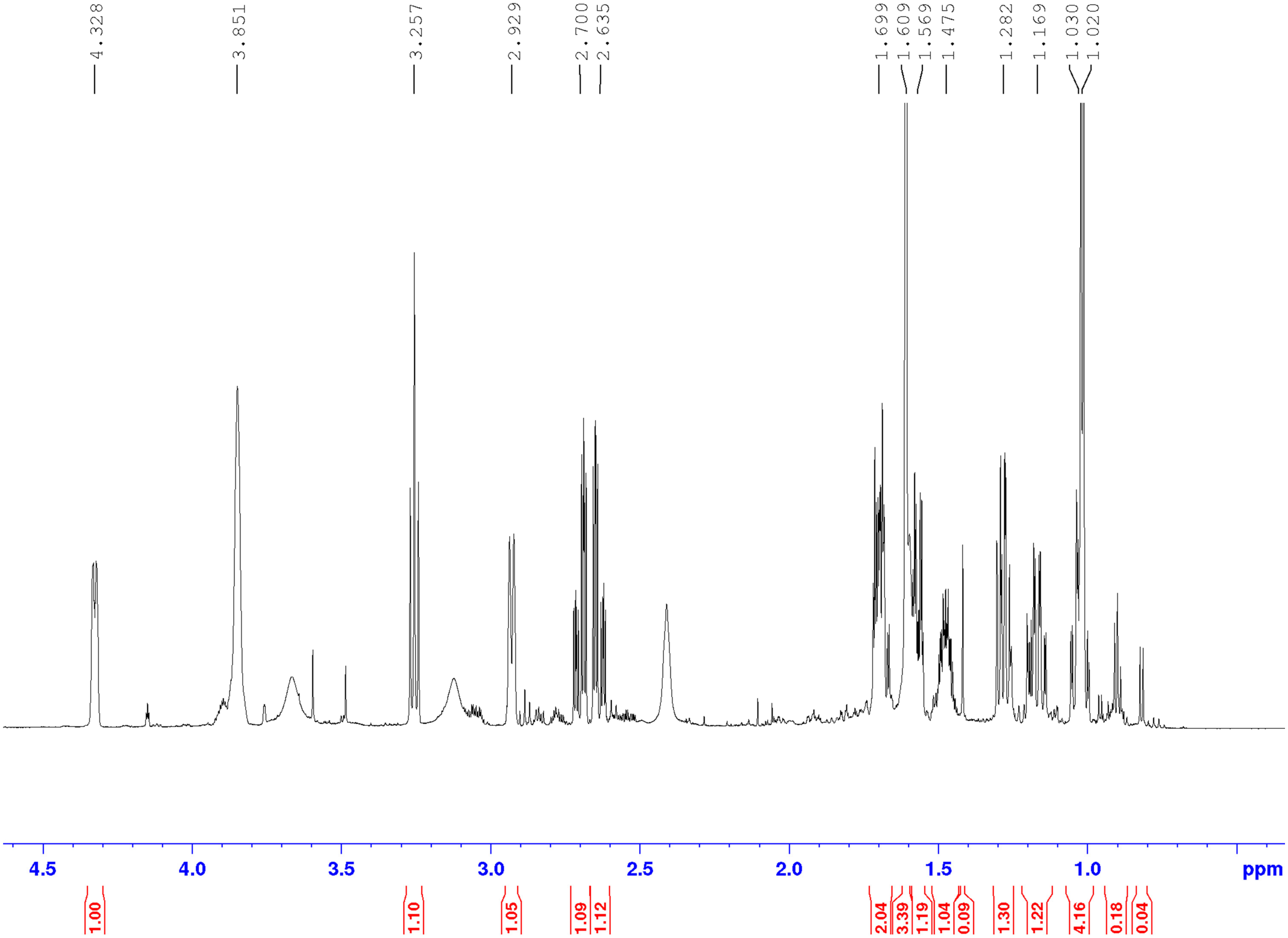

4.328
3.851
3.257
2.929
2.700
2.635
1.699
1.609
1.569
1.475
1.282
1.169
1.030
1.020
4.5
4.0
3.5
3.0
2.5
2.0
1.5
1.0
ppm
1.00
1.10
1.05
1.09
1.12
2.04
3.39
1.19
1.04
0.09
1.30
1.22
4.16
0.18
0.04

Figure S79. ^{13}C NMR spectrum (176.04 MHz) of **8** in $CDCl_3$

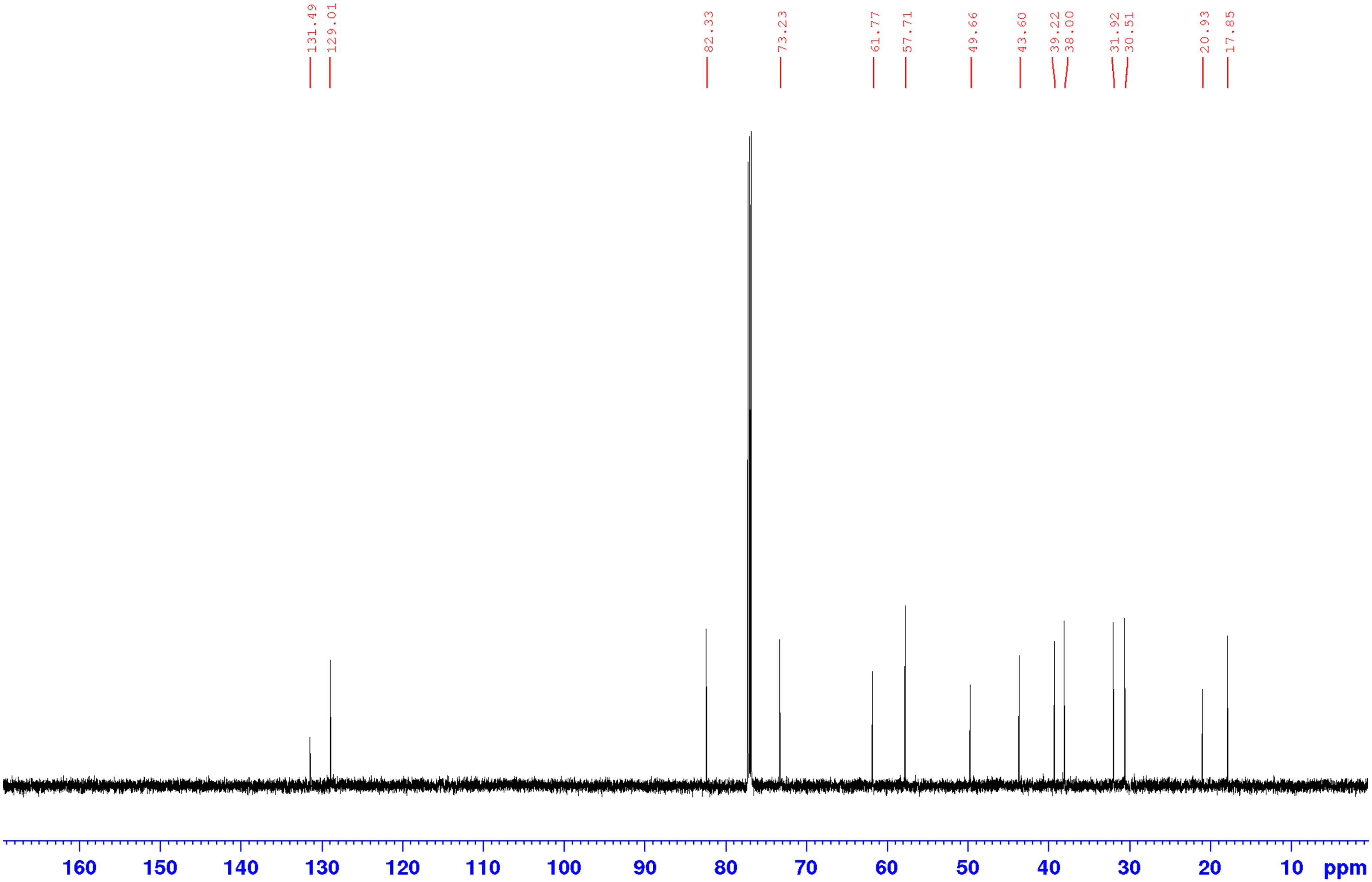

Figure S80. DEPT-135 spectrum (176.04 MHz) of **8** in $CDCl_3$

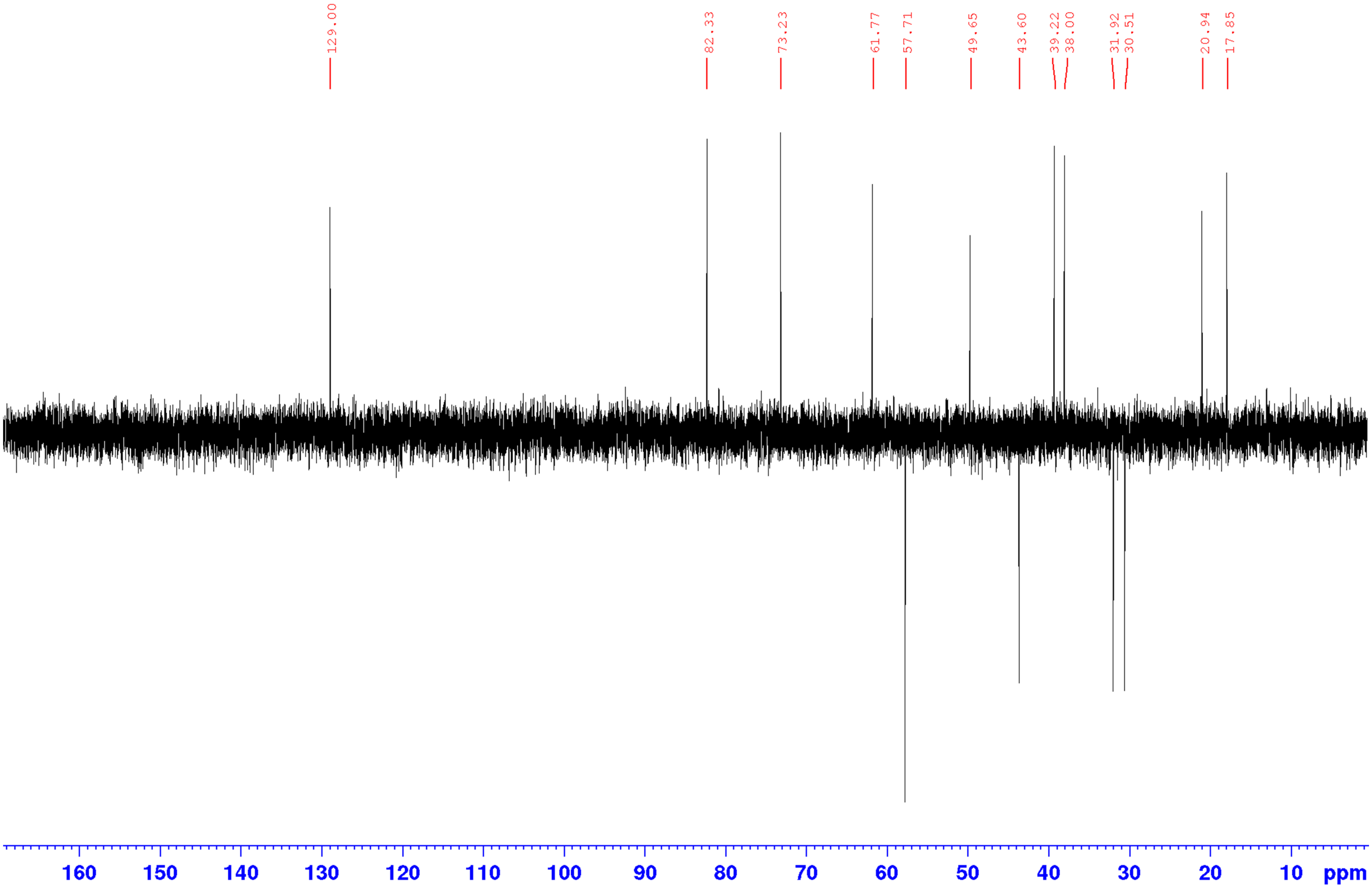

Figure S81. DEPT-90 spectrum (176.04) of **8** in $CDCl_3$

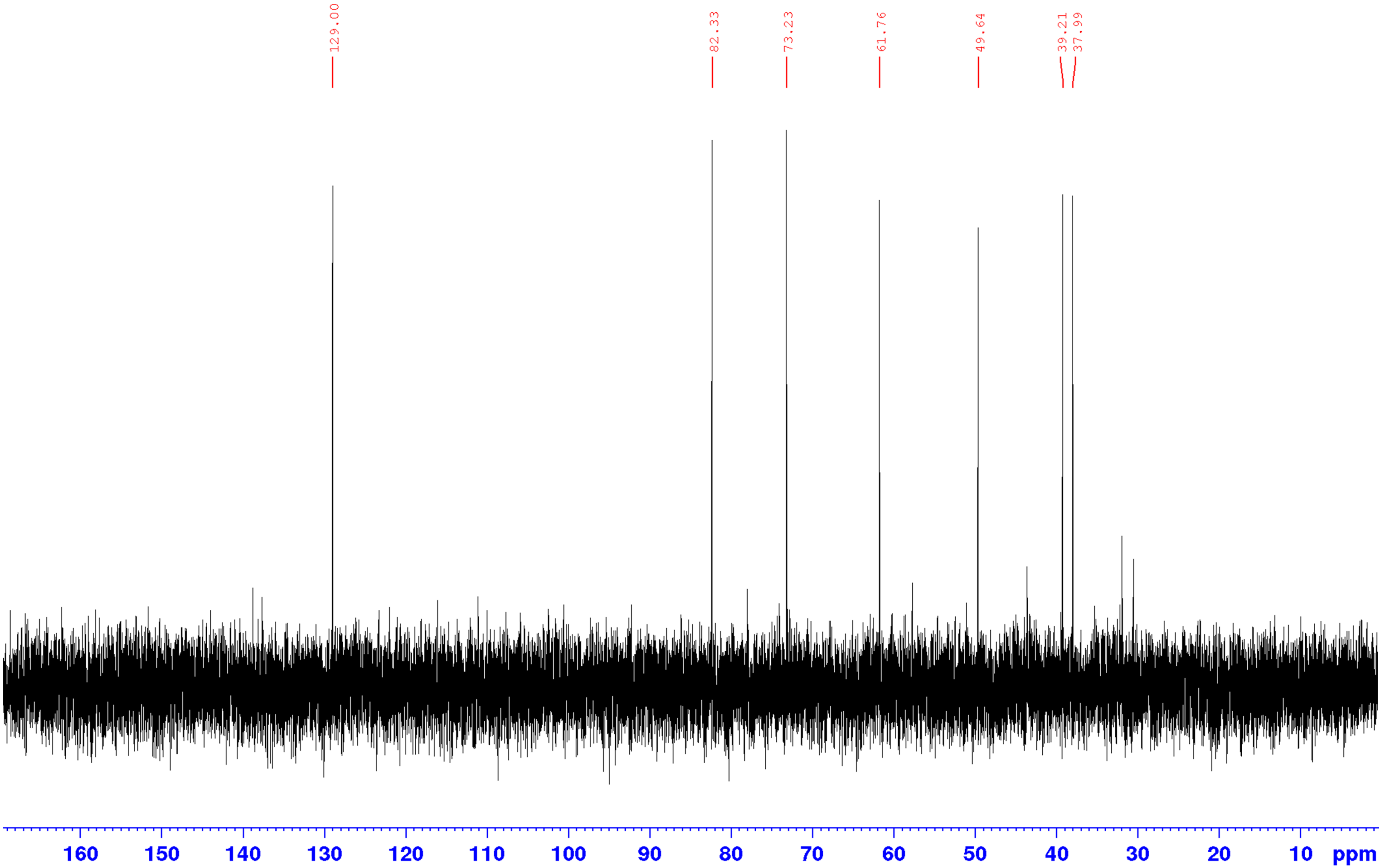

Figure S82. HSQC spectrum of **8** in $CDCl_3$

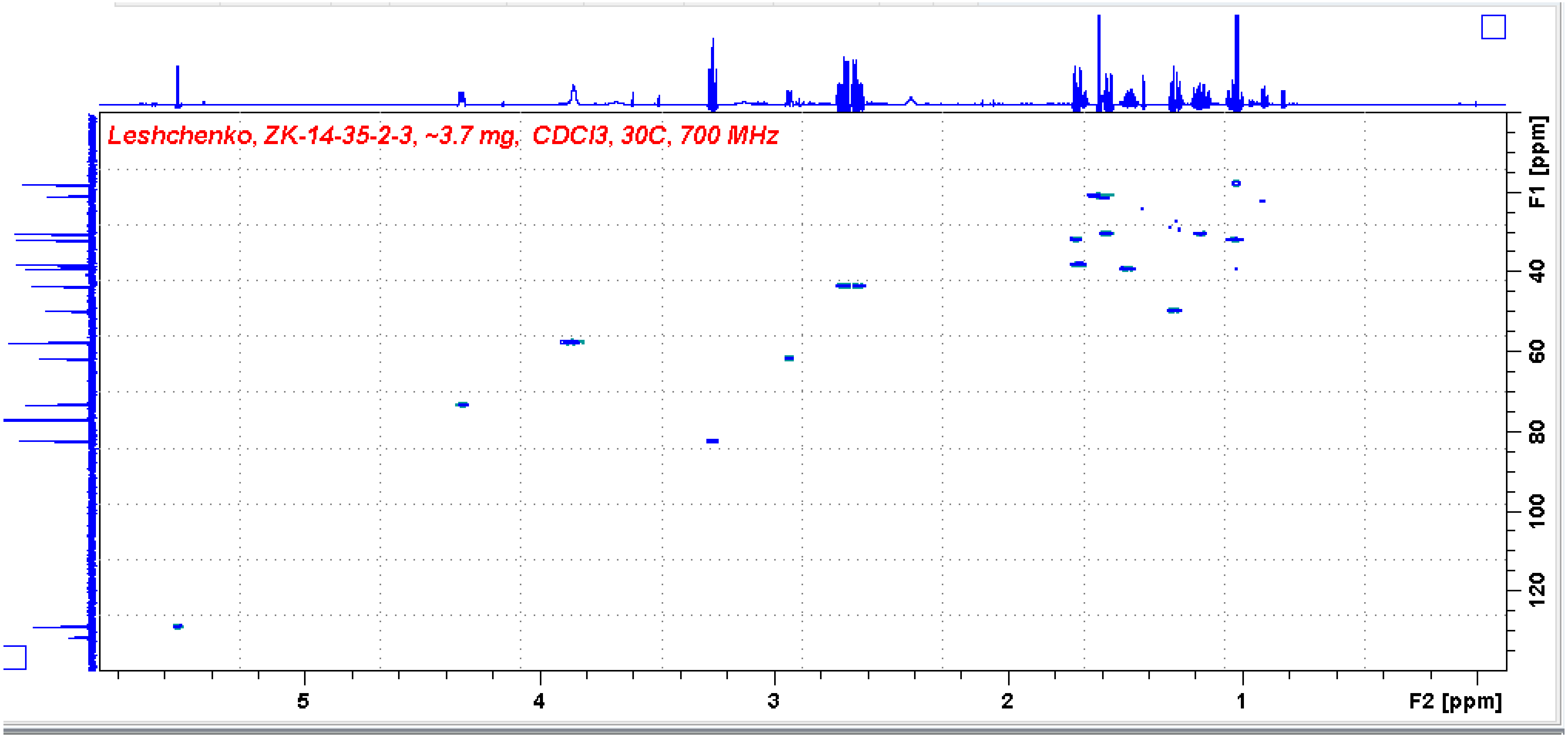

Figure S83. HMBC spectrum of **8** in $CDCl_3$

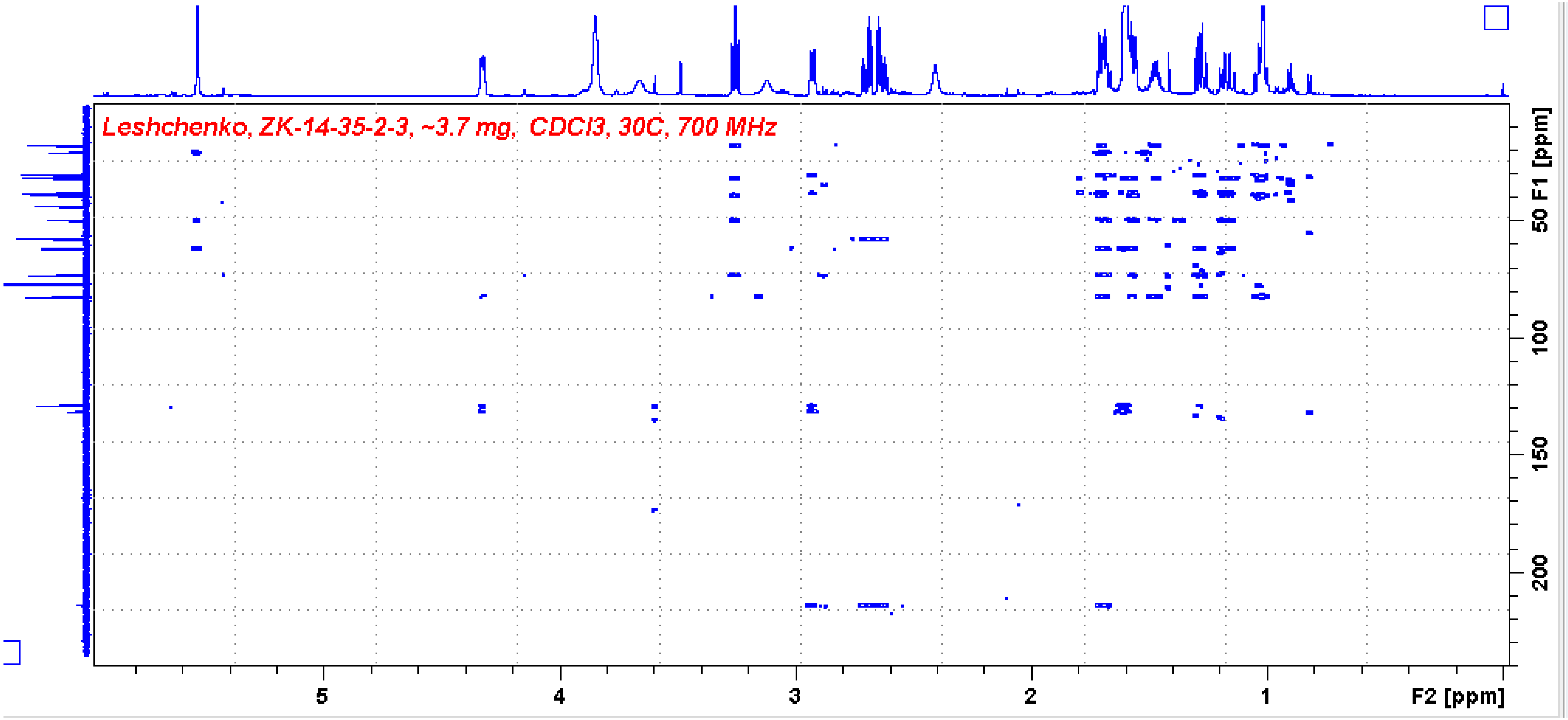

Figure S84. 1H-1H COSY spectrum of **8** in $CDCl_3$

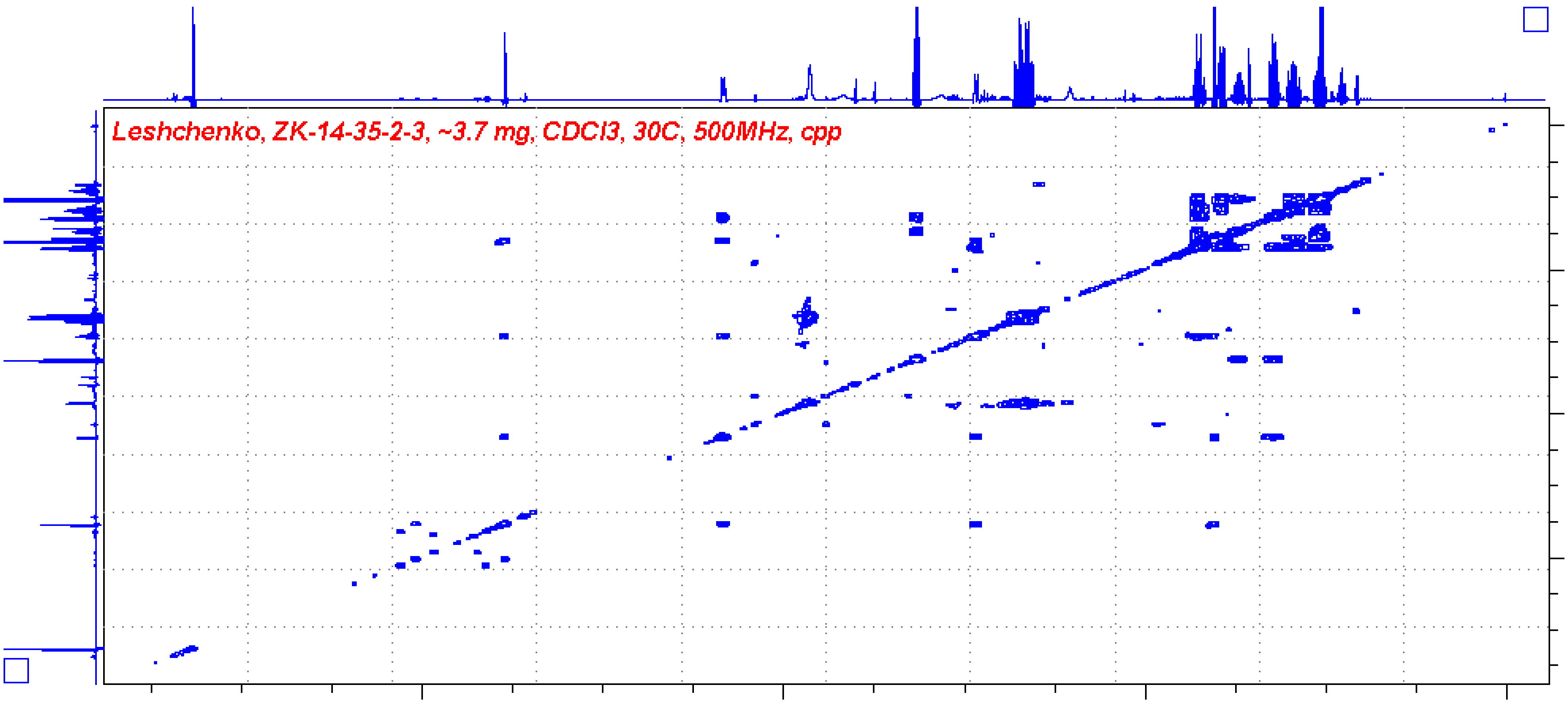

Figure S85. NOESY spectrum of **8** in $CDCl_3$

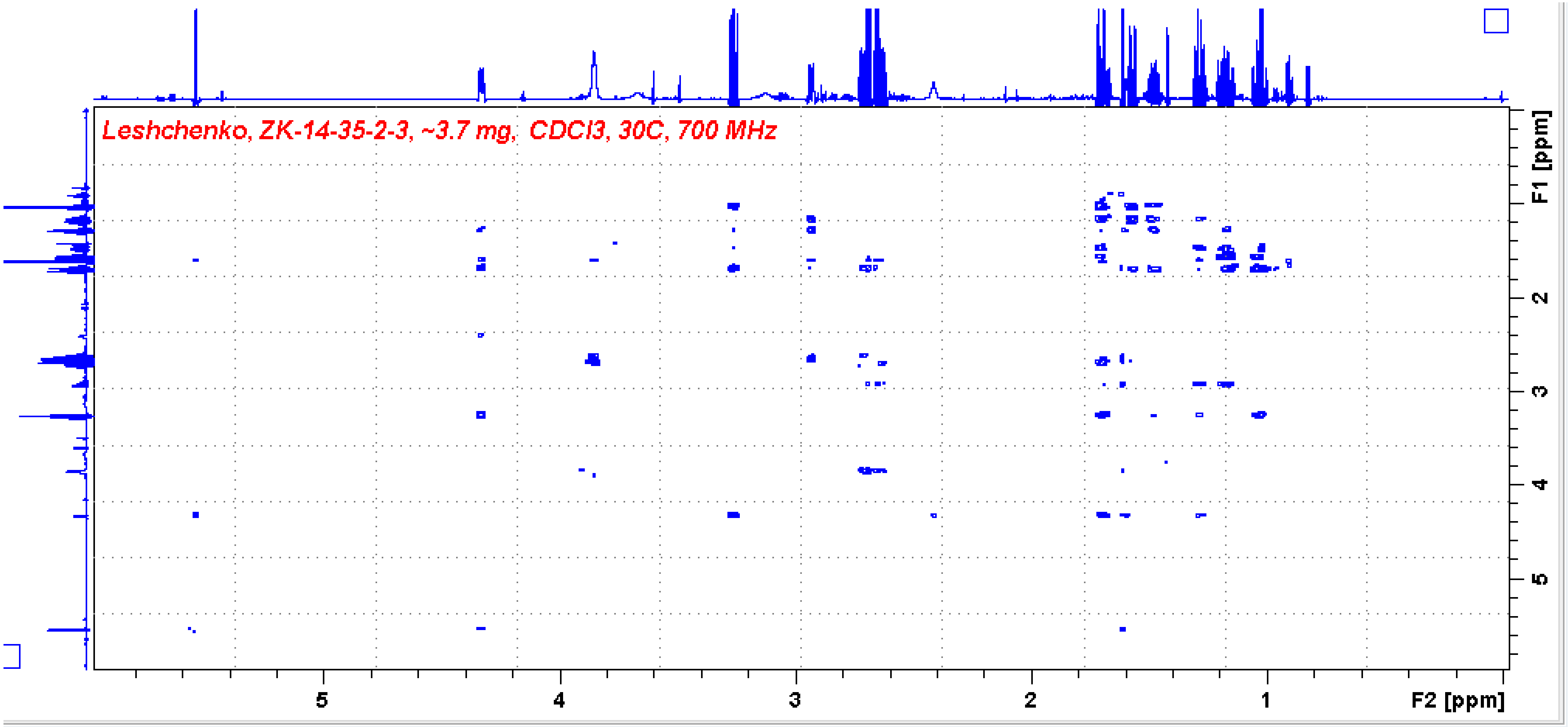

Figure S86. ^{1}H NMR spectrum (125.77 MHz) of **9** in $CDCl_3$

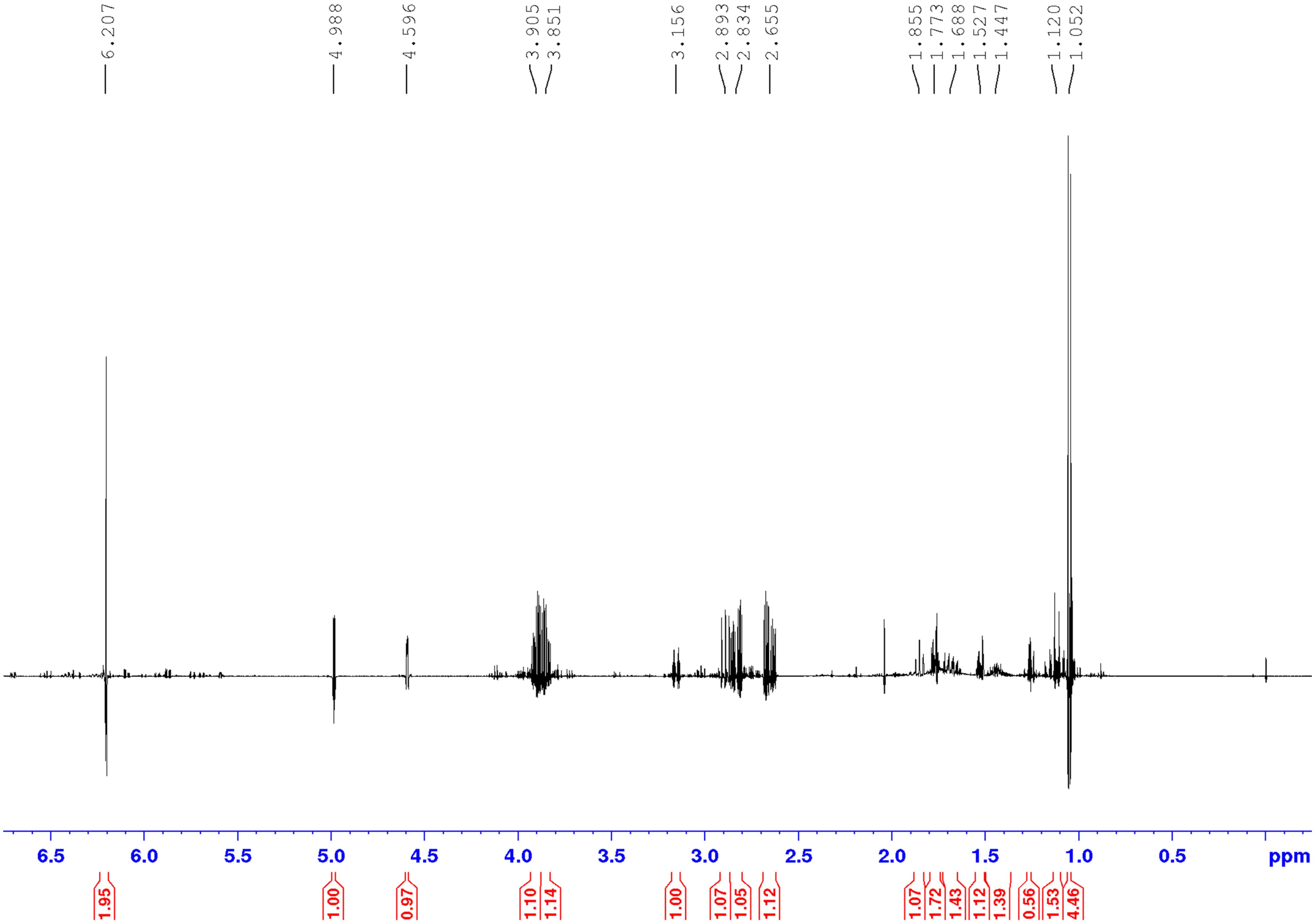

Figure S87. ^{13}C NMR spectrum (125.77 MHz) of **9** in $CDCl_3$

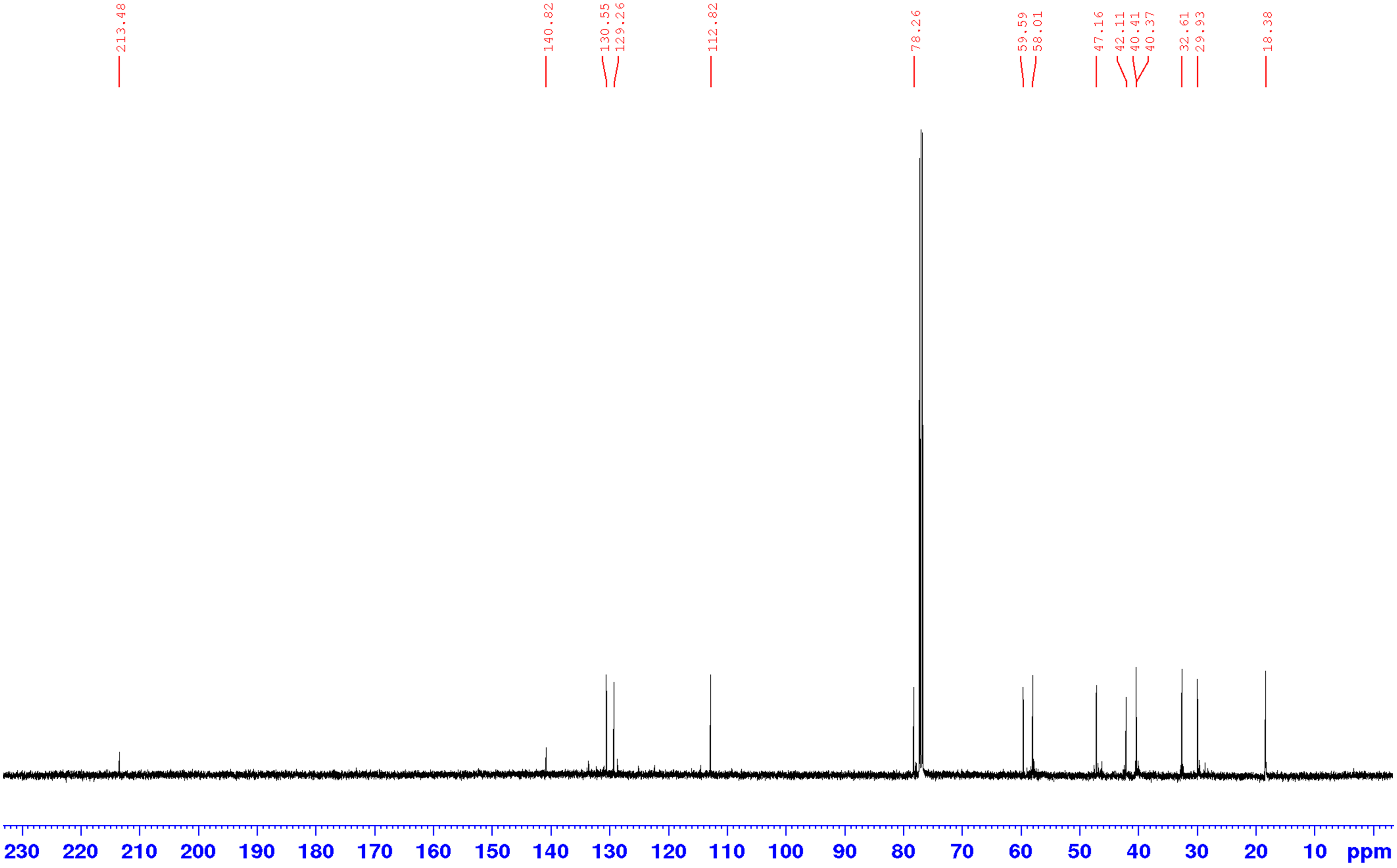

Figure S88. DEPT-135 spectrum (125.77 MHz) of **9** in $CDCl_3$

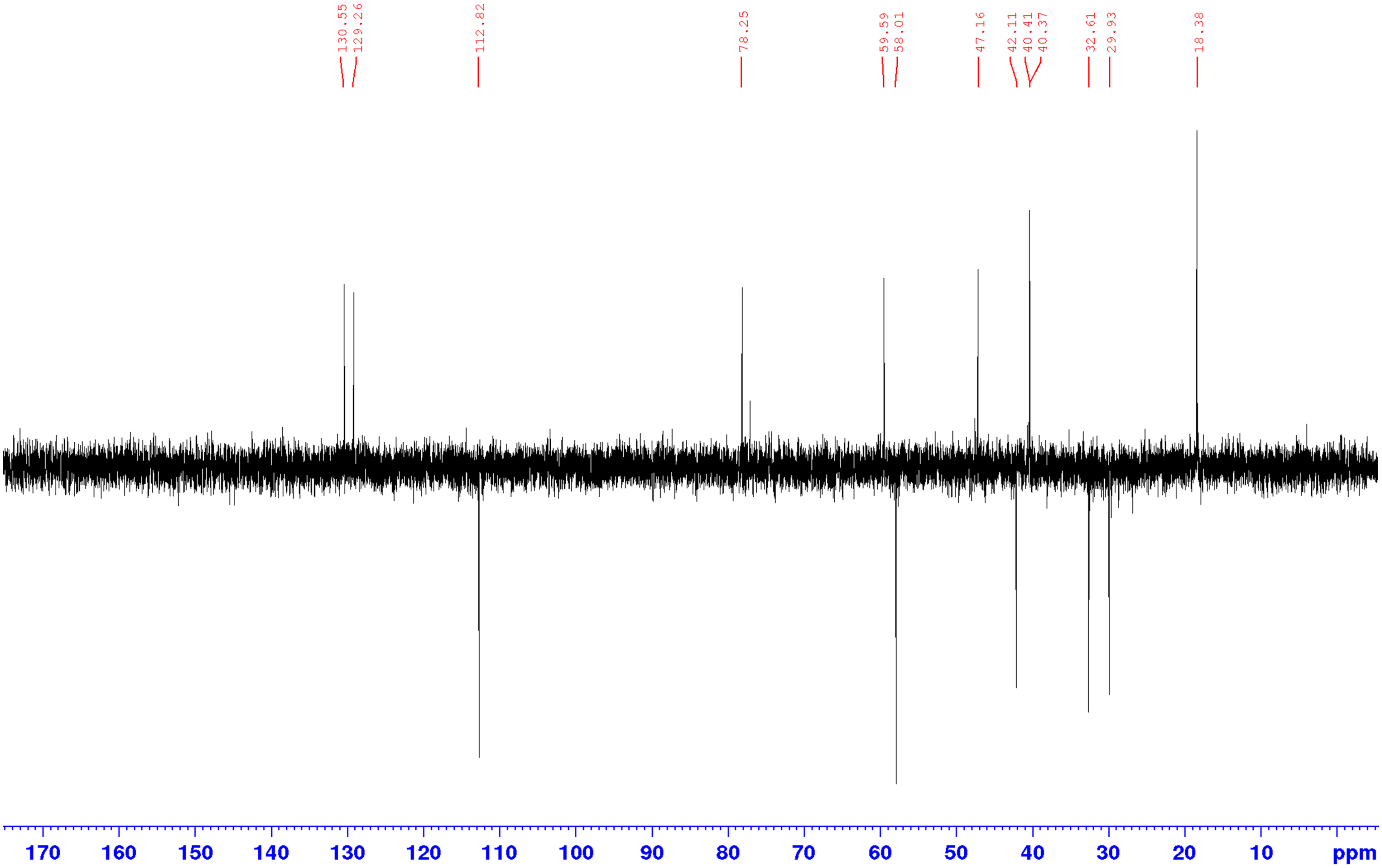

Figure S89. DEPT-90 spectrum (125.77 MHz) of **9** in $CDCl_3$

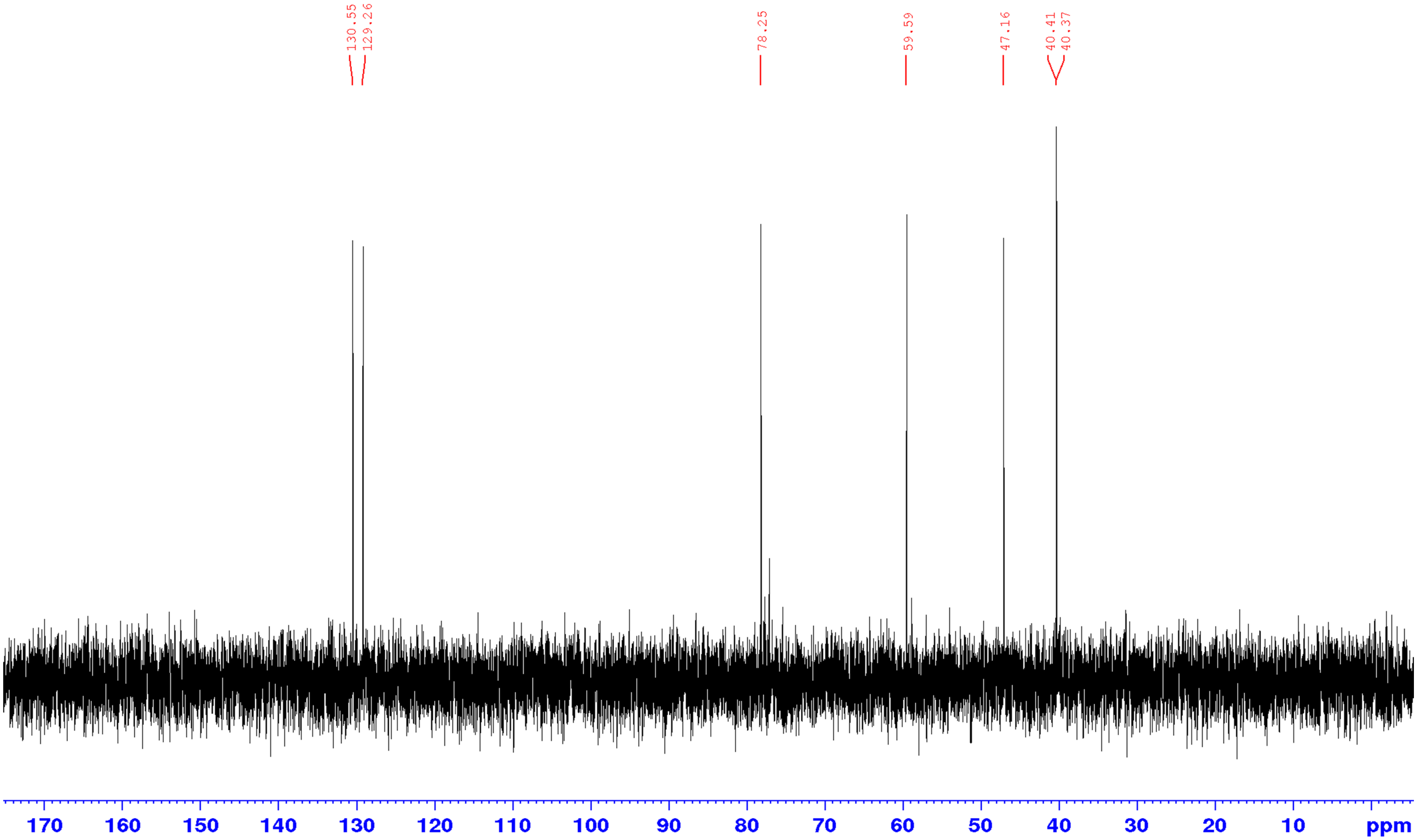

Figure S90. HSQC spectrum of **9** in $CDCl_3$

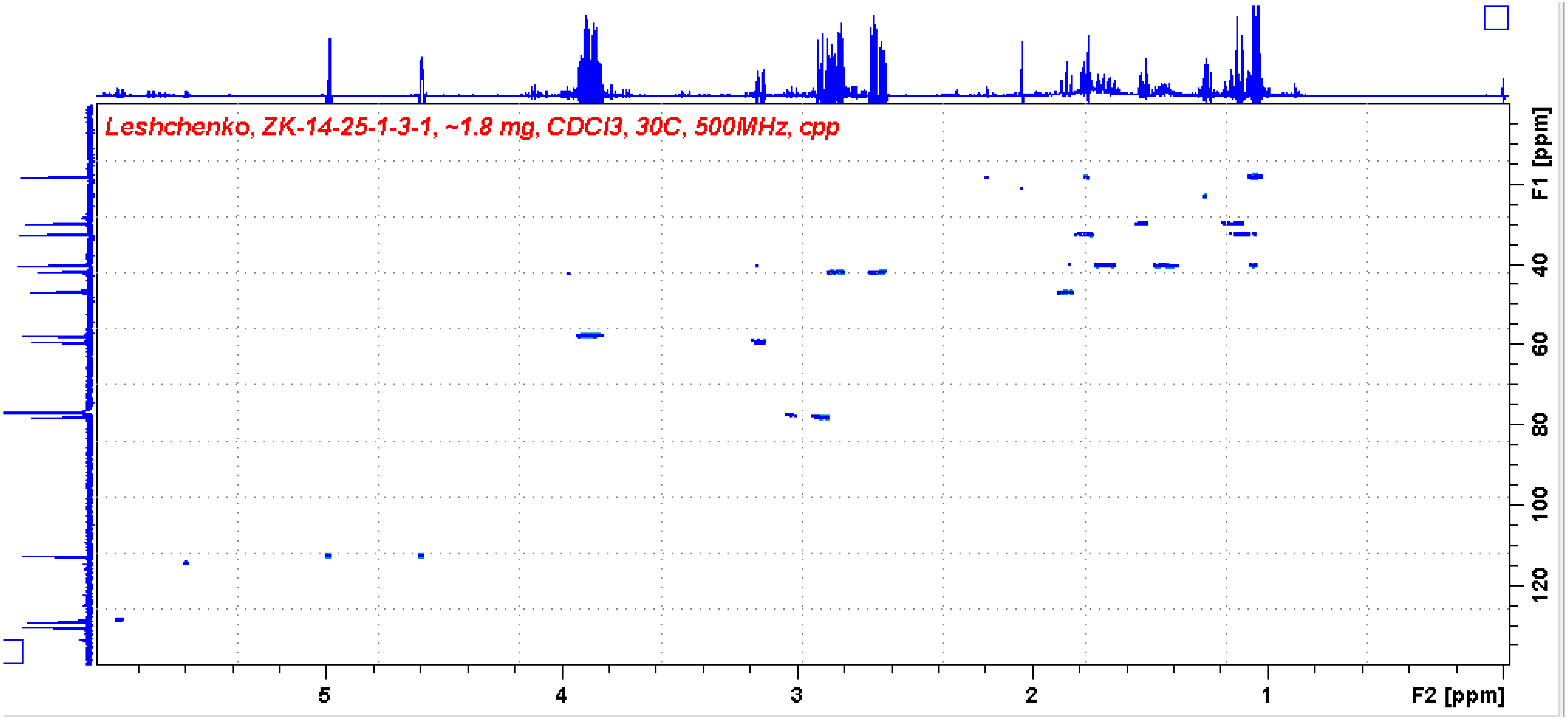

Figure S91. HMBC spectrum of **9** in $CDCl_3$

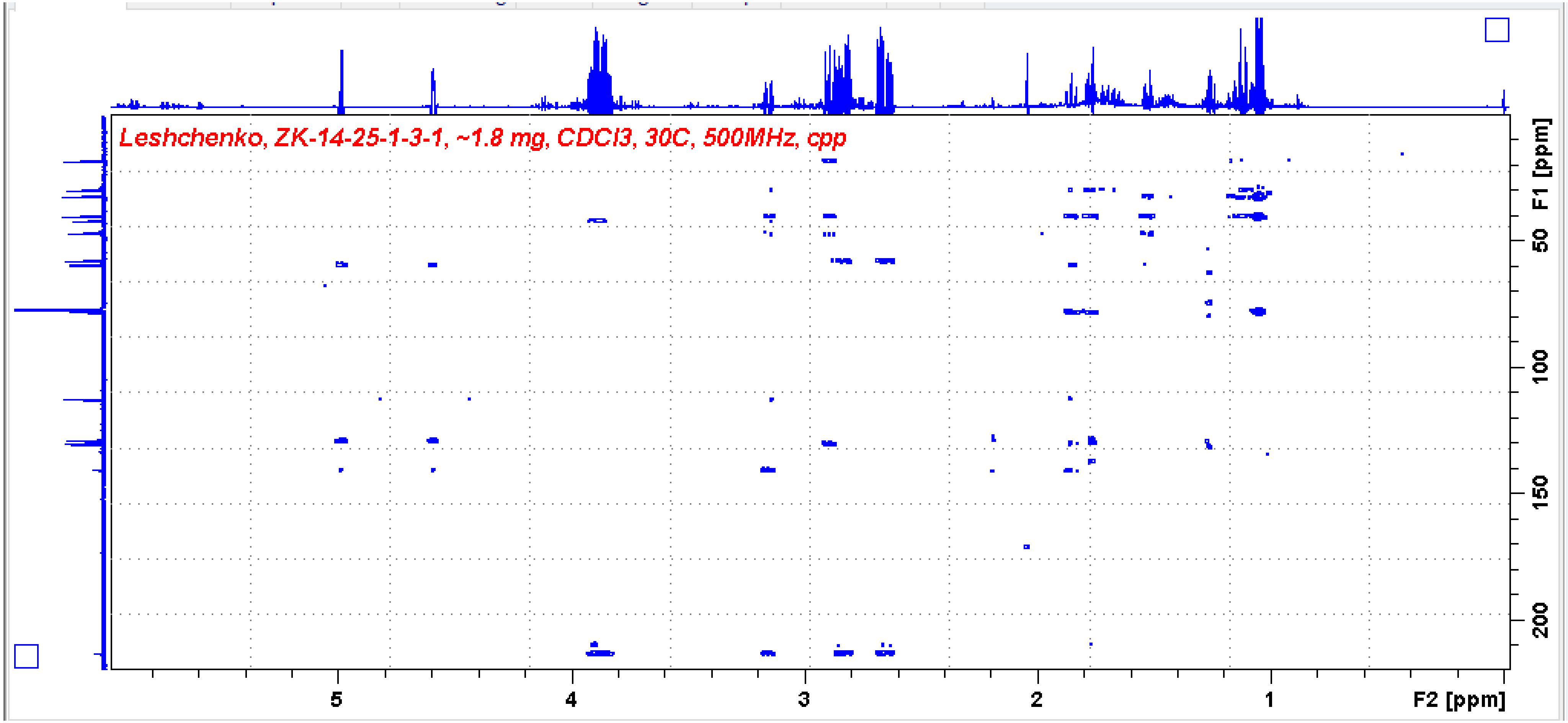

Figure S92. 1H-1H COSY spectrum of **9** in $CDCl_3$

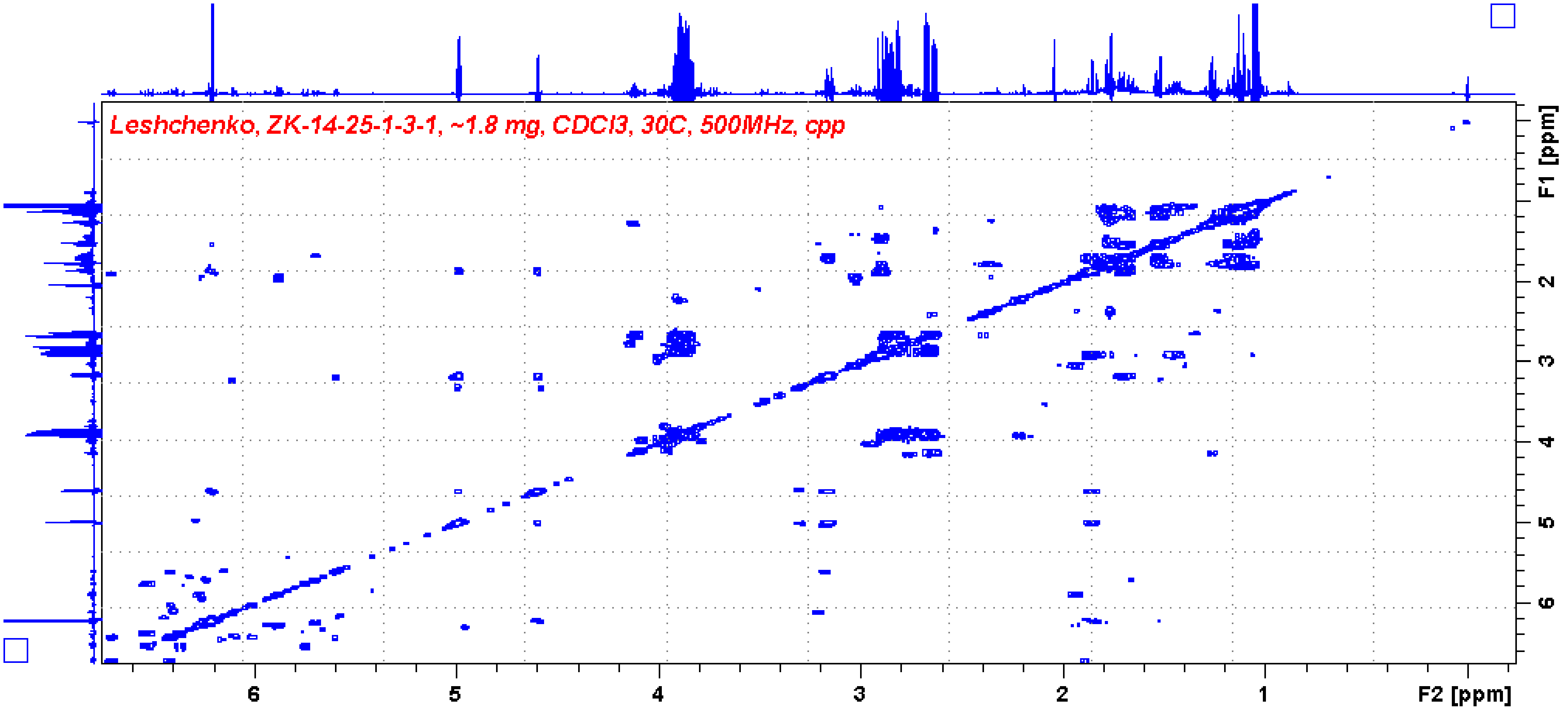

Figure S93. NOESY spectrum of **9** in $CDCl_3$

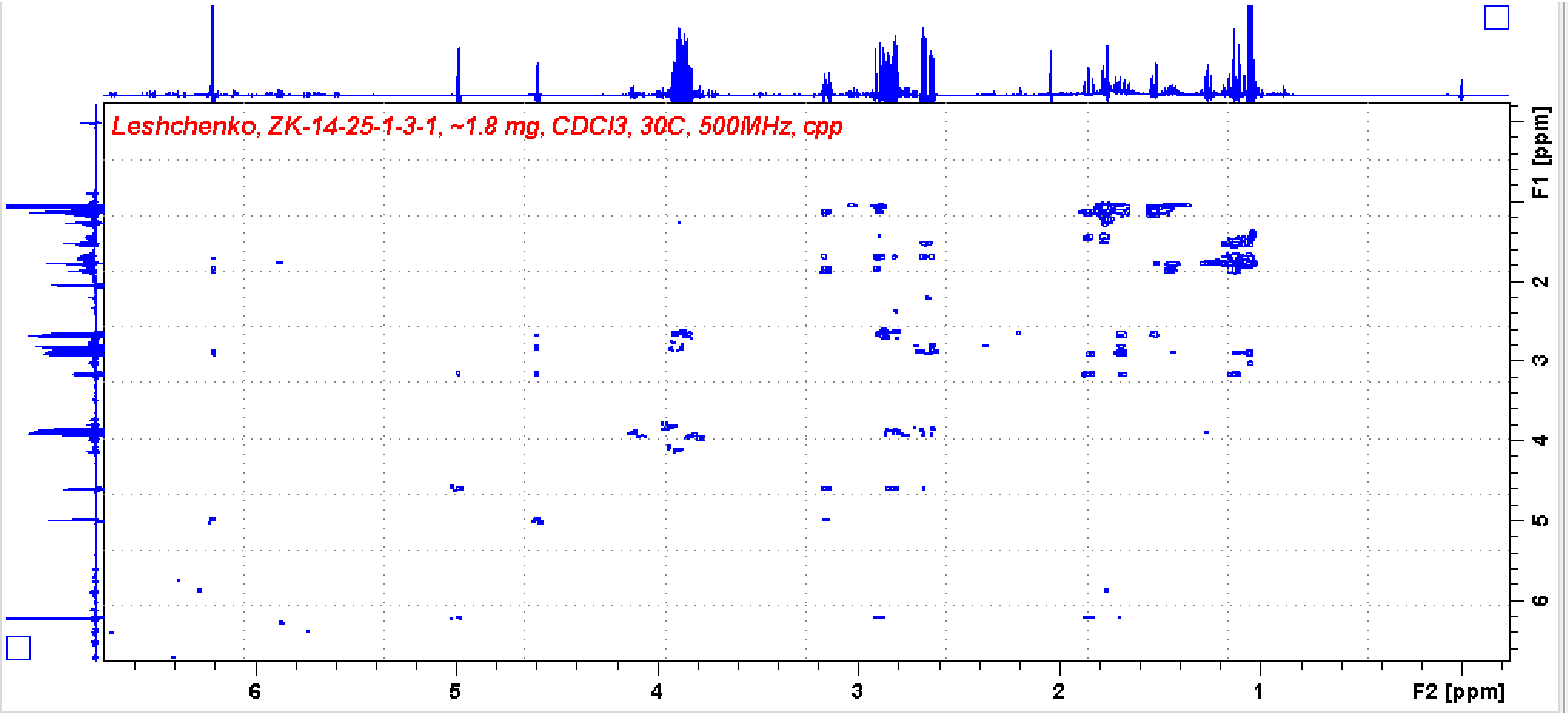

Figure S94. ^{1}H NMR spectrum (700.13 MHz) of **10** in $CDCl_3$

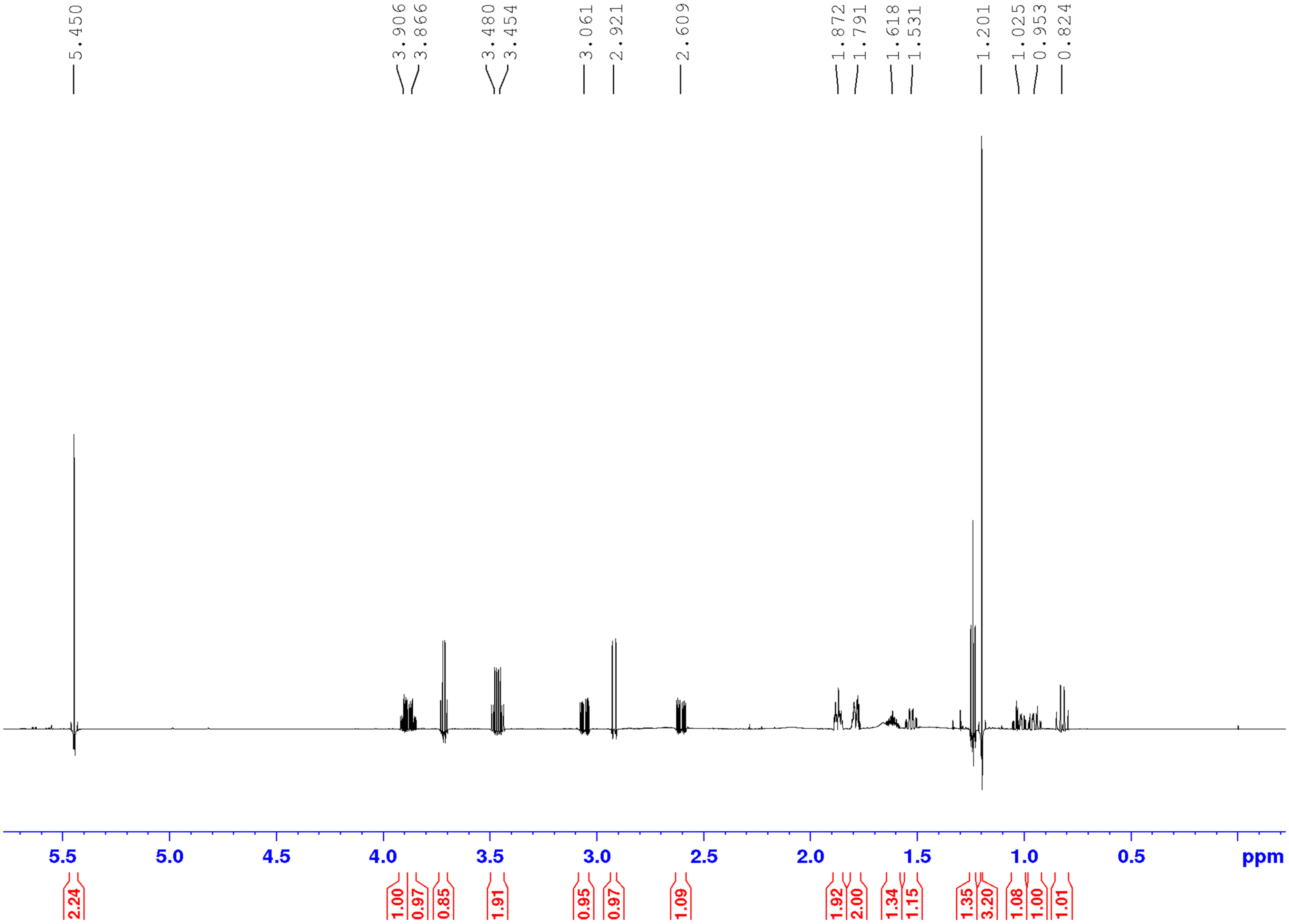

Figure S95. ^{13}C NMR spectrum (176.04 MHz) of **10** in $CDCl_3$

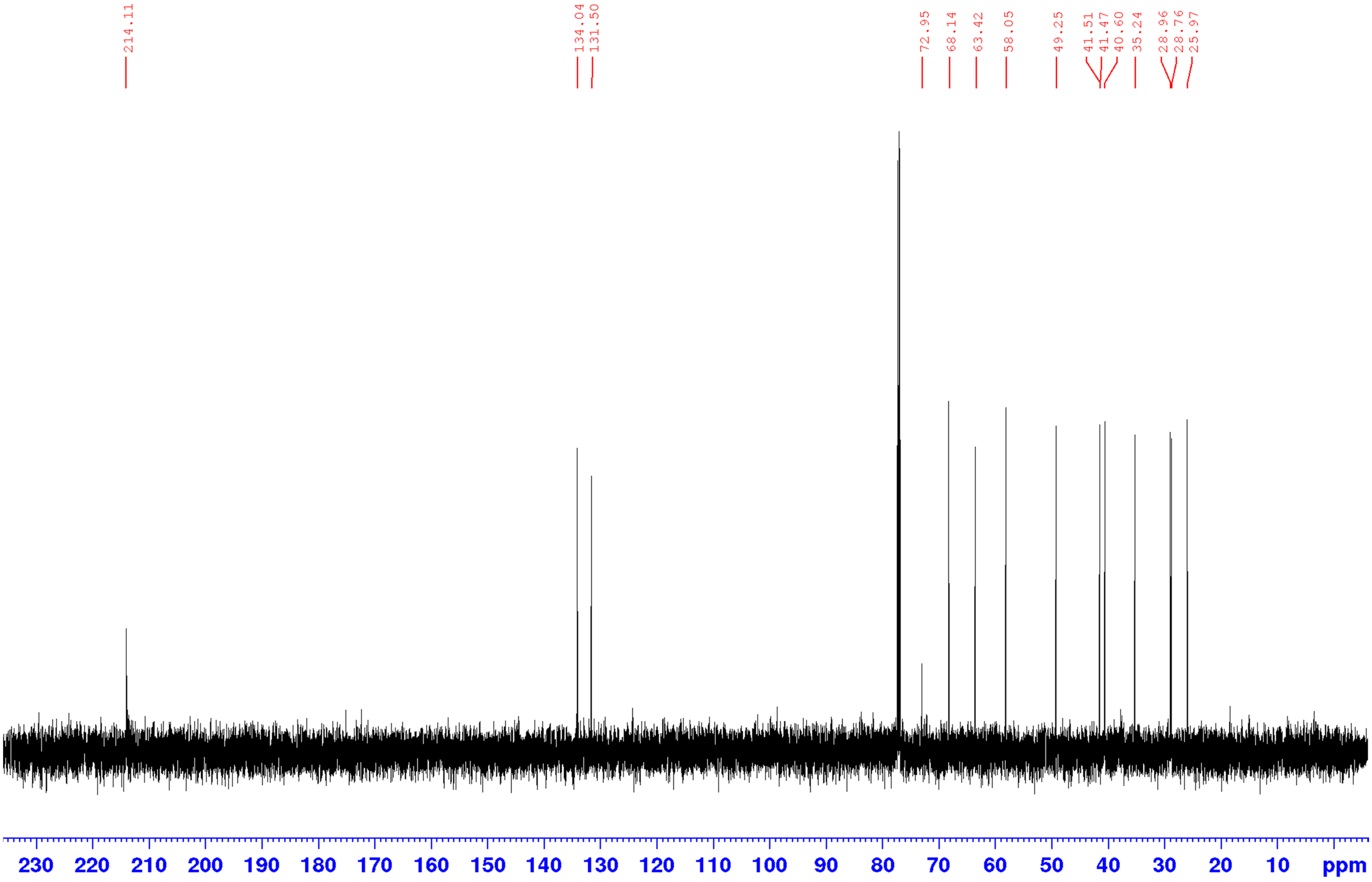

Figure S96. DEPT-135 spectrum (176.04 MHz) of **10** in $CDCl_3$

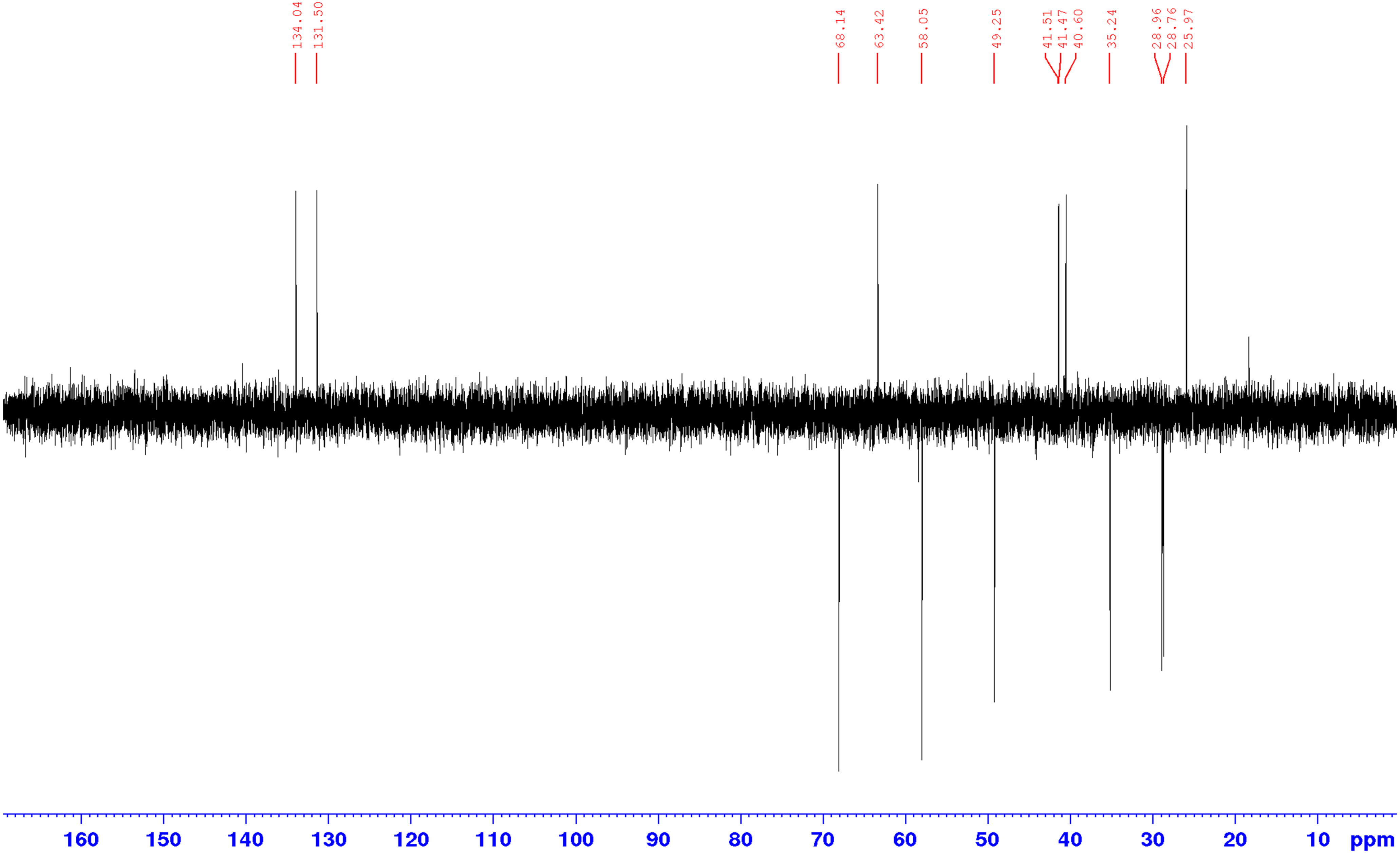

Figure S97. DEPT-90 spectrum (176.04) of **10** in $CDCl_3$

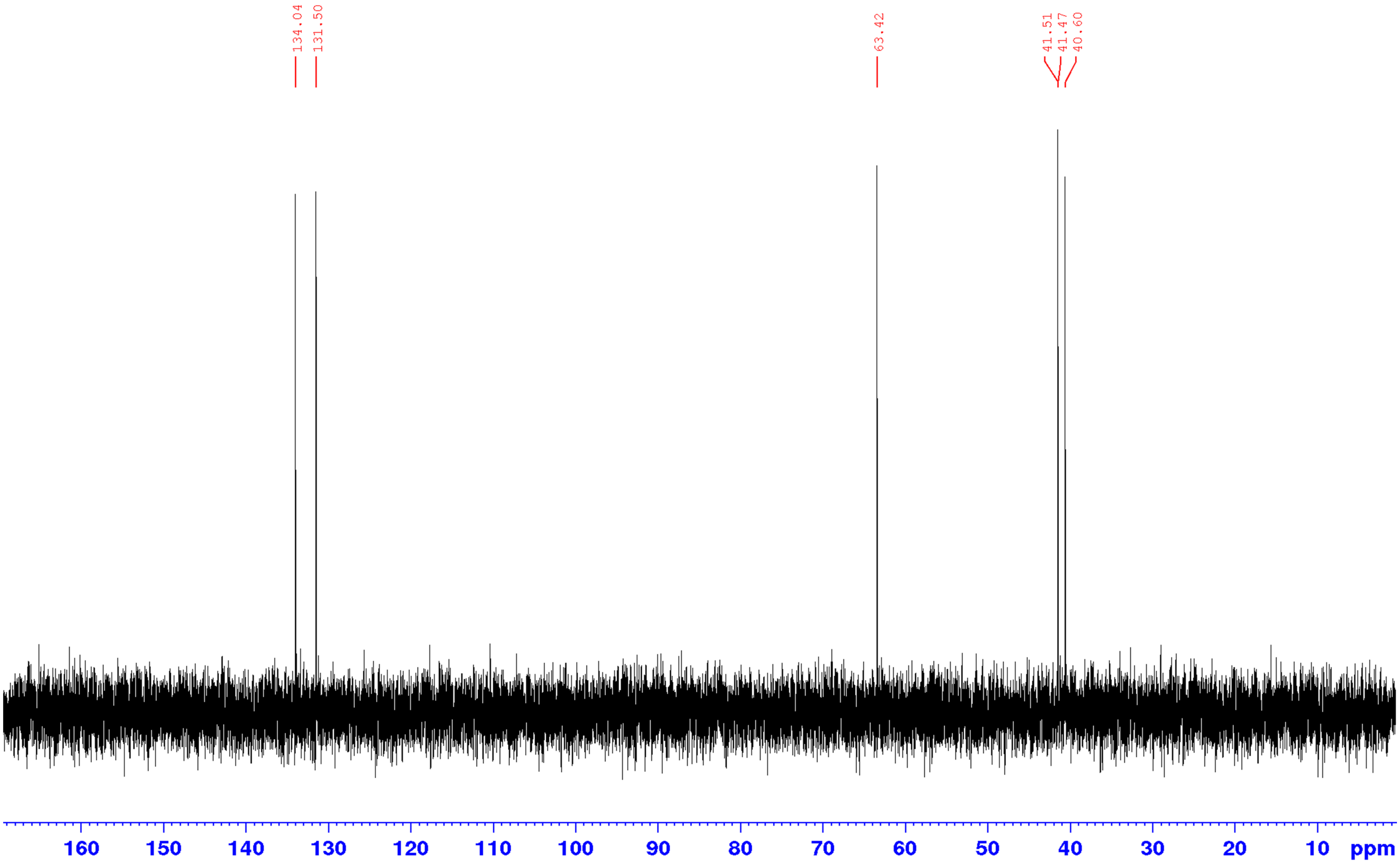

Figure S98. HSQC spectrum of **10** in $CDCl_3$

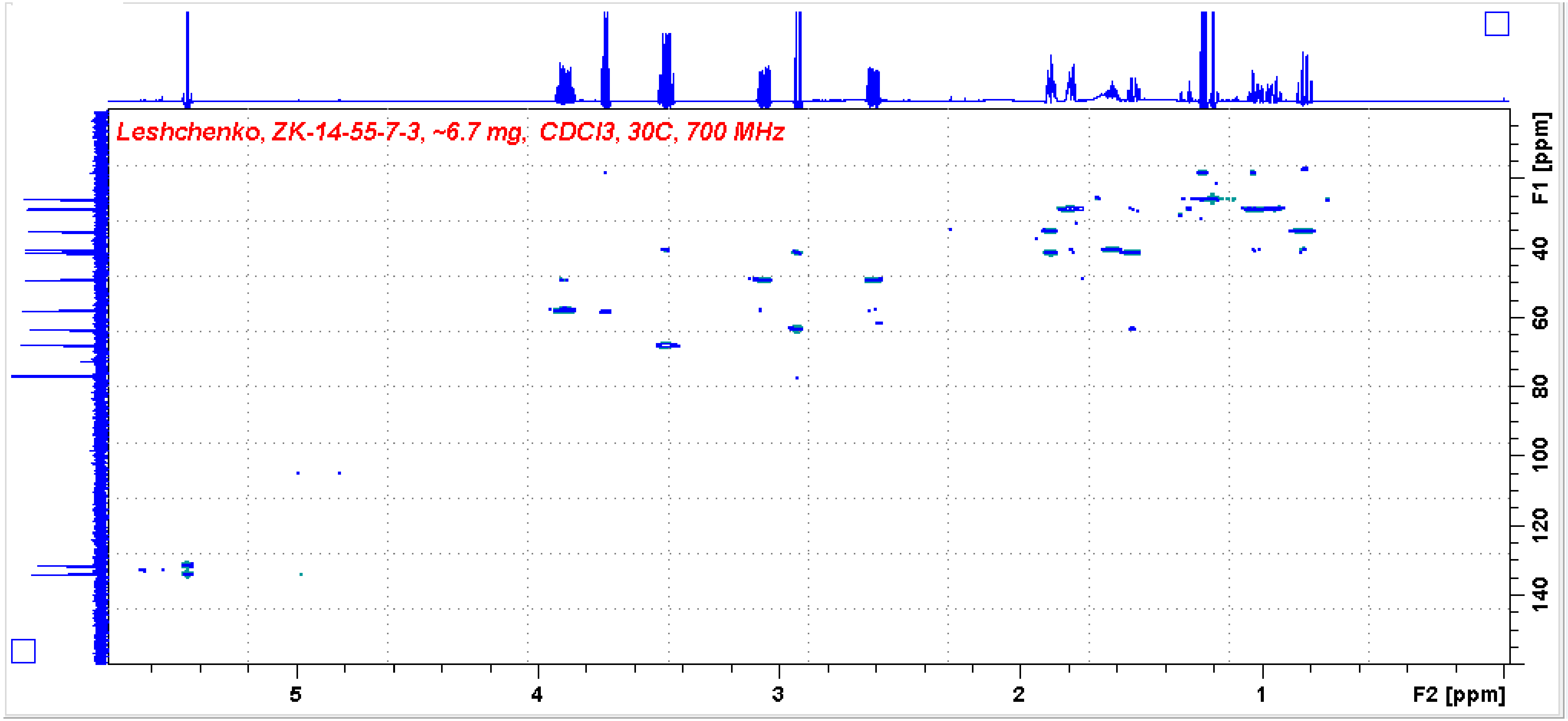

Figure S99. HMBC spectrum of **10** in $CDCl_3$

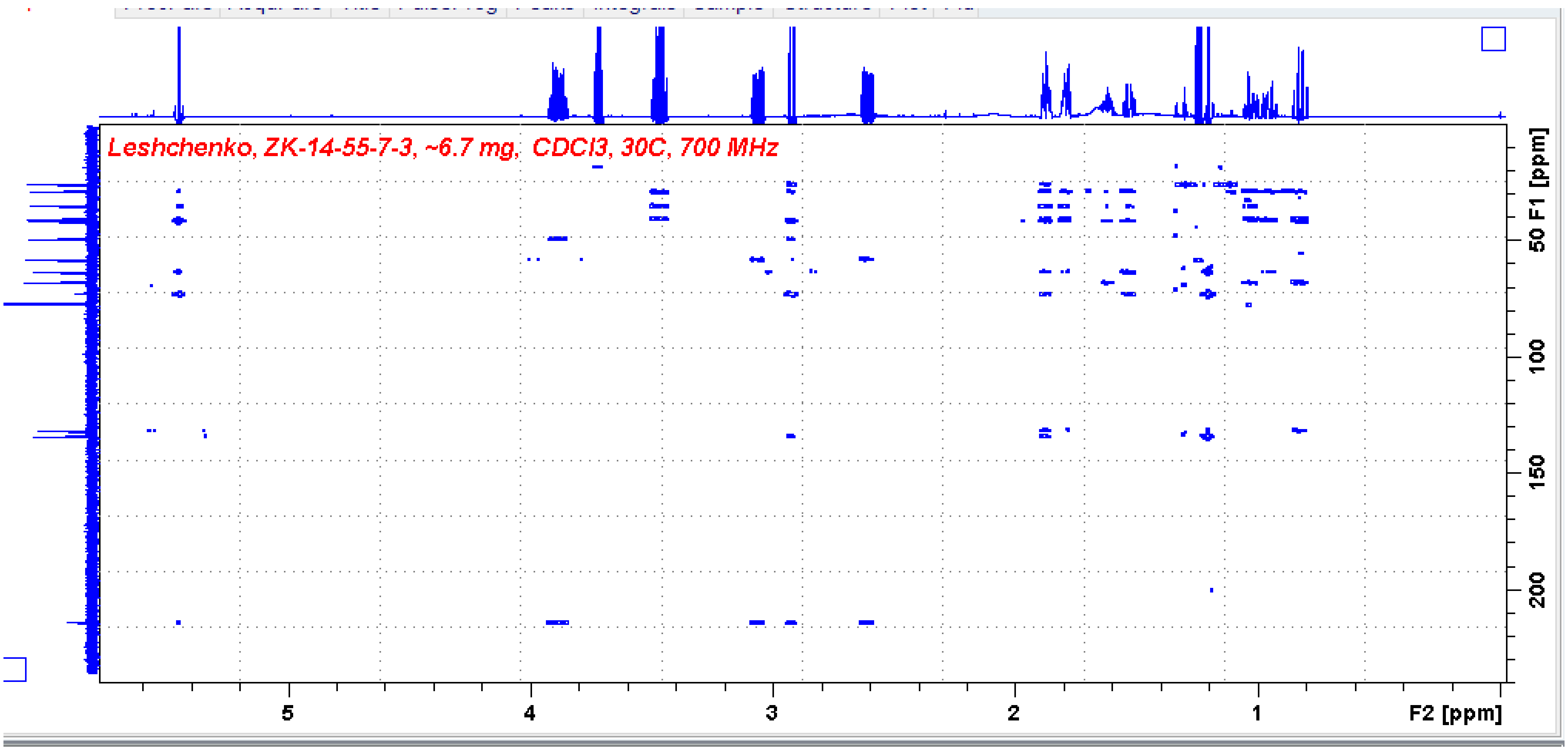

Figure S100. 1H-1H COSY spectrum of **10** in $CDCl_3$

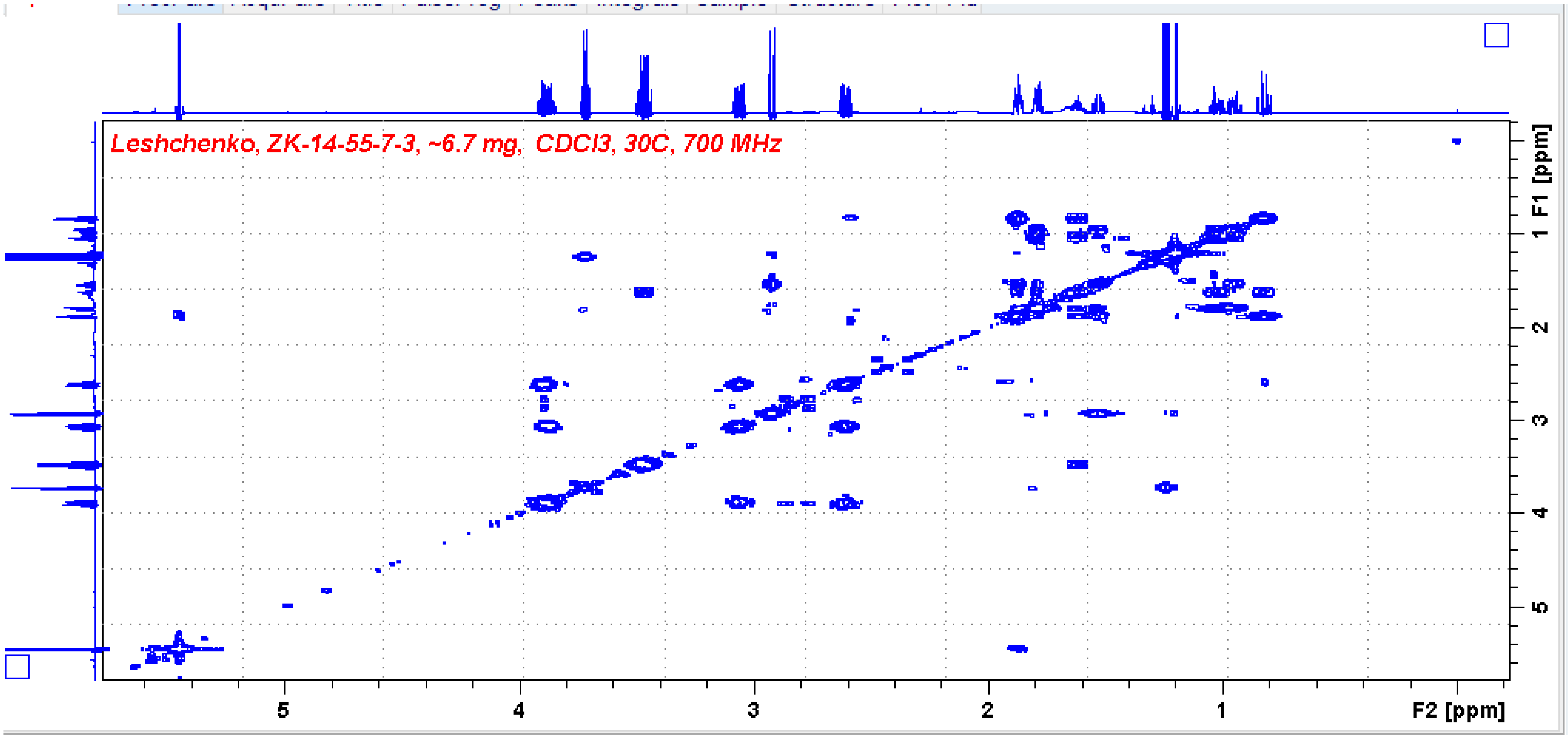

Figure S101. NOESY spectrum of **10** in $CDCl_3$

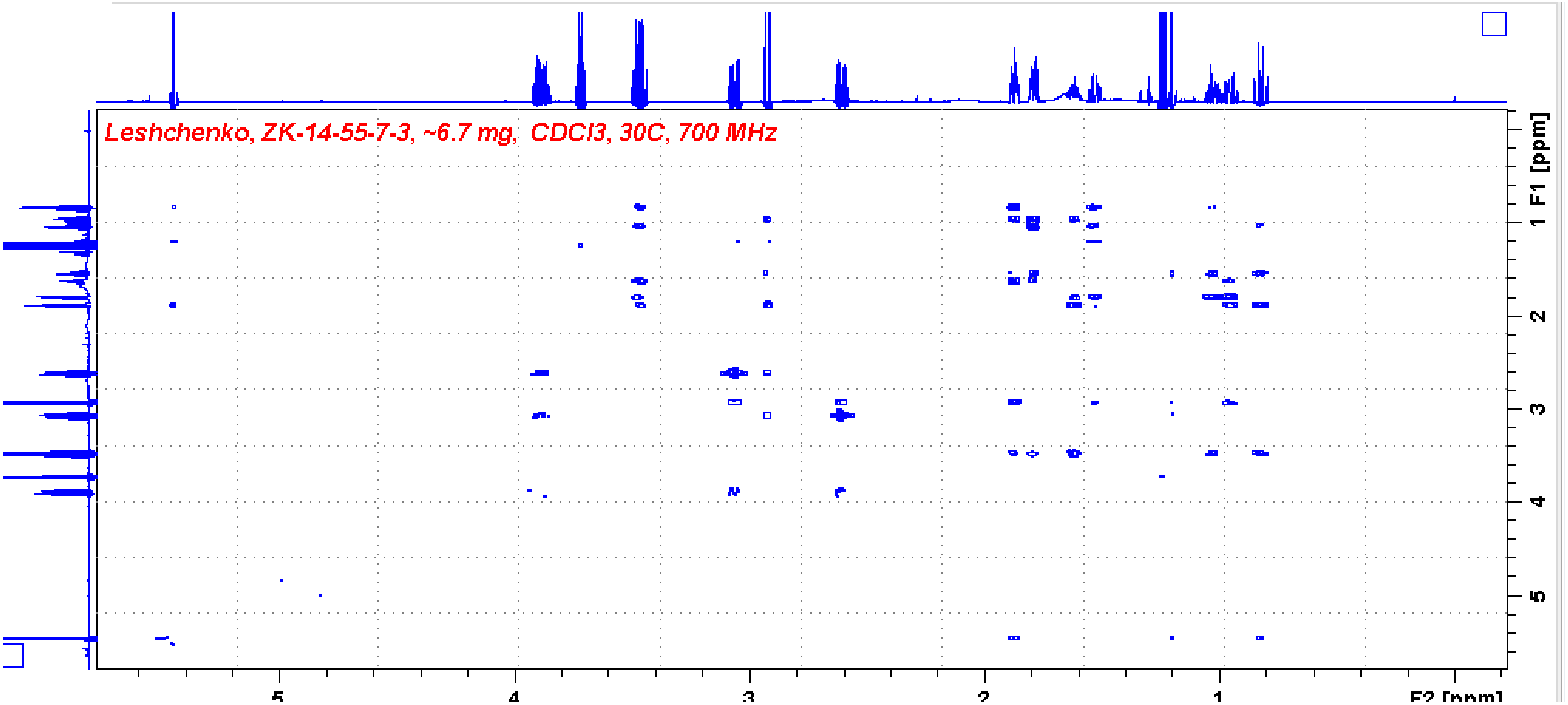

Figure S102. ^{1}H NMR spectrum (500.13 MHz) of **11** in $CDCl_3$

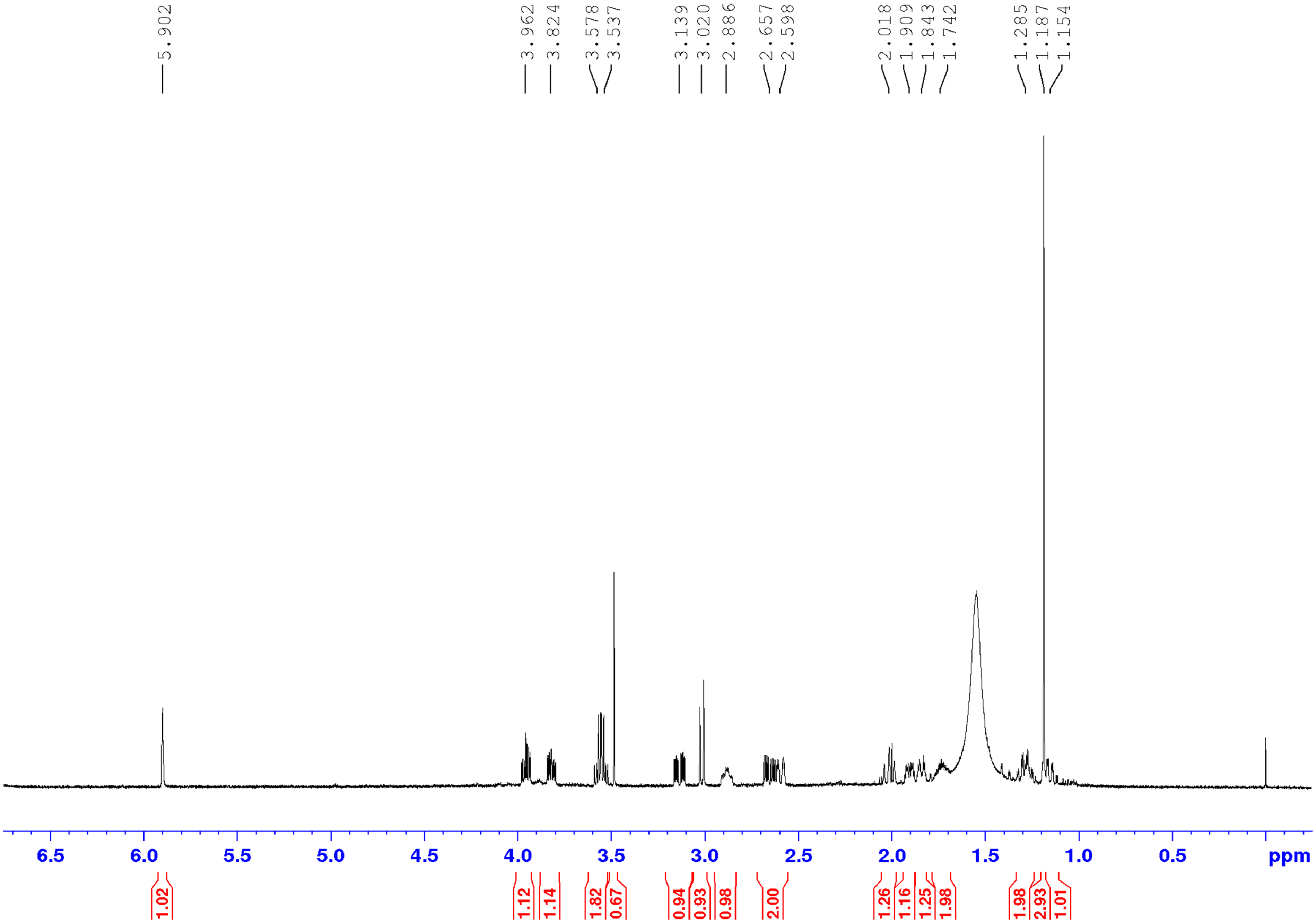

Figure S103. ^{13}C NMR spectrum (125.77 MHz) of **11** in $CDCl_3$

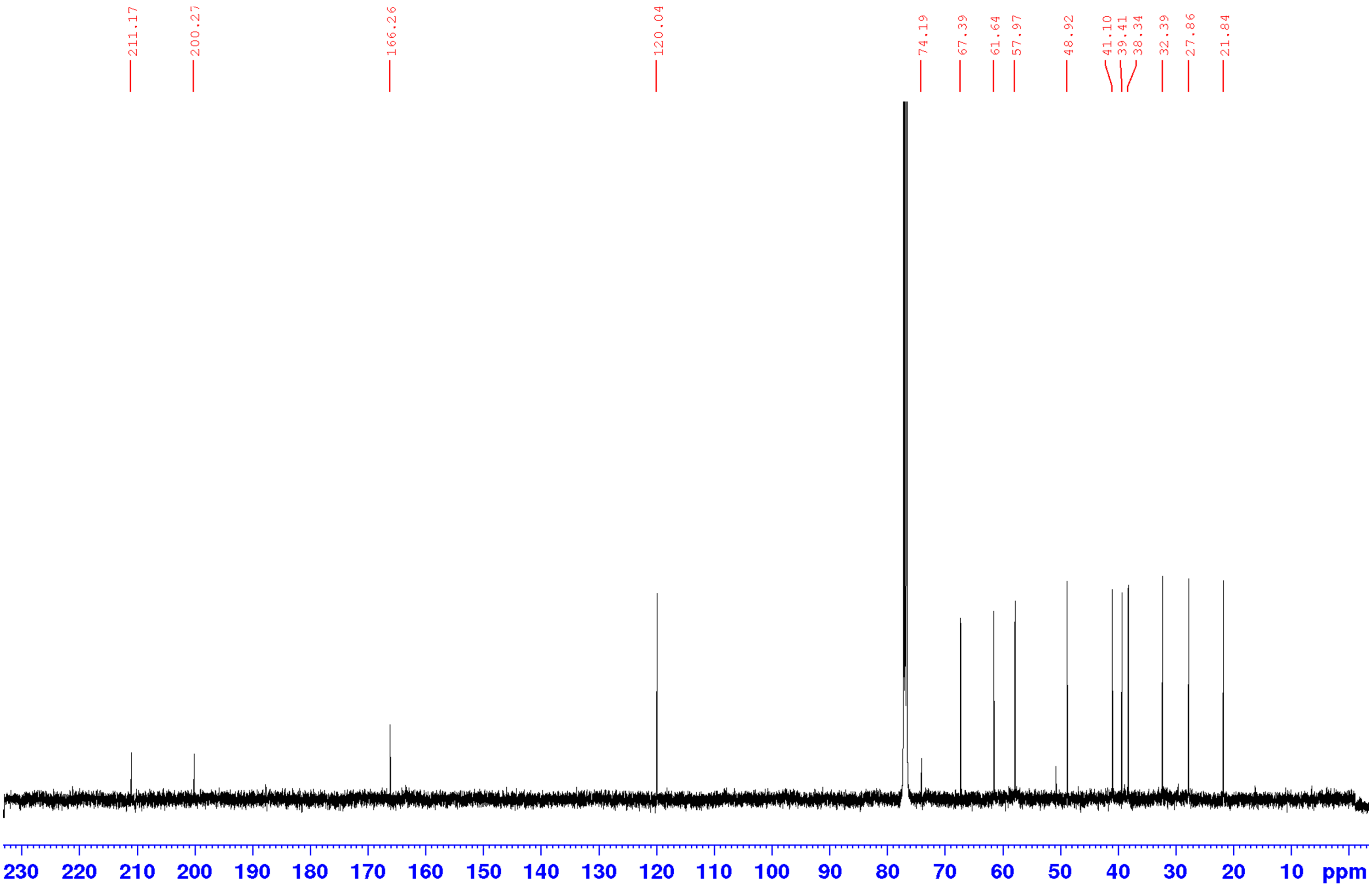

Figure S104. DEPT-135 spectrum (125.77 MHz) of **11** in $CDCl_3$

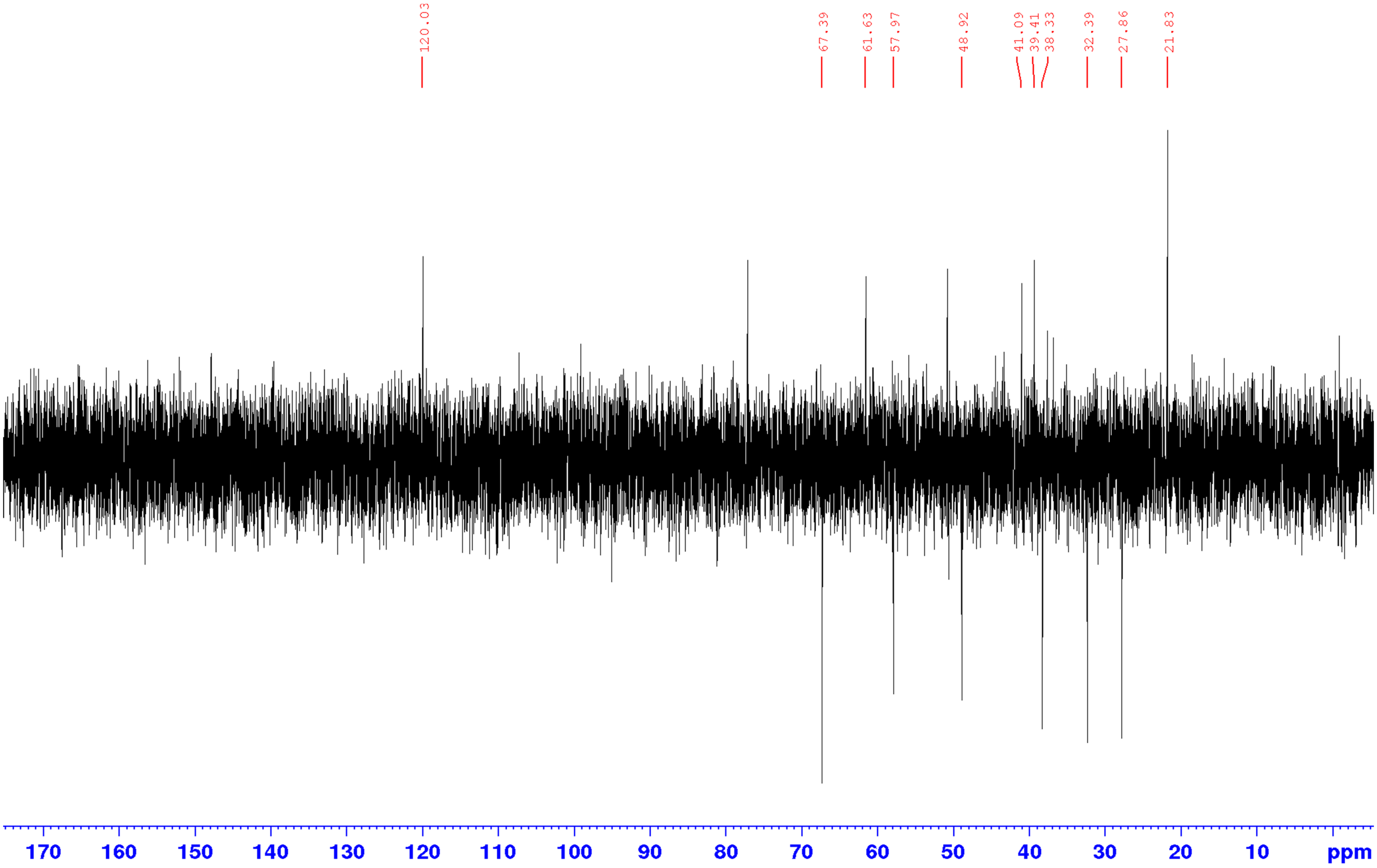

Figure S105. DEPT-90 spectrum (125.77 MHz) of **11** in $CDCl_3$

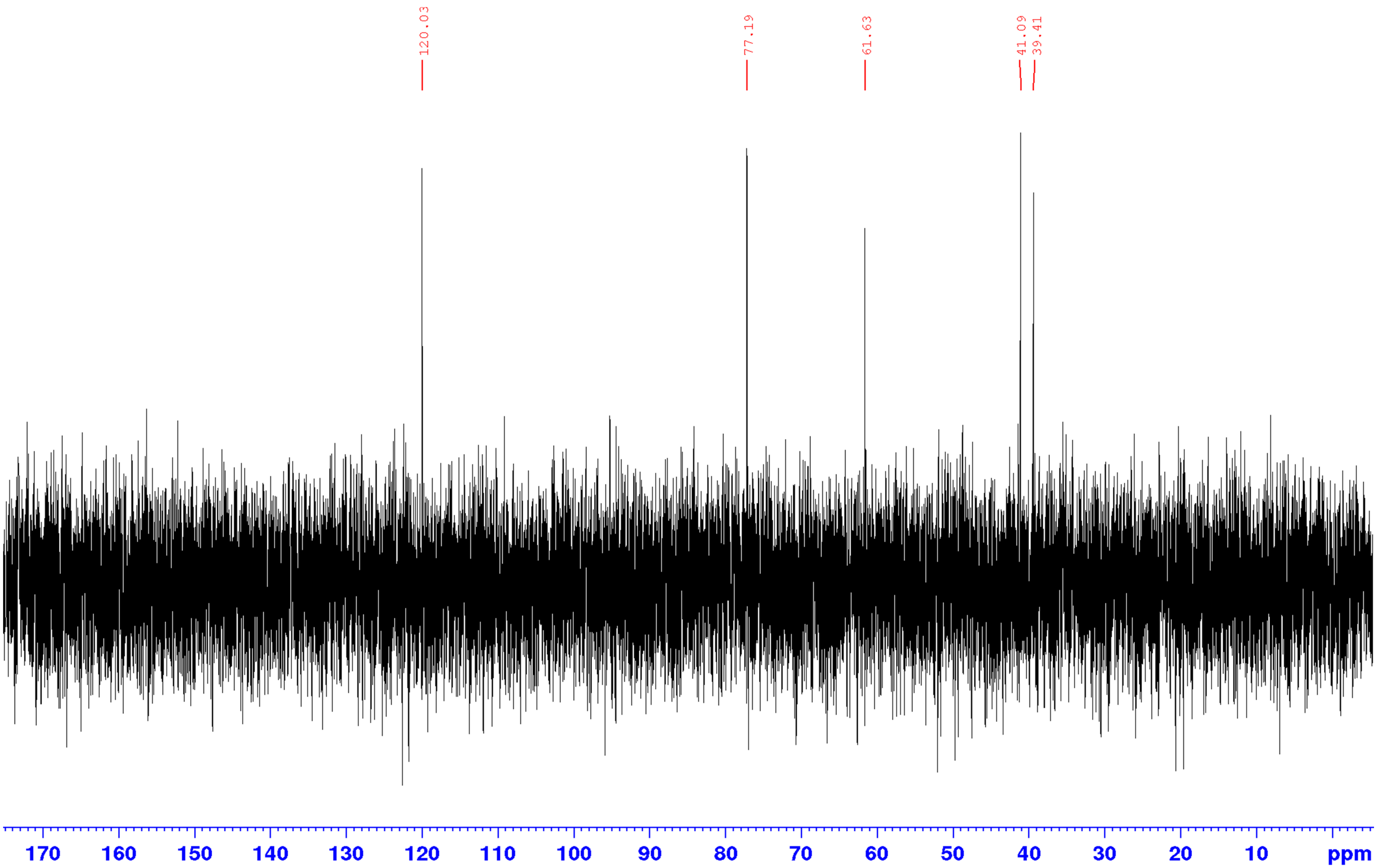

Figure S106. HSQC spectrum of **11** in $CDCl_3$

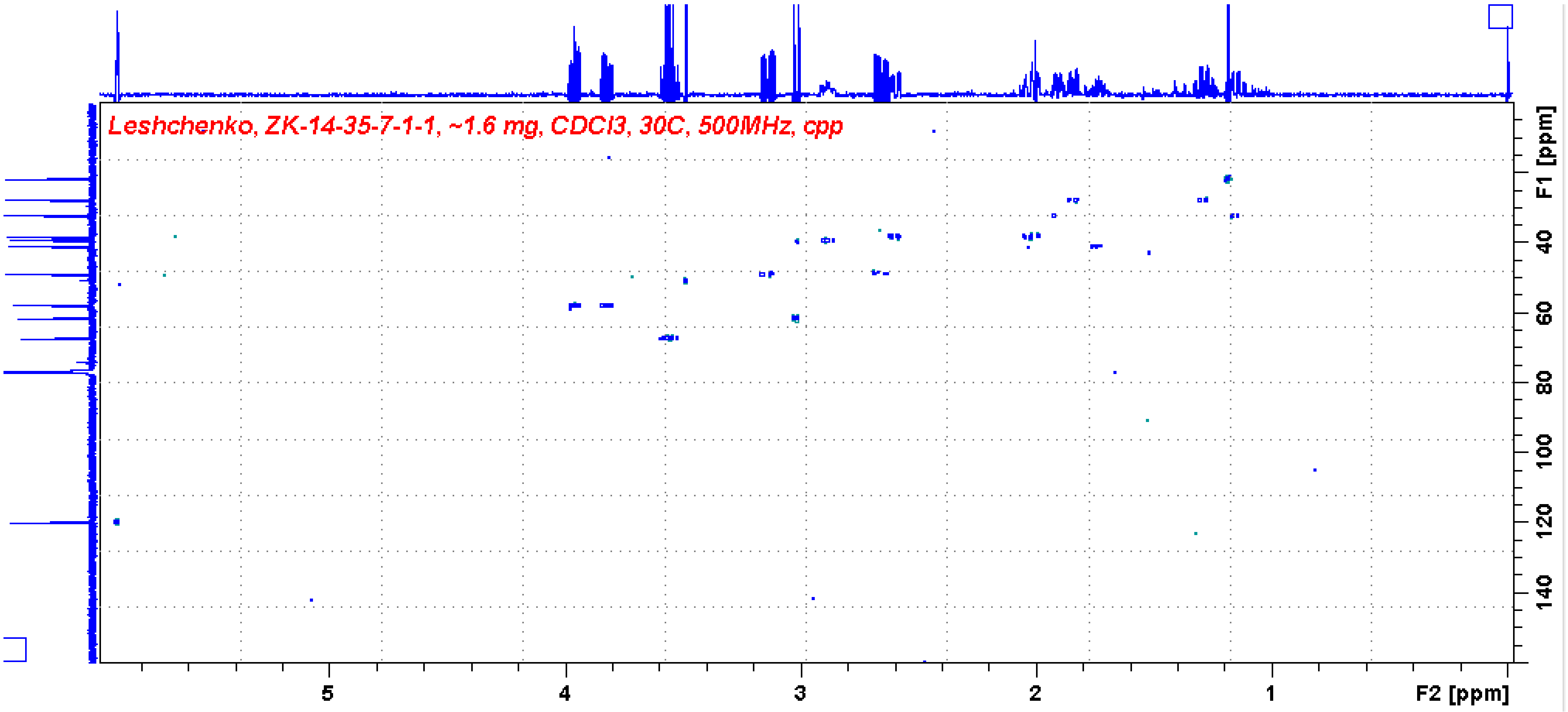

Figure S107. HMBC spectrum of **11** in $CDCl_3$

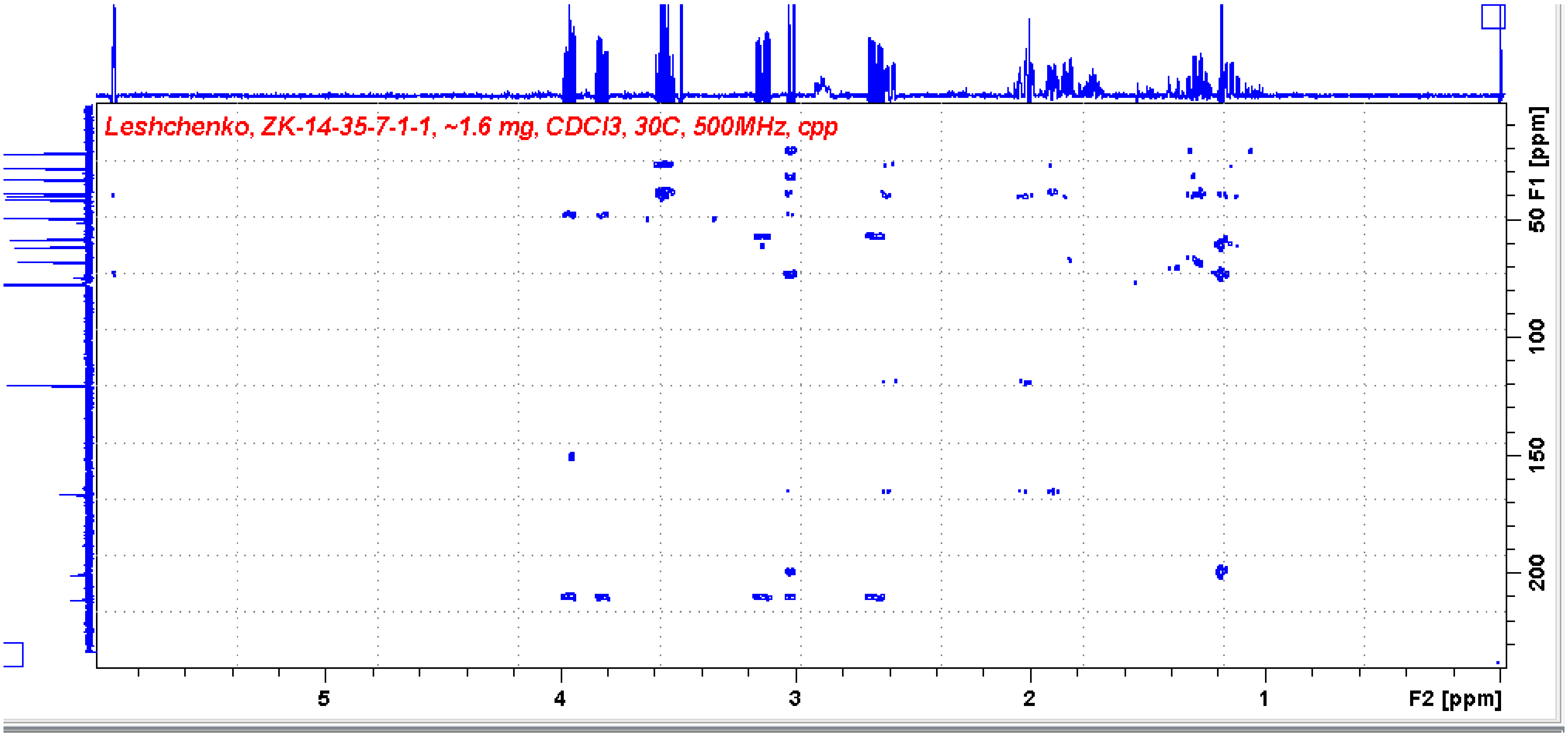

Figure S108. 1H-1H COSY spectrum of **11** in $CDCl_3$

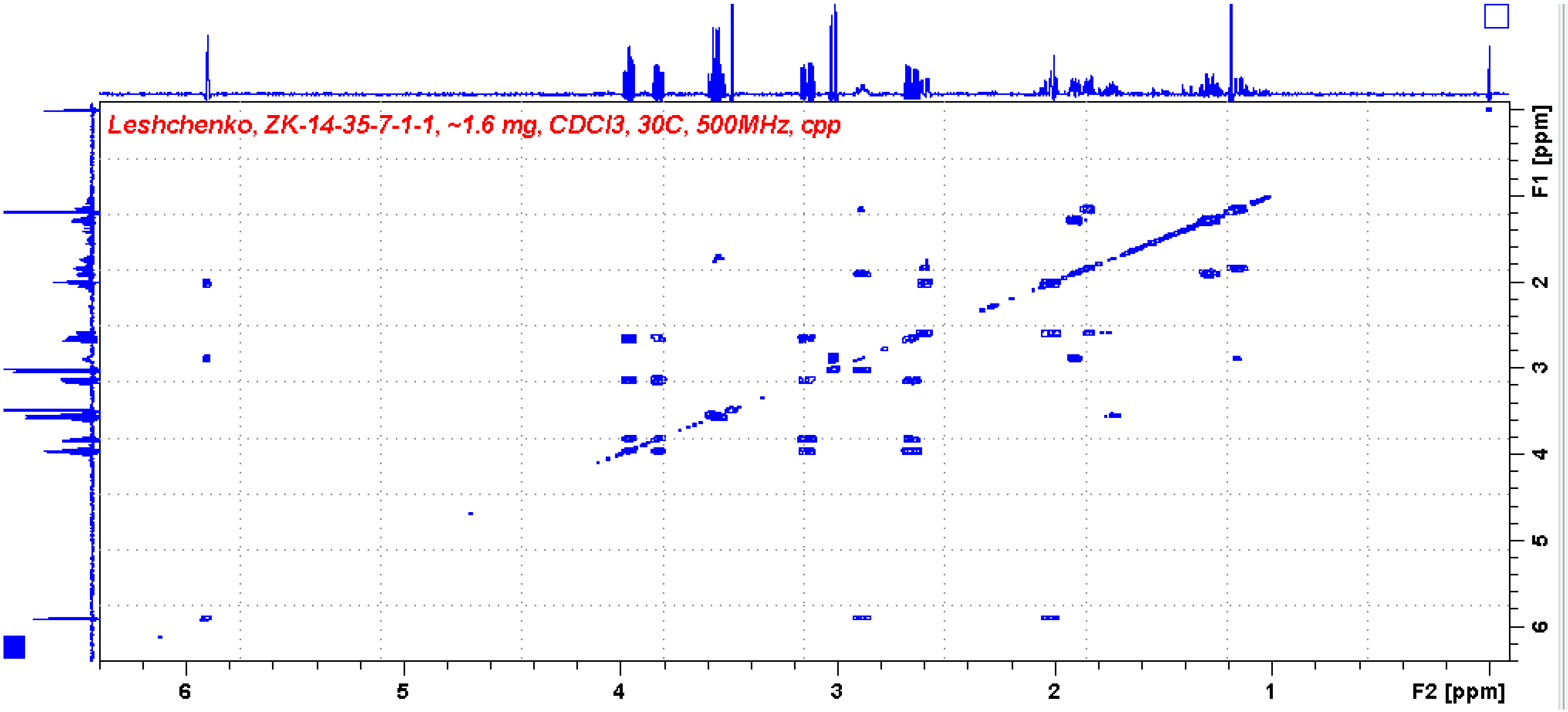

Figure S106. NOESY spectrum of **11** in $CDCl_3$

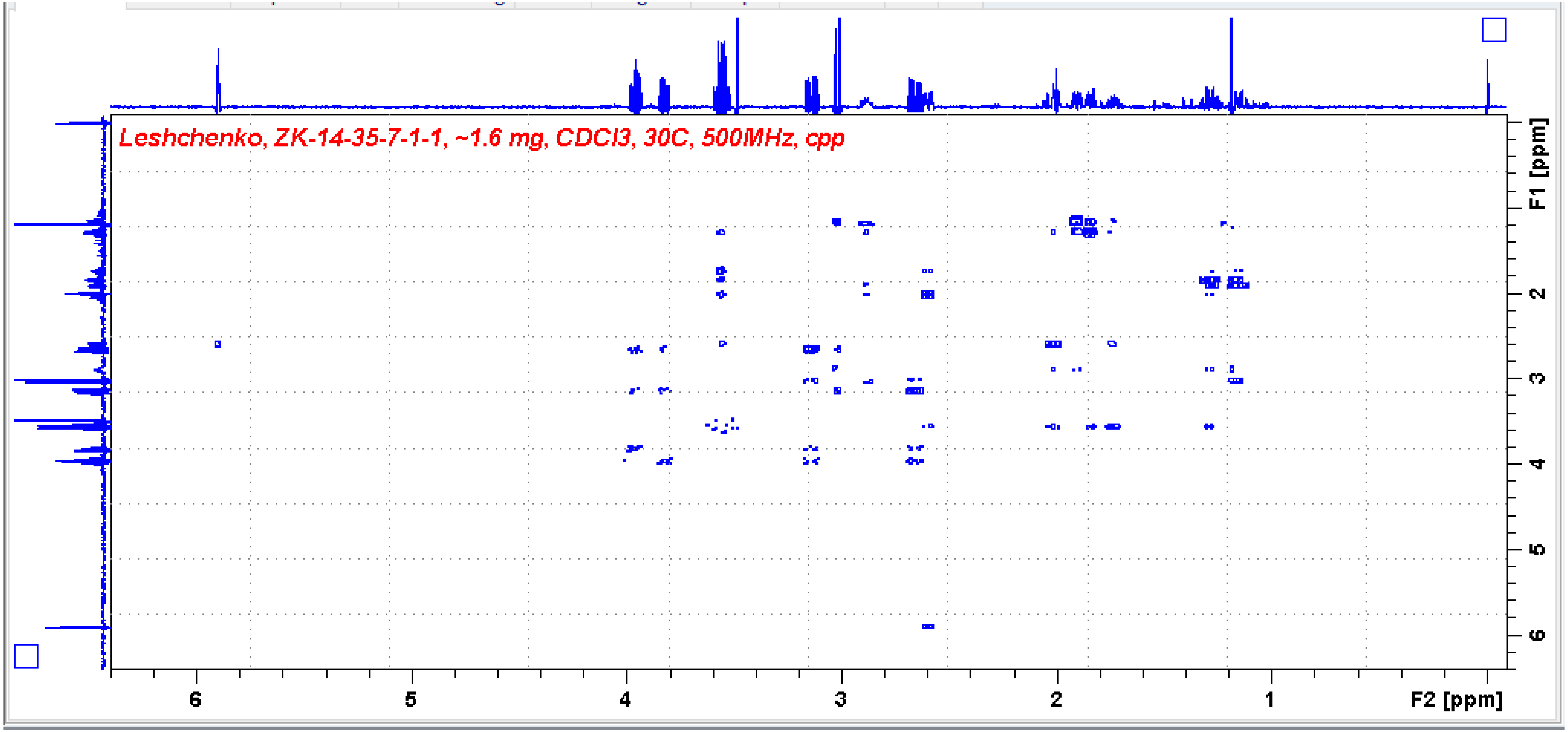

Figure S110. ^{1}H NMR spectrum (700.13 MHz) of **12** in $CDCl_3$

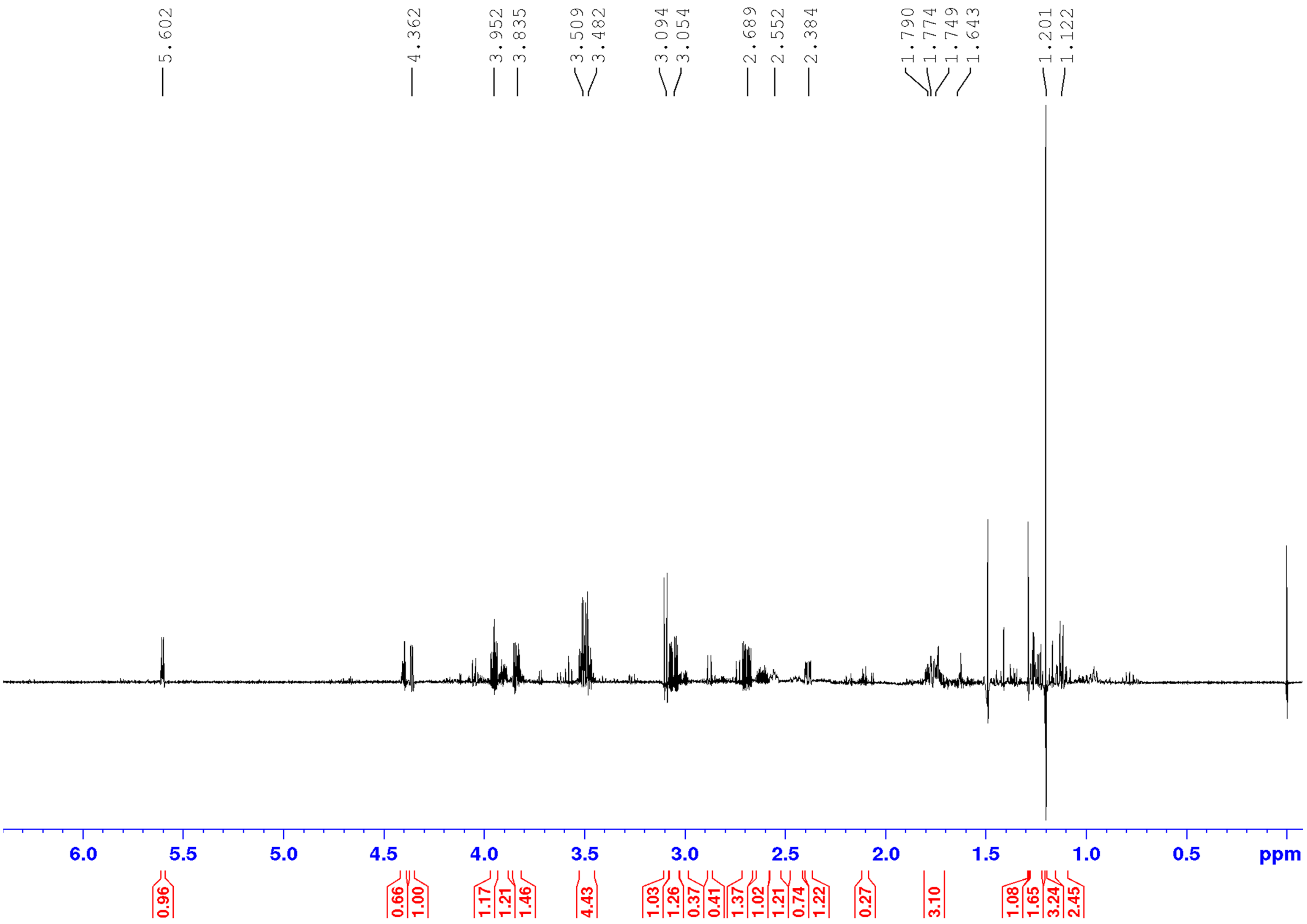

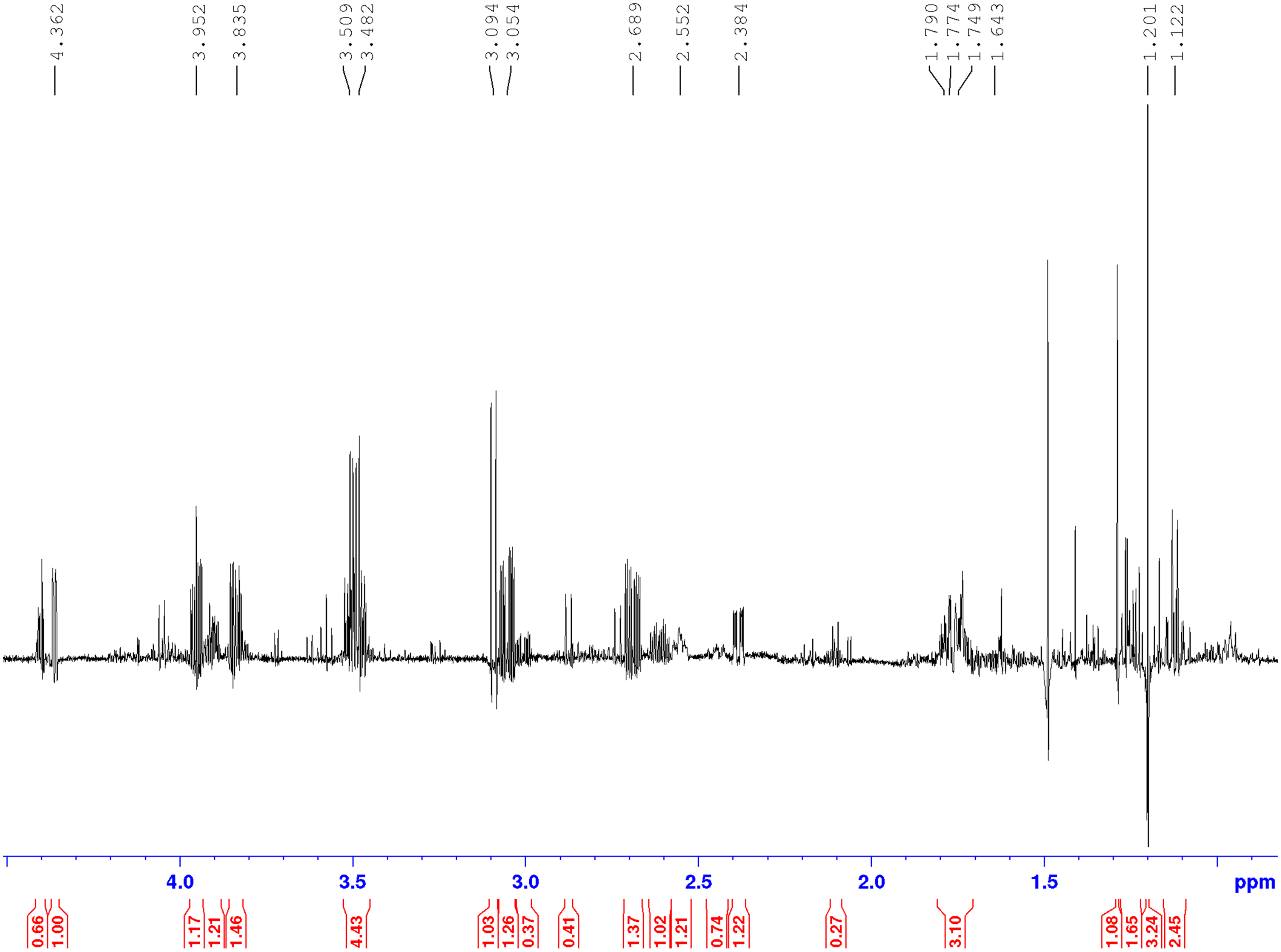
4.362
3.952
3.835
3.509
3.482
3.094
3.054
2.689
2.552
2.384
1.790
1.774
1.749
1.643
1.201
1.122
4.0
3.5
3.0
2.5
2.0
1.5
ppm
0.66
1.00
1.17
1.21
1.46
4.43
1.03
1.26
0.37
0.41
1.37
1.02
1.21
0.74
1.22
0.27
3.10
1.08
1.65
3.24
2.45

Figure S111. ^{13}C NMR spectrum (125.77 MHz) of **12** in $CDCl_3$

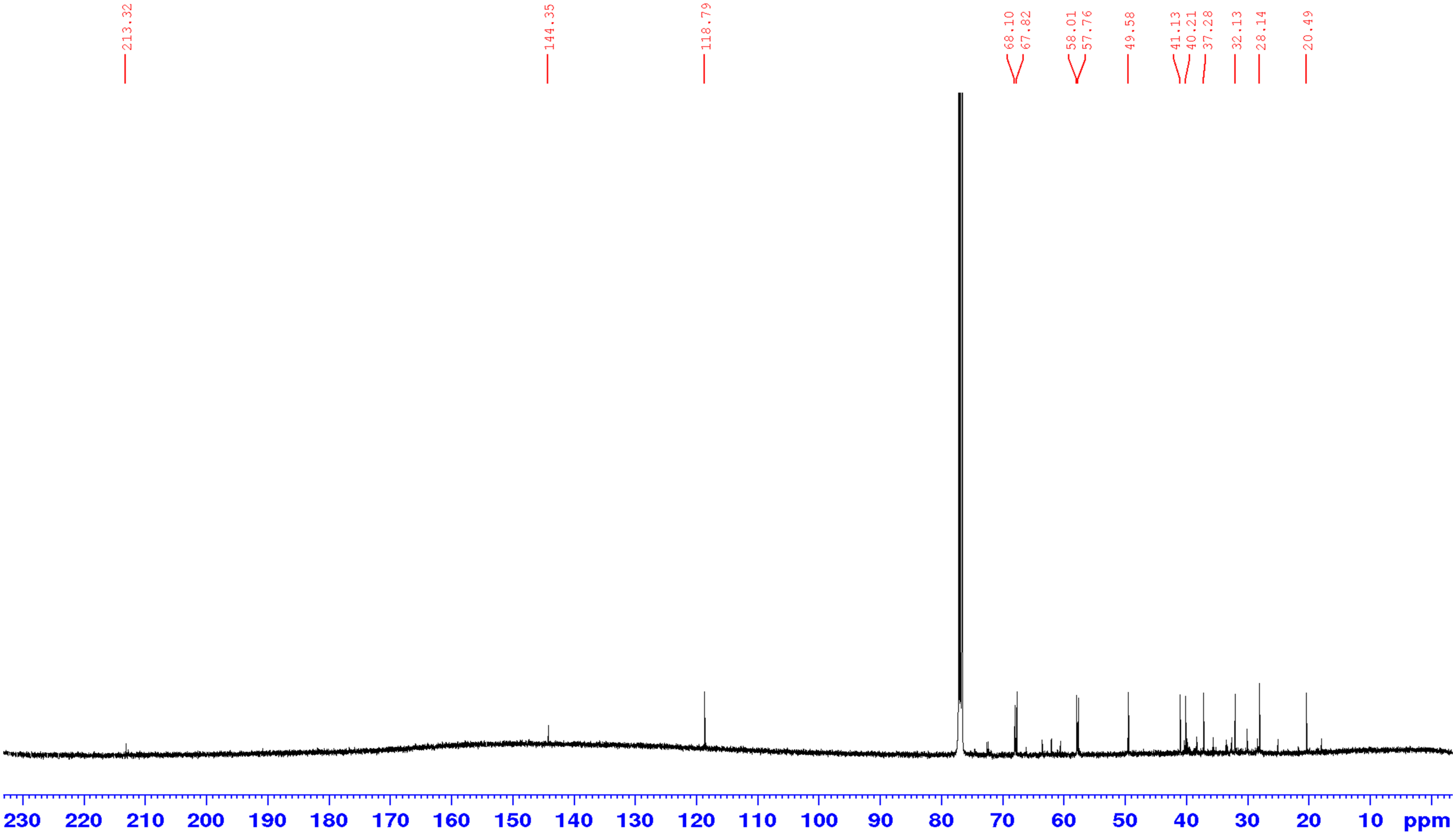

Figure S112. DEPT-135 spectrum (125.77 MHz) of **12** in $CDCl_3$

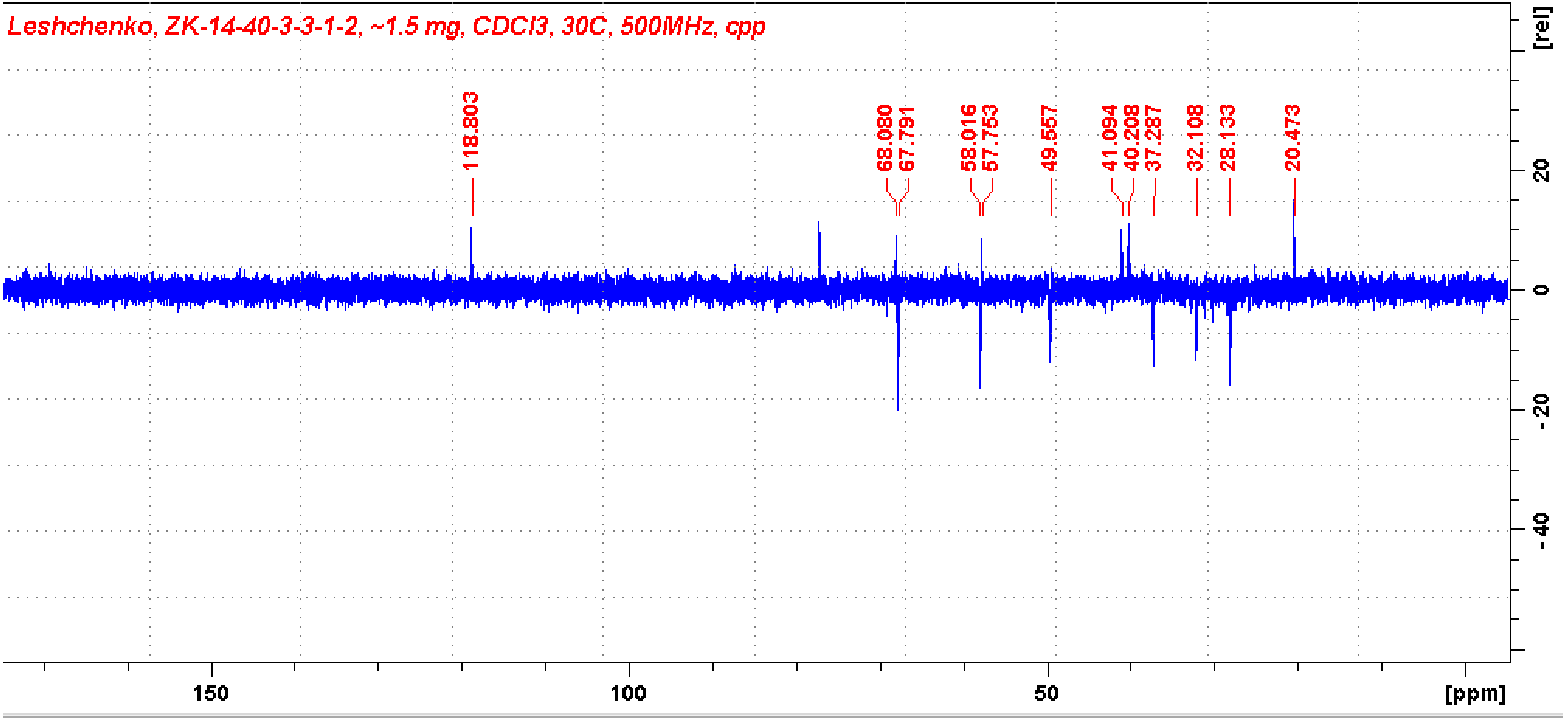

Figure S113. DEPT-90 spectrum (125.77 MHz) of **12** in $CDCl_3$

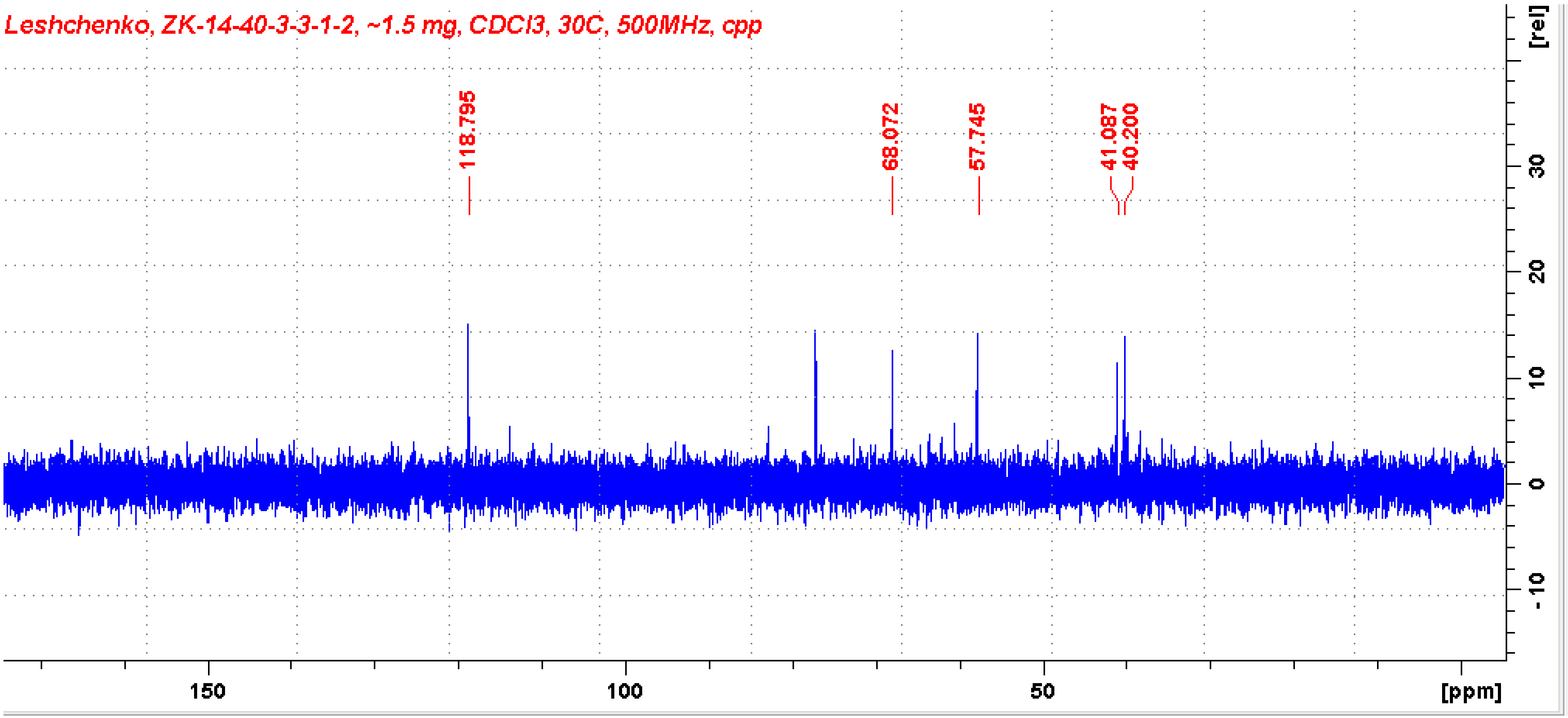

Figure S114. HSQC spectrum of **12** in $CDCl_3$

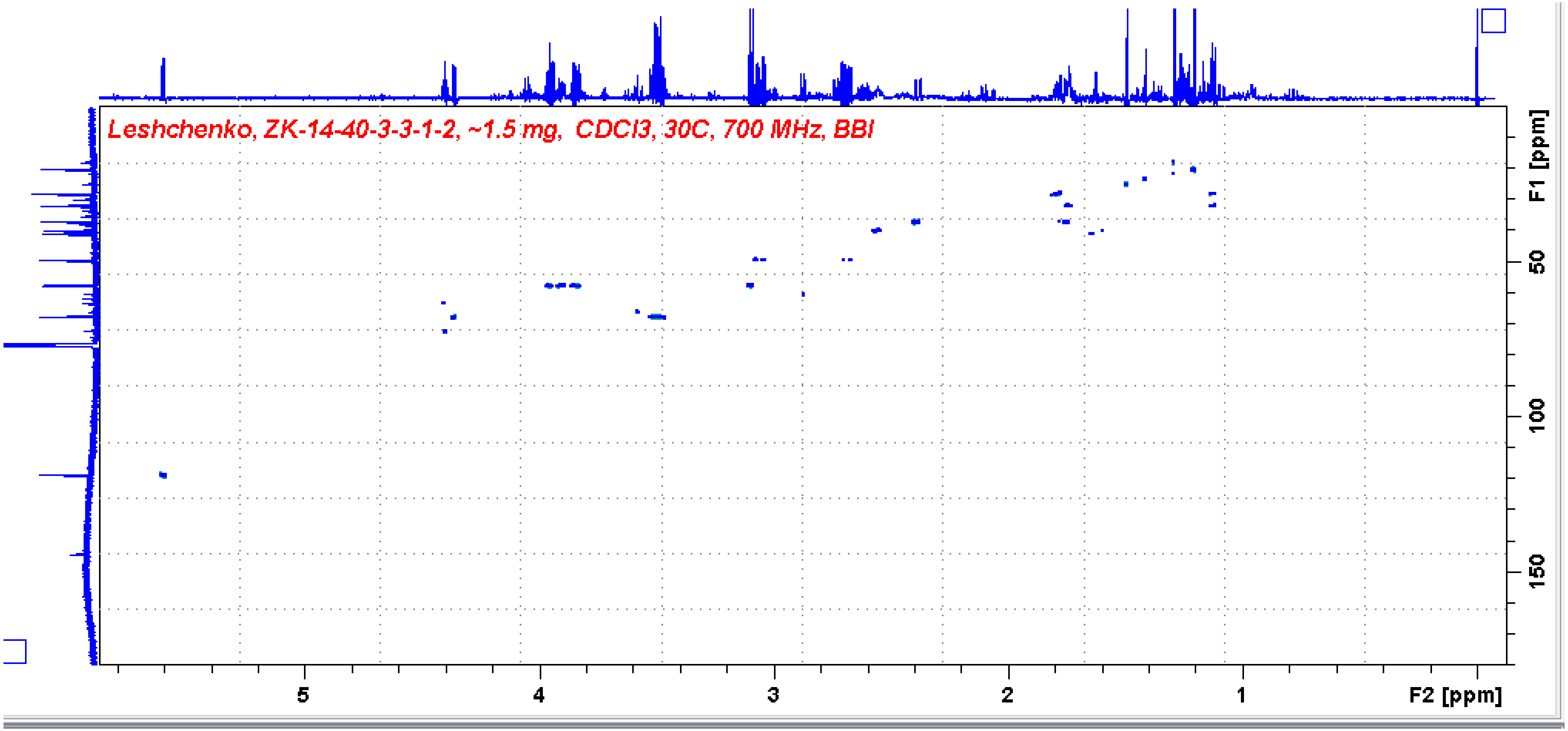

Figure S115. HMBC spectrum of **12** in $CDCl_3$

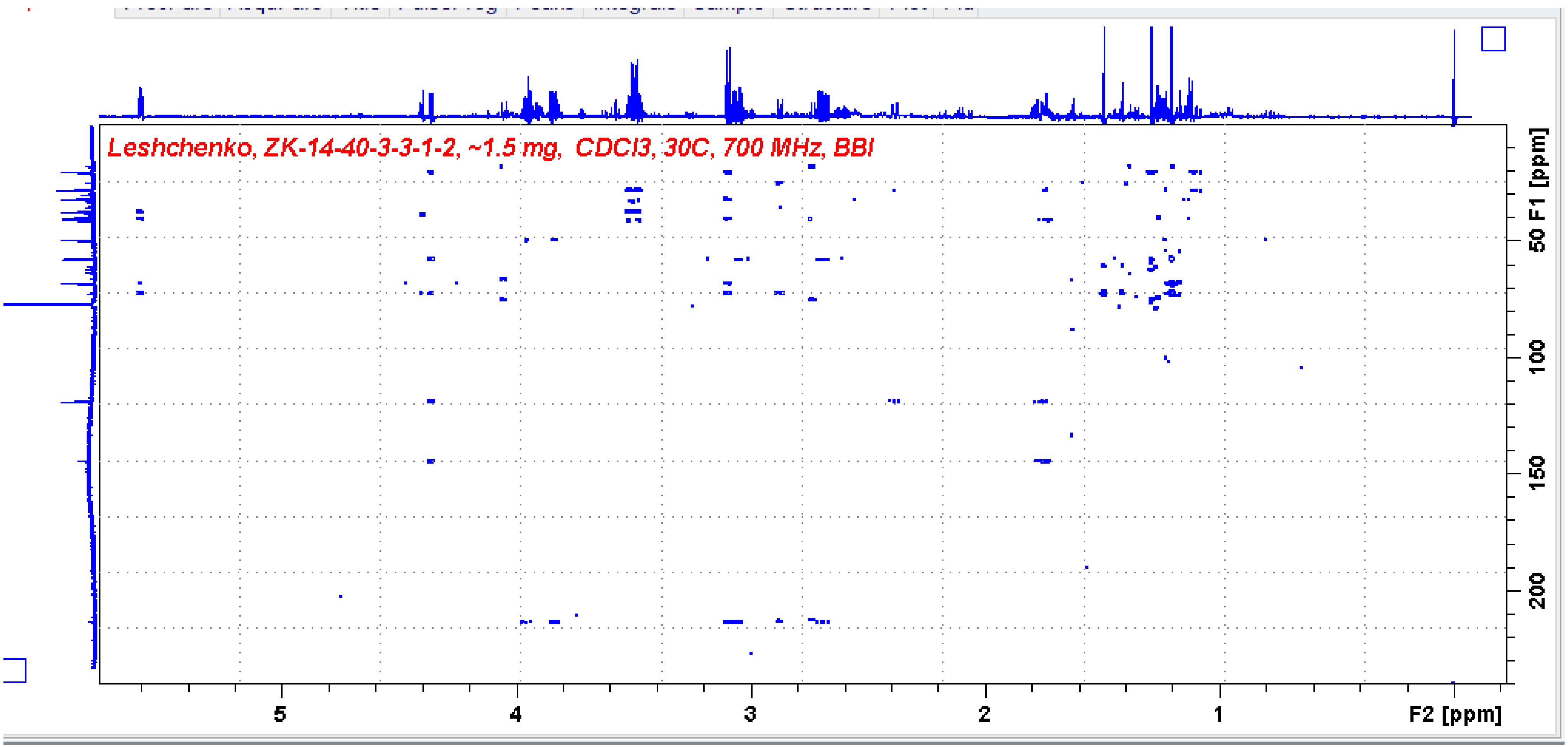

Figure S116. 1H-1H COSY spectrum of **12** in $CDCl_3$

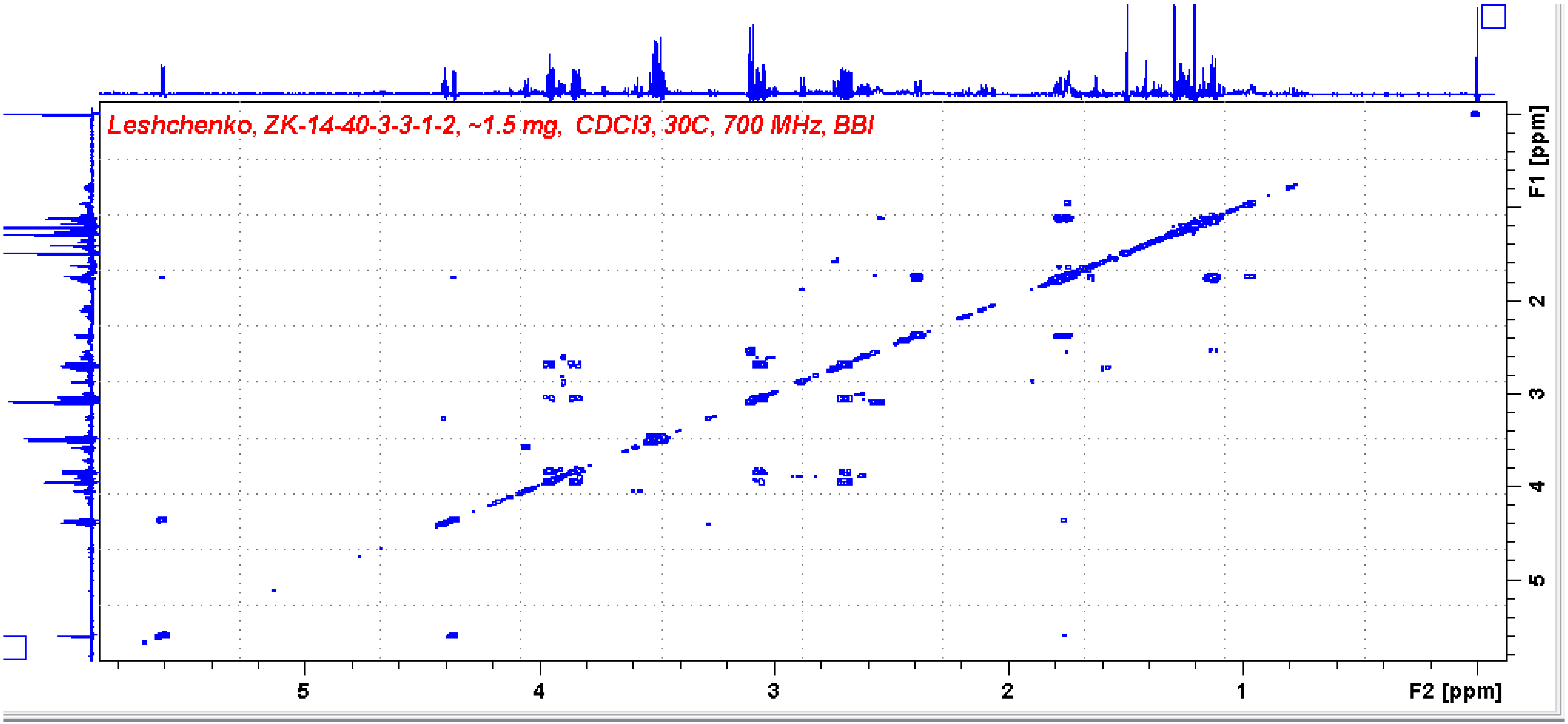

Figure S117. NOESY spectrum of **12** in $CDCl_3$

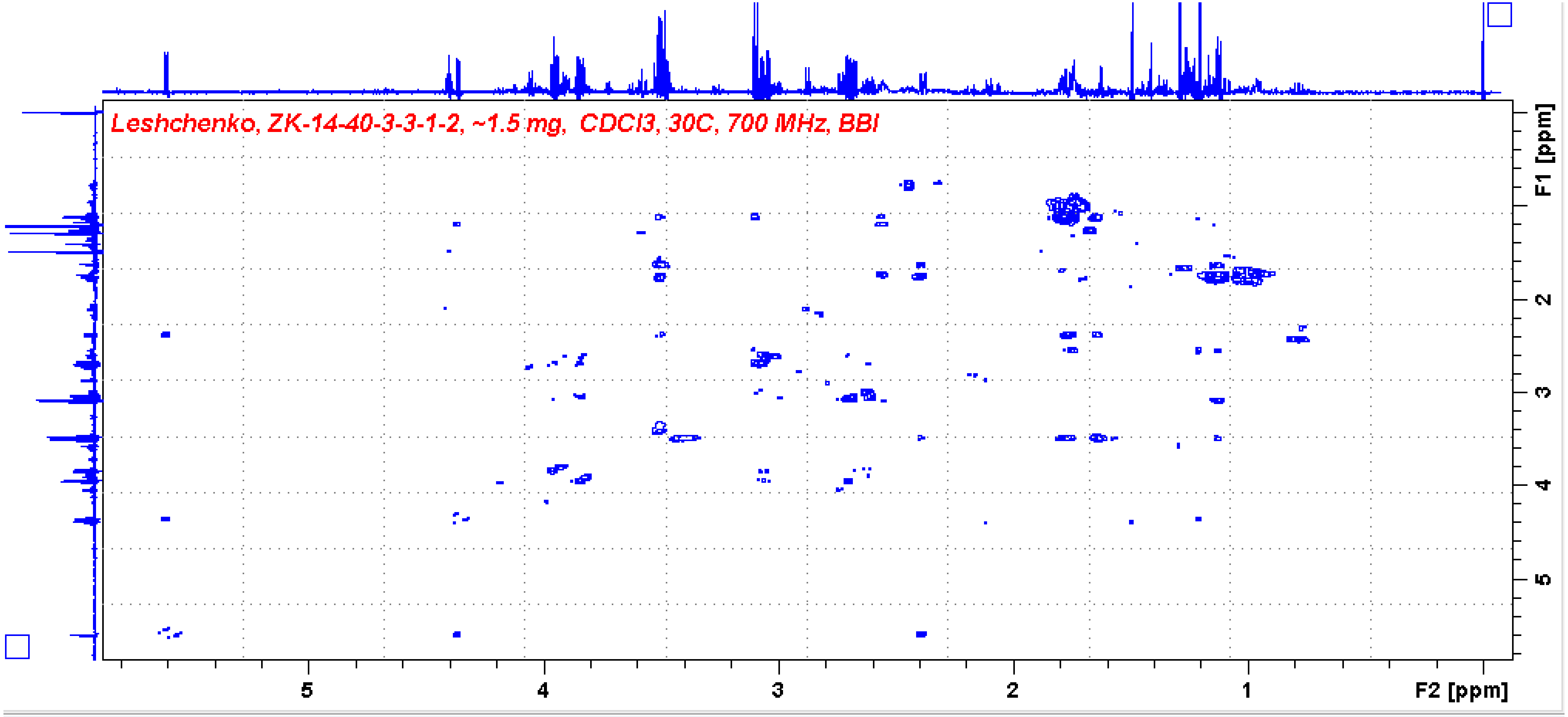

Supplement: Supplementary file 1 [file marinedrugs-15-00046-s001.pdf]
